# Risk Factors for the Development of Food Allergy in Infants and Children: A Systematic Review and Meta-Analysis

**DOI:** 10.1001/jamapediatrics.2025.6105

**Published:** 2026-02-09

**Authors:** Nazmul Islam, Alexandro W. L. Chu, Falana Sheriff, Farid Foroutan, Gordon H. Guyatt, Romina Brignardello-Petersen, Paul Oykhman, Alfonso Iorio, Ariel Izcovich, Katherine M. Morrison, Yetiani Roldan Benitez, Rachel J. Couban, Dorota Borovsky, Yiming Zhang, Leonardo Ologundudu, Keerthana Pasumarthi, Syed Fahad Farooq, Kyle Tong, Wang-Choi Tang, Haseeb Faisal, Muhammad Faran Khalid, Mohammad Saad Asif, Shannon French, Susan Waserman, R. Sharon Chinthrajah, Hugh A. Sampson, S. Shahzad Mustafa, Jay A. Lieberman, Kirsi M. Järvinen, Sally Bailey, Philippe Bégin, Scott H. Sicherer, Jennifer Gerdts, Melanie Carver, Lynda Mitchell, Kelly Cleary, Matthew J. Greenhawt, Julie Wang, Aikaterini Anagnostou, Marcus S. Shaker, Anita Chandra-Puri, Patricia C. Fulkerson, Robert A. Wood, Derek K. Chu

**Affiliations:** 1 Evidence in Allergy Group, McMaster University, Hamilton, Ontario, Canada; 2 Department of Health Research Methods, Evidence and Impact, McMaster University, Hamilton, Ontario, Canada; 3 Department of Medicine, McMaster University, Hamilton, Ontario, Canada; 4 Department of Medicine, University of Toronto, Toronto, Ontario, Canada; 5 Department of Pediatrics, McMaster University, Hamilton, Ontario, Canada; 6 Faculty of Medicine, Universidad del Salvador, Buenos Aires, Argentina; 7 Department of Anesthesia, McMaster University, Hamilton, Ontario, Canada; 8 Michael G. DeGroote School of Medicine, McMaster University, Hamilton, Ontario, Canada; 9 Faculty of Medicine, University of Ottawa, Ottawa, Ontario, Canada; 10 Sean N. Parker Center for Allergy and Asthma Research, Stanford University, Palo Alto, California; 11 Jaffe Food Allergy Institute, Division of Pediatric Allergy and Immunology, Department of Pediatrics, Icahn School of Medicine at Mount Sinai, New York, New York; 12 Department of Medicine, Rochester Regional Health, Rochester, New York; 13 Division of Allergy, Immunology, and Rheumatology, University of Rochester School of Medicine and Dentistry, Rochester, New York; 14 Division of Pulmonology, Allergy and Immunology, Department of Pediatrics, The University of Tennessee Health Science Center, Le Bonheur Children’s Hospital, Memphis; 15 Center for Food Allergy, Division of Allergy and Immunology, Department of Pediatrics, University of Rochester Medical Center, Rochester, New York; 16 Division of Pediatric Allergy and Immunology, Department of Pediatrics, Georgetown University, Washington, DC; 17 Centre Hospitalier Universitaire Sainte-Justine, Montréal, Québec, Canada; 18 Jaffe Food Allergy Institute, Serena and John Liew Division of Pediatric Allergy and Immunology, Icahn School of Medicine at Mount Sinai, New York, New York; 19 Food Allergy Canada, Toronto, Ontario, Canada; 20 The Asthma and Allergy Foundation of America, Arlington, Virginia; 21 Allergy and Asthma Network, Fairfax, Virginia; 22 Food Allergy Research and Education, McLean, Virginia; 23 Division of Allergy and Immunology, Department of Pediatrics, Baylor College of Medicine, Houston, Texas; 24 Department of Pediatrics and Department of Medicine, Geisel School of Medicine at Dartmouth, Lebanon, New Hampshire; 25 Section of Allergy and Immunology, Dartmouth Hitchcock Medical Center, Lebanon, New Hampshire; 26 Division of Community-Based Primary Care, Department of Pediatrics, Northwestern University Feinberg School of Medicine, Chicago, Illinois; 27 US National Institute of Allergy and Infectious Diseases, Bethesda, Maryland; 28 Division of Allergy and Immunology, Department of Pediatrics, Johns Hopkins University School of Medicine, Baltimore, Madison; 29 The Research Institute of St Joe’s Hamilton, St Joseph’s Healthcare Hamilton, Hamilton, Ontario, Canada

## Abstract

**Importance:**

The incidence and risk (predictive) factors for early life food allergy development remain uncertain.

**Objective:**

To estimate the incidence and quantify risk factors for food allergy development.

**Data Sources:**

MEDLINE and Embase were systematically searched to January 1, 2025. Data were analyzed from June 1, 2025, to November 25, 2025.

**Study Selection:**

Incidence estimates included studies confirming food allergy via food challenge. Risk factor analyses included cohort, case-control, and cross-sectional studies in any language assessing children younger than 6 years using multivariable analyses.

**Data Extraction and Synthesis:**

Paired reviewers independently extracted data. Random-effects meta-analyses pooled incidence and adjusted odds ratios (ORs). Risk of bias was assessed using the QUIPS tool, and certainty of evidence assessed using GRADE.

**Main Outcome and Measure:**

The primary outcome was food allergy to age 6 years.

**Results:**

A total of 190 studies involving 2.8 million participants across 40 countries were analyzed. Among studies using food challenge, overall food allergy incidence was likely 4.7% (moderate certainty). Among 176 studies identifying 342 risk factors with varying certainty, the strongest and most certain factors included prior allergic conditions (eg, atopic dermatitis [eczema] within the first year of life [OR, 3.88; risk difference [RD], 12.0%; 95% CI, 8.8%-15.7%], allergic rhinitis [OR, 3.39; RD, 10.1%; 95% CI, 6.7%-14.4%], and wheeze [OR, 2.11; RD, 5.0%; 95% CI, 2.1%-8.8%]), severity of atopic dermatitis (OR, 1.22; RD, 1.0%; 95% CI, 0.6%-1.6%), increased skin transepidermal water loss (OR, 3.36; RD, 10.0%; 95% CI, 6.3%-14.8%), filaggrin gene sequence variations (OR, 1.93; RD, 4.2%; 95% CI, 2.4%-6.4%), delayed solid food introduction (eg, peanut after age 12 months [OR, 2.55; RD, 6.8%; 95% CI, 1.9%-14.6%]), infant antibiotic use (first month [OR, 4.11; RD, 12.8%; 95% CI, 0.4%-40%], first year [OR, 1.39; RD, 1.8%; 95% CI, 0.8%-3.1%], during pregnancy [OR, 1.32; RD, 1.5%; 95% CI, 0.6%-2.5%]), male sex (OR, 1.24; RD, 1.1%; 95% CI, 0.7%-1.6%), firstborn child (OR, 1.13; RD, 0.6%; 95% CI, 0.3%-1.0%), family history of food allergy (eg, mother [OR, 1.98; RD, 4.4%; 95% CI, 2.5%-6.8%], father [OR, 1.69; RD, 3.2%; 95% CI, 1.3%-5.5%], both parents [OR, 2.07; RD, 4.8%; 95% CI, 1.3%-5.5%], siblings [OR, 2.36; RD, 6.0%; 95% CI, 4.4%-8.0%]), parental migration (OR, 3.28; RD, 9.7%; 95% CI, 4.9%-16.3%), self-identification as Black (vs White [OR, 3.93; RD, 12.1%; 95% CI, 5.2%-22.5%], vs non-Hispanic White [OR, 2.23; RD, 5.5%; 95% CI, 3.0%-8.7%]), and cesarean delivery (OR, 1.16; RD, 1.0%; 95% CI, 0.3%-1.2%). Factors like low birth weight, postterm birth, maternal diet, and stress during pregnancy showed no significant risk difference.

**Conclusions and Relevance:**

In this meta-analysis, the most credible risk factors associated with development of childhood food allergy are a combination of major and minor risk factors, including early allergic conditions (atopic march/diathesis), delayed allergen introduction, genetics, antibiotic exposure, demographic factors, and birth-related variables.

## Key Points

**Question:**

What are the risk factors associated with the development of food allergy in children?

**Findings:**

This systematic review and meta-analysis of 2.8 million participants in 190 studies identified the following largest and most certain risk factors associated with the development of food allergies in children: prior allergic conditions (atopic march/diathesis), atopic dermatitis, increased skin transepidermal water loss, filaggrin gene sequence variations, delayed solid food introduction, infant and intrapartum antibiotic exposure, male sex, being first born, family history of allergy, parental migration, self-identification as Black, and cesarean delivery.

**Meaning:**

This systematic review and meta-analysis clarifies the major and minor risk factors associated with developing early-onset food allergy to inform optimal prevention clinical practice, policy, and research.

## Teaser

This systematic review and meta-analysis estimates the incidence of and quantifies risk factors for food allergy development in children.

## Introduction

Food allergy is a growing global health burden and in the US alone affects more than 33 million people.^1,2,3^ Immunoglobulin E (IgE)–mediated food allergy, the most common type of food allergy, often develops early in life, lasts a lifetime, and can cause acute, life-threatening reactions called anaphylaxis.^4^ Understanding why food allergy is rising and predicting who will or will not develop it remains challenging, as there is no systematic evidence-based consensus on at-risk populations to target for prevention.^5,6^

Despite the need for evidence-based food allergy prevention strategies, the incidence of food allergy and the risk factors for developing it remain uncertain.^5^ Risk factors help identify individuals likely to develop an outcome and, as associations, may or may not be causal. Incidence and risk factors are closely linked, as quantifying incidence is critical to interpreting the absolute risk of each identified risk factor. Ongoing uncertainty leaves parents, clinicians, policymakers, and researchers without clear guidance on recognizing children at high risk or on influencing modifiable factors for allergy prevention. Therefore, we systematically reviewed and synthesized the incidence of and risk factors for the development of food allergy in infants and children.

## Methods

We followed the Grading of Recommendations Assessment, Development, and Evaluation (GRADE), Cochrane, Preferred Reporting Items for Systematic Reviews and Meta-Analyses (PRISMA), and Meta-Analysis of Observational Studies in Epidemiology (MOOSE) guidance for conducting and reporting this systematic review (eMethods 1 in Supplement 1).^7,8,9,10^ The protocol was registered prospectively (PROSPERO: CRD42021282358). The eMethods 2 in Supplement 1 present additional methods details. The CHILD cohort study was approved by research ethics boards at each recruitment site and the Hamilton Integrated Research Ethics Board.

### Data Sources and Search Strategy

Supported by an information specialist (R.J.C.), MEDLINE (from 1946) and Embase (from 1974) were systematically searched through January 1, 2025 (eMethods 3 in Supplement 1). Forward and backward citation analyses were additionally performed using Web of Science (all databases).

### Study Selection

To assess risk (ie, predictive) factors, cohort, case-control, and cross-sectional studies published in any language evaluating 1 or more factors for the development of IgE-mediated food allergy (accepting the definitions within the Outcomes section) in children 6 years of age or younger that used multivariable-adjusted analyses at least including age or sex were included. To estimate food allergy incidence, only studies that confirmed food allergy through food challenge(s) were included. Whether assessing risk factors or incidence, studies addressing posttransplant populations or alpha-gal food allergy were excluded.

Paired reviewers (among 14 reviewers) independently screened titles and abstracts and, subsequently, full texts of potentially eligible studies using Covidence (Veritas Health Innovation). The reviewers discussed and resolved disagreements by consensus, and, if necessary, the senior investigator (D.K.C.) adjudicated.

### Data Extraction

Consistent with routine guidance from the Cochrane Handbook^11^ and previous American Academy of Allergy, Asthma & Immunology (AAAAI)/American College of Allergy, Asthma & Immunology (ACAAI) Joint Task Force systematic reviews,^12,13,14^ the same pairs of reviewers independently extracted data using a standardized data extraction form. Calibration during screening and extraction involved completing the first 10 studies, discussing discrepancies, and refining consistency before proceeding with the remaining studies. Information regarding study characteristics, exposures, and outcomes, such as adjusted odds ratios (ORs) and 95% confidence intervals for each risk factor, were extracted. Study definitions of sex, gender, race, and ethnicity were used.

### Outcomes

Food allergy diagnostic definitions were stratified based on whether a food challenge was conducted. If no food challenge was performed, the diagnosis was further stratified based on whether they were derived from skin testing alone, a combination of blood and skin testing and clinical history, or clinical history alone. In all cases, we ensured consistency with definitions of IgE-mediated food allergy as outlined by the AAAAI/ACAAI Joint Task Force on Practice Parameters^15,16^ and the US National Academies of Sciences, Engineering, and Medicine.^2^

### Risk of Bias Assessment

Paired reviewers assessed risk of bias independently for each outcome using the Quality in Prognosis Studies (QUIPS) tool for predictive factor studies.^17^ The tool’s 6 domains address study participation, attrition, prognostic factor measurement, outcome measurement, study confounding, and statistical analysis. The prognostic factor measurement and study confounding domains did not apply when addressing incidence.

### Certainty of Evidence Assessment

We rated the overall certainty (ie, quality) of evidence for each risk factor as high, moderate, low, or very low using the GRADE approach.^10,18^ GRADE domains include risk of bias, imprecision, inconsistency, indirectness, and publication bias, as well as factors that enhance the certainty of the evidence (eg, large effects). Heterogeneity (between-study variability of estimates) was assessed according to the GRADE approach.^10,19,20^ Consistent with established AAAAI/ACAAI Joint Task Force on Practice Parameter trustworthy guideline development methods,^21,22,23^ this study’s linked multistakeholder guideline development group, including frontline clinicians (eg, pediatricians), allergy experts (eg, allergists-immunologists), patient and caregiver partners (people with food allergy and/or their family or caregivers), and methodologists, established a risk difference (RD) of 1% as a threshold for a minimally important difference increase, the smallest change that patients perceive as important^24,25^ in food allergy risk.

### Data Synthesis and Analysis

Logistic-normal random-effects meta-analysis using maximal likelihood estimation^26^ synthesized food allergy incidence. The mean and variance (τ) are pooled on the logit scale and then back-transformed to the proportion scale to facilitate interpretation. Exact (Clopper-Pearson) 95% confidence intervals were calculated.

For risk factor meta-analyses, random-effects generic inverse variance models pooled adjusted ORs with their 95% confidence intervals. If studies reported hazard ratios (HRs) or risk ratios (RRs), established methods^27^ were used to convert estimates to ORs using baseline risks (ie, incidence of food allergy in each respective study). The associations of high-, moderate-, and low-certainty factors with absolute effects that exceed the minimally important difference threshold of 1% for developing food allergy are summarized in forest plots.^24,25^

The following 3 a priori subgroup analyses^28^ were conducted for each outcome: (1) high vs low risk of bias, with studies at high risk of bias expected to show stronger associations; (2) food allergy definition, where studies using food challenges were anticipated to show weaker associations compared to those using other diagnostic tests (eg, skin testing, blood testing, self-report); and (3) study year (pre- vs post-2015), reflecting the change in recommendations to introduce food allergens early in life to prevent allergy.^29^ It was hypothesized that while the association between risk factors might remain similar across these periods, the baseline risk for developing food allergy could be lower in studies conducted after 2015 due to the change in food allergen introduction recommendations. Subgroup analyses were also conducted based on the number of foods participants were allergic to (single vs multiple). The credibility of subgroup effects was assessed using the following Instrument to assess the Credibility of Effect Modification Analyses (ICEMAN) criteria^30^: within- vs between-study comparisons, number of comparisons, a limited number of prespecified hypotheses, support by prior evidence, and statistical support and cutpoints. Analyses were performed using Stata version 16.1 (StataCorp).

## Results

**Figure 1.**
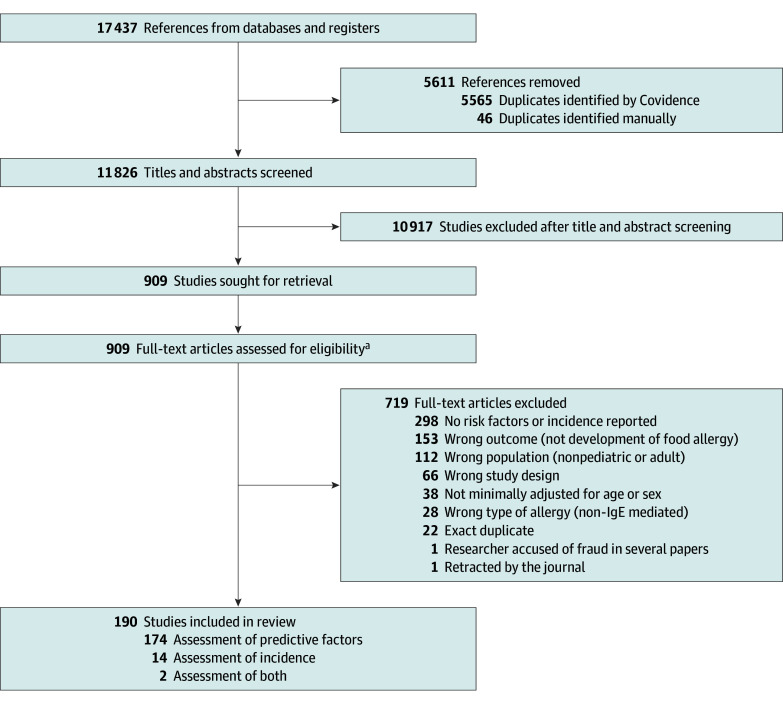
Flow Diagram of Study Selection in Systematic Review Addressing Food Allergy Incidence and Risk Factors ^a^For risk factor analysis, the following eligibility criteria were applied: observational studies (cohort, case-control, cross-sectional), children aged 6 years or younger, evaluated ≥1 risk factors for immunoglobulin E (IgE)–mediated food allergy, used multivariable-adjusted analyses (at least age or sex), and food allergy diagnosis based on clinical history, physician assessment, sensitization tests, or food challenge. For incidence estimation, the following eligibility criteria were applied: observational studies (cohort or cross-sectional), children aged 6 years or younger, and confirmed food allergy through food challenge.

The systematic search identified 11 826 unique records, from which 190^31,32,33,34,35,36,37,38,39,40,41,42,43,44,45,46,47,48,49,50,51,52,53,54,55,56,57,58,59,60,61,62,63,64,65,66,67,68,69,70,71,72,73,74,75,76,77,78,79,80,81,82,83,84,85,86,87,88,89,90,91,92,93,94,95,96,97,98,99,100,101,102,103,104,105,106,107,108,109,110,111,112,113,114,115,116,117,118,119,120,121,122,123,124,125,126,127,128,129,130,131,132,133,134,135,136,137,138,139,140,141,142,143,144,145,146,147,148,149,150,151,152,153,154,155,156,157,158,159,160,161,162,163,164,165,166,167,168,169,170,171,172,173,174,175,176,177,178,179,180,181,182,183,184,185,186,187,188,189,190,191,192,193,194,195,196,197,198,199,200,201,202,203,204,205,206,207,208,209,210,211,212,213,214,215,216,217,218,219,220^ studies were included, enrolling 2 750 495 participants (Figure 1). Of these, 156 were cohort studies,^31,33,34,35,36,37,38,40,41,42,44,45,46,47,48,50,51,52,53,54,55,57,58,59,60,62,63,64,65,67,68,69,70,71,72,73,75,76,77,78,79,80,82,83,84,85,86,87,88,90,93,94,95,96,97,98,99,101,102,103,104,105,106,107,108,109,110,111,112,113,114,115,116,117,118,119,120,121,122,123,124,125,126,127,128,129,130,131,132,133,135,136,137,138,139,140,141,142,143,144,145,146,147,148,150,153,154,155,157,159,160,161,162,168,170,171,172,173,174,175,176,179,181,182,183,184,185,186,188,190,191,192,193,194,195,196,197,198,201,203,204,205,206,207,208,210,211,212,213,214,215,216,217,218,219,220^ 22 were case-control studies,^32,43,49,56,61,66,81,89,91,92,100,149,158,164,165,167,169,178,180,187,189,199^ and 12 were cross-sectional studies.^39,74,134,151,152,156,163,166,177,200,202,209^ Among the included studies, 174 evaluated risk factors, 14 addressed incidence, and 2 addressed both.

The Table summarizes the characteristics of the included studies. The median (IQR) sample size was 1184 (459-2834) participants, with studies conducted across 40 countries in the following regions: Europe (36.8%), the Americas (27.9%), Asia (16.3%), Australia and New Zealand (15.3%), the Middle East (3.2%), and Africa (0.5%). The included studies were published between 1973 and 2024. eTable 1 in Supplement 1 presents the characteristics of each individual study.

**Table.** Summary of Characteristics of 190 Included Studies in Systematic Review Addressing Food Allergy Incidence and Risk Factors

Study characteristic	No. (%)
Study design	
Cohort	156 (82.1)
Case-control	22 (11.6)
Cross-sectional	12 (6.3)
No. of participants, median (IQR)	1184 (459-2834)
Regions	
Europe	70 (36.8)
Americas	53 (27.9)
Australia and New Zealand	29 (15.3)
Asia	31 (16.3)
Middle East	6 (3.2)
Africa	1 (0.5)

Overall, 125 studies (66.0%) were at high or probably high risk of bias for at least 1 criterion (eTable 2 in Supplement 1). The most frequent concern, detected in 65 studies, was measurement of prognostic factors, often due to inconsistent tools or methods across participants.

### Incidence of IgE-Mediated Food Allergy

**Figure 2.**
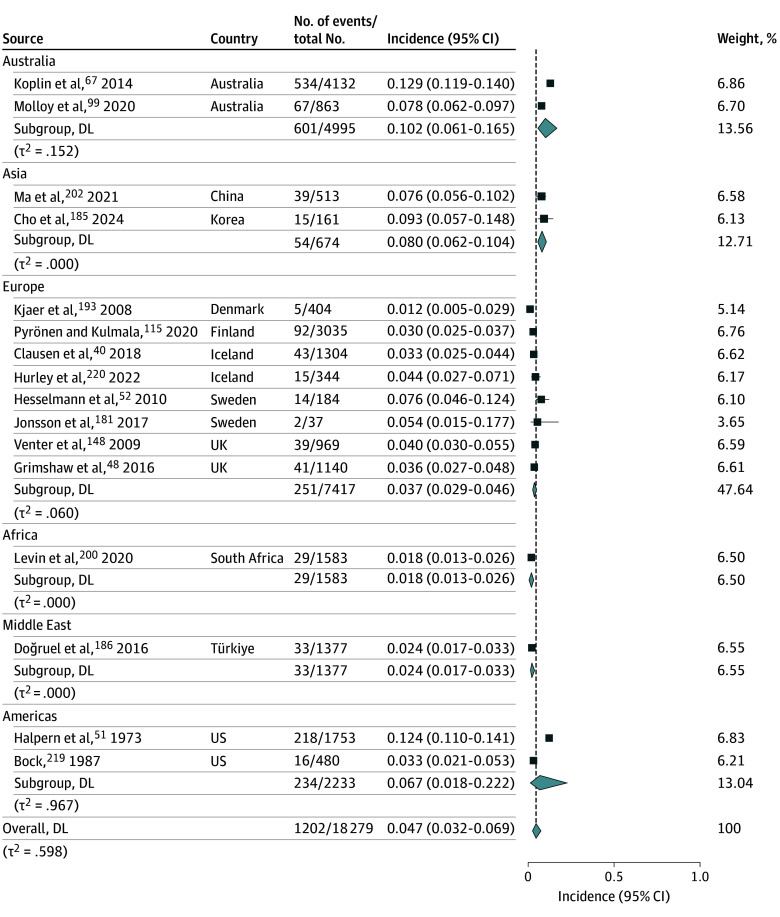
Systematic Review and Meta-Analysis of Incidence of Food Allergy Diagnosed by Food Challenge Forest plot showing the incidence of food allergy confirmed by food challenge, stratified by region. Each square represents the incidence estimate for an individual study, with the size proportional to study weight; horizontal lines indicate 95% confidence intervals. Diamonds indicate pooled random-effects estimates for each region and the overall estimate. DL indicates DerSimonian-Laird.

A total of 16 studies^40,48,51,52,67,99,115,148,181,185,186,193,200,202,219,220^ (n = 18 279 participants) assessed the incidence of IgE-mediated food allergy diagnosed by food challenge. Moderate-certainty evidence suggests that the average baseline incidence of food allergy is likely 4.7% (95% CI, 3.2%-6.9%). Regional variations include possible higher incidence in Australia (10.2%; 95% CI, 6.1%-16.5%) and the US (6.7%; 95% CI, 1.8%-22.2%) and lower incidence in the Middle East (2.4%; 95% CI, 1.7%-3.3%) and in Africa (1.8%; 95% CI, 1.3%-2.6%) (Figure 2; eTable 3 in Supplement 1).

### Risk Factors (Predictors) of IgE-Mediated Food Allergy

A total of 176 studies^31,32,33,34,35,36,37,38,39,41,42,43,44,45,46,47,49,50,53,54,55,56,57,58,59,60,61,62,63,64,65,66,68,69,70,71,72,73,74,75,76,77,78,79,80,81,82,83,84,85,86,87,88,89,90,91,92,93,94,95,96,97,98,100,101,102,103,104,105,106,107,108,109,110,111,112,113,114,116,117,118,119,120,121,122,123,124,125,126,127,128,129,130,131,132,133,134,135,136,137,138,139,140,141,142,143,144,145,146,147,149,150,151,152,153,154,155,156,157,158,159,160,161,162,163,164,165,166,167,168,169,170,171,172,173,174,175,176,177,178,179,180,181,182,183,184,186,187,188,189,190,191,192,194,195,196,197,198,199,201,203,204,205,206,207,208,209,210,211,212,213,214,215,216,217,218^ reported 342 risk factors associated with the development of food allergy. Of these, 38 were supported by high-certainty evidence, 69 by moderate-certainty, 120 by low-certainty, and 115 by very low–certainty evidence.

**Figure 3.**
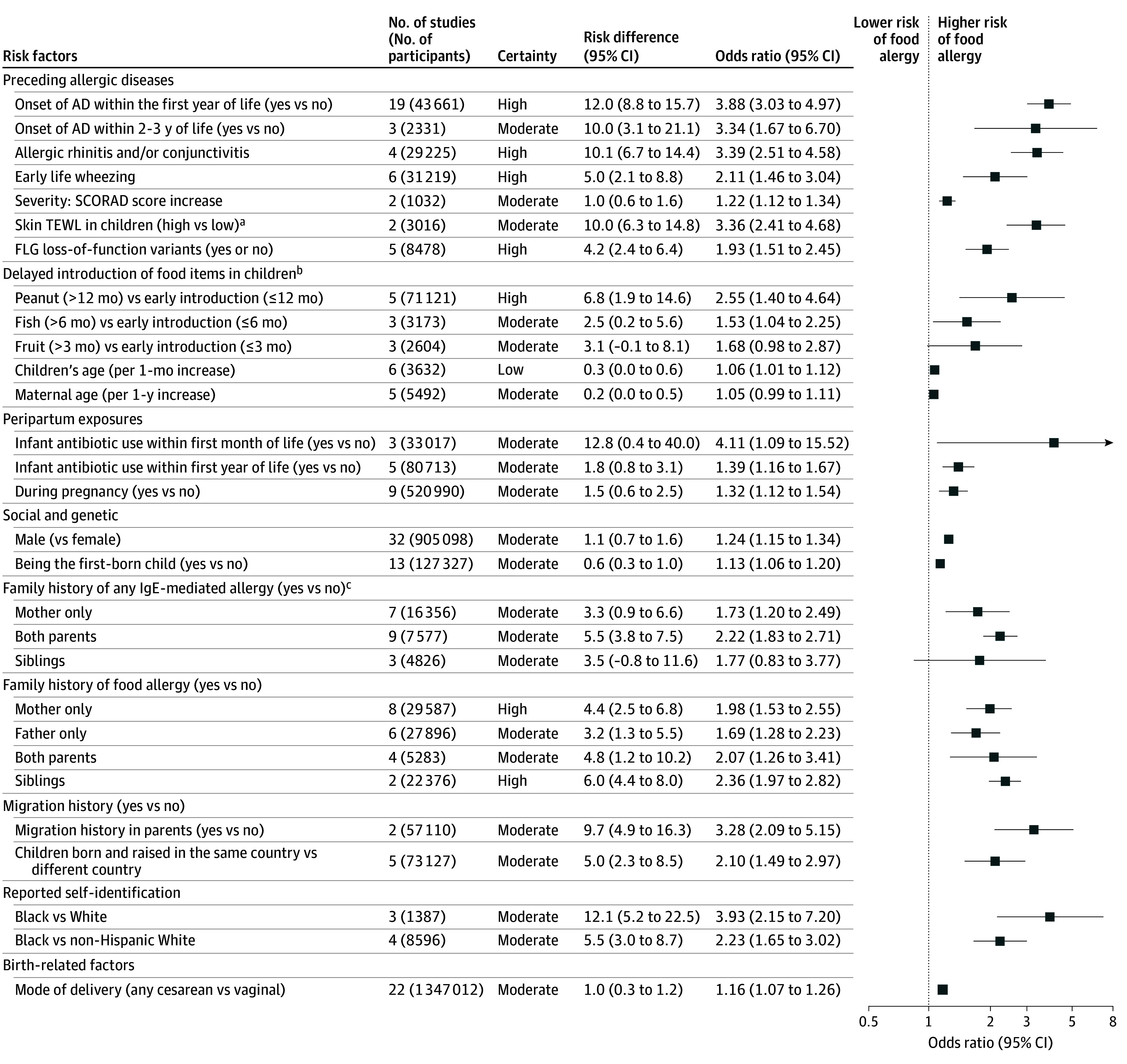
Systematic Review and Meta-Analysis of High- and Moderate-Certainty Risk Factors Associated With Developing Food Allergy Forest plot showing pooled associations between risk factors and development of food allergy in children, summarized across 190 studies (2.8 million participants). Risk factors are grouped into categories, including prior allergic diseases, delayed introduction of allergenic foods, peripartum exposures (during pregnancy primarily second or third trimester), social and genetic factors, family history of allergy or food allergy, nativity and migration history, reported self-identification, and birth-related factors. Each point estimate is shown as an odds ratio (OR) with 95% confidence intervals; squares represent pooled estimates. Certainty of evidence was graded using Grading of Recommendations Assessment, Development, and Evaluation (GRADE) methodology. AD indicates atopic dermatitis; FLG; filaggrin gene; SCORAD, Scoring Atopic Dermatitis (score ranges 0-103, with higher scores indicating greater severity); TEWL, transepidermal water loss. ^a^High skin TEWL: ≥9 g/m^2^/h; low skin TEWL: <9 g/m^2^/h. ^b^Delayed introduction of other foods showed a similar magnitude and direction. ^c^Family history of any immunoglobulin E (IgE)–mediated allergy (eg, asthma, AD, food allergy, allergic rhinitis, and/or conjunctivitis).

Figure 3 and eTable 4 in Supplement 1 summarize the high- and moderate-certainty evidence for risk factors associated with the development of food allergy in early life, while eTable 5 in Supplement 1 summarizes factors with low or very low certainty.

The high- and moderate-certainty factors include preceding allergic diseases (also referred to as the atopic march^221^ or diathesis), a history of atopic dermatitis (AD) (eg, eczema) within the first year of life (eFigure 1 in Supplement 1; OR, 3.88; RD, 12.0%; 95% CI, 8.8%-15.7%), allergic rhinitis and/or conjunctivitis (eFigure 2 in Supplement 1; OR, 3.39; RD, 10.1%; 95% CI, 6.7%-14.4%), or wheezing (eFigure 3 in Supplement 1; OR, 2.11; RD, 5.0%; 95% CI, 2.1%-8.8%). The association with AD persisted within the first 3 years of life (OR, 3.34; RD, 10.0%; 95% CI, 3.1%-21.1%), among those with mild disease (OR, 3.91; RD, 12.1%; 95% CI, 3.2%-27.1%), and increased with severity of AD (eFigure 4 in Supplement 1; OR, 1.22 per 5-10 points as measured by Scoring Atopic Dermatitis [SCORAD; 0-103, with higher scores indicating greater severity, averages 20-30]; RD, 1.0%; 95% CI, 0.6%-1.6%). Consistent with this, increased skin transepidermal water loss (eFigure 5 in Supplement 1; OR, 3.36; RD, 10.0%; 95% CI, 6.3%-14.8%), the presence of filaggrin gene loss-of-function sequence variation (eFigure 6 in Supplement 1; OR, 1.93; RD, 4.2%; 95% CI, 2.4%-6.4%), and child’s age (per 1-month increase) (eFigure 36 in Supplement 1; OR, 1.06; RD, 0.3%; 95% CI, 0.0%-0.6%) were also risk factors.

In terms of oral allergen exposure, it was found that delayed introduction of peanut after 12 months (eFigure 7 in Supplement 1; OR, 2.55; RD, 6.8%; 95% CI, 1.9%-14.6%) was associated with developing food allergy. Similar associations were found with delayed fish (eFigure 8 in Supplement 1; OR, 1.53; RD, 2.5%; 95% CI, 0.2%-5.6%), egg (eFigure 9 in Supplement 1), and fruit introduction (eFigure 10 in Supplement 1).

In terms of peripartum exposures, infant systemic antibiotic use within the first month of life was a stronger risk factor (eFigure 11 in Supplement 1; OR, 4.11; RD, 12.8%; 95% CI, 0.4%-40%) than exposure within the first year of life (eFigure 12 in Supplement 1; OR, 1.39; RD, 1.8%; 95% CI, 0.8%-3.1%) or during pregnancy (eFigure 13 in Supplement 1; OR, 1.32; RD, 1.5%; 95% CI, 0.6%-2.5%).

Social and genetic risk factors include male sex (eFigure 14 in Supplement 1; OR, 1.24; RD, 1.1%; 95% CI, 0.7%-1.6%) and being the firstborn child (eFigure 15 in Supplement 1; OR, 1.13; RD, 0.6%; 95% CI, 0.3%-1.0%); similarly increased risk was found with a family history of any allergy (eFigure 16 in Supplement 1), asthma (eFigure 17 in Supplement 1), AD (eFigure 18 in Supplement 1), food allergy (eFigure 19 in Supplement 1), or allergic rhinitis (eFigure 20 in Supplement 1) in either parent or sibling, and greatest risk when both parents had allergies (eg, a family history of food allergy in either the mother [OR, 1.98; RD, 4.4%; 95% CI, 2.5%-6.8%], father [OR, 1.69; RD, 3.2%; 95% CI, 1.3%-5.5%], both parents [OR, 2.07; RD, 4.8%; 95% CI, 1.3%-5.5%], or siblings [OR, 2.36; RD, 6.0%; 95% CI, 4.4%-8.0%]). We also found parental migration prior to birth (eFigure 21 in Supplement 1; OR, 3.28; RD, 9.7%; 95% CI, 4.9%-16.3%) and children born and raised in the same country (eFigure 21 in Supplement 1; OR, 2.10; RD, 5.0%; 95% CI, 2.3%-8.5%) were associated with an increased risk for developing food allergy. Self-identification, as reported in the individual studies, as Black vs White (eFigure 22 in Supplement 1; OR, 3.93; RD, 12.1%; 95% CI, 5.2%-22.5%) or vs non-Hispanic White (eFigure 22 in Supplement 1; OR, 2.23; RD, 5.5%; 95% CI, 3.0%-8.7%) was also associated with increased risk.

In terms of birth-related factors, cesarean delivery (eFigure 23 in Supplement 1; OR, 1.16; RD, 1.0%; 95% CI, 0.3%-1.2%) was likely associated with an important increased risk, and increasing maternal age may be (eFigure 26 in Supplement 1; OR, 1.05 per year). Low birth weight (defined as weight <2500 g), postterm birth (defined as ≥42 weeks of gestation), partial breastfeeding, maternal intake of fish or cheese during pregnancy, maternal stress during pregnancy, and high household income showed no important risk difference (eFigures 24-29 in Supplement 1).

Factors with low-certainty evidence for possibly being an important risk factor included facial AD lesions with and without exudates, AD affecting beyond the flexural folds, exposure to pollutants, social history (eg, higher parental education, having a general [primary care] physician, family history of farming), birth-related factors (eg, preeclampsia, increased duration of ruptured membranes), maternal intake of acid suppressant medication during pregnancy, maternal use of antibiotics during the postnatal period, maternal intake of allergenic foods and nutrients during pregnancy and the postnatal period, maternal depression during pregnancy or the postnatal period, delayed introduction of other solid foods (eg, meat, cabbage, bread, carrots, soy), presence of a dog or cat at home, pacifier sanitization by antiseptic, household peanut protein (environmental exposure to allergen), metabolic biomarkers (eg, low vitamin D in children, high vaccenic acid), genetic biomarkers (C11orf30 [EMSY] variant LRRC32, SPINK5 variant rs9325071, MALT1 variant rs57265082), and the elevated ratio of infant gut Enterobacteriaceae to Bacterioidaceae relative abundance detected from stool (eFigures 30-34 in Supplement 1). Factors that may have no important risk difference are presented in eFigures 35 through 43 in Supplement 1.

### Subgroup Analyses

No credible subgroup differences were found across any of the risk factors based on risk of bias, food allergy definitions, study year (pre- vs post-2015), or the number of allergenic foods (single vs multiple) (eTables 6 and 7 in Supplement 1). Subgroup analysis of incidence suggested possible but uncertain lower incidence of food allergy after early introduction guidelines were issued compared to before (ratio of incidence, 0.92; 95% CI, 0.77-1.10; low certainty) and no other credible effect modifiers.

## Discussion

This systematic review and meta-analysis of 190 studies involving 2.8 million participants found an average 5% incidence of food allergy by age 6 years and evaluated the strength and credibility of 342 associated risk (predictive) factors. The strongest and most credible risk factors can be classified as major (OR ≥2 and RD ≥5%) or minor (OR ≤1.5, RD 1%-2%). Major risk factors include early life antibiotic use, self-identification as Black, early onset of allergic conditions (atopic march or diathesis,^221^ AD, rhinitis, asthma or wheezing), elevated skin transepidermal water loss, parental migration before birth, delayed introduction of solid foods, and family history of food allergy or related allergies. Minor risk factors include filaggrin gene sequence variations, male sex, cesarean delivery, and firstborn status. Factors like low birth weight, postterm birth, maternal age, breastfeeding promotion, and maternal stress during pregnancy showed no important risk difference.

Strengths of this review compared to previous reviews^222,223,224^ include being the first to systematically synthesize a large number of risk factors and contextualize them among each other, rather than focusing on isolated exposures (eg, breastfeeding alone). Further, this systematic synthesis of the evidence clarifies associations that were uncertain or contentious in individual reports, such as cesarean delivery, family history, male sex, child age, and maternal age. Methodological strengths compared to previous reviews include requiring adjusted analyses and using structured tools, such as the GRADE approach, to systematically appraise evidence certainty for each outcome and factor.

By systematically quantifying and classifying both major and minor predictors, this study reconciles inconsistencies in definitions of at-risk populations for developing food allergy in clinical guidelines and trials,^6^ advances the concept that the development of food allergy is multifactorial rather than solely driven by eczema or timing of allergen introduction, and provides systematically appraised evidence directly responsive to the patient- and US National Academies of Medicine–identified priority gaps in food allergy prevention evidence,^2,225^ which are crucial not only for allergists and pediatricians, but also for internists, obstetricians and gynecologists, family medicine, public health, infectious disease, dermatology, nutritionists and dieticians, immigrant health, equity-deserving groups, policymakers, and researchers.

The implications of our findings to advancing understanding of food allergy pathophysiology and optimal management include structured interpretation of the risk factors for food allergy and contextualizing competing paradigms^226,227^ attempting to explain the incidence and factors promoting the development of food allergy with one another. For example, Strachan’s^228^ hygiene hypothesis suggested a microbial basis for food allergy (eg, antibiotic-induced promotion of food allergic immunity^229,230^); others have advanced a genetic basis for food allergy, and, most recently, a dual-allergen exposure hypothesis suggested skin exposure on eczematous skin promotes allergy, and oral exposure promotes tolerance. Rather than representing competing paradigms, our findings support a unifying multifactorial model in which food allergy arises from intersecting microbial, genetic, environmental, allergen exposure, and social influences, driven by a major risk factor or a combination of multiple major or minor risk factors. For example, migration may impact allergen exposure timing, worsen eczema, and alter the microbiome. Together, this concept and the associated quantitative risk estimates should inform updated food allergy guidelines, clinical practice, and research.

The clinical implications of our findings include clarifying which children are at highest risk and therefore enabling targeted prevention strategies. From a practice and policy standpoint, these findings support global consensus on defining high-risk infants. For researchers, the findings highlight key variables to prioritize in future randomized clinical trials and mechanistic studies, helping refine study design and improve intervention development. For instance, addressing risk factors enables robust and efficient trials that are critical to understanding the origins and mechanisms of food allergy and developing new prevention strategies.

### Limitations

The limitations of this systematic review largely stem from the limitations of the available literature. First, many risk factors were supported by low- or very low–certainty evidence (often single small studies). Informed by this large meta-analysis, investigators can target future rigorous observational and interventional studies to address the identified evidence gaps. Second, not all studies adjusted for the same variables, and they rarely adjusted for all identified risk factors. Thus, inferences regarding the effect or interaction between combinations of the identified factors may not be additive. For example, estimates of increased food allergy risk with filaggrin sequence variation, skin transepidermal water loss, and AD were consistent among studies that did or did not adjust for all 3 factors, implying that each factor might be independently associated with developing food allergy, but being limited to between-study comparisons does not fully rule out that these factors might represent a single major skin risk factor. Likewise, the inconsistent findings among studies examining exclusive breastfeeding for more than 5 to 6 months and low certainty for increased food allergy might be confounded and partly explained by delayed introduction of foods. Our findings therefore strengthen calls to harmonize birth cohorts^231^ and suggest that future studies should adjust for common core factors and/or make their datasets publicly available. Third, while we identify several important factors associated with food allergy development, causality remains uncertain and requires further investigation through robust randomized trials. Fourth, most included studies were conducted in higher-income countries, which may limit the generalizability of findings to low- and middle-income settings. Fifth, while food allergy incidence estimates pooled only studies using food challenge, risk factor analyses included studies that did and did not confirm food allergy by challenge, albeit their estimates were consistent with each other. Noting the lack of modern US birth cohorts using study-protocolized food challenge(s), the ongoing SunBEAm birth cohort^232^ may help address this gap.

## Conclusions

This systematic review and meta-analysis of 2.8 million participants in 190 studies identified the strongest and most credible risk factors for developing food allergy in children. The findings support a unifying paradigm in which food allergy arises from the convergence of genetic, microbial, environmental, social, and comorbid mechanisms, in addition to requisite allergen exposure, often driven by 1 or more major risk factors in combination with additional minor contributors, to inform optimal clinical practice, policy, and research.

## Notes

Supplement 1. eMethods 1. PRISMA Checklists eMethods 2. Additional Methods Details Including Data Extraction, Synthesis, Analysis, Certainty of Evidence Assessment and Heterogeneity Assessment eMethods 3. Search Strategies **eTable 1.** Study Characteristics of the Included Studies Addressing Food Allergy Incidence and/or Predictive Factors **eTable 2.** Risk of Bias Assessment of Included Studies for the Outcome of Developing Food Allergy **eTable 3.** GRADE Evidence Profile: Incidence of Food Allergy Diagnosed by Food Challenge **eTable 4.** GRADE Evidence Profile: Risk Factors of Food Allergy in Children **eTable 5.** Predictive Factors for the Development of Food Allergy in Early Life Reported in a Single Study **eTable 6.** Subgroup Analyses **eTable 7.** Credibility of Association of Significant Subgroup Analyses by Using Modified ICEMAN **eFigure** 1. Meta-Analysis of the Association Between Timing of Onset of Atopic Dermatitis in Children and Development of Food Allergy in Children **eFigure 2.** Meta-Analysis of the Association Between Previous History of Allergic Rhinitis and/or Conjunctivitis in Children and Development of Food Allergy in Children **eFigure 3.** Meta-Analysis of the Association Between History of Early Life Wheezing in Children and Development of Food Allergy in Children **eFigure 4.** Meta-Analysis of the Association Between the Severity of Atopic Dermatitis in Children and Development of Food Allergy in Children **eFigure 5.** Meta-Analysis of the Association Between High Skin Transepidermal Water Loss (TEWL) in Children and Development of Food Allergy in Children **eFigure 6.** Meta-Analysis of the Association Between Filaggrin Gene (FLG) Loss-of-Function Mutations in Children and Development of Food Allergy in Children **eFigure 7.** Meta-Analysis of the Association Between Delayed Introduction of Peanut and Development of Food Allergy to the Displayed Food in Children **eFigure 8.** Meta-Analysis of the Association Between Delayed Introduction of Fish and Development of Food Allergy to the Displayed Food in Children **eFigure 9.** Meta-Analysis of the Association Between Delayed Introduction of Egg and Development of Food Allergy to the Displayed Food in Children **eFigure 10.** Meta-Analysis of the Association Between Delayed Introduction of Fruit and Development of Food Allergy to the Displayed Food in Children **eFigure 11.** Meta-Analysis of the Association Between Antibiotic Use in Children Within 1st Month of Life and Development of Food Allergy in Children **eFigure 12.** Meta-Analysis of the Association Between Antibiotic Use in Children Within 1st Year of Life and Development of Food Allergy in Children **eFigure 13.** Meta-Analysis of the Association Between Maternal Use of Antibiotics During Pregnancy and Development of Food Allergy in Children **eFigure 14.** Meta-Analysis of the Association Between Sex and Development of Food Allergy in Children **eFigure 15.** Meta-Analysis of the Association Between Being the Firstborn Child and Development of Food Allergy in Children **eFigure 16.** Meta-Analysis of the Association Between Allergic Disorders (Asthma, Atopic Dermatitis, Food Allergy, Allergic Rhinitis, and/or Conjunctivitis) and Development of Food Allergy in Children **eFigure 17.** Meta-Analysis of the Association Between Family History of Asthma and Development of Food Allergy in Children **eFigure 18.** Meta-Analysis of the Association Between Family History of Atopic Dermatitis (Eczema) and Development of Food Allergy in Children **eFigure 19.** Meta-Analysis of the Association Between Family History of Food Allergy and Development of Food Allergy in Children **eFigure 20.** Meta-Analysis of the Association Between Family History of Allergic Rhinitis and/or Conjunctivitis and Development of Food Allergy in Children **eFigure 21.** Meta-Analysis of the Association Between Migration History and Development of Food Allergy in Children **eFigure 22.** Meta-Analysis of the Association Between Reported Self-Identification and Development of Food Allergy in Children **eFigure 23.** Meta-Analysis of the Association Between Any Caesarean Delivery and Development of Food Allergy in Children **eFigure 24.** Meta-Analysis of the Association Between High Birth Weight, Low Birth Weight, Mother BMI Obese, Mother BMI Underweight and Development of Food Allergy in Children **eFigure 25.** Meta-Analysis of the Association Between Postterm Birth, Preterm Birth, Season of Birth (Autumn), Season of Birth (Spring), Season of Birth (Winter) and Development of Food Allergy in Children **eFigure 26.** Meta-Analysis of the Association Between Maternal Age and Development of Food Allergy in Children **eFigure 27.** Meta-Analysis of the Association Between Pattern and Duration of Breastfeeding and Development of Food Allergy in Children **eFigure 28.** Meta-Analysis of the Association Between Maternal Diet During Pregnancy and Development of Food Allergy in Children **eFigure 29.** Meta-Analysis of the Association Between Household Income and Development of Food Allergy in Children **eFigure 30.** Meta-Analysis of the Association Between the Site of Atopic Dermatitis in Children and Development of Food Allergy in Children **eFigure 31.** Meta-Analysis of the Association Between Childcare/Day-Care Attendance, Parental Education and Development of Food Allergy in Children **eFigure 32.** Meta-Analysis of the Association Between Delayed Introduction of Food Items and Development of Food Allergy in Children **eFigure 33.** Meta-Analysis of the Association Between Presence of Pets and Development of Food Allergy in Children **eFigure 34.** Meta-Analysis of the Association Between Low Vitamin D in Children and Development of Food Allergy in Children **eFigure 35.** Meta-Analysis of the Association Between High Maternal Folate and Development of Food Allergy in Children **eFigure 36.** Meta-Analysis of the Association Between Children’s Age and Development of Food Allergy in Children **eFigure 37.** Meta-Analysis of the Association Between Maternal Smoking During Pregnancy and Development of Food Allergy in Children **eFigure 38.** Meta-Analysis of the Association Between History of Smoking in Both Parents and Development of Food Allergy in Children **eFigure 39.** Meta-Analysis of the Association Between Diphtheria-Tetanus-Pertussis (DTP) Vaccination and Development of Food Allergy in Children **eFigure 40.** Meta-Analysis of the Association Between Pattern and Duration of Breastfeeding and Development of Food Allergy in Children **eFigure 41.** Meta-Analysis of the Association Between Pattern and Duration of Breastfeeding and Development of Food Allergy in Children **eFigure 42.** Meta-Analysis of the Association Between Pattern and Duration of Breastfeeding and Development of Food Allergy in Children **eFigure 43.** Meta-Analysis of the Association Between History of Breastfeeding and Development of Food Allergy in Children eReferences.

Supplement 2. Data Sharing Statement
