# Supplementary material for: Risk Factors for the Development of Food Allergy in Infants and Children: A Systematic Review and Meta-Analysis
Source: JAMA Pediatr. 2026 Feb 9;180(5):486–99. doi: 10.1001/jamapediatrics.2025.6105 (PMC12887841; doi:10.1001/jamapediatrics.2025.6105)
Supplement: Supplement 1. — eMethods 1. PRISMA Checklists eMethods 2. Additional Methods Details Including Data Extraction, Synthesis, Analysis, Certainty of Evidence Assessment and Heterogeneity Assessment eMethods 3. Search Strategies eTable 1. Study Characteristics of the Included Studies Addressing Food Allergy Incidence and/or Predictive Factors eTable 2. Risk of Bias Assessment of Included Studies for the Outcome of Developing Food Allergy eTable 3. GRADE Evidence Profile: Incidence of Food Allergy Diagnosed by Food Challenge eTable 4. GRADE Evidence Profile: Risk Factors of Food Allergy in Children eTable 5. Predictive Factors for the Development of Food Allergy in Early Life Reported in a Single Study eTable 6. Subgroup Analyses eTable 7. Credibility of Association of Significant Subgroup Analyses by Using Modified ICEMAN eFigure 1. Meta-Analysis of the Association Between Timing of Onset of Atopic Dermatitis in Children and Development of Food Allergy in Children eFigure 2. Meta-Analysis of the Association Between Previous History of Allergic Rhinitis and/or Conjunctivitis in Children and Development of Food Allergy in Children eFigure 3. Meta-Analysis of the Association Between History of Early Life Wheezing in Children and Development of Food Allergy in Children eFigure 4. Meta-Analysis of the Association Between the Severity of Atopic Dermatitis in Children and Development of Food Allergy in Children eFigure 5. Meta-Analysis of the Association Between High Skin Transepidermal Water Loss (TEWL) in Children and Development of Food Allergy in Children eFigure 6. Meta-Analysis of the Association Between Filaggrin Gene (FLG) Loss-of-Function Mutations in Children and Development of Food Allergy in Children eFigure 7. Meta-Analysis of the Association Between Delayed Introduction of Peanut and Development of Food Allergy to the Displayed Food in Children eFigure 8. Meta-Analysis of the Association Between Delayed Introduction of Fish and Development of Food Allergy to the Displayed Food i [file jamapediatr-e256105-s001.pdf]

Supplemental Online Content

Islam N, Chu AWL, Sheriff F, et al. Risk factors for the development of food allergy in infants and children: a systematic review and meta-analysis. *JAMA Pediatr*. Published online February 9, 2026. doi:10.1001/jamapediatrics.2025.6105

eMethods 1. PRISMA Checklists

eMethods 2. Additional Methods Details Including Data Extraction, Synthesis, Analysis, Certainty of Evidence Assessment and Heterogeneity Assessment

eMethods 3. Search Strategies

eTable 1. Study Characteristics of the Included Studies Addressing Food Allergy Incidence and/or Predictive Factors

eTable 2. Risk of Bias Assessment of Included Studies for the Outcome of Developing Food Allergy.

eTable 3. GRADE Evidence Profile: Incidence of Food Allergy Diagnosed by Food Challenge

eTable 4. GRADE Evidence Profile: Risk Factors of Food Allergy in Children

eTable 5. Predictive Factors for the Development of Food Allergy in Early Life Reported in a Single Study

eTable 6. Subgroup Analyses

eTable 7. Credibility of Association of Significant Subgroup Analyses by Using Modified ICEMAN

eFigure 1. Meta-Analysis of the Association Between Timing of Onset of Atopic Dermatitis in Children and Development of Food Allergy in Children

eFigure 2. Meta-Analysis of the Association Between Previous History of Allergic Rhinitis and/or Conjunctivitis in Children and Development of Food Allergy in Children

eFigure 3. Meta-Analysis of the Association Between History of Early Life Wheezing in Children and Development of Food Allergy in Children

eFigure 4. Meta-Analysis of the Association Between the Severity of Atopic Dermatitis in Children and Development of Food Allergy in Children

eFigure 5. Meta-Analysis of the Association Between High Skin Transepidermal Water Loss (TEWL) in Children and Development of Food Allergy in Children

eFigure 6. Meta-Analysis of the Association Between Filaggrin Gene (FLG) Loss-of-Function Sequence Variations in Children and Development of Food Allergy in Children

eFigure 7. Meta-Analysis of the Association Between Delayed Introduction of Peanut and Development of Food Allergy to the Displayed Food in Children

eFigure 8. Meta-Analysis of the Association Between Delayed Introduction of Fish and Development of Food Allergy to the Displayed Food in Children

© 2026 Islam N et al. *JAMA Pediatrics*.

eFigure 9. Meta-Analysis of the Association Between Delayed Introduction of Egg and Development of Food Allergy to the Displayed Food in Children

eFigure 10. Meta-Analysis of the Association Between Delayed Introduction of Fruit and Development of Food Allergy to the Displayed Food in Children

eFigure 11. Meta-Analysis of the Association Between Antibiotic Use in Children Within 1st Month of Life and Development of Food Allergy in Children

eFigure 12. Meta-Analysis of the Association Between Antibiotic Use in Children Within 1st Year of Life and Development of Food Allergy in Children

eFigure 13. Meta-Analysis of the Association Between Maternal Use of Antibiotics During Pregnancy and Development of Food Allergy in Children

eFigure 14. Meta-Analysis of the Association Between Sex and Development of Food Allergy in Children

eFigure 15. Meta-Analysis of the Association Between Being the Firstborn Child and Development of Food Allergy in Children

eFigure 16. Meta-Analysis of the Association Between Allergic Disorders (Asthma, Atopic Dermatitis, Food Allergy, Allergic Rhinitis, and/or Conjunctivitis) and Development of Food Allergy in Children

eFigure 17. Meta-Analysis of the Association Between Family History of Asthma and Development of Food Allergy in Children

eFigure 18. Meta-Analysis of the Association Between Family History of Atopic Dermatitis (Eczema) and Development of Food Allergy in Children

eFigure 19. Meta-Analysis of the Association Between Family History of Food Allergy and Development of Food Allergy in Children

eFigure 20. Meta-Analysis of the Association Between Family History of Allergic Rhinitis and/or Conjunctivitis and Development of Food Allergy in Children

eFigure 21. Meta-Analysis of the Association Between Migration History and Development of Food Allergy in Children

eFigure 22. Meta-Analysis of the Association Between Reported Self-Identification and Development of Food Allergy in Children

eFigure 23. Meta-Analysis of the Association Between Any Caesarean Delivery and Development of Food Allergy in Children

eFigure 24. Meta-Analysis of the Association Between High Birth Weight, Low Birth Weight, Mother BMI Obese, Mother BMI Underweight and Development of Food Allergy in Children

eFigure 25. Meta-Analysis of the Association Between Postterm Birth, Preterm Birth, Season of Birth (Autumn), Season of Birth (Spring), Season of Birth (Winter) and Development of Food Allergy in Children

eFigure 26. Meta-Analysis of the Association Between Maternal Age and Development of Food Allergy in Children

eFigure 27. Meta-Analysis of the Association Between Pattern and Duration of Breastfeeding and Development of Food Allergy in Children

© 2026 Islam N et al. *JAMA Pediatrics*.

eFigure 28. Meta-Analysis of the Association Between Maternal Diet During Pregnancy and Development of Food Allergy in Children

eFigure 29. Meta-Analysis of the Association Between Household Income and Development of Food Allergy in Children

eFigure 30. Meta-Analysis of the Association Between the Site of Atopic Dermatitis in Children and Development of Food Allergy in Children

eFigure 31. Meta-Analysis of the Association Between Childcare/Day-Care Attendance, Parental Education and Development of Food Allergy in Children

eFigure 32. Meta-Analysis of the Association Between Delayed Introduction of Food Items and Development of Food Allergy in Children

eFigure 33. Meta-Analysis of the Association Between Presence of Pets and Development of Food Allergy in Children

eFigure 34. Meta-Analysis of the Association Between Low Vitamin D in Children and Development of Food Allergy in Children

eFigure 35. Meta-Analysis of the Association Between High Maternal Folate and Development of Food Allergy in Children

eFigure 36. Meta-Analysis of the Association Between Children's Age and Development of Food Allergy in Children

eFigure 37. Meta-Analysis of the Association Between Maternal Smoking During Pregnancy and Development of Food Allergy in Children

eFigure 38. Meta-Analysis of the Association Between History of Smoking in Both Parents and Development of Food Allergy in Children

eFigure 39. Meta-Analysis of the Association Between Diphtheria-Tetanus-Pertussis (DTP) Vaccination and Development of Food Allergy in Children

eFigure 40. Meta-Analysis of the Association Between Pattern and Duration of Breastfeeding and Development of Food Allergy in Children

eFigure 41. Meta-Analysis of the Association Between Pattern and Duration of Breastfeeding and Development of Food Allergy in Children

eFigure 42. Meta-Analysis of the Association Between Pattern and Duration of Breastfeeding and Development of Food Allergy in Children

eFigure 43. Meta-Analysis of the Association Between History of Breastfeeding and Development of Food Allergy in Children

eReferences.

This supplemental material has been provided by the authors to give readers additional information about their work.

© 2026 Islam N et al. *JAMA Pediatrics*.

eMethods 1. PRISMA checklist.

Section and Topic	Item #	Checklist item	Location where item is reported
**TITLE**			
Title	1	Identify the report as a systematic review.	1
**ABSTRACT**			
Abstract	2	Provide a structured summary including, as applicable: background; objectives; data sources; study eligibility criteria, participants, and interventions; study appraisal and synthesis methods; results; limitations; conclusions and implications of key findings; systematic review registration number	5-6
**INTRODUCTION**			
Rationale	3	Describe the rationale for the review in the context of existing knowledge.	7
Objectives	4	Provide an explicit statement of the objective(s) or question(s) the review addresses.	7
**METHODS**			
Eligibility criteria	5	Specify the inclusion and exclusion criteria for the review and how studies were grouped for the syntheses.	8
Information sources	6	Specify all databases, registers, websites, organisations, reference lists and other sources searched or consulted to identify studies. Specify the date when each source was last searched or consulted.	8
Search strategy	7	Present the full search strategies for all databases, registers and websites, including any filters and limits used.	8 & eMethods 3
Selection process	8	Specify the methods used to decide whether a study met the inclusion criteria of the review, including how many reviewers screened each record and each report retrieved, whether they worked independently, and if applicable, details of automation tools used in the process.	8 & eMethods 2
Data collection process	9	Specify the methods used to collect data from reports, including how many reviewers collected data from each report, whether they worked independently, any processes for obtaining or confirming data from study investigators, and if applicable, details of automation tools used in the process.	8-9 & eMethods 2
Data items	10a	List and define all outcomes for which data were sought. Specify whether all results that were compatible with each outcome domain in each study were sought (e.g. for all measures, time points, analyses), and if not, the methods used to decide which results to collect.	9
	10b	List and define all other variables for which data were sought (e.g. participant and intervention characteristics, funding sources). Describe any assumptions made about any missing or unclear information.	8-9
Study risk of bias assessment	11	Specify the methods used to assess risk of bias in the included studies, including details of the tool(s) used, how many reviewers assessed each study and whether they worked independently, and if applicable, details of automation tools used in the process.	9
Effect measures	12	Specify for each outcome the effect measure(s) (e.g. risk ratio, mean difference) used in the synthesis or presentation of results.	10 & eMethods 2
Synthesis methods	13a	Describe the processes used to decide which studies were eligible for each synthesis (e.g. tabulating the study intervention characteristics and comparing against the planned groups for each synthesis (item #5)).	NA
	13b	Describe any methods required to prepare the data for presentation or synthesis, such as handling of missing summary statistics, or data conversions.	NA
	13c	Describe any methods used to tabulate or visually display results of individual studies and syntheses.	10 & eMethods 2
	13d	Describe any methods used to synthesize results and provide a rationale for the choice(s). If meta-analysis was performed, describe the model(s), method(s) to identify the presence and extent of statistical heterogeneity, and software package(s) used.	10 & eMethods 2
	13e	Describe any methods used to explore possible causes of heterogeneity among study results (e.g.	10 &

© 2026 Islam N et al. *JAMA Pediatrics*.

Section and Topic	Item #	Checklist item	Location where item is reported
		subgroup analysis, meta-regression).	eMethods 2
	13f	Describe any sensitivity analyses conducted to assess robustness of the synthesized results.	NA
Reporting bias assessment	14	Describe any methods used to assess risk of bias due to missing results in a synthesis (arising from reporting biases).	NA
Certainty assessment	15	Describe any methods used to assess certainty (or confidence) in the body of evidence for an outcome.	9 & eMethods 2
**RESULTS**			
Study selection	16a	Describe the results of the search and selection process, from the number of records identified in the search to the number of studies included in the review, ideally using a flow diagram.	11 & Figure 1
	16b	Cite studies that might appear to meet the inclusion criteria, but which were excluded, and explain why they were excluded.	NA
Study characteristics	17	Cite each included study and present its characteristics.	11 & eTable 1
Risk of bias in studies	18	Present assessments of risk of bias for each included study.	11 & eTable 2
Results of individual studies	19	For all outcomes, present, for each study: (a) summary statistics for each group (where appropriate) and (b) an effect estimate and its precision (e.g. confidence/credible interval), ideally using structured tables or plots.	NA
Results of syntheses	20a	For each synthesis, briefly summarise the characteristics and risk of bias among contributing studies.	11 & Table 1
	20b	Present results of all statistical syntheses conducted. If meta-analysis was done, present for each the summary estimate and its precision (e.g. confidence/credible interval) and measures of statistical heterogeneity. If comparing groups, describe the direction of the effect.	11-13, Figures 2-3, eTables 3-4 & eFigures 1-43
	20c	Present results of all investigations of possible causes of heterogeneity among study results.	14 & eTables 6-7
	20d	Present results of all sensitivity analyses conducted to assess the robustness of the synthesized results.	NA
Reporting biases	21	Present assessments of risk of bias due to missing results (arising from reporting biases) for each synthesis assessed.	NA
Certainty of evidence	22	Present assessments of certainty (or confidence) in the body of evidence for each outcome assessed.	11-14, Figures 2-3 & eTables 3-4
**DISCUSSION**			
Discussion	23a	Provide a general interpretation of the results in the context of other evidence.	15
	23b	Discuss any limitations of the evidence included in the review.	16
	23c	Discuss any limitations of the review processes used.	16
	23d	Discuss implications of the results for practice, policy, and future research.	15-16
**OTHER INFORMATION**			
Registration and protocol	24a	Provide registration information for the review, including register name and registration number, or state that the review was not registered.	8
	24b	Indicate where the review protocol can be accessed, or state that a protocol was not prepared.	8

© 2026 Islam N et al. *JAMA Pediatrics*.

Section and Topic	Item #	Checklist item	Location where item is reported
	24c	Describe and explain any amendments to information provided at registration or in the protocol.	NA
Support	25	Describe sources of financial or non-financial support for the review, and the role of the funders or sponsors in the review.	18
Competing interests	26	Declare any competing interests of review authors.	18
Availability of data, code and other materials	27	Report which of the following are publicly available and where they can be found: template data collection forms; data extracted from included studies; data used for all analyses; analytic code; any other materials used in the review.	19

© 2026 Islam N et al. *JAMA Pediatrics*.

eMethods 2. Additional methods details including data extraction, synthesis, analysis, certainty of evidence assessment and heterogeneity assessment.

Pilot extraction involved pairs of reviewers extracting data from the same 2 articles. Once 100% agreement was achieved for the 10 articles, reviewers extracted data from the remaining articles independently and in duplicate. Disagreements were resolved through consensus. If a study reported multiple regression models, we included the model that adjusted for the largest number of variables, prioritizing those that included the most critical prognostic factors, such as age and sex. We combined ratios from studies with different numbers of adjustments if the key prognostic factors were consistently accounted for across the models, ensuring they estimated similar effects.

We obtained individual patient-level data from Canadian Healthy Infant Longitudinal Development (CHILD)[1-3] because of its unique and relevant information on early-life risk factors for food allergy to examine the effect of prenatal and postnatal antibiotic exposure on the development of food allergy in children within the first three years of life. The cohort comprised women and their children meeting specified criteria, including age ≥18 (≥19 in Vancouver), residing near designated recruitment centers, capable of English communication, and willing to provide consent. We employed the Least Absolute Shrinkage and Selection Operator (LASSO)[4] and literature review to identify potential confounders. We identified the following confounders- maternal reported ethnicity, family income, gestational age (weeks), paternal reported ethnicity, atopic dermatitis within the first year of life, maternal history of asthma, maternal history of food allergy, maternal history of atopic dermatitis, paternal history of food allergy and sex of the children. After investigating the missing data pattern, we used a multivariable logistic regression with multiple imputations by chained equations (MICE)[5,6] adjusting for potential confounders to investigate the effect of prenatal and postnatal use of antibiotics on food allergy development.

In data synthesis and analysis, to calculate the absolute risk increase or decrease for each predictor, we estimated the baseline risk for food allergy as the calculated incidence, using data from the studies where participants underwent OFC, which is the gold standard for diagnosing food allergy and thus provides more precise and reliable data.

© 2026 Islam N et al. *JAMA Pediatrics*.

We rated the overall certainty (quality) of evidence for each prognostic factor using the GRADE approach.[7,8] The GRADE approach evaluates imprecision, inconsistency, risk of bias, indirectness, publication bias, and factors that enhance the certainty of the evidence (e.g. large effects, dose-response, and accounting for plausible residual confounding). The risk difference (RD) was categorized as important (increase or decrease) or no association based on the MID. We rated the certainty of evidence down for imprecision if the 95% confidence interval for the RD included 1%, indicating that the effect could be as small as 1% or less. We also rated down for imprecision of factors that is reported in a single study if the ratio of 95% confidence interval (upper limit/lower limit) is ≥2. We present the review's findings in an evidence profile table, summarizing the certainty of the evidence and the magnitude of the impact of the studied prognostic factors, following Cochrane guidance.[9] The summary of association tables synthesizes the evidence according to the magnitude of effect and certainty.

We assessed heterogeneity according to the GRADE approach.[8,10,11] Given that common statistical tests like I^2 are likely to lead to spurious conclusions in prognosis studies, we focused on visual inspection of the forest plots, particularly examining differences in point estimates and the overlap of confidence intervals.[7]

© 2026 Islam N et al. *JAMA Pediatrics*.

eMethods 3. Search strategies.

Database: Ovid MEDLINE(R) ALL	
1	exp Food Hypersensitivity/
2	((food or peanut or egg or milk or nut or wheat or fish or shellfish) adj3 (allergy or allergic or hypersensitiv*)).mp.
3	1 or 2
4	exp adolescent/ or exp child/ or exp infant/ or (infant disease* or childhood disease*).ti,ab,kf. or (adolescen* or babies or baby or boy? or boyfriend or boyhood or girlfriend or girlhood or child* or girl? or infan* or juvenil* or kid? or minors or minors* or neonat* or neo-nat* or newborn* or new-born* or paediatric* or peadiatric* or pediatric* or perinat* or preschool* or puber* or pubescen* or school* or teen* or toddler? or underage? or under-age? or youth*).ti,ab,kf. or (pediatric* or paediatric* or infan* or child* or adolescen* or young).jn,jw. or (pediatric* or paediatric* or infan* or child* or adolescen* or young).in.
5	3 and 4
6	prognosis/
7	exp Risk/
8	exp Probability/
9	exp Regression Analysis/
10	"analysis of variance"/ or multivariate analysis/
11	exp Epidemiologic Studies/
12	(prognosis or prognostic or predict* or risk*).mp.
13	((univariate or covariate or variance or covariance or multivariate or regression or adjusted or unadjusted or logistic or diagnostic) adj2 (analys* or model*)).mp.
14	(logistic adj2 regress*).mp.
15	((cohort or observational) adj3 (study or studies or analy*)).mp.
16	(longitudinal or retrospective or cross sectional or prospective).mp.
17	(Follow up adj (study or studies)).tw.
18	ep.fs.
19	6 or 7 or 8 or 9 or 10 or 11 or 12 or 13 or 14 or 15 or 16 or 17 or 18
20	5 and 19
21	skin prick test*.mp. or exp Skin Tests/
22	IgE.mp. or exp Immunoglobulin E/
23	oral food challenge*.mp.
24	21 or 22 or 23
25	exp Child/ or exp Cohort Studies/ or exp Child, Preschool/ or birth cohort*.mp. or exp Infant/
26	20 and 24 and 25
**Embase**	
1	exp Food Hypersensitivity/
2	((food or peanut or egg or milk or nut or wheat or fish or shellfish) adj3 (allergy or allergic or hypersensitiv*)).mp
3	1 or 2
4	exp adolescent/ or exp child/ or exp infant/ or (infant disease* or childhood disease*).ti,ab,kw. or (adolescen* or babies or baby or boy? or boyfriend or boyhood or girlfriend or girlhood or child* or girl? or infan* or juvenil* or kid? or minors or minors* or neonat* or neo-nat* or newborn* or new-born* or paediatric* or peadiatric* or pediatric* or perinat* or preschool* or puber* or pubescen* or school* or teen* or toddler? or underage? or under-age? or youth*).ti,ab,kw. or (pediatric* or paediatric* or infan* or child* or adolescen* or young).jn,jw. or (pediatric* or paediatric* or infan* or child* or adolescen* or young).in.
5	3 and 4
6	prognosis/
7	exp Risk/
8	exp Probability/

© 2026 Islam N et al. *JAMA Pediatrics*.

9	exp Regression Analysis/
10	"analysis of variance"/ or multivariate analysis/
11	exp Epidemiologic Studies/
12	(prognosis or prognostic or predict* or risk*).mp.
13	((univariate or covariate or variance or covariance or multivariate or regression or adjusted or unadjusted or logistic or diagnostic) adj2 (analys* or model*)).mp.
14	(logistic adj2 regress*).mp.
15	((cohort or observational) adj3 (study or studies or analy*)).mp.
16	(longitudinal or retrospective or cross sectional or prospective).mp
17	(Follow up adj (study or studies)).tw.
18	ep.fs.
19	6 or 7 or 8 or 9 or 10 or 11 or 12 or 13 or 14 or 15 or 16 or 17 or 18
20	5 and 19
21	skin prick test*.mp. or exp Skin Tests/
22	IgE.mp. or exp Immunoglobulin E/
23	oral food challenge*.mp.
24	21 or 22 or 23
25	exp Child/ or exp Cohort Studies/ or exp Child, Preschool/ or birth cohort*.mp. or exp Infant/
26	20 and 24 and 25

© 2026 Islam N et al. *JAMA Pediatrics*.

eTable 1. Study characteristics of the included studies addressing food allergy incidence and/or predictive factors.

Author-Year	Design	Country	Inclusion criteria	Cohort Year(s)	Age at diagnosis of FA (Years)	Sample Size of the Cohort (N)	Number of Males
Aksoy 2021[12]	Retrospective cohort	Turkey	General population; all healthy infants with regular well-child visits during the observation period.	2011-2012	Mean, 0.54	949	518
Alkazemi 2018[13]	Case-control	Kuwait	Pediatric patients registered with atopic diseases; healthy children attending the vaccination centers without chronic illnesses.	2015 (FA cases), 2016 (controls)	NR	132	NR
Allen 2013[14]	Prospective cohort	Australia	General population; infants between 11-15 months attending council-led immunization sessions	2007-2011	1	5276	NR
Arroyo 2023 (MARC-35)[15]	Prospective cohort	USA	Children <1 year hospitalized for bronchiolitis	2011-2014	1	921	NR
Ashley 2017[16]	Case-control	Australia	12-month-old infants presenting for scheduled immunizations at council-run clinics	2007-2011	1	533	NR
Azad 2015[17]	Prospective cohort	Canada	General population: pregnant women aged 18 years and older (19 in Vancouver); residence in reasonable proximity to the delivery hospital; willing to consent to cord blood collection for the study; planning to give birth at a designated recruitment centre participating hospital; infants born at or after 35 weeks	2009-2011	1	166	85
Baiz 2017[18]	Prospective cohort	France	General population: all women visiting the prenatal clinic.	2003-2006	5	247	NR
Bedolla-Barajas 2016[19]	Prospective cohort	Mexico	Children >16 years examined for the first time in an allergy clinic with a history compatible with asthma, allergic rhinitis, or atopic dermatitis and ≥1	2011-2012	2	304	NR

© 2026 Islam N et al. *JAMA Pediatrics*.

			positive SPT for an allergen				
Bedolla-Barajas 2018[20]	Case-control	Mexico	Children aged between 2 and 5, all of whom were referred to the allergy department after being diagnosed with an allergic disease (asthma, allergic rhinitis, or atopic dermatitis).	2012-2013	Range: 2-5	194	126
Best 2021[21]	Retrospective cohort	Australia	General population; mother–infant pairs >18y whose infant had a first-degree relative with a history of medically diagnosed allergic disease; recruited from local maternity antenatal clinics and classes	2011-2016	1	558	NR
Bock 1987[22]	Prospective cohort	USA	Infants who were consecutively born and received their newborn care at the Fort Collins Youth Clinic, Colorado, USA	1980-1981	3	480	NR
Bouma 2023 (PIAMA)[23]	Retrospective cohort	Netherlands	Children with at least one IgE measurement at age 4, 8, 12 or 16 and air pollution exposure estimates at birth address.	1996-1997	4	2278	NR
Brettig 2023[24]	Retrospective cohort	Australia	1-year-old infants with a SPT >1 mm from council-run immunization centers around Melbourne	2016-2019	1	1933	NR
Carlsten 2013[25]	Prospective cohort	Canada	General population; all preschoolers between 2-5 years recruited from 17 cities and towns in New Zealand	NR	1	373	199
Chandran 2013[26]	Cross-sectional	USA	General population; data from the National Health Interview Survey (NHIS); children aged 0–17 years	2005-2009	Range, 0-5	51748	NR
Chen Arroyo 2019[27]	Prospective cohort	USA	Cohort of infants enrolled during hospitalization for bronchiolitis	NR	Mean, 0.25	921	NR
CHILD 2024[1]	Prospective cohort	Canada	Women ≥ 18 years of age (≥19 years in Vancouver), residence within an acceptable distance of the delivery hospital, willing to	2008	3	3263	1670

© 2026 Islam N et al. *JAMA Pediatrics*.

			consent to cord blood collection for the study, considering giving birth at a designated recruitment center participating hospital				
Cho 2024[28]	Prospective cohort	Korea	General population; pregnant women at 28 weeks of gestation or later	NR	Range, 0.17-2	161	69
Clausen 2018 (EuroPrevall)[29]	Prospective cohort	Iceland	General population; all healthy infants	2005 - 2008	1	1304	668
Collier 2019 (BIS)[30]	Prospective cohort	Australia	General population; all healthy infants via unselected sampling frame in southeast Australia.	NR	1	1074	556
Davisse-Paturet 2019 (ELFE)[31]	Prospective cohort	France	Singleton or twins born after 33 weeks of gestation, to mothers aged 18 years or older and not planning to move outside of metropolitan France in the next 3 years	2011-2013	Mean, 0.17	11720	6029
Depner 2013 (PASTURE)[32-47]	Prospective cohort	Austria, Finland, France, Germany, Switzerland	General population; pregnant women in the third trimester of pregnancy.	2002-2005	Mean, 0.17	793	400
Desroches 2010[48]	Case-control	Canada	Cases were infants ≤18 months with suspected peanut allergy in the month before the visit; controls were healthy infants ≤18 months	1998-2004	1	403	222
Dhudasia 2021[49]	Retrospective cohort	USA	General population; all healthy infants born at gestational age ≥ 37 weeks and birth weight ≥2000g	2012-2017		14046	7136
Dogruel 2016 (ADAPAR)[50]	Prospective cohort	Turkey	General population	2010-2011	1	1377	731
Du Toit 2013[51]	Prospective cohort	UK	Children aged >4 or <11 months of age with egg allergy, severe eczema, or both; without peanut allergy	2006-2009	5	834	505
Ehlayel 2008[52]	Cross-sectional study	Qatar	General population	2006-2007	NR	1278	632
Elizur 2024[53]	Case-control	Israel	General population	2004-2006	1	433	240
Flohr 2014[54]	Prospective cohort	UK	General population; 3-month–old exclusively breastfed	2009-2012	0.25	619	NR
Fox 2009[55]	Case-control	UK	Children with peanut allergy (both high-risk and low-risk controls) in 1 large London pediatric	2004-2005	Mean, 2.17	443	NR

© 2026 Islam N et al. *JAMA Pediatrics*.

			department. Parents of cases and high-risk controls completed the questionnaire before knowing whether their child had peanut allergy. All children were younger than 48 months at recruitment.				
Gabet 2016 (PARIS)[56]	Prospective cohort	France	Birth cohort; full-term and healthy singletons born from parents living in Paris or close suburbs	2004-2008	Mean, 1.5	1860	926
Gao 2019[57]	Prospective cohort	China	General population; all women who delivered live-born babies in three Streets of Kaifu District, Changsha City.	2015	Mean, 0.08	976	467
Gao 2023 (BIS)[58]	Case-control	Australia	General population; infants with FA at age 1 year to random sample of infants.	2010-2013	1	323	176
Goldsmith 2016 (HealthNuts)[59]	Prospective cohort	Australia	General population; 12 month-old infants from immunisation clinics	2006–2009	1	4537	2314
Grimshaw 2015 (PIFA)[60,61]	Prospective cohort	UK	PIFA birth cohort of EuroPrevall; all eligible pregnant women registered with the Hampshire Hospitals Foundation Trust midwifery service with healthy infants.	2006-2008	1	1140	583
Grimshaw 2020[62]	Case-control	UK	General population; all health infants with a gestational age of >34 weeks and an Apgar score of >7 at 5 minutes after birth.	2005-2010	1	226	137
Gruber 2008[63]	Retrospective cohort	Australia, Austria, Belgium, Czech Republic, France, Germany, Italty, Netherlands, Poland, South Africa, Spain, United Kingdom	All infants with AD and a family history of allergy (first degree relative with a history of atopic dermatitis, bronchial asthma or hay fever), but no history of asthma or wheezing.	2002-2004	Range, 1-2	2184	NR

© 2026 Islam N et al. *JAMA Pediatrics*.

Halpern 1973[64]	Cohort	USA	General population; all infants between September, 1963, and July, 1966, by the 11 pediatricians from the study	1963-1966	Mean, 0.5	1753	893
Hesselmar 2010 (ALLERGYFLORA)[65]	Prospective cohort	Sweden	Families with at least 1 parent with a history of allergic disease; child born at term (~38 weeks gestational age) at Sahlgrenska University Hospital and did not receive mechanical ventilation.	1998-2003	1.5	184	93
Hong 2011 (CHS)[66,67]	Prospective cohort	USA	Birth cohort; multiethnic mother-infant pairs with primary or specialist care at Boston University Medical Center	NR	Mean, 0.5	970	486
Hong 2024 (BBC)[68]	Prospective cohort	USA	General population	1998	Range, 1.1-5.5	782	393
Hurley 2022 (CORAL)[69]	Prospective cohort	Ireland	General population; healthy infants	2020	1	344	189
Jones 2013[70]	Prospective cohort	Australia	Children with at least 1 parent with a history of allergic disease (eczema, asthma, or hay fever)	2002-2009	1	231	119
Jonsson 2017 (FARM-FLORA)[71]	Prospective cohort	Sweden	General population; children born within gestational weeks 36–42	2005-2008	3	120	64
Joseph 2011 (WHEALS)[72]	Prospective cohort	USA	General population; pregnant woman 21-50 years seen for prenatal care	2003-2007	2	1258	NR
Joseph 2016 (WHEALS)[73]	Prospective cohort	USA	General population; pregnant woman ages 21-50 years seen for prenatal care in 1 of 5 Henry Ford Hospital obstetric clinics; resided in Detroit/Detroit western suburbs	2003-2007	2	1258	312
Karpa 2012[74]	Case-control	USA	Children visiting either a primary care practice (controls) or allergy specialty clinic for a food allergy-related concern (cases) who were born at its medical center.	2000-2006	1.5	291	57.4
Kalb 2022[75]	Case-control	Germany	General population; all healthy infants at 4 German pediatric clinic	1990	2.36	1761	NR

© 2026 Islam N et al. *JAMA Pediatrics*.

Kawada 2020[76]	Retrospective cohort	Japan	Children with single or multiple atopic diseases aged 2–19 years	2015-2019	Range, 2-6	186	118
Keet 2012 (NHANES)[77]	Retrospective cohort	USA	General population; between 1-21 years old	2005-2006		3550	1832
Keet 2021[78]	Prospective cohort	USA	Infants with no history of peanut allergy with eczema, allergy, or relative with peanut allergy	2016-2019	Range, 0.33-0.92	325	186
Kelderer 2022 (NorthPop)[79]	Prospective cohort	Sweden	General population; all pregnant women in the catchment area of Umeå University Hospital ≥18 years of age, in gestational weeks 14– 24	2016-2018	1.5	1387	725
Keski-Nisula 2010[80]	Prospective cohort	Finland	Living on a farm with livestock, maternal age greater than 18 years, term delivery, no genetic disease in offspring, telephone connection, and sufficient knowledge of the country's language	2002-2005	1	388	193
Kikkawa 2018[81]	Prospective cohort	Japan	General population; all infants born between January 10-17 or July 10-17, 2001	2001	Mean, 0.5	47015	24425
Kim 2009[82]	Case-control	USA	General population; Chicago, children with and without FA	NR	3.6	562	NR
Kim 2011[83]	Prospective cohort	South Korea	General population; all healthy infants from pregnant women at >34 wk gestation at enrolment	2006-2007	0.33	1720	601
Kim 2015[84]	Case-control	South Korea	Children aged 0–10 years presenting with typical signs and symptoms of immediate type food allergies, who underwent allergy testing for the following foods: hen's eggs, cow's milk, peanuts, soybeans, and wheat by measuring food-specific serum IgE, and who had more than one clinic visit during the study period.	2008-2013	Mean, 1.6	126	75
Kim 2019 (COCOA)[85]	Prospective cohort	South Korea	General population: healthy infants of mothers who (1)	2007-2015	1	2512	765

© 2026 Islam N et al. *JAMA Pediatrics*.

			lacked high-risk conditions and (2) planned to deliver at affiliated medical centers				
Kiraly 2016 (HealthNuts)[86]	Prospective cohort	Australia	General population; all infants at immunization clinics across Melbourne, Australia without eczema before 3 months of age	2007-2011	1	5276	2233
Kjaer 2008 (DARC)[87]	Prospective cohort	Denmark	General population	1998-1999	6	404	200
Kljakovic 2009[88]	Prospective cohort	Australia	All new entrants to primary schools with parent-reported nut allergy who took part in the ACT Kindergarten Health Screen in 2005.	2005		4327	1880
Kojima 2024 (JECS)[89]	Prospective cohort	Japan	General population	2011-2014	4	74240	37974
Koksal 2023[90]	Case-control	Turkey	Children with IgE-mediated CMPA and exclusively breastfed until commencement of complementary feeding	2023	1	160	106
Koplin 2012 (HealthNuts)[91]	Prospective cohort	Australia	General population; 11-15-month-old infants	2007-2011	Range, 0.92-1.25	4963	2423
Koplin 2014 (HealthNuts)[92]	Prospective cohort	Australia	Birth cohort	2007-2011	1	5276	NR
Kotsapas 2022 (MAAS)[93]	Prospective cohort	United Kingdom	General population; ≤10 weeks of pregnancy, maternal age ≥18 years, and questionnaire/SPT data test available for both parents	1995	1	1184	514
Kull 2002[94]	Prospective cohort	Sweden	General population; children born in predefined areas of Stockholm,	1994-1996	1	4089	2065
Kumar 2009 (BBC)[95]	Prospective cohort	USA	General population; any woman admitted to the labor and delivery floor at BMC who delivered a singleton live infant; case (gestational age <37 weeks or birth weight <2,500 g); control (gestational age <37 weeks with birth weight <2,500 g)	1998	3	1262	631
Kumar 2010[96]	Prospective cohort	USA	General population; all healthy infants	NR	6	960	539
Kuwabara 2020[97]	Retrospective cohort	Japan	General population; children born in an obstetric/pediatric	2013-2014	0.08	612	269

© 2026 Islam N et al. *JAMA Pediatrics*.

			clinic in a rural area in Mie Prefecture, whose mothers visited the clinic for a health checkup 1 month after birth				
Lamminsalo 2022 (DIPP)[98]	Prospective cohort	Finland	Children carrying genotypes conferring high/moderate risk for type 1 diabetes	1997-2004	3	4921	2590
Landau 2023 (LEAP)[99]	Retrospective cohort	UK	Infants with severe eczema and/or egg allergy between 4-11 months	2006-2009	Range, 1-5	281	177
Laubereau 2004 (GINI)[100]	Prospective cohort	Germany	Birth cohort; healthy full-term neonates with a family history of allergy in two regions in Germany for the German Infant Nutritional Intervention Program	1995-1998	1	889	433
Lee 2022[101]	Prospective cohort	Singapore	General population	2011-2015	Range, 1-2.5	4115	5
Lee-Sarwar 2023[102]	Case-control	USA	Offspring of participants in VDAART, a RCT of Vitamin D supplementation during pregnancy	NR	Range, 0-6	614	343
Leung 2009[103]	Cross-sectional	Hong Kong	General population; all children from selected nurseries and kindergartens	2006-2007	Range, 2-7 (≥6 years of age is 2.2%)	3827	1856
Levin 2020 (SAFFA)[104]	Cross-sectional	South Africa	General population; urban and rural toddlers	NR	2.16 (urban), 1.75 (rural)	1583	847
Liao 2014 (PATCH)[105,106]	Prospective cohort	Taiwan	General population; pregnant women during their third-trimester clinical visits; excluding neonates with congenital anomaly or those with gestational age <37 weeks	NR	0.5	258	125
Liem 2007 (SAGE)[107]	Prospective cohort	Canada	General population; all children born in 1995.	1995-2002	Range, 0-5	13980	NR
Liu 2010 (NHANES)[108]	Prospective cohort	USA	General population; participants (1-85 years) in the National Health and Nutrition Examination Survey (NHANES) 2005-2006	2005-2006	Range, 1-5	8203	3998
Liu 2011 (BBC)[109]	Prospective cohort	USA	General population; mother-infant pairs followed prospectively at the Boston Medical Center.	NR	Mean, 2.1	649	329

© 2026 Islam N et al. *JAMA Pediatrics*.

Liu 2013 (BBC)[110]	Prospective cohort	USA	General population; mother-infant pairs followed prospectively at the Boston Medical Center.	NR	3	460	NR
Loo 2016 (GUSTO)[111]	Prospective cohort	Singapore	General population; all healthy pregnant mothers	NR	1.5	1247	402
Lopez 2024 (HealthNuts)[112]	Prospective cohort	Australia	General population; infants	2007-2011	1	4668	2374
Love 2016[113]	Case-control	USA	Children in 2010 with an office visit or hospitalization associated with specific ICD9-CM codes attributed to food allergy; controls were aged 0-3, enrolled in SC Medicaid in 2010 and never had a diagnosis of food allergy; all children enrolled in SC Medicaid for their first year of life	2007-2009	Range, 0-3	7499	4186
Lowe 2007 (MACS)[114]	Prospective cohort	Australia	Infants with mothers attending the Mercy Hospital Antenatal Clinic, and one or more of their first-degree family members with eczema, asthma, hay fever or severe FA	1990-1994	1	552	318
Loewen 2021[115]	Prospective cohort	Canada	Mother-child dyads with maternal allergen consumption prospectively reported at recruitment in the second or third trimester and infant skin prick testing data to allergens including egg and peanut at 1 and 3 years.	2008-2012	3	3412	NR
Luccioli 2014 (Y6FU)[116]	Prospective cohort	USA	Mother-infant pairs with no medical condition within 6 months that would affect feeding; all singleton infants after 35 weeks' gestation, weigh >5lb, and not have stayed in intensive care for >3 days	2005-2007	6	1542	688
Ma 2021[117]	Cross-sectional	China	General population	2019	Range, 0-2	513	268
Mai 2010 (BAMSE)[118]	Prospective cohort	Sweden	General population: all newborn children living in certain areas	1995-1996	4	4089	NR

© 2026 Islam N et al. *JAMA Pediatrics*.

			of northern and central Stockholm				
Martin 2015 (HealthNuts)[119]	Prospective cohort	Australia	General population: infants between 11-15 months with SPT reaction to food	2007-2011	1	4453	2253
Martone 2023 (Project Viva)[120]	Prospective cohort	USA	General population	1999-2002	2	1159	NR
Mathias 2019[121]	Prospective cohort	USA	General population: pregnant women in their third trimester; all healthy singleton infants	2005-2007	0.30	1542	769
Matsumoto 2020[122]	Retrospective cohort	Japan	General population: all infants born in Japan between 10-17 January or 10-17 July in 2001	2001	0.50	46,616	24220
Marenholz 2018 (GOFA)[123]	Case-control	Germany	Children's visiting pediatric clinics in Germany	NR	5	902	577
McGowan 2015 (URECA)[124]	Prospective cohort	USA	Pregnant women in Baltimore, Boston, New York City, and St Louis with a mother or father with a history of allergic rhinitis, eczema, or asthma; gestational age of 34 weeks or greater; and collection of a suitable cord blood sample	2005-2007	1	516	268
McGowan 2018 (BBC)[125,126]	Case-control	USA	General population: mother-infant pairs enrolled in the CHS	2004	Mean, 2.4	1394	713
McMilin 2016[127]	Prospective cohort	New Zealand	General population	2009-2010	2	6853	NR
Metsala 2010[128]	Case-control	Finland	Infants born in Finland up to 2 years who need special infant formulas for management of diagnosed CMA by the end of November 2005	1996-2004	2	32474	NR
Metsala 2013[129]	Case-control	Finland	General population: all infants born in Finland	1996-2004	2	32474	9547
Metzler 2019 (PASTURE)[130]	Prospective cohort	Austria, Finland, France, Germany, Switzerland	General population: children living in rural areas	2002-2005	1	1080	560
Miliku 2018 (CHILD)[131]	Prospective cohort	Canada	Birth cohort: pregnant women between 2008 and 2012 across four Canadian sites (Vancouver, Edmonton, Manitoba, and Toronto); mother-	2008-2012	1	421	218

© 2026 Islam N et al. *JAMA Pediatrics*.

			infant dyads that breastfed for at least 3 months				
Miliku 2021 (CHILD)[132]	Prospective cohort	Canada	Birth cohort: pregnant women between 2008 and 2012 across four Canadian sites (Vancouver, Edmonton, Manitoba, and Toronto); mother-infant dyads that breastfed for at least 3 months	2008-2012	1	1109	606
Milner 2004 (NMIHS)[133]	Prospective cohort	USA	General population: mothers who gave birth in 1988	1988-1991	3	8285	4225
Mitre 2018 (TRICARE MHS)[134]	Retrospective cohort	USA	General population: all healthy infants with records and data until the age of 1 year old	2001-2013	Mean, 0.5	792130	396915
Mitselou 2018[135]	Prospective cohort	Sweden	General population: all children born in Sweden in the MBR, excluding subjects who died before 60 days or had a diagnosis of FA in the first 2 months of life	2001-2012	1	1086378	NR
Mitselou 2022 (BAMSE)[136]	Prospective cohort	Sweden	Infants born in predefined areas of Stockholm	1994-1996	4	3522	1757
Molloy 2020 (BIS)[137]	Prospective cohort	Australia	General population: all healthy infants of mothers in the Barwon Statistical Division, pregnant at <28 weeks of gestation, planning to give birth at either Geelong Hospital (public) or St John of God Hospital (private)	2010-2013	1	863	NR
Mullins 2012[138]	Case-control	Australia	Peanut allergic patients <72 months.	2003-2010	1	115	51
Negele 2004[139]	Prospective cohort	Germany	General population: healthy full-term neonates from four chosen German cities	1997-1999	2	2500	1295
Nicklaus 2019 (PASTURE)[44]	Prospective cohort	Austria, Finland, France, Germany, Switzerland	General population: children from rural areas in 5 European countries (Austria, Finland, France, Germany, and Switzerland)	2002-2005	1	931	471
Nwaru 2010a (DIPP)[140]	Prospective cohort	Finland	All newborn infants with human leukocyte antigen (HLA)-conferred	1998-2000	5	1067	551

© 2026 Islam N et al. *JAMA Pediatrics*.

			susceptibility to T1 diabetes from the university hospital areas of Turku, Oulu, and Tampere				
Nwaru 2010b (DIPP)[141]	Prospective cohort	Finland	All newborn infants with human leukocyte antigen (HLA)-conferred susceptibility to T1 diabetes from the university hospital areas of Turku, Oulu, and Tampere	1998-2000	5	1018	551
Nwaru 2011 (DIPP)[142]	Prospective cohort	Finland	All newborn infants with HLA-DQB1-conferred susceptibility to type 1 diabetes are recruited from the university hospital areas of Finland (Turku, Oulu, and Tampere)	1998-2000	5	1018	551
Nwaru 2013 (DIPP)[143]	Prospective cohort	Finland	Infants born with human leukocyte antigen (HLA)-conferred susceptibility to T1 diabetes from 3 university hospitals in Finland (Turku, Oulu, and Tampere)	1996-2004	5	3675	1930
Panjari 2016[144]	Prospective cohort	Australia	General population: all children beginning primary school in the state of Victoria, Australia, during 2010	2010	5	57005	28476
Papathoma 2016[145]	Prospective cohort	Greece	General population: children born at ≥34 weeks of gestation at the University Hospital of Patras	2009-2011	Range, 0.01-3	459	246
Pelak 2021 (M2M)[146]	Prospective cohort	USA	General population: women >18 years of age who delivered a singleton, liveborn infant at >24 weeks of gestation at The Ohio State University Wexner Medical Center; excluding women with the intention to exclusively "bottle feed"	2011	6	305	150
Pele 2013 (PELAGIE)[147]	Prospective cohort	France	General population: all pregnant women <19 weeks of gestation in 3 districts of Brittany, France	2002-2006	2	1500	772
Perez-Chacon 2024[148]	Retrospective cohort	Australia	Children with priming dose, but no	1997-1999	<5	218,093	NR

© 2026 Islam N et al. *JAMA Pediatrics*.

			evidence of $2^{nd}/3^{rd}$ pertussis vaccine dose				
Perkin 2021 (EAT)[149]	Prospective cohort	United Kingdom	General population: all singleton infants 3 months of age who were healthy, exclusively breastfed, and born at term (>37 weeks' gestation)	2009-2012	1	1303	NR
Pesonen 2006[150]	Prospective cohort	Finland	Healthy full-term infant with appropriate weight for gestational age, a 1-min Apgar score of at least 8, and a healthy, non-smoking mother with uncomplicated pregnancy and delivery.	1981	5	200	NR
Peters 2015 (HealthNuts)[151]	Prospective cohort	Australia	General population: all infants aged 11-15 months, recruited through council-run immunisation sessions	2007-2011	1	5276	2226
Polaskey 2023[152]	Retrospective cohort	USA	Children with ICD 9/10 diagnosis of AD	2007-2021	≤3	109	66
Ponsonby 2022 (BIS)[153]	Prospective cohort	Australia	General population; healthy infants	2010-2013	1	1074	519
Pretorius 2024[154]	Prospective cohort	Australia	Infants born ≥36 weeks gestation with 1+ immediate family member with a history of allergic disease	2011-2016	1	639	333
Pyrhonen 2011 (SKARP)[155]	Prospective cohort	Finland	General population; all infants born in 2001 residing in South Karelia, Finland	2001-2006	Range, 0-4	3800	NR
Pyrhonen 2013 (SKARP)[156]	Prospective cohort	Finland	General population; all children, from the province of South Karelia from 1-4 years old when their parents were invited	2001-2005	0.5	3181	1620
Pyrhonen 2020 (SKARP)[157]	Prospective cohort	Finland	General population; all children born between April 2001 and March 2006 who resided in South Karelia, Finland one month before the time of the questionnaire	2001-2006	Range, 0-4	3035	1546
Roduit 2014 (PASTURE/EFRAIM)[33]	Prospective cohort	Austria, Finland, France, Germany, Switzerland	General population; rural-living pregnant women during the third trimester of pregnancy between	2002-2005	Range, 0-6	856	432

© 2026 Islam N et al. *JAMA Pediatrics*.

			August 2002 and March 2005				
Roduit 2017 (PASTURE)[43]	Prospective cohort	Austria, Finland, France, Germany, Switzerland	General population; pregnant women in rural areas	2002-2005	Range, 0-2	1038	532
Röhrl 2021 (EuroPrevall)[158]	Prospective cohort	Germany	KUNO study-all mothers who gave birth at St. Hedwig in Regensburg; Ulm SPATZ study-general population; born at the University Medical Centre Ulm.	2015-2019 (KUNO), 2012-2013 (Ulm SPATZ)	1	2145	1092
Rosas-Salazar 2022 (INSPIRE)[159]	Prospective cohort	USA	Healthy term infants	2012-2013	1	1949	1020
Ruohomaki 2021 (KuBiCo)[160]	Prospective cohort	Finland	All women who are likely to give birth at Kuopio University Hospital	2012-2016	1	969	510
Saarinen 2000[161]	Prospective cohort	Finland	Healthy, full-term newborn infants	1994-1995	1	6209	NR
Sakihara 2016[162]	Prospective cohort	Japan	Patients with hen's egg allergy at the Aichi Children's Health and Medical Center at ≤6 years of age.	2012-2015	Range, 0.25-2	374	244
Sánchez-Valverde 2009[163]	Prospective cohort	Spain	Children diagnosed with cow's milk allergy at the Gastroenterology and Nutrition Unit of a referral hospital in a region of Spain.	1998-2002	0.3	225	103
Sato 2023 (CHIBA)[164]	Prospective cohort	Japan	Children with family history of allergy	2001	Range, 2-5	263	132
Sausenthaler 2007 (LISA)[165]	Prospective cohort	Germany	Birth cohort; all infants who were followed-up at 2 years without chronic disease	1997-1999	2	2641	1360
Savage 2012 (NHANES)[166]	Prospective cohort	USA	General population; from non-institutionalized US population; children aged 6-18 years old	2005-2006	6	859	450
Sbihi 2015 (CHILD)[167]	Prospective cohort	Canada	General population; pregnant mothers ≥18 years of age (19 years in Vancouver) and give birth at one of the recruitment centers; infants had to be born at ≥ 35.5 weeks gestation with weight ≥ 2,500 g	2008-2012	1	2482	1282
Seay 2022 (GMAP)[168]	Prospective cohort	USA	Birth cohort; all newborn infants at their first office visit at a single private primary pediatrics	2014-2017	Range,0.1-3	797	430

© 2026 Islam N et al. *JAMA Pediatrics*.

			office (Pediatrics at Newton Wellesley) up to 2 months of age				
Shek 2010[169]	Retrospective cohort	Australia, the Philippines	General population: students from 4-6 years old	2007-2008	Range, 4-6	23425	10653
Shibuya 2013[170]	Prospective cohort	Japan	General population; all infants born at the East Japan Kanto Hospital	2008-2009	0.25	317	174
Shoda 2016 (T-CHILD)[171]	Prospective cohort	Japan	General population; all pregnant women who visited NCCHD before the 16th week of gestation and delivered at the centre	2003-2005	1	1330	699
Sicherer 2010 (COFAR)[172]	Prospective cohort	USA	Children with a history of a convincing immediate allergic reaction to cow's milk (and/or egg) and a positive SPT, and/or (2) moderate to severe atopic dermatitis (AD) and a positive SPT to milk and/or egg	NR	Range, 0.25-1.25	503	342
Sicherer 2019 (COFAR2)[173]	Prospective cohort	USA	Infants with convincing allergic reaction to milk and/or egg with a positive prick SPT, and/or moderate to severe AD and a positive SPT to milk and/or egg; without peanut allergy	2006-2008	Range, 0.25-1.25	511	345
Sidorchuk 2004 (BAMSE)[174]	Prospective cohort	Sweden	Birth cohort; infants born from February 11, 1994, until November 22, 1996, in Stockholm	1994-1996	4	2581	1308
Simons 2020 (CHILD)[175]	Prospective cohort	Canada	General population; all healthy infants at 4 sites (Vancouver, Edmonton, Winnipeg/Morden-Winkler, and Toronto)	2008-2012	1	2434	NR
Smejda 2018[176]	Prospective cohort	Poland	Women with ingle pregnancy up to 12 weeks of gestation, no assisted conception, no pregnancy complications, and no chronic diseases	2007	1	370	NR
Snijders 2008 (KOALA)[177]	Prospective cohort	Netherlands	General population; pregnant women from an ongoing prospective cohort study in the	2000	2	2558	1083

© 2026 Islam N et al. *JAMA Pediatrics*.

			Netherlands, excluding infants with Down syndrome and eczema.				
Soriano 2021 (BIS)[178]	Prospective cohort	Australia	BIS birth cohort; all healthy infants from two main hospitals in the Barwon region in southeast Australia; pregnant mothers >18 and at <28 weeks' gestation	2010	1	787	401
Soriano 2023 (EarlyNuts)[179]	Cross-sectional	Australia		2017-2019	Range 0.92-1.25	1419	743
Stokholm 2017 (COPSAC2000)[180]	Prospective cohort	Denmark	Children born to mothers with doctor-diagnosed asthma; excluding children with gestational age less than 36 weeks, severe congenital abnormalities, or any lung symptoms	1998-2001; 1978-2011	Range, 0.5-6	4788	NR
Suaini 2021 (GUSTO)[181]	Prospective cohort	Singapore	General population; women in their first trimester of pregnancy and their infants from the two major public obstetric hospitals in Singapore and Children's Hospital.	2009-2010	Range, 1.16-1.5	878	169
Suaini 2024 (GUSTO)[182]	Prospective cohort	Singapore	General population; pregnant women aged 18-50, spouses of homogenous ethnicity	2009-2010	1.5	728	372
Sugiura 2021[183]	Cross-sectional	Japan	NR	2016-2017	NR	18549	9840
Sung 2022 (PATCH)[184]	Prospective cohort	Taiwan	Children who completed a 4-year follow-up period.	2007-2008	2	183	101
Tan 2012[185]	Case-control	Australia	Wwhite infants only (infants with 1 or both parents born in Australia, Europe, the United Kingdom, North America, or New Zealand) for whom DNA was available for analysis.	2008-2011	1	554	NR
Tanaka 2015[186]	Retrospective cohort	Japan	Patients who had visited the outpatient allergy clinic from 2006-2010 living in Aichi prefecture who visited us before 2 years	2006-2010	0.5	772	484
Tanaka 2021 (KOMCHS)[187]	Prospective cohort	Japan	General population; pregnant women in Kyushu Island/Okinawa	2007-2008	2	1522	737

© 2026 Islam N et al. *JAMA Pediatrics*.

			Prefecture in southern Japan and their children.				
Tedner 2021 (PreventADALL)[188]	Prospective cohort	Norway, Sweden	General population; infants who had available serum for IgE analyses and their mothers at 3 months	2014-2016	0.25	1100	NR
Thacher 2016 (BAMSE)[189]	Retrospective cohort	Sweden	NR	1994-1996	4	3316	NR
Thijs 2011 (KOALA)[190]	Prospective cohort	Netherlands	General population; all children with availability of a breast milk sample	2002-2003	1	315	71
Toelle 2020 (CAPS)[191]	Prospective cohort	Australia	General population; children with their first dose of pertussis vaccine between the ages of 6-18 weeks	1997-2000	5	497	NR
Tokinobu 2020[192]	Prospective cohort	Japan	General population; all children in Japan who were born during 10–17 January or 10–17 July in 2001	2001	1.5	43783	22742
Tuokkola 2016b (DIPP)[193]	Prospective cohort	Finland	Caucasian families with healthy infants carrying human leukocyte antigen genotypes conferring high and moderate risk	1997-2004	0.5	6288	3304
Tuokkola 2021 (DIPP)[194]	Prospective cohort	Finland	Caucasian families with healthy infants carrying human leukocyte antigen genotypes conferring high and moderate risk	1997-2004	0.5	4403	3304
Ushiyama 2002[195]	Retrospective cohort	Japan	General population; all mothers who participated in a regular infant health examination held by the Pediatric Association of Kochi Prefecture	2000-2001	1	2070	NR
Van den Berg 2011[196]	Prospective cohort	Netherlands	Patients were seen at a tertiary care pediatric allergy outpatient clinic because of suspected FA	2001-2006	Mean, 5.4	396	251
van Nimwegen 2011 (KOALA)[197]	Prospective cohort	Netherlands	General population; all pregnant women at 34 weeks of gestation; newborns with congenital abnormalities, premature newborns (gestational age <37 weeks), and who received antibiotics	2002	1	2834	1385

© 2026 Islam N et al. *JAMA Pediatrics*.

			in the first month of life were excluded				
Venero-Fernandez 2018[198]	Prospective cohort	Cuba	General population; children aged from 1-3	2010-2013	1	1543	790
Venter 2009[199]	Prospective cohort	United Kingdom	General population; pregnant mothers with an estimated delivery time between September 2001 to August 2002 at antenatal clinics	2001-2002	1	969	500
Venter 2016 (FAIR)[200]	Case-control	United Kingdom	General population; all pregnant mothers with an estimated delivery date of September 2001 to August 2002 at antenatal clinics	2001-2002	1	117	71
Venter 2021 (Healthy Start)[201]	Prospective cohort	USA	General population; pregnant women aged >16 with singleton pregnancies from obstetrics clinics at the local hospital	2009-2014	Range, 0-4	1410	652
Venter 2024 (Healthy Start)[202]	Prospective cohort	USA	General population; mother-offspring dyads	2009-2014	Range, 0.5-4	967	507
Verhoeven 2023 (PeanutNL)[203]	Prospective cohort	Netherlands	Children aged 4-12 months with moderate-to-severe eczema (SCORAD ≥15), previous immediate reactions to another food than peanut, or a first-degree family member with systemic reactions to peanut	2018-2021	0.5	707	424
Vernon 2012 (NHANES)[204]	Cross sectional study	USA	All participants who had dichlorophenols and allergen specific IgE levels measured	2005-2006	Mean, 3.3	2548	1083
Venkatamaran 2014 (IOWBC)[205]	Prospective cohort	United Kingdom	All children consecutively born on the Isle of Wight, UK between 1 January 1989 and 28 February 1990	1989-1990	NR	1536	NR
Wang 2018[206]	Cross sectional study	Australia	General population; all children entering primary school (preparatory grade students) in Victoria	2010	4.9	66444	28476
Wärnberg Gerdin 2022 (PreventADALL)[207]	Retrospective cohort	Norway, Sweden	General population; all healthy infants	2014-2016	0.5	2397	1047
Wen 2023 (IFPS II)[208]	Retrospective cohort	USA	General population	2005-2007	6	1252	620
West 2012[209]	Prospective cohort	Australia	All pregnant women with a family history of allergic rhinitis,	2005-2008	1	450	160

© 2026 Islam N et al. *JAMA Pediatrics*.

			asthma, eczema, food, or other allergy				
Wetzig 2000 (LARS)[210]	Prospective cohort	Germany	Newborns in one calendar year in the City and District of Leipzig with their family history of atopic disorders	1995-1996	1	475	NR
Winters 2019[211]	Retrospective cohort	England	Participants that completed the LEAP study, followed the protocol to avoid or consume peanuts according to their randomization assignment, and consented to genetic studies	2006-2009	5	542	316
Wright 2001[212]	Prospective cohort	USA	General population; newborns enrolled at birth	1980-1984	6	1246	NR
Yang 2019[213]	Cross sectional study	China	Infants ≤12 months of age during the survey	2014-2015	1	6768	3702
Yuenyongviwat 2024[214]	Cross sectional study	Thailand	Children with atopic dermatitis attended the pediatric allergy clinic at tertiary referral center.	2016-2017	Range, 2-5	119	75
Zeng 2015[215]	Cross sectional study	China	General population; children from 24 kindergartens in Guangdong; excluded those with skin rash and/or gastrointestinal reactions that were associated with infection	2013	4.6	2540	1331
Zhou 2017[216]	Prospective cohort	France	No twin pregnancy, no known diabetes before pregnancy, not planned to move outside of the region in the next 3 years and being able to speak or read French	2003	1	1139	607

© 2026 Islam N et al. *JAMA Pediatrics*.

eTable 2. Risk of bias assessment of included studies for the outcome of developing food allergy.

Study	Study participation	Study Attrition	Prognostic Factor Measurement	Outcome Measurement	Study Confounding	Statistical Analysis	Overall Risk of Bias
Aksoy 2021	Low	Probably low	Probably low	Low	Probably low	Probably low	Probably low
Alkazemi 2018	Probably low	Probably high	Probably low	Low	Probably low	Low	Probably high
Allen 2013	Low	Probably high	Probably low	Low	Probably low	Low	Probably high
Arroyo 2023	Probably high	Probably low	Probably low	Probably low	Probably low	Probably low	Probably high
Ashley 2017	Low	Low	Low	Low	Low	Probably high	Probably high
Azad 2015	Probably low	Probably low	Probably low	Low	Probably high	Probably low	Probably high
Baiz 2017	Probably low	Probably low	Low	Low	Low	Low	Probably low
Bedolla-Bajaras 2016	Low	Low	Probably low	Low	Low	Low	Probably low
Bedolla-Barajas 2018	Probably low	Probably low	Probably low	Probably low	Probably low	Probably low	Probably low
Best 2021	Low	Low	Low	Low	Probably low	Low	Probably low
Bock 1987	Low	Low	NA	Low	NA	Low	Low
Bouma 2023	Probably low	Probably low	Probably low	Probably high	Probably low	Probably low	Probably high
Brettig 2023	Probably low	Probably low	Probably low	Probably low	Probably high	Low	Probably high
Carlsten 2013	Probably high	Probably low	Probably low	Probably low	Probably high	Low	Probably high
Chandran 2013	Low	Low	Low	Probably low	Probably low	Low	Probably low
ChenArroyo 2019	Low	Probably low	Low	Low	Probably low	Probably low	Probably low
CHILD 2024	Low	Probably low	Low	Probably low	Low	Probably low	Probably low
Cho 2024	Probably high	Probably low	NA	Low	NA	Probably low	Probably high
Clausen 2018	Probably low	Probably low	NA	Low	NA	Probably high	Probably high
Collier 2019	Probably high	Probably low	Low	Low	Probably low	Probably low	Probably high
Davisse-Paturet 2019	Probably low	Probably low	Probably high	Low	Probably low	Probably low	Probably high
Depner 2013	Probably low	Probably low	Probably high	Probably low	Probably low	Probably low	Probably high
Desroches 2010	Low	Probably low	Probably high	Low	Probably high	Low	Probably high
Dhudasia 2021	Low	Low	Low	Probably low	Low	Low	Probably low
Dogruel 2016	Probably low	Probably low	Probably low	Low	Probably low	Probably low	Probably low
Du Toit 2013	Low	Probably low	Probably high	Probably low	Low	Probably high	Probably high
Ehlayel 2008	Probably low	Probably low	Probably high	High	Probably low	Probably high	High
Elizur 2024	Probably low	Probably low	Probably high	Probably low	Probably low	Probably low	Probably high
Flohr 2014	Probably high	Low	Probably low	Low	Probably low	Low	Probably high
Fox 2009	Probably low	Low	Probably high	Low	Probably low	Probably high	Probably high

© 2026 Islam N et al. *JAMA Pediatrics*.

Gabet 2016	Low	Probably low	Low	Low	Low	Probably low	Probably low
Gao 2019	Probably low	Probably low	Low	Probably high	Probably low	Low	Probably high
Gao 2023	Probably low	Probably low	Probably high	Probably low	Probably low	Probably low	Probably high
Gerdin 2022	Low	Probably low	Low	Probably low	Probably low	Probably high	Probably high
Goldsmith 2016	Probably low	Probably low	Probably low	Probably low	Probably low	Probably low	Probably low
Grimshaw 2015	Low	Probably high	NA	Low	NA	Probably low	Probably high
Grimshaw 2020	Low	Low	Probably low	Low	Low	Low	Probably low
Gruber 2008	Probably low	Probably low	Probably low	Probably low	Probably low	Probably high	Probably high
Halpern 1973	Probably high	Probably low	NA	Probably low	NA	Probably high	Probably high
Hesselmar 2010	Probably low	Probably low	NA	Low	NA	Probably low	Probably low
Hong 2011	Low	Low	Probably low	Probably low	Probably low	Low	Probably low
Hong 2024	Probably low	Probably low	Low	High	Probably low	Probably low	Probably high
Hurley 2022	Probably low	Probably low	NA	Low	NA	Probably low	Probably low
Jones 2013	Probably low	Probably low	Probably high	Probably low	Probably low	Probably low	Probably high
Jonsson 2017	Probably low	Probably low	Probably low	Probably low	Probably low	Probably low	Probably low
Joseph 2011	Probably low	Probably low	Probably low	Probably high	Low	Probably low	Probably high
Joseph 2016	Probably low	Probably low	Probably high	Probably low	Probably low	Probably low	Probably high
Kalb 2022	Probably low	Low	Low	Low	Low	Probably low	Probably low
Karpa 2012	Low	Low	Probably low	Probably high	Probably low	Low	Probably high
Kawada 2020	Probably low	Probably low	Probably high	Probably high	Probably low	Probably low	Probably high
Keet 2012	Probably low	Low	Probably low	Low	Probably low	Probably low	Probably low
Keet 2021	Probably low	Probably low	Probably low	Probably low	Probably low	Probably low	Probably low
Kelderer 2022	Low	Low	Probably low	Low	Probably low	Probably low	Probably low
Keski-Nisula 2010	Low	Probably low	Low	High	Probably low	Low	High
Kikkawa 2018	Probably low	Probably high	Probably high	Probably high	Probably high	Probably low	Probably high
Kim 2009	Probably high	Low	Probably low	Probably low	Probably low	Probably low	Probably high
Kim 2011	Probably low	Probably low	Low	Probably low	Probably low	Probably low	Probably low
Kim 2015	Probably low	Probably low	Probably high	Probably high	Probably low	Probably high	Probably high
Kim 2019	Probably low	Probably high	Probably low	Probably low	Probably low	Probably low	Probably high
Kiraly 2016	Probably low	Probably high	Probably low	Low	Probably high	Probably low	Probably high
Kjaer 2008	Probably high	Probably high	NA	Probably low	NA	Probably low	Probably high
Kljakovic 2009	Probably low	Probably low	Probably low	Probably high	Probably low	Probably low	Probably high
Kojima 2024	Probably low	Probably low	Probably high	High	Probably low	Probably low	Probably high

© 2026 Islam N et al. *JAMA Pediatrics*.

Koksal 2023	Low	Low	Probably low	Low	Probably high	Probably low	Probably high
Koplin 2012	Low	Probably low	Probably high	Low	Probably low	Probably low	Probably high
Koplin 2014	Probably low	Probably low	NA	Low	NA	Probably low	Probably low
Kotsapas 2022	Low	Probably low	Probably high	Low	Probably low	Low	Probably high
Kull 2002	Probably low	Probably low	Probably low	Probably low	Probably low	Probably low	Probably low
Kumar 2009	Probably low	Probably low	Probably low	Probably low	Probably low	Low	Probably low
Kumar 2010	Probably low	Probably low	Probably high	Probably low	Probably low	Low	Probably high
Kuwabara 2020	Probably low	Probably low	Low	Probably low	Probably low	Probably low	Probably low
Lamminsalo 2022	Low	Probably low	Probably low	Low	Probably low	Low	Probably low
Landau 2023	Probably low	Probably low	Probably low	Probably low	Probably low	Probably low	Probably low
Laubereau 2004	Probably low	Probably low	Probably high	Low	Probably low	Probably low	Probably high
Lee 2022	Probably low	Probably low	Probably low	Probably low	Probably low	Probably low	Probably low
Lee-Sarwar 2023	Probably low	Probably low	Probably low	Probably high	Probably low	Probably low	Probably high
Leung 2009	Probably low	Probably low	Probably high	Probably high	Probably low	Probably low	Probably high
Levin 2020	Probably low	Probably low	NA	Probably low	NA	Probably low	Probably low
Liao 2014	Probably low	Probably low	Low	Low	Low	Low	Probably low
Liem 2007	Probably low	Low	Low	Probably low	Probably low	Probably low	Probably low
Liu 2010	Probably low	Probably low	Probably low	Probably high	Probably low	Probably low	Probably high
Liu 2011	Probably low	Probably low	Low	Probably high	Probably low	Probably low	Probably high
Liu 2013	Probably high	Probably low	Probably high	Probably low	Probably low	Probably high	Probably high
Loewen 2021	Probably low	Low	Low	Low	Low	Low	Probably low
Loo 2016	Probably low	Probably low	Probably high	Probably low	Probably low	Probably low	Probably high
Lopez 2024	Low	Low	Low	Low	Probably low	Probably low	Probably low
Love 2016	Low	Low	Probably low	Probably low	Probably low	Probably low	Probably low
Lowe 2007	Low	Probably high	Probably low	Low	Probably low	Probably low	Probably high
Luccioli 2014	Probably low	Probably low	Probably low	Probably high	Probably low	Probably low	Probably high
Ma 2021	Probably low	Probably low	NA	Low	NA	Probably low	Probably low
Mai 2010	Probably low	Probably low	Probably low	Low	Probably low	Probably low	Probably low
Marenholz 2018	Probably low	Probably low	Probably low	Low	Probably high	Probably high	Probably high
Martin 2015	Low	Low	Low	Probably high	Probably low	Low	Probably high
Martone 2023	Probably low	Probably high	Probably high	Probably high	Low	Probably low	Probably high
Mathias 2019	Low	Low	Probably low	Probably high	Probably low	Low	Probably high
Matsumoto 2020	Low	Low	Probably low	Probably low	Low	Low	Probably low

© 2026 Islam N et al. *JAMA Pediatrics*.

McGowan 2015	Low	Probably low	Low	Probably low	Probably low	Probably low	Probably low
McGowan 2018	Low	Probably low	Probably low	Probably low	Probably low	Low	Probably low
McMilin 2016	Probably high	Probably low	Probably high	High	Probably high	High	High
Metsala 2010	Low	Probably low	Probably high	Probably low	Probably low	Probably low	Probably high
Metsala 2013	Low	Probably low	Probably high	Low	Probably low	Low	Probably high
Metzler 2019	Probably low	Probably low	Probably high	Probably low	Low	Low	Probably high
Miliku 2018	Probably high	Probably low	Probably low	Low	Probably low	Probably low	Probably high
Miliku 2020	Low	Low	Low	Probably low	Probably high	Low	Probably high
Milner 2004	Probably low	Probably low	Probably high	Probably high	Probably low	Probably low	Probably high
Mitre 2018	Probably high	Low	Low	Probably low	Probably low	Low	Probably high
Mitselou 2018	Probably high	Probably low	Probably high	Probably low	Probably low	Low	Probably high
Mitselou 2022	Probably low	Probably low	Probably low	Probably low	Probably low	Probably low	Probably low
Molloy 2020	Probably low	Probably low	NA	Low	NA	Probably low	Probably low
Mullins 2012	Probably low	Low	Probably high	Probably low	Low	Low	Probably high
Negele 2004	Low	Probably low	Probably low	Probably low	Probably low	Probably low	Probably low
Nicklaus 2019	Probably low	Probably low	Probably high	Low	Probably low	Low	Probably high
Nwaru 2010a	Probably low	Probably low	Probably high	Probably high	Low	Low	Probably high
Nwaru 2010b	Probably low	Probably low	Probably high	Low	Probably low	Low	Probably high
Nwaru 2011	Probably low	Probably low	Probably low	Probably high	Low	Probably low	Probably high
Nwaru 2013	Low	Probably low	Low	Low	Low	Probably low	Probably low
Panjari 2016	Low	Probably low	Low	Probably high	Probably low	Probably low	Probably high
Papathoma 2016	Low	Probably low	Low	Low	Low	Probably low	Probably low
Pelak 2021	Low	Probably low	Probably high	Probably low	Low	Probably low	Probably high
Pele 2013	Low	Low	Probably high	High	Probably low	Probably low	High
PerezChacon 2024	Probably low	Probably low	Probably low	Probably high	Probably high	Probably low	Probably high
Perkin 2021	Probably high	Probably low	Probably low	Low	Low	Low	Probably high
Pesonen 2006	Probably high	Low	Probably low	Probably low	Probably low	Probably high	Probably high
Peters 2015	Low	Probably high	Probably low	Low	Probably low	Probably low	Probably high
Polaskey 2023	Probably high	Probably low	Probably low	Probably high	Probably low	Probably low	Probably high
Ponsonby 2022	Low	Probably low	Probably high	Low	Probably low	Low	Probably high
Pretorius 2024	Probably low	Probably low	Probably low	Probably low	Probably low	Probably low	Probably low
Pyrhonen 2011	Probably low	Probably low	Probably high	Probably low	Probably low	Probably low	Probably high

© 2026 Islam N et al. *JAMA Pediatrics*.

Pyrhonen 2013	Low	Probably low	Probably high	Low	Probably low	Probably low	Probably high
Pyrhonen 2020	Probably low	Probably low	NA	Low	NA	Low	Probably low
Roduit 2014	Low	Probably low	Probably low	Probably low	Low	Low	Probably low
Roduit 2017	Probably low	Probably low	Probably high	Probably low	Probably low	Low	Probably high
Rohrl 2021	Low	Probably low	Probably high	Probably high	Low	Probably low	Probably high
Rosas-Salazar 2022	Probably low	Probably high	Probably high	Probably low	Probably low	Probably high	Probably high
Ruohomaki 2021	Probably low	Probably low	Probably high	Probably low	Probably low	Probably low	Probably high
Saarinen 2000	Probably low	Probably low	Probably high	Probably low	Probably low	Probably low	Probably high
Sakihara 2016	Low	Low	Low	Probably high	Probably low	Low	Probably high
Sánchez-Valverde 2009	Probably high	Probably low	Probably high	Probably high	Probably low	Probably low	Probably high
Sato 2023	Probably low	Probably low	Probably low	Probably high	Probably high	Probably low	Probably high
Sausenthaler 2007	Low	Probably low	Probably high	Probably low	Probably low	Low	Probably high
Savage 2012	Probably low	Probably low	Probably high	Low	Probably high	Low	Probably high
Sbihi 2015	Probably low	Probably low	Probably low	Low	Probably low	Probably low	Probably low
Seay 2022	Probably low	Probably low	Low	Probably high	Probably low	Probably low	Probably high
Shek 2010	Low	Probably low	Probably high	Probably high	Probably low	Low	Probably high
Shibuya 2013	Probably low	Probably high	Low	Probably high	Probably low	Low	Probably high
Shoda 2016	Probably low	Probably high	Probably high	High	Probably low	Low	High
Sicherer 2010	Low	Probably low	Probably low	Low	Probably low	Low	Probably low
Sicherer 2019	Low	Low	Probably low	Low	Probably low	Low	Probably low
Sidorchuk 2004	Probably low	Low	Low	Low	Low	Low	Probably low
Simons 2020	Low	Probably low	Probably low	Low	Probably low	Low	Probably low
Smejda 2018	Probably low	Probably low	Probably high	High	Probably low	Probably low	High
Snijders 2008	Low	Low	Low	Low	Probably low	Probably low	Probably low
Soriano 2021	Probably low	Probably low	Probably high	Low	Probably low	Low	Probably high
Soriano 2023	Probably low	Probably high	Probably high	Probably high	Probably low	Probably low	Probably high
Stockholm 2017	Low	Low	Probably low	Probably low	Probably low	Probably high	Probably high
Suaini 2021	Probably low	Probably low	Probably low	Probably low	Probably low	Probably low	Probably low
Suaini 2024	Probably low	Low	Probably low	Probably low	Probably high	Probably low	Probably low
Sugiura 2021	Probably low	Probably low	Probably low	Probably low	Probably low	Low	Probably low
Sung 2022	Probably low	Probably low	Probably low	Probably high	Probably low	Probably high	Probably high
Tan 2012	Probably low	Low	Low	Low	Low	Probably low	Probably low
Tanaka 2015	Probably low	Probably low	Low	Low	Probably low	Probably high	Probably high

© 2026 Islam N et al. *JAMA Pediatrics*.

Tanaka 2021	Probably low	Probably low	Probably high	Probably high	Probably low	Probably high	Probably high
Tedner 2021	Probably low	Probably low	Probably low	Low	Probably low	Low	Probably low
Thacher 2016	Probably low	Probably low	Probably high	Probably high	Probably low	Probably low	Probably high
Thijs 2011	Probably low	Probably low	Low	Probably high	Probably low	Low	Probably high
Toelle 2020	Probably low	Probably high	Probably high	Low	Probably low	Probably high	Probably high
Tokinobu 2020	Probably low	Probably high	Probably high	Probably high	Probably low	Low	Probably high
Tuokkola 2016	Low	Probably low	Probably low	Probably high	Probably low	Low	Probably high
Tuokkola 2021	Low	Probably low	Probably low	Low	Probably low	Low	Probably low
Ushiyama 2002	Probably low	Probably low	Probably high	High	Probably low	Probably high	High
vandenBerg 2011	Probably low	Probably low	Probably high	Probably low	Probably low	Probably low	Probably high
vanNimwegen 2011	Probably low	Probably low	Probably high	Probably low	Probably low	Low	Probably high
Venero-Fernandez 2018	Probably low	Low	Low	Probably high	Probably low	Probably high	Probably high
Venkatamaran 2014	Probably low	Probably high	Probably low	Probably high	High	Probably high	High
Venter 2009	Probably low	Probably low	NA	Probably low	NA	Probably low	Probably low
Venter 2016	Probably low	Probably low	Probably low	Low	Probably low	Probably high	Probably high
Venter 2021	Low	Probably low	Low	Probably low	Probably low	Probably low	Probably low
Venter 2024	Probably low	Probably low	Probably high	Probably high	Probably low	Probably low	Probably high
Verhoeven 2023	Probably low	Probably low	Probably low	Low	Probably low	Probably low	Probably low
Vernon 2012	Low	Probably low	Low	Probably low	Probably low	Probably high	Probably high
Wang 2018	Probably low	Probably low	Probably low	Probably high	Probably low	Probably low	Probably high
Wen 2023	Probably low	Probably low	Probably high	Probably high	Probably high	Probably low	Probably high
West 2012	Probably low	Probably high	Probably low	Probably low	Probably low	Probably low	Probably high
Wetzig 2000	Probably low	Probably low	Probably high	High	Probably low	Probably high	High
Winters 2019	Probably high	Probably low	Probably high	Low	Probably low	Probably high	Probably high
Wright 2001	Probably low	Probably low	Probably high	Probably low	Probably low	Probably low	Probably high
Yang 2019	Probably low	Probably low	Probably high	Low	Probably low	Probably high	Probably high
Yuenyongviwat 2024	Probably high	Low	Probably high	Probably high	Probably low	Probably low	Probably high
Zeng 2015	Probably low	Probably low	Probably low	Probably low	Probably low	Probably low	Probably low
Zhou 2017	Low	Probably low	Probably high	Probably high	Probably low	Low	Probably high

© 2026 Islam N et al. *JAMA Pediatrics*.

eTable 3. GRADE evidence profile: incidence of food allergy diagnosed by food challenge.

GRADE Evidence Profile									
	Number of Studies	Sample Size	Risk of Bias	Inconsistency	Indirectness	Imprecision	Publication Bias	**Overall Rating**	Incidence (95% CI)
Incidence of Food Allergy	16	18,279	Not Serious	Not Serious	Serious	Not Serious	Not Serious	**Moderate**[a]	4.7% (3.2% to 6.9%)

[a]The overall certainty rated down due to indirectness. Not all participants of the included studies underwent food challenge.

© 2026 Islam N et al. *JAMA Pediatrics*.

eTable 4. GRADE evidence profile: risk factors of food allergy in children.

GRADE Evidence Profile										
**Risk Factors**	**No. of Studies**	**Sample Size**	**Risk of Bias**	**Inconsistency**	**Indirectness**	**Imprecision**	**Publication bias**	**Overall Rating**	**Relative effect, adjusted OR (95% CI)**	**Risk Difference (95% CI)**
**Onset of Atopic Dermatitis in Children**										
Within the first year of life vs no onset at this time	19	43,661	Not serious[a]	Not serious	Not serious	Not serious	Not serious	High	3.88 (3.03 to 4.97)	12.0% more (8.8% to 15.7% more)
Within the first 2-3 years of life vs no onset at this time	3	2,331	Serious[b]	Not serious	Not serious	Not serious	Not serious	Moderate	3.34 (1.67 to 6.70)	10.0% more (3.1% to 21.1% more)
**Site of Atopic Dermatitis in Children**										
Face vs no atopic dermatitis	2	398	Serious[b]	Serious[c]	Not serious	Very serious[d]	Not serious	Very Low	0.86 (0.18 to 4.24)	0.7% less (4.1% less to 13.2% more)
**Severity of Atopic Dermatitis in Children**										
Measured by SCORing Atopic Dermatitis (SCORAD; 0–103, higher scores indicate greater severity)	2	1,032	Not serious[e]	Not serious	Not serious	Serious[f]	Not serious	Moderate	1.22 (1.12 to 1.34)	1.0% more (0.6% to 1.6% more)
**Previous History of Atopic Condition in Children**										
Allergic rhinitis and/or conjunctivitis (yes vs no)	4	29,225	Not serious[a]	Not serious	Not serious	Not serious	Not serious	High	3.39 (2.51 to 4.58)	10.1% more (6.7% to 14.4% more)
Early life wheezing (yes vs no)	6	31,219	Not serious[a]	Not serious	Not serious	Not serious	Not serious	High	2.11 (1.46 to 3.04)	5.0% more (2.1% to 8.8% more)
**Presence of high Skin Transepidermal water loss (TEWL) vs low in children**	2	3,016	Serious[b]	Not serious	Not serious	Not serious	Not serious	Moderate	3.36 (2.41 to 4.68)	10.0% more (6.3% to 14.8% more)
**Metabolic biomarkers**										
Presence of high maternal folate vs low[h]	2	1,952	Not serious[e]	Not serious	Not serious	Serious[f]	Not serious	Moderate	0.63 (0.38 to 1.05)	1.8% less (3.0% less to 0.2% more)
Presence of low Vitamin D biomarkers vs low in children[i]	5	8,302	Serious[b]	Not serious	Not serious	Serious[f]	Not serious	Low	1.64 (1.01 to 2.66)	2.9% more (0.0% to 7.3% more)
**Genetic biomarker**										
Filaggrin gene (FLG) loss-of-function sequence variation (yes vs no)	5	8,478	Not serious[a]	Not serious	Not serious	Not serious	Not serious	High	1.93 (1.51 to 2.45)	4.2% more (2.4% to 6.4% more)
**Delayed introduction of food items in children**										

© 2026 Islam N et al. *JAMA Pediatrics*.

Delayed introduction of peanut (>12 months) vs early introduction (≤12 months)	5	71,121	Not serious[a]	Not serious	Not serious	Not serious	Not serious	High	2.55 (1.40 to 4.64)	6.8% more (1.9% to 14.6% more)
Delayed introduction of fish (>6 months) vs early introduction (≤6 months)	3	3,173	Not serious[e]	Not serious	Not serious	Serious[f]	Not serious	Moderate	1.53 (1.04 to 2.25)	2.5% more (0.2% to 5.6% more)
Delayed introduction of fruit (>3 months) vs early introduction (≤3 months)	3	2,604	Not serious[a]	Not serious	Not serious	Serious[f]	Not serious	Moderate	1.68 (0.98 to 2.87)	3.1% more (0.1% less to 8.1% more)
Delayed introduction of cow's milk formula (>3 months) vs early introduction (≤3 months)	3	1,973	Not serious[a]	Not serious	Not serious	Serious[f]	Not serious	Moderate	0.71 (0.50 to 1.00)	1.4% less (2.4% less to 0.0% more)
Delayed introduction of cow's milk (>1 month) vs early introduction (≤1 month)	2	1,506	Not serious[a]	Not serious	Not serious	Very serious[d]	Not serious	Low	1.17 (0.79 to 1.74)	0.8% more (1.0% less to 3.4% more)
Delayed introduction of egg (>6 months) vs early introduction (≤6 months)	8	8,109	Serious[g]	Not serious	Not serious	Very serious[d]	Not serious	Very Low	1.44 (0.80 to 2.60)	2.0% more (1.0% less to 7.0% more)
Delayed introduction of wheat (>6 months) vs early introduction (≤6 months)	2	1,488	Serious[b]	Serious[c]	Not serious	Very serious[d]	Not serious	Very Low	0.94 (0.39 to 2.30)	0.3% less (3.0% less to 5.8% more)
Delayed introduction of meat (>6 months) vs early introduction (≤6 months)	3	3,119	Not serious[a]	Serious[c]	Not serious	Very serious[d]	Not serious	Very Low	1.21 (0.49 to 2.96)	1.0% more (2.5% less to 8.5% more)
**Antibiotic use in children (systemic)**										
Within 1st month of life (yes vs no)	3	33,017	Not serious[a]	Not serious	Not serious	Serious[f]	Not serious	Moderate	4.11 (1.09 to 15.52)	12.8% more (0.4% to 40% more)
Within 1st year of life (yes vs no)	5	80,713	Not serious[a]	Not serious	Not serious	Serious[f]	Not serious	Moderate	1.39 (1.16 to 1.67)	1.8% more (0.8% to 3.1% more)
**Maternal use of antibiotics during pregnancy (yes vs no)**	9	52,990	Not serious[a]	Not serious	Not serious	Serious[f]	Not serious	Moderate	1.32 (1.12 to 1.54)	1.5% more (0.6% to 2.5% more)
**Male sex**	32	905,098	Not serious[a]	Not serious	Not serious	Serious[f]	Not serious	Moderate	1.24 (1.15 to 1.34)	1.1% more (0.7% to 1.6% more)
**Social History**										
First-born child (yes vs no)	13	127,327	Not serious[a]	Not serious	Not serious	Serious[f]	Not serious	Moderate	1.13 (1.06 to 1.20)	0.6% more (0.3% to 1.0% more)
Parental education (high vs low)±	7	69,027	Not serious[a]	Serious[c]	Not serious	Serious[f]	Not serious	Low	1.18 (0.91 to 1.53)	1.0% more (0.4% less to 2.5% more)
Childcare/day-care attendance (yes vs no)	6	46,262	Serious[g]	Not serious	Not serious	Very Serious[d]	Not serious	Very Low	0.93 (0.67 to 1.28)	0.3% less (1.6% less to 1.3% more)
**Family history of allergic disorders (e.g. asthma, atopic dermatitis, food allergy, allergic rhinitis and/or conjunctivitis) (yes vs no)**										

© 2026 Islam N et al. *JAMA Pediatrics*.

Mother only	7	16,356	Not serious[a]	Not serious	Not serious	Serious[f]	Not serious	Moderate	1.73 (1.20 to 2.49)	3.3 more (0.9% to 6.6% more)
Father only	3	6,112	Not serious[a]	Not serious	Not serious	Very Serious[d]	Not serious	Low	1.18 (0.70 to 1.99)	0.8% more (1.4% less to 4.5% more)
Both parents	9	7,577	Serious[b]	Not serious	Not serious	Not serious	Not serious	Moderate	2.22 (1.83 to 2.71)	5.5% more (3.8% to 7.5% more)
Siblings	3	4,826	Not serious[a]	Not serious	Not serious	Serious[f]	Not serious	Moderate	1.77 (0.83 to 3.77)	3.5% more (0.8% less to 11.6% more)
Family member not specified	2	2,139	Not serious[a]	Not serious	Not serious	Serious[f]	Not serious	Moderate	1.27 (0.98 to 1.65)	1.3% more (0.1% less to 3.0% more)
**Family History of Asthma (yes vs no)**										
Mother only	4	23,339	Serious[g]	Not serious	Not serious	Serious[f]	Not serious	Low	1.27 (0.87 to 1.85)	1.3% more (0.6% less to 3.8% more)
Father only	3	22,013	Not serious[a]	Not serious	Not serious	Serious[f]	Not serious	Moderate	1.18 (1.00 to 1.40)	1.0% more (0.0% to 1.9% more)
Both parents	2	3,441	Serious[b]	Not serious	Not serious	Serious[f]	Not serious	Low	2.10 (0.98 to 4.49)	5.0% more (0.1% less to 14.1% more)
Siblings	2	21,089	Not serious[e]	Not serious	Not serious	Serious[f]	Not serious	Moderate	1.14 (0.88 to 1.47)	5.0% more (0.1% less to 14.1% more)
**Family History of Atopic Dermatitis (yes vs no)**										
Mother only	5	24,986	Serious[g]	Not serious	Not serious	Serious[f]	Not serious	Low	1.62 (1.03 to 2.53)	2.9% more (0.1% to 6.8% more)
Father only	5	25,543	Not serious[a]	Not serious	Not serious	Serious[f]	Not serious	Moderate	1.32 (0.83 to 2.10)	1.5% more (0.8% less to 5.0% more)
Both parents	4	4,446	Not serious[a]	Not serious	Not serious	Not serious	Not serious	High	2.78 (1.76 to 4.39)	7.8% more (3.5% to 13.8% more)
Siblings	2	21,089	Not serious[e]	Not serious	Not serious	Serious[f]	Not serious	Moderate	0.84 (0.67 to 1.05)	1.0% less (1.6% less to 0.2% more)
**Family History of Food Allergy (yes vs no)**										
Mother only	8	29,587	Not serious[a]	Not serious	Not serious	Not serious	Not serious	High	1.98 (1.53 to 2.55)	4.4% more (2.5% to 6.8% more)
Father only	6	27,896	Serious[g]	Not serious	Not serious	Not serious	Not serious	Moderate	1.69 (1.28 to 2.23)	3.2% more (1.3% to 5.5% more)
Both parents	4	5,283	Serious[b]	Not serious	Not serious	Not serious	Not serious	Moderate	2.07 (1.26 to 3.41)	4.8% more (1.2% to 10.2% more)
Siblings	2	22,376	Not serious[a]	Not serious	Not serious	Not serious	Not serious	High	2.36 (1.97 to 2.82)	6.0% more (4.4% to 8.0% more)
Family member not specified	2	4,817	Not serious[e]	Not serious	Not serious	Very Serious[d]	Not serious	Low	1.40 (0.55 to 3.53)	1.9% more (2.2% less to 10.7% more)
**Family History of Allergic Rhinitis and/or Conjunctivitis (yes vs no)**										
Mother only	4	22,773	Serious[g]	Serious[c]	Not serious	Very serious[d]	Not serious	Very Low	1.23 (0.65 to 2.34)	1.1% more 1.7% less to 6.0% more)

© 2026 Islam N et al. *JAMA Pediatrics*.

Father only	5	26,279	Not serious[a]	Not serious	Not serious	Serious[f]	Not serious	Moderate	1.28 (1.01 to 1.64)	1.3% more (0.1% to 3.0% more)
Both parents	3	4,176	Serious[c]	Not serious	Not serious	Serious[f]	Not serious	Low	1.68 (1.23 to 2.30)	3.1% more (1.0% to 5.8% more)
Siblings	2	21,089	Not serious[e]	Not serious	Not serious	Very Serious[d]	Not serious	Low	1.25 (0.58 to 2.67)	1.2% more 2.0% less to 7.3% more)
**Migration history (yes vs no)**										
Children born and raised in the same country vs different country	5	73,127	Not serious[a]	Serious[c]	Not serious	Not serious	Not serious	Moderate	2.10 (1.49 to 2.97)	5.0% more (2.3% to 8.5% more)
Migration history in parents (yes vs no)	2	57,110	Serious[b]	Not serious	Not serious	Not serious	Not serious	Moderate	3.28 (2.09 to 5.15)	9.7% more (4.9% to 16.3% more)
**Reported Self-identification**										
Black vs White	3	1,387	Serious[b]	Not serious	Not serious	Not serious	Not serious	Moderate	3.93 (2.15 to 7.20)	12.1% more (5.2% to 22.5% more)
Black vs Non-Hispanic White	4	8,596	Serious[b]	Not serious	Not serious	Not serious	Not serious	Moderate	2.23 (1.65 to 3.02)	5.5% more (3.0% to 8.7% more)
**Birth related factors**										
Mode of delivery (any caesarean vs vaginal)	22	1,347,012	Not serious[a]	Not serious	Not serious	Serious[f]	Not serious	Moderate	1.16 (1.07 to 1.26)	1.0% more (0.3% to 1.2% more)
Season of birth (Winter vs Summer)	2	770	Not serious[a]	Not serious	Not serious	Very serious[d]	Not serious	Low	1.04 (0.66 to 1.65)	0.2% more (1.6% less to 3.0% more)
Season of birth (Spring vs Summer)	5	4,952	Not serious[a]	Not serious	Not serious	Very serious[d]	Not serious	Low	0.98 (0.72 to 1.34)	0.1% less (1.3% less to 1.6% more)
Season of birth (Autumn vs Summer)	2	770	Not serious[a]	Not serious	Not serious	Very serious[d]	Not serious	Low	0.80 (0.50 to 1.30)	1.0% less (2.4% less to 1.4% more)
Pre-term birth (yes vs no)	9	1,128,005	Not serious[a]	Not serious	Not serious	Serious[f]	Not serious	Moderate	0.66 (0.47 to 0.92)	1.6% less (2.6% to 0.4% less)
Post-term birth (yes vs no)	3	1,090,188	Not serious[a]	Not serious	Not serious	Not serious	Not serious	High	1.02 (0.95 to 1.09)	0.1% more (0.2% less to 0.4% more)
Birthweight (low vs normal)≠	7	1,139,051	Not serious[a]	Not serious	Not serious	Not serious	Not serious	High	0.99 (0.89 to 1.10)	0.0% more (0.5% less to 0.5% more)
Birthweight (high vs normal)±	3	74,811	Serious[g]	Serious[c]	Not serious	Very serious[d]	Not serious	Very Low	0.95 (0.39 to 2.33)	0.2% less (3.0% less to 5.9% more)
Maternal BMI (Underweight vs normal)#	2	2,452	Serious[b]	Not serious	Not serious	Very serious[d]	Not serious	Very Low	1.13 (0.68 to 1.88)	0.6% more (1.5% less to 4.0% more)
Maternal BMI (Obese vs normal)$	2	2,452	Serious[b]	Not serious	Not serious	Serious[f]	Not serious	Low	1.26 (0.82 to 1.93)	1.2% more (0.9% less to 4.2% more)
**Age**										
Children's age (per month increase)	6	3,632	Serious[g]	Not serious	Not serious	Serious	Not serious	Low	1.06 (1.01 to 1.12)	0.3% more (0.0% to 0.6% more)
Maternal age (per year increase)	5	5492	Not serious[a]	Not serious	Not serious	Serious	Not serious	Moderate	1.05 (0.99 to 1.11)	0.2% more (0.0% to 0.5% more)
**Patterns and duration of breastfeeding (BF)**										

© 2026 Islam N et al. *JAMA Pediatrics*.

Exclusive BF≥3 months	3	2,651	Serious[g]	Not serious	Not serious	Very serious[d]	Not serious	Very Low	1.13 (0.57 to 2.26)	0.6% more (2.1% less to 5.6% more)
Exclusive BF≥4 months	7	18,984	Not serious[a]	Not serious	Not serious	Very serious[d]	Not serious	Low	1.10 (0.82 to 1.47)	0.5% more (1.0% less to 2.2% more)
Exclusive BF≥5 months	13	24,146	Not serious[a]	Serious[f]	Not serious	Serious[f]	Not serious	Low	1.44 (1.03 to 2.00)	2.0% more (0.1% to 4.5% more)
Partial BF≥6 months	14	51,937	Not serious[a]	Not serious	Not serious	Serious[f]	Not serious	Moderate	1.12 (0.91 to 1.37)	0.6% more (0.4% less to 1.7% more)
History of BF (yes vs no)	5	15,166	Serious[g]	Serious[c]	Not serious	Very serious[d]	Not serious	Very Low	1.24 (0.59 to 2.63)	1.1% more (2.0% less to 7.2% more)
**Maternal diet during pregnancy**										
Cheese (yes vs no)	2	3,659	Serious[b]	Not serious	Not serious	Not serious	Not serious	Moderate	0.96 (0.80 to 1.16)	0.2% less (0.01% less to 0.8% more)
Egg (yes vs no)	5	11,589	Not serious[a]	Not serious	Not serious	Very serious[d]	Not serious	Low	0.91 (0.49 to 1.67)	0.4% less (2.5% less to 3.1% more)
Fish (yes vs no)	3	10,429	Serious[b]	Not serious	Not serious	Serious[f]	Not serious	Low	1.18 (0.94 to 1.49)	0.8% more (0.3% less to 2.3% more)
Iron supplements (yes vs no)	2	5,471	Serious[b]	Not serious	Not serious	Serious[f]	Not serious	Low	0.78 (0.27 to 2.20)	1.1% less (3.6% less to 5.4% more)
Nuts (yes vs no)	2	3,044	Serious[b]	Not serious	Not serious	Serious[f]	Not serious	Low	1.18 (0.84 to 1.66)	0.8% more (0.8% less to 3.0% more)
Peanut (yes vs no)	3	1,153	Not serious[a]	Serious[c]	Not serious	Very serious[d]	Not serious	Very Low	1.60 (0.40 to 6.36)	2.8% more (2.9% less to 20.1% more)
Vitamin C (yes vs no)	2	1,468	Serious[b]	Not serious	Not serious	Serious[f]	Not serious	Low	0.77 (0.52 to 1.13)	1.1% less (2.3% less to 0.6% more)
Citrus Fruits	2	3,659	Serious[b]	Not serious	Not serious	Very serious[d]	Not serious	Very Low	1.27 (0.75 to 2.17)	1.3% more (1.2% less to 5.3% more)
Folic acid	2	5,492	Not serious[a]	Serious[c]	Not serious	Very serious[d]	Not serious	Very Low	0.49 (0.02 to 15.74)	2.5% less (4.9% less to 40.3% more)
**Maternal smoking during pregnancy (yes vs no)**	10	21,289	Not serious[a]	Not serious	Not serious	Serious[f]	Not serious	Moderate	1.09 (0.93 to 1.26)	0.4% more (0.3% less to 1.2% more)
**Smoking history in both parents (yes vs no)**	3	7,960	Serious[b]	Serious[c]	Not serious	Very serious[d]	Not serious	Very Low	0.78 (0.41 to 1.52)	1.1% less (2.9% less to 2.4% more)
**Household income (high vs low)±**	5	10,785	Not serious[a]	Not serious	Not serious	Serious[b]	Not serious	Moderate	1.23 (0.96 to 1.57)	1.1% more (0.2% less to 2.6% more)
**Diphtheria-Tetanus-Pertussis (DTP) vaccination vs no DTP vaccination**	3	7,957	Serious[b]	Not serious	Not serious	Very serious[d]	Not serious	Very Low	1.32 (0.69 to 2.52)	1.5% more (1.5% less to 6.7% more)
**Presence of dog at home (yes vs no)**	4	7,976	Not serious[a]	Not serious	Not serious	Very serious[d]	Not serious	Low	0.76 (0.44 to 1.34)	1.2% less (2.7% less to 1.6% more)
**Presence of cat at home (yes vs no)**	3	6,153	Not serious[a]	Not serious	Not serious	Very serious[d]	Not serious	Low	1.18 (0.69 to 2.01)	0.8% more (1.5% less to 4.6% more)
**Presence of any pets at home (yes vs no)**	3	4,575	Not serious[a]	Not serious	Not serious	Very serious[d]	Not serious	Low	0.93 (0.58 to 1.47)	0.3% less (2.0% less to 2.2% more)
*Baseline risk 4.6%; ROB; Risk of bias										

© 2026 Islam N et al. *JAMA Pediatrics*.

±We accepted the definitions of "high" and "low" as reported in the included studies
≠We accepted the definitions of "low" and "normal" as reported in the included studies
#We accepted the definitions of "underweight" and "normal" as reported in the included studies
$We accepted the definitions of "obese" and "normal" as reported in the included studies
[a]Certainty not rated down on the basis of risk of bias. Subgroup analyses found no differences between studies at high risk vs low risk of bias.
[b]Certainty rated down on the basis of risk of bias. All the evidence is coming from studies at high or probably high risk of bias.
[c]Certainty rated down due to inconsistency. The point estimates are widely variable, and many confidence intervals do not overlap.
[d]Certainty rated down for imprecision. The 95% confidence interval (CI) associated with risk difference crosses the threshold of 1% on both sides.
[e]Certainty not rated down on the basis of risk of bias. All the evidence comes from studies with low or probably low risk of bias.
[f]Certainty rated down for imprecision. The 95% confidence interval (CI) associated with risk difference crosses the threshold of 1% on one side.
[g]Certainty rated down on the basis of risk of bias. Subgroup analyses found differences between studies at high risk vs low risk of bias.
[h]Presence of high maternal folate (≥74.5 nmol/L) vs low maternal folate (<32.6 nmol/L).
[i]Presence of low Vitamin D [<50 nmol/L (<20 ng/mL)] vs high Vitamin D [≥75 nmol/L (≥30 ng/mL)] biomarkers in children.

© 2026 Islam N et al. *JAMA Pediatrics*.

eTable 5. Predictive factors reported in only one study.

Prognostic Factor	Number of studies	Sample size	Relative effect, adjusted OR (95% CI)	Absolute effect, Risk difference (95% CI)	Overall Rating
**Maternal diet during pregnancy (yes vs no)**					
Alpha-carotene (during third trimester of pregnancy)[141]	1	1018	0.98 (0.76 to 1.26)	0.1% less (1.2% less to 1.2% more)	Very Low[a,b]
Alpha-Tocopherol (during third trimester of pregnancy)[141]	1	1018	0.62 (0.26 to 1.52)	1.8% less (3.7% less to 2.4% more)	Very Low[a,b]
Apples (during third trimester of pregnancy)[165]	1	2641	1.01 (0.70 to 1.46)	0.0% more (1.4% less to 2.1% more)	Very Low[a,b]
Bananas (during third trimester of pregnancy)[165]	1	2641	1.14 (0.76 to 1.72)	0.7% more (1.2% less to 3.3% more)	Very Low[a,b]
Beta-Carotene (during third trimester of pregnancy)[141]	1	1018	0.94 (0.67 to 1.32)	0.3% less (1.6% less to 1.5% more)	Very Low[a,b]
Butter (during third trimester of pregnancy)[165]	1	2641	0.93 (0.60 to 1.43)	0.3% less (1.9% less to 2.0% more)	Very Low[a,b]
Cabbage (during third trimester of pregnancy)[165]	1	2641	0.84 (0.58 to 1.22)	0.8% less (2.0% less to 1.0% more)	Very Low[a,b]
Caffeine (during 2nd and 3rd trimester of pregnancy)[187]	1	1522	1.34 (1.01 to 1.70)	1.6% more (0.0% to 3.2% more)	Low[a,c]
Celery (during third trimester of pregnancy)[165]	1	2641	1.85 (1.18 to 2.89)	3.9% more (0.8% to 8.2% more)	Low[a,c]
Copper (during third trimester of pregnancy)[141]	1	1018	1.21 (0.40 to 3.69)	1.0% more (2.9% less to 11.3% more)	Very Low[a,b]
Cream (during third trimester of pregnancy)[165]	1	2641	1.26 (0.87 to 1.83)	1.2% more (0.6% less to 3.8% more)	Low[a,c]
Deep-frying vegetable fat (during third trimester of pregnancy)[165]	1	2641	1.12 (0.79 to 1.58)	0.6% more (1.0% less to 2.7% more)	Very Low[a,b]
Exotic fruit (during third trimester of pregnancy)[165]	1	2641	0.84 (0.58 to 1.23)	0.8% less (2.0% less to 1.1% more)	Very Low[a,b]
Fermented milk products (during third trimester of pregnancy)[141]	1	1018	1.00 (0.83 to 1.21)	0.0% more (0.8% less to 1.0% more)	Low[a,c]
Low intake of fruit juice (during third trimester of pregnancy)[165]	1	2641	1.12 (0.76 to 1.65)	0.6% more (1.2% less to 3.0% more)	Very Low[a,b]
Gamma-Tocopherol (during third trimester of pregnancy)[141]	1	1018	0.99 (0.58 to 1.70)	0.0% more (2.0% less to 3.2% more)	Very Low[a,b]
Juices (during third trimester of pregnancy)[141]	1	1018	0.99 (0.90 to 1.08)	0.0% more (0.5% less to 0.6% more)	Moderate[a]
Malaceous fruits (during third trimester of pregnancy)[141]	1	1018	0.97 (0.84 to 1.13)	0.1% less (0.8% less to 1.3% more)	Low[a,c]
Margarine only (during third trimester of pregnancy)[165]	1	2641	0.80 (0.50 to 1.27)	1.0% less (2.4% less to 1.3% more)	Very Low[a,b]
Margarine and low-fat spreads (during third trimester of pregnancy)[141]	1	1018	0.95 (0.76 to 1.20)	0.2% less (1.2% less to 0.9% more)	Very Low[a,b]
Milk (during third trimester of pregnancy)[165]	1	2641	0.95 (0.66 to 1.37)	0.2% less (1.6% less to 1.7% more)	Very Low[a,b]
Omega-3 polyunsaturated fatty acid-1st quarter low intake (during third trimester of pregnancy)[141]	1	1018	0.91 (0.58 to 1.43)	0.4% less (2.0% less to 2.0% more)	Very Low[a,b]
Omega-3 polyunsaturated fatty acid-4th quarter high intake (during third trimester of pregnancy)[141]	1	1018	0.80 (0.49 to 1.31)	1.0% less (2.5% less to 1.5% more)	Very Low[a,b]

© 2026 Islam N et al. *JAMA Pediatrics*.

Omega-6 polyunsaturated fatty acids-1st quarter low maternal intake (during third trimester of pregnancy)[141]	1	1018	0.78 (0.49 to 1.25)	1.1% less (2.5% less to 1.2% more)	Very Low[a,b]
Omega-6 polyunsaturated fatty acids-4th quarter high maternal intake (during third trimester of pregnancy)[141]	1	1018	0.69 (0.42 to 1.13)	1.5% less (2.8% less to 0.6% more)	Low[a,c]
Raw carrots (during third trimester of pregnancy)[165]	1	2641	1.02 (0.69 to 1.49)	0.1% more (1.5% less to 2.3% more)	Very Low[a,b]
Raw sweet pepper (during third trimester of pregnancy)[165]	1	2641	1.16 (0.79 to 1.72)	0.8% more (1.0% less to 3.3% more)	Very Low[a,b]
Raw tomatoes (during third trimester of pregnancy)[165]	1	2641	0.74 (0.49 to 1.11)	1.3% less (2.5% less to 0.5% more)	Low[a,c]
Salad (during third trimester of pregnancy)[165]	1	2641	1.14 (0.76 to 1.72)	0.7% more (1.2% less to 3.3% more)	Very Low[a,b]
Seeds (during third trimester of pregnancy)[165]	1	2641	0.72 (0.47 to 1.12)	1.3% less (2.6% less to 0.6% more)	Low[a,c]
Shelfish (during third trimester of pregnancy)[165]	1	2641	1.62 (1.11 to 2.37)	2.9% more (0.5% to 6.1% more)	Low[a,c]
Spinach (during third trimester of pregnancy)[165]	1	2641	0.82 (0.58 to 1.17)	0.9% less (2.0% less to 0.8% more)	Low[a,c]
Strawberries (during third trimester of pregnancy)[165]	1	2641	0.90 (0.60 to 1.34)	0.5% less (1.9% less to 1.6% more)	Very Low[a,b]
Total fat (during third trimester of pregnancy)[141]	1	1018	0.68 (0.17 to 2.76)	1.5% less (4.1% less to 7.7% more)	Very Low[a,b]
Total polyunsaturated fatty acids-1st quarter low maternal intake (during third trimester of pregnancy)[141]	1	1018	1.03 (0.65 to 1.62)	0.1% more (1.7% less to 2.9% more)	Very Low[a,b]
Total polyunsaturated fatty acids-4th quarter high maternal intake (during third trimester of pregnancy)[141]	1	1018	0.81 (0.50 to 1.33)	0.9% less (2.4% less to 1.5% more)	Very Low[a,b]
Vegetable juice (during third trimester of pregnancy)[165]	1	2641	0.85 (0.56 to 1.31)	0.7% less (2.1% less to 1.5% more)	Very Low[a,b]
Vegetable oils (during third trimester of pregnancy)[165]	1	2641	0.91 (0.61 to 1.34)	0.4% less (1.9% less to 1.6% more)	Very Low[a,b]
Wheat (during third trimester of pregnancy)[141]	1	1018	1.20 (0.75 to 1.93)	0.9% more (1.2% less to 4.2% more)	Very Low[a,b]
Zinc (during third trimester of pregnancy)[141]	1	1018	0.92 (0.25 to 3.37)	0.4% less (3.7% less to 10.1% more)	Very Low[a,b]
Long-chain omega-3 polyunsaturated fatty acids supplementation (during 2nd and 3rd trimester of pregnancy)[21]	1	706	0.55 (0.25 to 1.41)	2.2% less (3.7% less to 1.9% more)	Low[b]
Vitamin D (yes vs no)[101]	1	4115	4.42 (1.00 to 19.55)	13.9% more (0.0% to 45.7% more)	Moderate[c]
Vitamin E (yes vs no)[50]	1	1377	0.97 (0.02 to 48.02)	0.1% less (4.9% less to 66.7% more)	Low[b]
**Maternal diet during early pregnancy (eighth month) was assessed by a 181-item semi-quantitative Food Frequency Questionnaire (FFQ) – per 1 unit increase**					
Energy[194]	1	4403	0.98 (0.88 to 1.09)	0.1% less (0.6% less to 0.4% more)	High

© 2026 Islam N et al. *JAMA Pediatrics*.

Fat[194]	1	4403	0.95 (0.85 to 1.07)	0.2% less (0.7% less to 0.3% more)	High
Protein[194]	1	4403	0.94 (0.84 to 1.04)	0.3% less (1.0% less to 0.2% more)	Moderate[c]
Carbohydrates[194]	1	4403	1.07 (0.95 to 1.20)	0.3% more (0.2% less to 1.0% more)	Moderate[c]
Vitamin A total[194]	1	4403	1.02 (0.93 to 1.12)	0.1% more (0.3% less to 0.6% more)	High
Vitamin A diet[194]	1	4403	1.02 (0.93 to 1.12)	0.1% more (0.3% less to 0.6% more)	High
Vitamin C total[194]	1	4403	1.06 (0.95 to 1.17)	0.3% more (0.2% less to 1.0% more)	Moderate[c]
Vitamin C diet[194]	1	4403	1.02 (0.91 to 1.14)	0.1% more (0.4% less to 0.7% more)	High
Vitamin E total[194]	1	4403	1.03 (0.98 to 1.08)	0.1% more (0.1% less to 0.4% more)	High
Vitamin E diet[194]	1	4403	1.06 (0.96 to 1.18)	0.3% more (0.2% less to 1.0% more)	Moderate[c]
β-Carotene total[194]	1	4403	1.10 (1.02 to 1.20)	0.5% more (0.1% to 1.0% more)	Moderate[c]
β-Carotene diet[194]	1	4403	1.10 (1.01 to 1.19)	0.5% more (0.0% to 1.0% more)	Moderate[c]
Retinol total[194]	1	4403	0.95 (0.85 to 1.06)	0.2% less (0.7% less to 0.3% more)	High
Retinol diet[194]	1	4403	0.95 (0.85 to 1.06)	0.2% less (0.7% less to 0.3% more)	High
Saturated fatty acids (SFA) total[98]	1	4921	0.96 (0.84 to 1.05)	0.2% less (0.8% less to 0.2% more)	High
Myristic acid 14:0, total[98]	1	4921	1.00 (0.85 to 1.06)	0.0% more (0.7% less to 0.3% more)	High
Palmitic acid 16:0, total[98]	1	4921	0.95 (0.84 to 1.06)	0.2% less (1.0% less to 0.3% more)	Moderate[c]
Stearic acid 18:0, total[98]	1	4921	0.94 (0.84 to 1.06)	0.3% less (1.0% less to 0.3% more)	Moderate[c]
Monounsaturated fatty acids (MUFA), total,[98]	1	4921	0.91 (0.85 to 1.07)	0.4% less (0.7% less to 0.3% more)	High
Sum of 18:1 isomer, total[98]	1	4921	0.91 (0.85 to 1.07)	0.4% less (0.7% less to 0.3% more)	High
Polyunsaturated fatty acids (PUFA), total[98]	1	4921	0.95 (0.90 to 1.13)	0.2% less (0.5% less to 0.6% more)	High
n-3 PUFA, food[98]	1	4921	0.92 (0.86 to 1.07)	0.4% less (0.7% less to 0.3% more)	High
n-3 PUFA, total[98]	1	4921	0.92 (0.86 to 1.08)	0.4% less (0.7% less to 0.4% more)	High
α-Linolenic acid (18:3n-3), total[98]	1	4921	0.93 (0.87 to 1.08)	0.3% less (0.6% less to 0.4% more)	High
Eicosapentaenoic acid (EPA) (20:5n-3), food[98]	1	4921	0.95 (0.85 to 1.06)	0.2% less (0.7% less to 0.3% more)	High
EPA (20:5n-3), total[98]	1	4921	0.95 (0.83 to 1.06)	0.2% less (1.0% less to 0.3% more)	Moderate[c]
Docosahexaenoic acid (DHA) (22:6n-3), food[98]	1	4921	0.94 (0.85 to 1.07)	0.3% less (0.7% less to 0.3% more)	High
DHA (22:6n-3), total[98]	1	4921	0.94 (0.84 to 1.07)	0.3% less (0.8% less to 0.3% more)	High
n-6 PUFA, total[98]	1	4921	0.97 (0.91 to 1.14)	0.1% less (0.4% less to 0.7% more)	High

© 2026 Islam N et al. *JAMA Pediatrics*.

Linoleic acid (18:2n-6), total[98]	1	4921	0.97 (0.91 to 1.14)	0.1% less (0.4% less to 0.7% more)	High
Arachidonicacid (20:4n-6), food[98]	1	4921	0.97 (0.96 to 1.18)	0.1% less (0.2% less to 1.0% more)	Moderate[c]
Arachidonicacid (20:4n-6), total[98]	1	4921	0.97 (0.96 to 1.18)	0.1% less (0.2% less to 1.0% more)	Moderate[c]
γ-Linolenic acid (18:3n-6), food[98]	1	4921	1.03 (0.89 to 1.11)	0.1% more (0.5% less to 0.5% more)	High
γ-Linolenic acid (18:3n-6), total[98]	1	4921	1.04 (0.91 to 1.12)	0.2% more (0.4% less to 0.6% more)	High
Conjugated linoleic acid (18:2n-6), total[98]	1	4921	1.03 (0.88 to 1.10)	0.1% more (0.6% less to 0.5% more)	High
Trans fatty acids, total[98]	1	4921	0.94 (0.86 to 1.07)	0.3% less (0.7% less to 0.3% more)	High
Ratios; n-6 PUFA: n-3PUFA, food[98]	1	4921	1.11 (0.97 to 1.18)	0.5% more (0.1% less to 1.0% more)	Moderate[c]
Ratios; n-6 PUFA: n-3PUFA, total[98]	1	4921	1.10 (0.97 to 1.18)	0.5% more (0.1% less to 1.0% more)	Moderate[c]
Ratios; Linoleicacid(18:2n-6): α-linolenicacid(18:3n-3)[98]	1	4921	1.09 (0.95 to 1.17)	0.4% more (0.2% less to 1.0% more)	Moderate[c]
**Maternal diet in late pregnancy (32–36 weeks gestation) measured by Australian validated semi-quantitative food frequency questionnaire (SQFFQ) - 1-unit change in the standardized food or food group intake[154]**					
Fibre-rich bread	1	639	1.04 (0.85 to 1.24)	0.2% more (0.7% less to 1.1% more)	Moderate[c]
White bread	1	639	1.14 (1.02 to 1.28)	0.7% more (0.1% to 1.3% more)	Moderate[c]
Cold cereal	1	639	1.06 (0.89 to 1.26)	0.3% more (0.5% less to 1.2% more)	Moderate[c]
Rice	1	639	1.03 (0.86 to 1.23)	0.1% more (0.7% less to 1.1% more)	Moderate[c]
Oats	1	639	0.79 (0.61 to 1.02)	1.0% less (1.9% less to 0.1% more)	Moderate[c]
Pasta	1	639	1.06 (0.92 to 1.22)	0.3% more (0.4% less to 1.0% more)	Moderate[c]
Potatoes	1	639	1.08 (0.91 to 1.29)	0.4% more (0.4% less to 1.4% more)	Moderate[c]
Chips	1	639	1.06 (0.92 to 1.22)	0.3% more (0.4% less to 1.0% more)	Moderate[c]
Avocados	1	639	1.13 (1.00 to 1.28)	0.6% more (0.0% to 1.3% more)	Moderate[c]
Onions	1	639	0.98 (0.79 to 1.21)	0.1% less (1.0% less to 1.0% more)	Low[b]
Mushrooms	1	639	0.94 (0.74 to 1.19)	0.3% less (1.3% less to 1.0% more)	Low[b]
Legumes	1	639	1.04 (0.88 to 1.23)	0.2% more (0.6% less to 1.1% more)	Moderate[c]
Chicken	1	639	1.02 (0.85 to 1.21)	0.1% more (0.7% less to 1.0% more)	Moderate[c]
Beef/veal/lamb	1	639	1.11 (0.96 to 1.29)	0.5% more (0.2% less to 1.4% more)	Moderate[c]
Processed meat	1	639	0.96 (0.82 to 1.13)	0.2% less (1.0% less to 0.6% more)	Moderate[c]

© 2026 Islam N et al. *JAMA Pediatrics*.

**Maternal use of medications during pregnancy**					
Maternal acid suppressant medication (ASM) use (proton pump inhibitors or H2 receptor antagonists) during pregnancy (yes vs no)[15]	1	921	2.33 (1.07 to 5.07)	5.9% more (0.3% to 16.1% more)	Low[a,c]
**Maternal diet during postnatal period (yes vs no)**					
Alpha-carotene (during the first 6 months)[142]	1	1018	0.77 (0.50 to 1.19)	1.1% less (2.4% less to 0.9% more)	Very Low[a,b]
Alpha-tocopherol (during the first 6 months)[142]	1	1018	0.76 (0.44 to 1.30)	1.2% less (2.7% less to 1.4% more)	Very Low[a,b]
Beta-carotene (during the first 6 months)[142]	1	1018	0.74 (0.47 to 1.16)	1.3% less (2.7% less to 0.8% more)	Low[a,c]
Copper (during the first 6 months)[142]	1	1018	1.20 (0.82 to 1.75)	0.9% more (0.9% less to 3.4% more)	Very Low[a,b]
Gamma-tocopherol (during the first 6 months)[142]	1	1018	1.25 (0.85 to 1.85)	1.2% more (0.7% less to 3.9% more)	Low[a,c]
Iron (during the first 6 months)[142]	1	1018	0.99 (0.73 to 1.34)	0.0% more (1.3% less to 1.6% more)	Very Low[a,b]
Selenium (during the first 6 months)[142]	1	1018	1.05 (0.68 to 1.62)	0.2% more (1.5% less to 2.9% more)	Very Low[a,b]
Zinc (during the first 6 months)[142]	1	1018	1.05 (0.73 to 1.51)	0.2% more (1.3% less to 2.4% more)	Very Low[a,b]
Margarine and low-fat spreads (during the first 6 months)[142]	1	1018	0.96 (0.70 to 1.31)	0.2% less (1.4% less to 1.5% more)	Very Low[a,b]
Nuts (during the first 12 months)[48]	1	403	1.50 (0.90 to 2.52)	2.3% more (0.5% less to 6.7% more)	Low[a,c]
Omega−3 fatty acids (during the first 6 months)[142]	1	1018	1.09 (0.71 to 1.68)	0.4% more (1.4% less to 3.1% more)	Very Low[a,b]
Omega−3 fatty acids from fish (during the first 6 months)[142]	1	1018	0.80 (0.53 to 1.19)	1.0% less (2.3% less to 0.9% more)	Very Low[a,b]
Omega−3 fatty acids from vegetables (during the first 6 months)[142]	1	1018	1.21 (0.81 to 1.81)	1.0% more (0.9% less to 3.7% more)	Very Low[a,b]
Omega−6 fatty acids (during the first 6 months)[142]	1	1018	0.92 (0.61 to 1.41)	0.4% less (1.9% less to 1.9% more)	Very Low[a,b]
Total polyunsaturated fatty acids (PUFA) (during the first 6 months)[142]	1	1018	0.96 (0.62 to 1.49)	0.2% less (1.8% less to 2.3% more)	Very Low[a,b]
Vitamin C (during the first 6 months)[142]	1	1018	1.11 (0.84 to 1.47)	0.5% more (0.8% less to 2.2% more)	Low[a,c]
**Patterns and duration of breastfeeding (BF)**					
Exclusive BF≥2 months vs <2 months[163]	1	225	0.24 (0.13 to 0.45)	3.8% less (4.3% to 2.7% less)	Low[a,d]
Partial BF≥4 months vs <4 months[215]	1	2761	1.34 (0.84 to 2.13)	1.6% more (0.8% less to 5.1% more)	Moderate[c]
**Delayed introduction of food items**					
Cabbage (>6 vs ≤6 months)[140]	1	1067	1.51 (0.94 to 2.42)	2.4% more (0.3% less to 6.3% more)	Low[a,c]
Bread (>9 vs ≤9 months)[33]	1	856	1.43 (0.71 to 2.85)	2.0% more (1.4% less to 8.0% more)	Low[b]
Chocolate (>12 vs ≤12 months)[33]	1	856	1.45 (0.70 to 2.94)	2.1% more (1.4% less to 8.4% more)	Low[b]

© 2026 Islam N et al. *JAMA Pediatrics*.

Vegetables or fruits (>6 vs ≤6 months)[33]	1	856	0.99 (0.44 to 2.22)	0.0% more (2.7% less to 5.5% more)	Low[b]
Vegetables (>6 vs ≤6 months)[82]	1	562	1.19 (0.60 to 2.38)	0.9% more (1.9% less to 6.1% more)	Very Low[a,b]
Rye (>6 vs ≤6 months)[140]	1	1067	2.30 (1.40 to 3.75)	5.8% more (1.9% to 11.5% more)	Moderate[a]
Cheese (>18 vs ≤18 months)[44]	1	931	0.32 (0.14 to 0.69)	3.3% less (4.3% to 1.5% less)	Low[a,d]
Shop milk (>12 vs ≤12 months)[33]	1	856	0.99 (0.43 to 2.27)	0.0% more (2.8% less to 5.7% more)	Low[b]
Cake (>9 vs ≤9 months)[33]	1	856	1.12 (0.54 to 2.33)	0.6% more (2.2% less to 5.9% more)	Low[b]
Margarine (>12 vs ≤12 months)[33]	1	856	1.15 (0.55 to 2.44)	0.7% more (2.2% less to 6.4% more)	Low[b]
Oats (>6 vs ≤6 months)[140]	1	1067	1.82 (0.99 to 3.37)	3.7% more (0.0% to 10.1% more)	Low[a,c]
Carrots (>4 vs ≤4 months)[140]	1	1067	1.66 (0.97 to 2.83)	3.0% more (0.1% less to 8.0% more)	Low[a,c]
Soya (>12 vs ≤12 months)[33]	1	856	0.52 (0.16 to 1.67)	2.3% less (4.2% less to 3.1% more)	Low[b]
Farm milk (>12 vs ≤12 months)[33]	1	856	0.68 (0.24 to 2.00)	1.5% less (3.8% less to 4.5% more)	Low[b]
Potato (>4 vs ≤4 months)[140]	1	778	2.56 (1.49 to 4.39)	6.9% more (2.3% to 13.8% more)	Low[a,d]
Yogurt (>12 vs ≤12 months)[33]	1	856	1.45 (0.62 to 3.23)	2.1% more (1.8% less to 9.5% more)	Low[b]
Butter (>12 vs ≤12 months)[33]	1	856	1.18 (0.60 to 2.33)	0.8% more (1.9% less to 5.9% more)	Low[b]
Vitamin drops in breastfed child (>6 vs ≤6 months)[133]	1	7574	1.02 (0.75 to 1.38)	0.1% more (1.2% less to 1.8% more)	Very Low[a,b]
Vitamin drops in non-breastfed child (>6 vs ≤6 months)[133]	1	7574	1.63 (1.21 to 2.20)	2.9% more (1.0% less to 5.4% more)	Low[a,c]
Complementary food (eggs, cow's milk (not processed in a formula), peanuts) introduction ≥4 vs <4 months[72]	1	234	1.10 (0.70 to 1.50)	0.5% more (1.4% less to 2.3% more)	Very Low[a,b]
**Children's diet**					
Egg consumption ≥2 times per week at 12 months of age vs <2 times[208]	1	1252	0.11 (0.01 to 0.88)	4.4% less (4.9% to 0.6% less)	Low[a,c]
Infant diet diversity score at 6 months > median vs infant diet diversity score ≤median±[202]	1	967	1.52 (0.33 to 7.00)	2.4% more (3.3% less to 21.9% more)	Very Low[a,b]
Infant diet diversity score at 12 months ≥ median vs infant diet diversity score <median±[202]	1	967	0.77 (0.36 to 1.65)	1.1% more (3.1% less to 3.0% more)	Very Low[a,b]
Infant Diet Certainty Index (DQI) score at 18 months – per 1 score increase€[182]	1	728	0.91 (0.84 to 0.98)	0.4% less (1.0% to 0.1% less)	Moderate[c]
**Vaccination in children (yes vs no)**					
BCG vaccine[63]	1	2184	1.11 (0.56 to 1.98)	0.5% more (2.1% less to 4.4% more)	Very Low[a,b]
Varicella vaccine[63]	1	2184	1.21 (0.35 to 3.81)	1.0% more (3.2% less to 11.7% more)	Very Low[a,b]
Haemophilus influenzae type b vaccine[63]	1	2184	1.06 (0.79 to 1.49)	0.3% more (1.0% less to 2.3% more)	Very Low[a,b]

© 2026 Islam N et al. *JAMA Pediatrics*.

Rubella vaccine[63]	1	2184	0.92 (0.51 to 1.65)	0.4% less (2.4% less to 3.0% more)	Very Low[a,b]
Pneumococci vaccine[63]	1	2184	0.69 (0.30 to 1.44)	1.5% less (3.4% less to 2.0% more)	Very Low[a,b]
Mumps vaccine[63]	1	2184	1.00 (0.56 to 1.75)	0.0% more (2.1% less to 3.4% more)	Very Low[a,b]
Measles vaccine[63]	1	2184	0.95 (0.56 to 1.59)	0.2% less (2.1% less to 2.7% more)	Very Low[a,b]
Polio vaccine[63]	1	2184	1.08 (0.69 to 1.65)	0.4% more (1.5% less to 3.0% more)	Very Low[a,b]
Diphtheria vaccine[63]	1	2184	2.11 (0.74 to 5.90)	5.0% more (1.3% less to 18.7% more)	Very Low[a,b]
Tetanus vaccine[63]	1	2184	1.95 (0.69 to 5.53)	4.3% more (1.5% less to 17.5% more)	Very Low[a,b]
Hepatitis B vaccine[63]	1	2184	1.06 (0.74 to 1.49)	0.3% more (1.3% less to 2.3% more)	Very Low[a,b]
Meningococci vaccine[63]	1	2184	0.92 (0.35 to 2.13)	0.4% less (3.2% less to 5.1% more)	Very Low[a,b]
Any whole-cellular pertussis vaccine (before age 4 months) vs acellular pertussis[148]	1	218,093	0.44 (0.24 to 0.82)	2.7% less (3.8% to 1.0% less)	Low[a,c]
**Presence of dog outside of the home only (yes vs no)[91]**	1	5276	0.72 (0.52 to 0.99)	1.3% less (2.3% less to 0.0% more)	Low[a,c]
**Presence of cat outside of the home only (yes vs no)[91]**	1	5276	0.93 (0.49 to 1.77)	0.3% less (2.5% less to 3.5% more)	Very Low[a,b]
**Social history**					
Children has a usual general physician (yes vs no)[88]	1	3,739	2.64 (1.16 to 6.03)	7.2% more (0.8% to 19.1% more)	Low[a,c]
Family history of farming (yes vs no)[32]	1	686	2.11 (1.33 to 3.34)	5.0% more (1.5% to 10.0% more)	Low[a,d]
Smoking - any members of household (yes vs no)[101]	1	4115	4.44 (1.06 to 17.01)	13.9% more (0.3% to 42.2% more)	Low[c,d]
**Birth related factors (yes vs no)**					
Preeclampsia[180]	1	4788	1.21 (1.05 to 1.39)	1.0% more (0.2% to 1.8% more)	Low[a,c]
Gestational diabetes[158]	1	729	1.68 (0.70 to 11.30)	3.1% more (1.4% less to 32.3% more)	Very Low[a,b]
Season of birth (Autumn vs Spring)[50]	1	1377	0.76 (0.09 to 5.95)	1.2% less (4.5% less to 18.8% more)	Low[b]
Season of birth (Winter vs Spring)[50]	1	1377	1.61 (0.28 to 9.32)	2.8% more (3.5% less to 27.9% more)	Low[b]
Type of ruptured fetal membrane during delivery: artificial ruptured membranes vs spontaneously ruptured membranes[80]	1	332	1.12 (0.59 to 2.13)	0.6% more (2.0% less to 5.1% more)	Very Low[a,b]
Duration of ruptured membranes: 45–155 min vs <45 min[80]	1	198	2.05 (0.90 to 4.69)	4.7% more (0.5% less to 14.8% more)	Low[a,c]
Duration of ruptured membranes: 156–343 min vs <45 min[80]	1	191	1.32 (0.53 to 3.30)	1.5% more (2.3% less to 9.8% more)	Very Low[a,b]
Duration of ruptured membranes: >344 min vs <45 min[80]	1	191	1.13 (0.44 to 2.87)	0.6% more (2.7% less to 8.1% more)	Very Low[a,b]
Duration of labor: 208–356 min vs <208 min[80]	1	198	1.19 (0.52 to 2.74)	0.9% more (2.3% less to 7.6% more)	Very Low[a,b]

© 2026 Islam N et al. *JAMA Pediatrics*.

Duration of labor: 356–590 min vs <208 min[80]	1	195	0.97 (0.41 to 2.30)	0.1% less (2.9% less to 5.8% more)	Very Low[a,b]
Duration of labor: >590 min vs <208 min[80]	1	197	1.33 (0.53 to 3.30)	1.5% more (2.3% less to 9.8% more)	Very Low[a,b]
Any use of oxytocin (augmentation or induction) during delivery: yes vs no[80]	1	388	1.09 (0.59 to 2.03)	0.4% more (2.0% less to 4.7% more)	Very Low[a,b]
Any use of prostaglandin (induction) before delivery: yes vs no[80]	1	388	1.12 (0.49 to 2.60)	0.6% more (2.5% less to 7.0% more)	Very Low[a,b]
Certainty of amniotic fluid: meconium-stained vs normal[80]	1	388	0.98 (0.43 to 2.24)	0.1% less (2.8% less to 5.5% more)	Very Low[a,b]
**Reported self-identification**					
Black vs Non-African American[73]	1	590	1.12 (0.58 to 2.17)	0.6% more (2.0% less to 5.3% more)	Very Low[a,b]
**History of children infection vs no history of children infection**					
Human herpesvirus (Cytomegalovirus (CMV) and Epstein–Barr virus (EBV)[174]	1	2581	1.10 (0.80 to 1.50)	0.5% more (1.0% less to 2.3% more)	Low[b]
**Having another food allergy in children (yes vs no)**					
Physician diagnosed[78]	1	325	2.56 (1.02 to 6.43)	6.9% more (0.1% to 20.3% more)	Low[b,d]
Suspected by the parents or diagnosed food allergy combined[78]	1	325	3.98 (1.62 to 9.80)	12.3% more (2.9% to 29.0% more)	Low[d,e]
**Biomarkers**					
Post-natal cord blood (<30 ng/mL vs ≥30 ng/mL)[110]	1	460	1.10 (0.71 to 1.70)	0.5% more (1.4% less to 3.2% more)	Very Low[a,b]
Cord blood plasma specific IgE (sIgE) for peanut (<11 ng/mL vs >11 ng/mL) [109]	1	649	1.16 (0.83 to 1.63)	0.8% more (0.8% less to 2.9% more)	Low[a,c]
Post-natal Human-milk-oligosaccharides (HMO) profile: Partial Least Squares Discriminant Analysis (PLS-DA) score (quintile 1: -1.69-0.63 vs quintile 5: 2.17-5.16)[131]	1	421	0.10 (0.03 to 0.34)	4.5% less (5.0% to 3.2% less)	Low[a,d]
Propyl paraben tertile 3[166]	1	859	1.21 (0.64 to 2.27)	1.0% more (1.7% less to 5.7% more)	Very Low[a,b]
Eicosatetraenoic acid (Quintile 1:0.01-0.06 vs Q5:0.10-0.27)[132]	1	1109	1.05 (0.62 to 1.89)	0.2% more (1.8% less to 4.0% more)	Very Low[a,b]
Breast milk vaccenic acid (VA) concentration (highest 0.57–1.66 (wt%) vs lowest 0.08–0.34 (wt%) quartiles))[190]	1	315	0.34 (0.10 to 1.17)	3.2% less (4.5% less to 0.8% more)	Low[a,c]
C11orf30 variant LRRC32 (yes vs no)[123]	1	902	1.34 (1.20 to 1.49)	1.6% more (0.9% to 2.3% more)	Low[a,c]
SPINK5 variant rs9325071 (yes vs no)[16]	1	533	2.95 (1.49 to 5.83)	8.4% more (2.3% to 18.5% more)	Low[a,d]
Mucosa-associated lymphoid tissue lymphoma translocation protein 1 (MALT1) variant rs57265082 in peanut allergy vs peanut avoidance group[211]	1	542	8.15 (3.79 to 17.52)	25.0% more (11.6% to 43.0% more)	Low[a,d]
Cord blood Thymus-and-activation-regulated chemokine (cTARC (x10^2pg/mL) - measured at birth[164]	1	263	1.14 (1.07 to 1.23)	0.7% more (0.3% to 1.1% more)	Low[a,c]

© 2026 Islam N et al. *JAMA Pediatrics*.

Microbiota-by-age Z score (MAZ score) – per score increase[58]	1	323	0.45 (0.33 to 0.61)	2.7% less (3.3% to 1.9% less)	Low[a,d]
Higher proportion of cord blood naïve regulatory T cells (nTreg) in infancy measured by flow cytometry[30]	1	675	0.64 (0.47 to 0.93)	1.7% less (2.6% to 0.3% less)	Very low[a,b]
**Metabolomic profiles during early childhood (measured within 1st year of life)**					
Dehydroepiandrosterone sulfate (DHEA-S)[68]	1	782	0.57 (0.44 to 0.75)	2.1% less (2.7% to 1.2% less)	Low[a,d]
Androsterone sulfate[68]	1	782	0.56 (0.40 to 0.77)	2.1% less (2.9% to 1.1% less)	Low[a,d]
Androstenediol (3a, 17a) monosulfate[68]	1	782	0.68 (0.53 to 0.87)	1.5% less (2.3% to 0.6% less)	Low[a,c]
Androstenediol (3b,17b) monosulfate[68]	1	782	0.58 (0.40 to 0.83)	2.0% less (2.9% to 0.8% less)	Low[a,c]
1-Oleoyl-2-arachidonoyl-GPI (18:1/20:4)[68]	1	782	0.68 (0.54 to 0.85)	1.5% less (2.2% to 0.7% less)	Low[a,c]
Pregnenediol sulfate[68]	1	782	0.44 (0.27 to 0.73)	2.7% less (3.6% to 1.3% less)	Low[a,d]
Pregnenolone sulfate[68]	1	782	0.48 (0.32 to 0.73)	2.5% less (3.3% to 1.3% less)	Low[a,d]
N,N,N-trimethyl-5-aminovalerate[68]	1	782	0.58 (0.41 to 0.82)	2.0% less (2.9% to 1.0% less)	Low[a,c]
5a-pregnan-3b, 20a-diol monosulfate[68]	1	782	0.46 (0.31 to 0.68)	2.6% less (3.4% to 1.5% less)	Low[a,d]
5a-pregnan-3b, 20b-diol monosulfate[68]	1	782	0.48 (0.31 to 0.74)	2.5% less (3.4% to 1.3% less)	Low[a,d]
5a-pregnan-3b, 20a-diol disulfate[68]	1	782	0.42 (0.25 to 0.72)	2.8% less (3.7% to 1.3% less)	Low[a,d]
Taurohyocholate[68]	1	782	1.95 (1.34 to 2.84)	4.3% more (1.6% to 8.0% more)	Low[a,d]
Taurocholate[68]	1	782	1.99 (1.27 to 3.14)	4.5% more (1.3% to 9.2% more)	Low[a,d]
Palmitoylcholine[68]	1	782	1.56 (1.18 to 2.06)	2.6% more (0.8% to 4.8% more)	Low[a,c]
Arachidonoylcholine[68]	1	782	1.54 (1.16 to 2.03)	2.5% more (0.8% to 4.7% more)	Low[a,c]
Arachidoylcarnitine (C20)[68]	1	782	2.42 (1.36 to 4.32)	6.3% more (1.7% to 13.5% more)	Low[a,d]
Lactosyl-N-palmitoyl-sphingosine (d18:1/16:0)[68]	1	782	9.18 (2.20 to 38.20)	27.6% more (5.4% to 61.8% more)	Low[a,d]
Uracil[68]	1	782	2.31 (1.34 to 3.96)	5.8% more (1.6% to 12.2% more)	Low[a,d]
N-stearoylserine[68]	1	782	2.05 (1.27 to 3.31)	4.7% more (1.3% to 9.8% more)	Low[a,d]
Androstenediol (3b, 17b) disulfate (1)[68]	1	782	0.60 (0.43 to 0.85)	1.9% less (2.8% to 0.7% less)	Low[a,c]
Fecal Bile acid/bilirubin module (age 3-6 months) - food allergy vs healthy controls[102]	1	614	0.26 (0.07 to 0.66)	3.7% less (4.6% to 1.6% less)	Low[a,d]
Fecal amino acids (including 16 of the 20 proteinogenic amino acids) and the	1	614	0.48 (0.21 to 0.96)	2.5% less (3.9% to 0.2% less)	Low[a,c]

© 2026 Islam N et al. *JAMA Pediatrics*.

sphingolipid 3-ketosphinganine (age 3-6 months) - food allergy vs healthy controls[102]					
Fecal diacylglycerolssphingolipid 3-ketosphinganine (age 3-6 months) - food allergy vs healthy controls[102]	1	614	2.53 (1.17 to 6.20)	6.8% more (0.8% to 19.6% more)	Low[a,c]
Fecal caffeine metabolites (age 3-6 months) - food allergy vs healthy controls[102]	1	614	2.92 (0.94 to 11.23)	8.3% more (0.3% less to 32.1% more)	Low[a,c]
**Exposure to Traffic-related air pollution surrogates in children**					
Nitrogen dioxide (NO_2) exposure (yes vs no)[167]	1	2482	1.27 (1.00 to 1.41)	1.3% more (0.0% to 1.9% more)	Moderate[c]
Nitrogen Dioxide (NO2-per billion (ppb)) exposure at 1 year - per IQR (3.2) increase[112]	1	4688	1.22 (0.96 to 1.55)	1.0% more (0.2% to 2.5% more)	Moderate[c]
Fine particulate matter ($PM_{2.5}$-µg/m) exposure at 1 year - per IQR (1.6) increase[112]	1	4688	1.07 (0.97 to 1.19)	0.3% more (0.1% to 1.0% more)	Moderate[c]
Ultrafine particles (UFP-particles/cm^3) exposure at birth address – per IQR (2267) increase[23]	1	2278	1.04 (0.94 to 1.16)	0.2% more (0.3% less to 1.0% more)	Low[a,c]
Ultrafine particles (UFP-particles/cm^3) exposure at current address – per IQR (NR) increase[23]	1	2278	1.03 (0.92 to 1.14)	0.1% more (0.4% less to 1.0% more)	Low[a,c]
**Site of Atopic Dermatitis (eczema)[76]**					
Any vs no AD	1	289	3.14 (0.38 to 25.91)	9.2% more (3.0% less to 52.7% more)	Very Low[a,b]
AD in Head vs no AD	1	289	1.79 (0.79 to 4.25)	3.6% more (1.0% less to 13.3% more)	Very Low[a,b]
AD in Trunk vs no AD	1	289	1.14 (0.38 to 3.45)	0.7% more (3.0% less to 10.4% more)	Very Low[a,b]
AD in Upper limbs vs no AD	1	289	1.52 (0.22 to 10.39)	2.4% more (3.9% less to 30.4% more)	Very Low[a,b]
AD in Lower limbs vs no AD	1	289	0.31 (0.04 to 2.21)	3.4% less (4.8% less to 5.4% more)	Very Low[a,b]
**Lesion of Facial Atopic Dermatitis (eczema)[76]**					
Erythema vs no Erythema	1	289	1.88 (0.67 to 5.30)	4.0% more (1.6% less to 16.8% more)	Very Low[a,b]
Xerosis vs no Xerosis	1	289	1.09 (0.46 to 2.60)	0.4% more (2.6% less to 7.0% more)	Very Low[a,b]
Exudation vs no Exudation	1	289	3.05 (1.25 to 7.43)	8.8% more (1.2% to 23.1% more)	Low[a,d]
Papule vs no Papule	1	289	1.41 (0.59 to 3.36)	1.9% more (2.0% less to 10.0% more)	Very Low[a,b]
**Phenotype of Atopic Dermatitis (eczema)[54]**					
Flexural AD (either around the eyes, neck, antecubital, and popliteal fossae or ankles) vs no AD	1	619	0.98 (0.20 to 4.91)	0.1% more (4.0% less to 15.5% more)	Very Low[a,b]
Non-flexural AD vs no AD	1	619	3.07 (0.92 to 10.28)	8.9% more (0.4% less to 30.1% more)	Low[a,c]
Both flexural and non-flexural AD vs no AD	1	619	2.55 (0.74 to 8.83)	6.8% more (1.3% less to 26.7% more)	Very Low[a,b]

© 2026 Islam N et al. *JAMA Pediatrics*.

**Severity of Atopic Dermatitis (eczema)**					
Baseline SCORAD score <20 vs no AD[54]	1	619	3.91 (1.70 to 9.00)	12.1% more (3.2% to 27.1% more)	Low[a,d]
Baseline SCORAD score ≥20 vs no AD[54]	1	619	25.60 (9.03 to 72.57)	52.4% more (27.2% to 74.3% more)	Low[a,d]
Baseline Investigator Global Assessment (IGA) score, per 1 point increase[152]	1	109	1.06 (1.34 to 2.25)	0.3% more (1.6% to 5.6% more)	Low[a,d]
**Moisturization frequency at 3 months[149]**					
Once a week vs never	1	1,011	1.33 (0.38 to 4.66)	1.5% more (3.0% less to 14.7% more)	Very Low[a,b]
2-4 times a week vs never	1	1,011	2.25 (0.72 to 7.18)	5.6% more (1.3% less to 22.4% more)	Very Low[a,b]
5-6 times a week vs never	1	1,011	1.84 (0.40 to 8.68)	3.8% more (3.0% less to 26.4% more)	Very Low[a,b]
Daily vs never	1	1,011	4.32 (1.49 to 12.82)	13.5% more (2.3% to 35.3% more)	Low[a,d]
More than daily vs never	1	1,011	12.75 (4.10 to 39.55)	35.2% more (12.7% to 62.5% more)	Low[a,d]
**Infant pacifier sanitization at 6 months[178]**					
No pacifier sanitization vs no pacifier use	1	787	1.74 (0.42 to 7.18)	3.4% more (2.8% less to 22.4% more)	Very Low[a,b]
Pacifier sanitization by own mouth vs no pacifier use	1	787	0.73 (0.12 to 4.24)	1.3% less (4.4% less to 13.2% more)	Very Low[a,b]
Pacifier sanitization by tap water vs no pacifier use	1	787	1.36 (0.37 to 4.98)	1.7% more (3.1% less to 15.8% more)	Very Low[a,b]
Pacifier sanitization by boiling water vs no pacifier use	1	787	1.01 (0.25 to 4.05)	0.0% more (3.7% less to 12.6% more)	Very Low[a,b]
Pacifier sanitization by antiseptic vs no pacifier use	1	787	4.83 (1.10 to 21.18)	15.3% more (0.5% to 47.7% more)	Low[a,c]
**Household peanut-protein exposure (g/week): Environmental[55]**					
0.1-3.75 vs 0.0	1	283	1.57 (0.5 to 4.6)	2.6% more (2.4% less to 14.5% more)	Very Low[a,b]
3.75-15 vs 0.0	1	283	2.77 (1.2 to 6.5)	7.7% more (0.9% to 20.5% more)	Low[a,c]
>15.0 vs 0.0	1	283	6.09 (2.7 to 13.8)	19.3% more (7.4% to 37.1% more)	Low[a,d]
**Household peanut-protein exposure (gm/week): During pregnancy[55]**					
0.1-3.75 vs 0.0	1	283	1.28 (0.7 to 2.3)	1.3% more (1.4% less to 5.8% more)	Very Low[a,b]
3.75-15 vs 0.0	1	283	1.55 (0.8 to 2.9)	2.5% more (1.0% less to 8.2% more)	Very Low[a,b]
>15.0 vs 0.0	1	283	1.19 (0.6 to 2.4)	0.9% more (1.9% less to 6.2% more)	Very Low[a,b]
**Household peanut-protein exposure (g/week): During lactation[55]**					
0.1-3.75 vs 0.0	1	283	0.98 (0.5 to 1.9)	0.1% less (2.4% less to 4.1% more)	Very Low[a,b]

© 2026 Islam N et al. *JAMA Pediatrics*.

3.75-15 vs 0.0	1	283	1.02 (0.5 to 1.9)	0.1% more (2.4% less to 4.1% more)	Very Low[a,b]
>15.0 vs 0.0	1	283	0.89 (0.4 to 2.2)	0.5% less (2.9% less to 5.4% more)	Very Low[a,b]
**Maternal stress during pregnancy[176]**	1	370	0.97 (0.93 to 1.02)	0.1% less (0.3% less to 0.1% more)	Low[a]
**Maternal depression**					
During pregnancy (yes vs no)[216]	1	1139	1.87 (1.33 to 2.62)	4.0% more (1.5% to 7.1% more)	Low[a,d]
During postnatal period (elevated postpartum depressive symptoms vs no elevated postpartum depressive symptoms)[160]	1	969	3.33 (1.72 to 6.42)	9.9% more (3.3% to 20.3% more)	Low[a,d]
**Infant gut microbiota at 1 year[17]**					
Ratio of Enterobacteriaceae/Bacteroidaceae relative abundance detected from stool (per quartile increase)	1	166	4.43 (1.72 to 11.44)	13.9% more (3.3% to 32.6% more)	Low[a,d]
Chao1 Richness (per quartile increase)	1	166	1.24 (0.73 to 2.11)	1.1% more (1.3% less to 5.0% more)	Very Low[a,b]

*Baseline risk 4.6%; ROB; Risk of bias
±Diet diversity (DD) is defined using 4 measures: the World Health Organization definition of minimum DD at 6 months, as food diversity and fruit and vegetable diversity at 3, 6, and 9 months, and as food allergen diversity at 3, 6, 9, and 12 months.
€The DQI comprises 7 components: total rice, bread, and alternatives; total fruit; total vegetables; total meat and alternatives; total milk and dairy products; whole grains; and foods high in sugar. Participants were scored for each component, and a raw DQI score was eventually derived by adding the individual scores. The score was then adjusted by standardizing to an energy intake of 845 kcal/d, based on average daily energy requirements for 1-y olds in Singapore. A higher DQI score correlates with better diet certainty, which is associated with higher consumption of several nutrients and food groups, but no standard cutoffs exist in literature (theoretical range: 0–65).
[a]Certainty rated down on the basis of risk of bias. All the evidence is coming from high or probably high risk of bias.
[b]Certainty rated down for imprecision. The 95% confidence interval (CI) associated with risk difference crosses the threshold of 1% on both sides
[c]Certainty rated down for imprecision. The 95% confidence interval (CI) associated with risk difference crosses the threshold of 1% on one side.
[d]Certainty rated down for imprecision. The ratio of 95% CI (upper limit/lower limit) is ≥2.
[e]Certainty rated down for indirectness. Not all participants underwent oral food challenge.

© 2026 Islam N et al. *JAMA Pediatrics*.

eTable 6. Subgroup analyses.

Predictive Factors	Subgroup Factor	Subgroup Categories	No. of Studies	Sample Size	Adjusted OR & 95% CI	Interaction p-value
**Male sex**	Risk of bias	High/Probably High	21	877,955	1.23 (1.13 to 1.33)	0.66
		Low/Probably Low	11	27,143	1.29 (1.06 to 1.56)	
	Definition of FA	OFC	9	8,039	1.18 (0.95 to 1.47)	0.64
		No OFC	23	897,059	1.25 (1.15 to 1.37)	
	Number of FA	Single	7	9,801	1.52 (1.23 to 1.88)	0.07
		Multiple	20	874,994	1.23 (1.10 to 1.36)	
	Year of Publication	Before 2015	7	13,956	1.32 (1.04 to 1.69)	0.52
		2015 & after	25	891,142	1.22 (1.13 to 1.32)	
**Onset of Atopic Dermatitis in children**						
Within the first year of life (yes vs no)	Risk of bias	High/Probably High	15	23,507	3.85 (2.75 to 5.40)	0.45
		Low/Probably Low	4	20,154	4.58 (3.42 to 6.12)	
	Definition of FA	OFC	7	9,178	3.11 (1.73 to 5.60)	0.34
		No OFC	12	34,483	4.29 (3.18 to 5.77)	
	Number of FA	Single	5	9,608	3.70 (1.51 to 9.07)	0.95
		Multiple	10	12,730	3.81 (2.65 to 5.49)	
	Year of Publication	Before 2015	5	3,548	3.88 (2.53 to 5.93)	0.97
		2015 & after	14	40,113	3.83 (2.86 to 5.14)	
Within the first 2-3 years of life (yes vs no)	Risk of bias	High/Probably High	3	2,331	3.34 (1.67 to 6.70)	NA
		Low/Probably Low	-	-	-	
	Definition of FA	OFC	-	-	-	NA
		No OFC	3	2,331	3.34 (1.67 to 6.70)	
	Number of FA	Single	-	-	-	NA

© 2026 Islam N et al. *JAMA Pediatrics*.

		Multiple	3	2,331	3.34 (1.67 to 6.70)	
	Year of Publication	Before 2015	1	324	1.70 (0.62 to 4.66)	0.13
		2015 & after	2	2,007	4.20 (2.33 to 7.54)	
**Site of Atopic Dermatitis in Children**						
AD in Face vs no AD	Risk of bias	High/Probably High	2	398	0.86 (0.18 to 4.24)	NA
		Low/Probably Low	-	-	-	
	Definition of FA	OFC	-	-	-	NA
		No OFC	2	398	0.86 (0.18 to 4.24)	
	Number of FA	Single	-	-	-	NA
		Multiple	2	398	0.86 (0.18 to 4.24)	
	Year of Publication	Before 2015	-	-	-	NA
		2015 & after	2	398	0.86 (0.18 to 4.24)	
**Severity of Atopic Dermatitis**						
SCORAD score increase	Risk of bias	High/Probably High	-	-	-	NA
		Low/Probably Low	2	1,032	1.22 (1.12 to 1.34)	
	Definition of FA	OFC	2	1,032	1.22 (1.12 to 1.34)	NA
		No OFC	-	-	-	
	Number of FA	Single	2	1,032	1.22 (1.12 to 1.34)	NA
		Multiple	-	-	-	
	Year of Publication	Before 2015	-	-	-	NA
		2015 & after	2	1,032	1.22 (1.12 to 1.34)	
**Social History**						
Parental education (high vs low)	Risk of bias	High/Probably High	6	67,850	1.16 (0.86 to 1.55)	0.71
		Low/Probably Low	1	1,177	1.36 (0.64 to 3.03)	
	Definition of FA	OFC	-	-	-	NA
		No OFC	7	69,027	1.18 (0.91 to 1.53)	
	Number of FA	Single	-	-	-	NA
		Multiple	4	66,069	1.24 (0.86 to 1.79)	

© 2026 Islam N et al. *JAMA Pediatrics*.

	Year of Publication	Before 2015	4	9,783	0.99 (0.79 to 1.25)	0.12
		2015 & after	3	59,244	1.36 (0.99 to 1.87)	
Childcare/day-care attendance (yes vs no)	Risk of bias	High/Probably High	4	44,187	1.10 (0.91 to 1.34)	0.03
		Low/Probably Low	2	2,075	0.63 (0.44 to 0.91)	
	Definition of FA	OFC	3	2,651	0.99 (0.56 to 1.75)	0.75
		No OFC	3	43,611	0.87 (0.51 to 1.48)	
	Number of FA	Single	1	1,419	0.31 (0.07 to 1.37)	0.18
		Multiple	3	2,685	0.90 (0.56 to 1.43)	
	Year of Publication	Before 2015	-	-	-	NA
		2015 & after	6	46,262	0.93 (0.67 to 1.28)	
Being the first-born (yes vs no)	Risk of bias	High/Probably High	8	104,984	1.09 (1.00 to 1.20)	0.43
		Low/Probably Low	5	22,343	1.22 (1.09 to 1.37)	
	Definition of FA	OFC	2	621	1.11 (0.78 to 1.59)	0.95
		No OFC	11	126,706	1.12 (1.05 to 1.22)	
	Number of FA	Single	-	-	-	NA
		Multiple	8	62,626	1.05 (0.95 to 1.18)	
	Year of Publication	Before 2015	1	686	0.91 (0.59 to 1.39)	0.31
		2015 & after	12	126,641	1.14 (1.06 to 1.20)	
**Family history of allergic disorders (asthma, atopic dermatitis, food allergy, allergic rhinitis and/or conjunctivitis) (yes vs no)**						
Mother only (yes vs no)	Risk of bias	High/Probably High	4	11,339	1.75 (1.30 to 2.35)	0.88
		Low/Probably Low	3	5,017	1.93 (0.54 to 6.85)	
	Definition of FA	OFC	3	6,512	1.74 (0.59 to 5.09)	0.95
		No OFC	4	9,844	1.67 (1.24 to 2.27)	
	Number of FA	Single	1	6,853	1.40 (1.00 to 1.97)	0.35
		Multiple	5	9,240	1.90 (1.11 to 3.26)	

© 2026 Islam N et al. *JAMA Pediatrics*.

	Year of Publication	Before 2015	1	2,595	2.74 (1.66 to 4.56)	0.07
		2015 & after	6	13,761	1.54 (1.06 to 2.24)	
Father only (yes vs no)	Risk of bias	High/Probably High	1	2,195	1.65 (0.89 to 3.07)	0.13
		Low/Probably Low	2	3,917	0.83 (0.45 to 1.57)	
	Definition of FA	OFC	3	6,112	1.18 (0.70 to 1.99)	NA
		No OFC	-	-	-	
	Number of FA	Single	-	-	-	NA
		Multiple	3	6,112	1.18 (0.70 to 1.99)	
	Year of Publication	Before 2015	1	2,195	1.65 (0.89 to 3.07)	0.13
		2015 & after	2	3,917	0.83 (0.45 to 1.57)	
Both parents (yes vs no)	Risk of bias	High/Probably High	9	7,577	2.22 (1.83 to 2.71)	NA
		Low/Probably Low	-	-	-	
	Definition of FA	OFC	5	3,968	2.03 (1.34 to 3.08)	0.47
		No OFC	4	3,609	2.44 (1.85 to 3.23)	
	Number of FA	Single	1	374	1.01 (0.44 to 2.32)	0.09
		Multiple	7	5,347	2.29 (1.86 to 2.83)	
	Year of Publication	Before 2015	2	3,870	3.24 (2.00 to 5.25)	0.10
		2015 & after	7	3,707	2.06 (1.66 to 2.56)	
Siblings (yes vs no)	Risk of bias	High/Probably High	1	909	1.53 (1.04 to 2.25)	0.49
		Low/Probably Low	2	3,917	3.82 (0.29 to 50.25)	
	Definition of FA	OFC	2	3,917	3.82 (0.29 to 50.25)	0.49
		No OFC	1	909	1.53 (1.04 to 2.25)	
	Number of FA	Single	-	-	-	NA
		Multiple	2	3,917	3.82 (0.29 to 50.25)	
	Year of Publication	Before 2015	-	-	-	NA

© 2026 Islam N et al. *JAMA Pediatrics*.

		2015 & after	3	4,826	1.77 (0.83 to 3.77)	
Family member not specified	Risk of bias	High/Probably High	1	686	1.10 (0.73 to 1.66)	0.38
		Low/Probably Low	1	1,453	1.40 (1.00 to 1.96)	
	Definition of FA	OFC	-	-	-	NA
		No OFC	2	2,139	1.27 (0.98 to 1.65)	
	Number of FA	Single	-	-	-	NA
		Multiple	1	1,453	1.40 (1.00 to 1.96)	
	Year of Publication	Before 2015	1	686	1.10 (0.73 to 1.66)	0.38
		2015 & after	1	1,453	1.40 (1.00 to 1.96)	
**Family history of Asthma (yes vs no)**						
Mother only (yes vs no)	Risk of bias	High/Probably High	1	3,249	2.16 (1.11 to 4.28)	0.04
		Low/Probably Low	3	20,090	1.02 (0.86 to 1.21)	
	Definition of FA	OFC	2	3,530	1.86 (1.09 to 3.18)	0.03
		No OFC	2	19,809	1.00 (0.84 to 1.20)	
	Number of FA	Single	1	281	1.46 (0.61 to 3.49)	0.82
		Multiple	2	4,509	1.65 (0.95 to 2.86)	
	Year of Publication	Before 2015	1	3,249	2.16 (1.11 to 4.28)	0.04
		2015 & after	3	20,090	1.02 (0.86 to 1.21)	
Father only (yes vs no)	Risk of bias	High/Probably High	1	3,183	0.89 (0.38 to 2.04)	0.55
		Low/Probably Low	2	18,830	1.17 (0.87 to 1.56)	
	Definition of FA	OFC	2	3,464	0.81 (0.43 to 1.53)	0.22
		No OFC	1	18,549	1.22 (1.02 to 1.45)	
	Number of FA	Single	1	281	0.72 (0.28 to 1.88)	0.74
		Multiple	1	3,183	0.89 (0.38 to 2.04)	
	Year of Publication	Before 2015	1	3,183	0.89 (0.38 to 2.04)	0.55
		2015 & after	2	18,830	1.17 (0.87 to 1.56)	

© 2026 Islam N et al. *JAMA Pediatrics*.

Both parents (yes vs no)	Risk of bias	High/Probably High	2	3,441	2.10 (0.98 to 4.49)	NA
		Low/Probably Low	-	-	-	
	Definition of FA	OFC	2	3,441	2.10 (0.98 to 4.49)	NA
		No OFC	-	-	-	
	Number of FA	Single	-	-	-	NA
		Multiple	2	3,441	2.10 (0.98 to 4.49)	
	Year of Publication	Before 2015	2	3,441	2.10 (0.98 to 4.49)	NA
		2015 & after	-	-	-	
Siblings (yes vs no)	Risk of bias	High/Probably High	-	-	-	NA
		Low/Probably Low	2	21,089	1.14 (0.88 to 1.47)	
	Definition of FA	OFC	1	2,540	1.65 (0.52 to 5.25)	0.52
		No OFC	1	18,549	1.12 (0.86 to 1.45)	
	Number of FA	Single	-	-	-	NA
		Multiple	1	2,540	1.65 (0.52 to 5.25)	
	Year of Publication	Before 2015	-	-	-	NA
		2015 & after	2	21,089	1.14 (0.88 to 1.47)	
**Family history of Atopic Dermatitis (yes vs no)**						
Mother only (yes vs no)	Risk of bias	High/Probably High	3	6,156	2.17 (1.29 to 3.65)	0.04
		Low/Probably Low	2	18,830	1.21 (0.96 to 1.52)	
	Definition of FA	OFC	3	6,146	1.78 (0.82 to 3.87)	0.39
		No OFC	2	18,840	1.26 (1.11 to 1.43)	
	Number of FA	Single	1	281	0.80 (0.35 to 1.79)	0.04
		Multiple	3	6,156	2.17 (1.29 to 3.65)	
	Year of Publication	Before 2015	2	3,361	2.43 (1.14 to 5.20)	0.08
		2015 & after	3	21,625	1.27 (1.02 to 1.58)	
Father only (yes vs no)	Risk of bias	High/Probably High	2	5,536	1.96 (1.19 to 3.22)	0.17

		Low/Probably Low	3	2,007	1.00 (0.45 to 2.25)	
	Definition of FA	OFC	3	5,817	1.18 (0.44 to 3.19)	0.80
		No OFC	2	19,726	1.34 (1.17 to 1.53)	
	Number of FA	Single	1	281	0.40 (0.17 to 0.94)	0.01
		Multiple	3	6,713	1.95 (1.24 to 3.07)	
	Year of Publication	Before 2015	2	3,918	2.22 (1.23 to 3.99)	0.09
		2015 & after	3	21,625	1.03 (0.54 to 1.98)	
Both parents (yes vs no)	Risk of bias	High/Probably High	3	3,987	2.46 (1.47 to 4.11)	0.31
		Low/Probably Low	1	459	4.33 (1.73 to 12.10)	
	Definition of FA	OFC	3	3,987	2.46 (1.47 to 4.11)	0.31
		No OFC	1	459	4.33 (1.73 to 12.10)	
	Number of FA	Single	1	1,036	1.39 (0.51 to 3.77)	0.13
		Multiple	3	3,410	3.34 (2.00 to 5.57)	
	Year of Publication	Before 2015	2	2,951	3.02 (1.65 to 5.51)	0.75
		2015 & after	2	1,495	2.47 (0.81 to 7.51)	
Siblings (yes vs no)	Risk of bias	High/Probably High	-	-	-	NA
		Low/Probably Low	2	21,089	0.84 (0.67 to 1.05)	
	Definition of FA	OFC	1	2,540	0.83 (0.38 to 1.79)	0.97
		No OFC	1	18,549	0.84 (0.67 to 1.06)	
	Number of FA	Single	-	-	-	NA
		Multiple	1	2,540	0.83 (0.38 to 1.79)	
	Year of Publication	Before 2015	-	-	-	NA
		2015 & after	2	21,089	0.84 (0.67 to 1.05)	
**Family history of Food Allergy (yes vs no)**						
Mother only (yes vs no)	Risk of bias	High/Probably High	5	9,580	2.20 (1.45 to 3.34)	0.37
		Low/Probably Low	3	20,007	1.78 (1.49 to 2.13)	

© 2026 Islam N et al. *JAMA Pediatrics*.

	Definition of FA	OFC	3	3,401	1.67 (0.87 to 3.19)	0.56
		No OFC	5	26,186	2.04 (1.67 to 2.51)	
	Number of FA	Single	2	1,527	1.98 (0.73 to 5.34)	0.76
		Multiple	4	8,124	2.01 (1.23 to 3.29)	
	Year of Publication	Before 2015	4	9,253	2.70 (2.04 to 3.58)	0.01
		2015 & after	4	20,334	1.62 (1.26 to 2.09)	
Father only (yes vs no)	Risk of bias	High/Probably High	3	7,889	2.15 (1.50 to 3.09)	0.03
		Low/Probably Low	3	20,007	1.34 (1.10 to 1.64)	
	Definition of FA	OFC	2	2,956	1.70 (0.97 to 2.96)	0.85
		No OFC	4	24,940	1.81 (1.20 to 2.74)	
	Number of FA	Single	1	281	1.26 (0.42 to 3.83)	0.36
		Multiple	3	7,679	2.16 (1.51 to 3.10)	
	Year of Publication	Before 2015	3	7,679	2.16 (1.51 to 3.10)	0.08
		2015 & after	3	20,217	1.34 (1.10 to 1.63)	
Both parents (yes vs no)	Risk of bias	High/Probably High	4	5283	2.07 (1.26 to 3.41)	NA
		Low/Probably Low	-	-	-	
	Definition of FA	OFC	3	3,920	2.10 (0.86 to 5.13)	0.81
		No OFC	1	1,363	1.86 (1.12 to 3.03)	
	Number of FA	Single	1	1,036	0.54 (0.07 to 4.05)	0.14
		Multiple	2	2,884	2.73 (1.28 to 5.79)	
	Year of Publication	Before 2015	3	4,247	2.20 (1.42 to 3.40)	0.19
		2015 & after	1	1,036	0.54 (0.07 to 4.05)	
Siblings (yes vs no)	Risk of bias	High/Probably High	1	3,827	2.00 (1.11 to 3.57)	0.56
		Low/Probably Low	1	18,549	2.40 (1.99 to 2.90)	
	Definition of FA	OFC	-	-	-	NA

© 2026 Islam N et al. *JAMA Pediatrics*.

		No OFC	2	22,376	2.36 (1.97 to 2.82)	
	Number of FA	Single	-	-	-	NA
		Multiple	1	3,827	2.00 (1.11 to 3.57)	
	Year of Publication	Before 2015	1	3,827	2.00 (1.11 to 3.57)	0.56
		2015 & after	1	18,549	2.40 (1.99 to 2.90)	
Family member not specified	Risk of bias	High/Probably High	-	-	-	NA
		Low/Probably Low	2	4,817	1.40 (0.55 to 3.53)	
	Definition of FA	OFC	2	4,817	1.40 (0.55 to 3.53)	NA
		No OFC	-	-	-	
	Number of FA	Single	1	702	0.90 (0.49 to 1.66)	0.06
		Multiple	1	4,115	2.32 (1.06 to 5.00)	
	Year of Publication	Before 2015	-	-	-	NA
		2015 & after	2	4,817	1.40 (0.55 to 3.53)	
**Family History of Allergic rhinitis and/or conjunctivitis**						
Mother only (yes vs no)	Risk of bias	High/Probably High	1	2,766	2.74 (1.66 to 4.56)	0.01
		Low/Probably Low	3	20,007	0.92 (0.62 to 1.36)	
	Definition of FA	OFC	2	3,047	1.34 (0.31 to 5.82)	0.76
		No OFC	2	19,726	1.04 (0.61 to 1.80)	
	Number of FA	Single	1	281	0.61 (0.27 to 1.35)	0.01
		Multiple	2	3,943	2.11 (1.19 to 3.74)	
	Year of Publication	Before 2015	2	3,943	2.11 (1.19 to 3.74)	0.01
		2015 & after	2	18,830	0.84 (0.74 to 0.96)	
Father only (yes vs no)	Risk of bias	High/Probably High	2	6,272	1.38 (1.07 to 1.76)	0.87
		Low/Probably Low	3	20,007	1.31 (0.79 to 2.17)	
	Definition of FA	OFC	2	2,876	1.95 (1.20 to 3.16)	0.03
		No OFC	3	23,403	1.11 (0.98 to 1.25)	

© 2026 Islam N et al. *JAMA Pediatrics*.

	Number of FA	Single	1	281	2.69 (1.14 to 6.35)	0.13
		Multiple	3	7,449	1.35 (1.07 to 1.71)	
	Year of Publication	Before 2015	3	7,449	1.35 (1.07 to 1.71)	0.80
		2015 & after	2	18,830	1.53 (0.63 to 3.72)	
Both parents (yes vs no)	Risk of bias	High/Probably High	3	4,176	1.68 (1.23 to 2.30)	NA
		Low/Probably Low	-	-	-	
	Definition of FA	OFC	2	2,633	1.96 (1.15 to 3.33)	0.48
		No OFC	1	1,543	1.55 (1.05 to 2.29)	
	Number of FA	Single	1	1,543	1.55 (1.05 to 2.29)	0.48
		Multiple	2	2,633	1.96 (1.15 to 3.33)	
	Year of Publication	Before 2015	2	2,633	1.96 (1.15 to 3.33)	0.48
		2015 & after	1	1,543	1.55 (1.05 to 2.29)	
Siblings (yes vs no)	Risk of bias	High/Probably High	-	-	-	NA
		Low/Probably Low	2	21,089	1.25 (0.58 to 2.67)	
	Definition of FA	OFC	1	2,540	1.96 (1.03 to 3.73)	0.03
		No OFC	1	18,549	0.89 (0.70 to 1.14)	
	Number of FA	Single	-	-	-	NA
		Multiple	1	2,540	1.96 (1.03 to 3.73)	
	Year of Publication	Before 2015	-	-	-	NA
		2015 & after	2	21,089	1.25 (0.58 to 2.67)	
**Birth related factors**						
Season of birth (Winter vs Summer)	Risk of bias	High/Probably High	1	158	1.12 (0.68 to 1.82)	0.43
		Low/Probably Low	1	612	0.64 (0.16 to 2.10)	
	Definition of FA	OFC	1	158	1.12 (0.68 to 1.82)	0.43
		No OFC	1	612	0.64 (0.16 to 2.10)	
	Number of FA	Single	-	-	-	NA

© 2026 Islam N et al. *JAMA Pediatrics*.

		Multiple	1	158	1.12 (0.68 to 1.82)	
	Year of Publication	Before 2015	-	-	-	NA
		2015 & after	2	770	1.04 (0.66 to 1.65)	
Season of birth (Spring vs Summer)	Risk of bias	High/Probably High	2	1,786	1.05 (0.74 to 1.48)	0.39
		Low/Probably Low	3	3,166	0.75 (0.37 to 1.50)	
	Definition of FA	OFC	2	1,535	1.16 (0.72 to 1.85)	0.36
		No OFC	3	3,417	0.87 (0.58 to 1.30)	
	Number of FA	Single	-	-	-	NA
		Multiple	4	4,340	0.98 (0.71 to 1.36)	
	Year of Publication	Before 2015	1	1,177	0.67 (0.24 to 1.90)	0.45
		2015 & after	4	3,775	1.02 (0.74 to 1.41)	
Season of birth (Autumn vs Summer)	Risk of bias	High/Probably High	1	158	0.77 (0.46 to 1.34)	0.71
		Low/Probably Low	1	612	0.98 (0.32 to 3.04)	
	Definition of FA	OFC	1	158	0.77 (0.46 to 1.34)	0.71
		No OFC	1	612	0.98 (0.32 to 3.04)	
	Number of FA	Single	-	-	-	NA
		Multiple	1	158	0.77 (0.46 to 1.34)	
	Year of Publication	Before 2015	-	-	-	NA
		2015 & after	2	770	0.80 (0.50 to 1.30)	
Birthweight (low vs normal)	Risk of bias	High/Probably High	4	1,120,135	0.98 (0.88 to 1.09)	0.38
		Low/Probably Low	3	18916	1.19 (0.78 to 1.80)	
	Definition of FA	OFC	1	374	1.58 (0.53 to 4.70)	0.39
		No OFC	6	1,138,677	0.99 (0.89 to 1.09)	
	Number of FA	Single	2	32,848	0.98 (0.87 to 1.09)	0.12
		Multiple	1	1100	3.59 (0.70 to 18.40)	

© 2026 Islam N et al. *JAMA Pediatrics*.

	Year of Publication	Before 2015	3	50,280	0.98 (0.88 to 1.09)	0.46
		2015 & after	4	1,088,771	1.15 (0.76 to 1.74)	
Mode of delivery (caesarean vs vaginal)	Risk of bias	High/Probably High	17	1,343,762	1.14 (1.05 to 1.24)	0.25
		Low/Probably Low	5	3,250	1.44 (0.98 to 2.11)	
	Definition of FA	OFC	6	11,285	1.24 (0.84 to 1.83)	0.60
		No OFC	16	1,335,727	1.11 (1.04 to 1.20)	
	Number of FA	Single	4	39,897	1.26 (0.76 to 2.07)	0.82
		Multiple	12	215,814	1.18 (1.01 to 1.38)	
	Year of Publication	Before 2015	7	38,492	1.19 (1.11 to 1.27)	0.42
		2015 & after	15	1,308,520	1.13 (1.02 to 1.26)	
Pre-term birth (yes vs no)	Risk of bias	High/Probably High	6	1,122,629	0.60 (0.38 to 0.95)	0.57
		Low/Probably Low	3	5,376	0.73 (0.44 to 1.21)	
	Definition of FA	OFC	2	532	0.28 (0.13 to 0.63)	0.03
		No OFC	7	1,127,473	0.72 (0.49 to 1.08)	
	Number of FA	Single	1	374	0.18 (0.04 to 0.80)	0.08
		Multiple	5	39,742	0.84 (0.63 to 1.11)	
	Year of Publication	Before 2015	3	5,593	0.88 (0.61 to 1.29)	0.23
		2015 & after	6	1,122,412	0.55 (0.34 to 0.88)	
Post-term birth (yes vs no)	Risk of bias	High/Probably High	1	1,086,378	1.01 (0.95 to 1.08)	0.26
		Low/Probably Low	2	3,810	1.22 (0.88 to 1.69)	
	Definition of FA	OFC	-	-	-	NA
		No OFC	3	1090188	1.02 (0.95 to 1.09)	
	Number of FA	Single	-	-	-	NA

© 2026 Islam N et al. *JAMA Pediatrics*.

		Multiple	1	3522	1.23 (0.87 to 1.74)	
	Year of Publication	Before 2015	1	288	1.18 (0.48 to 2.89)	0.75
		2015 & after	2	1,089,900	1.02 (0.95 to 1.09)	
Birthweight (high vs normal)	Risk of bias	High/Probably High	1	57,005	0.41 (0.20 to 0.84)	0.06
		Low/Probably Low	2	17,806	1.56 (0.46 to 5.27)	
	Definition of FA	OFC	-	-	-	NA
		No OFC	3	74,811	0.95 (0.39 to 2.33)	
	Number of FA	Single	-	-	-	NA
		Multiple	1	57,005	0.41 (0.20 to 0.84)	
	Year of Publication	Before 2015	2	17,806	1.56 (0.46 to 5.27)	0.06
		2015 & after	1	57,005	0.41 (0.20 to 0.84)	
Maternal BMI (underweight vs normal)	Risk of bias	High/Probably High	2	2,452	1.13 (0.68 to 1.88)	NA
		Low/Probably Low	-	-	-	
	Definition of FA	OFC	-	-	-	NA
		No OFC	2	2,452	1.13 (0.68 to 1.88)	
	Number of FA	Single	1	1543	0.99 (0.62 to 1.58)	NA
		Multiple	-	-	-	
	Year of Publication	Before 2015	-	-	-	NA
		2015 & after	2	2,452	1.13 (0.68 to 1.88)	
Maternal BMI (Obese vs normal)	Risk of bias	High/Probably High	2	2,452	1.26 (0.82 to1.93)	NA
		Low/Probably Low	-	-	-	
	Definition of FA	OFC	-	-	-	NA
		No OFC	2	2,452	1.26 (0.82 to1.93)	
	Number of FA	Single	1	1543	1.02 (0.47 to 2.23)	NA
		Multiple	-	-	-	

© 2026 Islam N et al. *JAMA Pediatrics*.

	Year of Publication	Before 2015	-	-	-	NA
		2015 & after	2	2,452	1.26 (0.82 to1.93)	
**Reported Self-identification**						
Black vs White	Risk of bias	High/Probably High	2	937	5.41 (3.15 to 9.30)	0.05
		Low/Probably Low	1	450	2.32 (1.20 to 4.51)	
	Definition of FA	OFC	2	937	5.41 (3.15 to 9.30)	0.05
		No OFC	1	450	2.32 (1.20 to 4.51)	
	Number of FA	Single	3	1,387	3.93 (2.15 to 7.20)	NA
		Multiple	-	-	-	
	Year of Publication	Before 2015	2	1,124	3.54 (1.57 to 7.94)	0.48
		2015 & after	1	263	5.79 (1.92 to 17.43)	
Black vs Non-Hispanic White	Risk of bias	High/Probably High	2	7,191	2.36 (1.40 to 3.97)	0.61
		Low/Probably Low	2	1,405	1.96 (1.22 to 3.15)	
	Definition of FA	OFC	-	-	-	NA
		No OFC	4	8,596	2.23 (1.65 to 3.02)	
	Number of FA	Single	-	-	-	NA
		Multiple	4	8,596	2.23 (1.65 to 3.02)	
	Year of Publication	Before 2015	2	7,191	2.36 (1.40 to 3.97)	0.61
		2015 & after	2	1,405	1.96 (1.22 to 3.15)	
**Previous history of atopic condition in children**						
Allergic rhinitis and/or conjunctivitis (yes vs no)	Risk of bias	High/Probably High	3	28,999	3.43 (2.51 to 4.68)	0.82
		Low/Probably Low	1	226	3.02 (1.04 to 8.77)	
	Definition of FA	OFC	1	226	3.02 (1.04 to 8.77)	0.82
		No OFC	3	28,999	3.43 (2.51 to 4.68)	
	Number of FA	Single	1	226	3.02 (1.04 to 8.77)	0.82
		Multiple	3	28,999	3.43 (2.51 to 4.68)	

© 2026 Islam N et al. *JAMA Pediatrics*.

	Year of Publication	Before 2015	2	27,752	3.10 (1.63 to 5.90)	0.89
		2015 & after	2	1,473	2.89 (1.43 to 5.88)	
Early life wheezing (yes vs no)	Risk of bias	High/Probably High	5	30,703	2.00 (1.29 to 3.10)	0.45
		Low/Probably Low	1	516	2.70 (1.40 to 5.00)	
	Definition of FA	OFC	1	374	1.52 (0.89 to 2.59)	0.23
		No OFC	5	30,845	2.28 (1.52 to 3.43)	
	Number of FA	Single	1	374	1.52 (0.89 to 2.59)	0.23
		Multiple	5	30,845	2.28 (1.52 to 3.43)	
	Year of Publication	Before 2015	2	27,752	3.16 (2.24 to 4.45)	0.01
		2015 & after	4	3,467	1.70 (1.20 to 2.39)	
**Presence of high Skin Transepidermal water loss (TEWL) vs low in children**	Risk of bias	High/Probably High	2	3,016	3.36 (2.41 to 4.68)	NA
		Low/Probably Low	-	-	-	
	Definition of FA	OFC	-	-	-	NA
		No OFC	2	3,016	3.36 (2.41 to 4.68)	
	Number of FA	Single	-	-	-	NA
		Multiple	2	3,016	3.36 (2.41 to 4.68)	
	Year of Publication	Before 2015	1	619	3.26 (1.61 to 6.61)	0.92
		2015 & after	1	2397	3.39 (2.32 to 4.91)	
**Metabolic biomarkers**						
Presence of high maternal folate vs low	Risk of bias	High/Probably High	-	-	-	NA
		Low/Probably Low	2	1,952	0.63 (0.38 to 1.05)	
	Definition of FA	OFC	1	1394	0.77 (0.37 to 1.61)	0.45
		No OFC	1	558	0.52 (0.25 to 1.07)	
	Number of FA	Single	-	-	-	NA
		Multiple	2	1,952	0.63 (0.38 to 1.05)	

© 2026 Islam N et al. *JAMA Pediatrics*.

	Year of Publication	Before 2015	-	-	-	NA
		2015 & after	2	1,952	0.63 (0.38 to 1.05)	
Presence of low Vitamin D biomarkers vs normal in children	Risk of bias	High/Probably High	5	8,302	1.64 (1.01 to 2.66)	NA
		Low/Probably Low	-	-	-	
	Definition of FA	OFC	-	-	-	NA
		No OFC	5	8,302	1.64 (1.01 to 2.66)	
	Number of FA	Single	-	-	-	NA
		Multiple	5	8,302	1.64 (1.01 to 2.66)	
	Year of Publication	Before 2015	4	8,170	1.26 (0.95 to 1.66)	0.01
		2015 & after	1	132	5.42 (1.92 to 15.30)	
**Genetic biomarker**						
Filaggrin gene (FLG) loss-of-function sequence variation (yes vs no)	Risk of bias	High/Probably High	3	6,289	1.24 (0.73 to 2.13)	0.08
		Low/Probably Low	2	2,189	2.15 (1.64 to 2.81)	
	Definition of FA	OFC	3	6,642	2.10 (1.61 to 2.73)	0.11
		No OFC	2	1,836	1.22 (0.66 to 2.23)	
	Number of FA	Single	-	-	-	NA
		Multiple	5	8478	1.93 (1.51 to 2.45)	
	Year of Publication	Before 2015	3	2,264	1.51 (0.89 to 2.58)	0.32
		2015 & after	2	6,214	2.05 (1.57 to 2.69)	
**Age**						
Children's age (per month increase)	Risk of bias	High/Probably High	3	2311	1.05 (1.00 to 1.10)	0.01
		Low/Probably Low	2	1032	1.30 (1.15 to 1.47)	
	Definition of FA	OFC	2	1032	1.30 (1.15 to 1.47)	0.01
		No OFC	3	2311	1.05 (1.00 to 1.10)	
	Number of FA	Single	2	1032	1.30 (1.15 to 1.47)	0.01
		Multiple	2	241	1.04 (1.03 to 1.05)	

© 2026 Islam N et al. *JAMA Pediatrics*.

	Year of Publication	Before 2015	1	2070	1.23 (1.07 to 1.41)	0.08
		2015 & after	4	1273	1.07 (1.01 to 1.15)	
Maternal age (per year increase)	Risk of bias	High/Probably High	4	5033	1.06 (0.98 to 1.13)	0.66
		Low/Probably Low	1	459	1.03 (0.95 to 1.12)	
	Definition of FA	OFC	-	-	-	NA
		No OFC	5	5492	1.05 (0.99 to 1.11)	
	Number of FA	Single	1	160	1.20 (1.09 to 1.32)	0.01
		Multiple	3	3262	1.02 (0.97 to 1.07)	
	Year of Publication	Before 2015	2	2361	1.05 (1.01 to 1.09)	0.82
		2015 & after	3	3131	1.06 (0.95 to 1.19)	
**Antibiotic use in children**						
Within 1st month of life (yes vs no)	Risk of bias	High/Probably High	2	32,791	3.91 (0.52 to 29.58)	0.72
		Low/Probably Low	1	226	6.17 (1.42 to 26.85)	
	Definition of FA	OFC	1	226	6.17 (1.42 to 26.85)	0.72
		No OFC	2	32,791	3.91 (0.52 to 29.58)	
	Number of FA	Single	2	32,700	2.61 (0.80 to 8.51)	0.14
		Multiple	1	317	14.20 (2.03 to 99.15)	
	Year of Publication	Before 2015	2	32,791	3.91 (0.52 to 29.58)	0.72
		2015 & after	1	226	6.17 (1.42 to 26.85)	
Within 1st year of life (yes vs no)	Risk of bias	High/Probably High	2	792,262	2.18 (0.55 to 8.57)	0.55
		Low/Probably Low	3	14,851	1.42 (1.22 to 1.66)	
	Definition of FA	OFC	-	-	-	NA
		No OFC	5	80,713	1.39 (1.16 to 1.67)	
	Number of FA	Single	-	-	-	NA
		Multiple	5	80,713	1.39 (1.16 to 1.67)	

© 2026 Islam N et al. *JAMA Pediatrics*.

	Year of Publication	Before 2015	1	4089	1.40 (1.13 to 1.74)	0.89
		2015 & after	4	80,3024	1.43 (1.11 to 1.86)	
**Maternal use of antibiotics during pregnancy (yes vs no)**	Risk of bias	High/Probably High	5	38,163	1.68 (1.13 to 2.49)	0.18
		Low/Probably Low	4	14,827	1.21 (0.93 to 1.53)	
	Definition of FA	OFC	-	-	-	NA
		No OFC	9	52,990	1.32 (1.12 to 1.54)	
	Number of FA	Single	2	34,017	1.38 (0.93 to 2.06)	0.65
		Multiple	5	7,110	1.58 (1.06 to 2.36)	
	Year of Publication	Before 2015	1	32,474	1.21 (1.14 to 1.28)	0.13
		2015 & after	8	20,516	1.46 (1.16 to 1.83)	
**Maternal diet during pregnancy (yes vs no)**						
Cheese	Risk of bias	High/Probably High	2	3659	0.96 (0.80 to 1.16)	NA
		Low/Probably Low	-	-	-	
	Definition of FA	OFC	-	-	-	NA
		No OFC	2	3659	0.96 (0.80 to 1.16)	
	Number of FA	Single	-	-	-	NA
		Multiple	2	3659	0.96 (0.80 to 1.16)	
	Year of Publication	Before 2015	2	3659	0.96 (0.80 to 1.16)	NA
		2015 & after	-	-	-	
Egg	Risk of bias	High/Probably High	3	4,062	0.83 (0.63 to 1.10)	0.99
		Low/Probably Low	2	7,527	0.84 (0.08 to 9.15)	
	Definition of FA	OFC	1	4,115	0.23 (0.06 to 0.89)	0.04
		No OFC	4	7,474	1.10 (0.61 to 2.00)	
	Number of FA	Single	1	403	0.56 (0.09 to 3.46)	0.60
		Multiple	4	11,186	0.94 (0.49 to 1.82)	

© 2026 Islam N et al. *JAMA Pediatrics*.

	Year of Publication	Before 2015	3	4,062	0.83 (0.63 to 1.10)	0.99
		2015 & after	2	7,527	0.84 (0.08 to 9.15)	
Fish	Risk of bias	High/Probably High	3	10,429	1.18 (0.94 to 1.49)	NA
		Low/Probably Low	-	-	-	-
	Definition of FA	OFC	1	6288	1.30 (0.93 to 1.81)	0.44
		No OFC	2	4,141	1.08 (0.79 to 1.49)	
	Number of FA	Single	1	6288	1.30 (0.93 to 1.81)	0.33
		Multiple	1	2,641	1.01 (0.69 to 1.48)	
	Year of Publication	Before 2015	2	4,141	1.08 (0.79 to 1.49)	0.44
		2015 & after	1	6288	1.30 (0.93 to 1.81)	
Iron	Risk of bias	High/Probably High	2	5471	0.78 (0.27 to 2.20)	NA
		Low/Probably Low	-	-	-	-
	Definition of FA	OFC	1	4453	0.50 (0.29 to 0.87)	0.08
		No OFC	1	1018	1.47 (0.51 to 4.23)	
	Number of FA	Single	-	-	-	NA
		Multiple	2	5471	0.78 (0.27 to 2.20)	
	Year of Publication	Before 2015	1	4453	0.50 (0.29 to 0.87)	0.08
		2015 & after	1	1018	1.47 (0.51 to 4.23)	
Nuts	Risk of bias	High/Probably High	2	3044	1.18 (0.84 to 1.66)	NA
		Low/Probably Low	-	-	-	
	Definition of FA	OFC	-	-	-	NA
		No OFC	2	3044	1.18 (0.84 to 1.66)	
	Number of FA	Single	1	403	1.36 (0.76 to 2.42)	0.56
		Multiple	1	2641	1.10 (0.72 to 1.68)	
	Year of Publication	Before 2015	2	3044	1.18 (0.84 to 1.66)	NA

© 2026 Islam N et al. *JAMA Pediatrics*.

		2015 & after	-	-	-	
Peanuts	Risk of bias	High/Probably High	1	403	4.22 (1.57 to 11.32)	0.25
		Low/Probably Low	2	750	0.95 (0.09 to 9.75)	
	Definition of FA	OFC	-	-	-	NA
		No OFC	3	1153	1.60 (0.40 to 6.36)	
	Number of FA	Single	3	1153	1.60 (0.40 to 6.36)	NA
		Multiple	-	-	-	
	Year of Publication	Before 2015	2	906	3.16 (2.01 to 4.98)	0.01
		2015 & after	1	247	0.27 (0.08 to 0.87)	
Vitamin C	Risk of bias	High/Probably High	2	1468	0.77 (0.52 to 1.13)	NA
		Low/Probably Low	-	-	-	
	Definition of FA	OFC	-	-	-	NA
		No OFC	2	1468	0.77 (0.52 to 1.13)	
	Number of FA	Single	-	-	-	NA
		Multiple	2	1468	0.77 (0.52 to 1.13)	
	Year of Publication	Before 2015	2	1468	0.77 (0.52 to 1.13)	NA
		2015 & after	-	-	-	-
Citrus Fruits	Risk of bias	High/Probably High	2	3659	1.27 (0.75 to 2.17)	NA
		Low/Probably Low	-	-	-	-
	Definition of FA	OFC	-	-	-	NA
		No OFC	2	3659	1.27 (0.75 to 2.17)	
	Number of FA	Single	-	-	-	NA
		Multiple	2	3659	1.27 (0.75 to 2.17)	
	Year of Publication	Before 2015	1	2641	1.73 (1.18 to 2.53)	0.01
		2015 & after	1	1018	1.00 (0.92 to 1.09)	

© 2026 Islam N et al. *JAMA Pediatrics*.

Folic acid	Risk of bias	High/Probably High	-	-	-	NA
		Low/Probably Low	2	5,492	0.49 (0.02 to 15.74)	
	Definition of FA	OFC	2	5,492	0.49 (0.02 to 15.74)	NA
		No OFC	-	-	-	
	Number of FA	Single	-	-	-	NA
		Multiple	2	5,492	0.49 (0.02 to 15.74)	
	Year of Publication	Before 2015	-	-	-	NA
		2015 & after	2	5,492	0.49 (0.02 to 15.74)	
**Parental smoking**						
Maternal smoking during pregnancy (yes vs no)	Risk of bias	High/Probably High	7	15,331	1.04 (0.85 to 1.27)	0.79
		Low/Probably Low	3	5,958	1.10 (0.80 to 1.50)	
	Definition of FA	OFC	2	5,527	0.93 (0.43 to 2.01)	0.80
		No OFC	8	15,762	1.09 (0.92 to 1.28)	
	Number of FA	Single	-	-	-	NA
		Multiple	7	13,513	0.89 (0.71 to 1.12)	
	Year of Publication	Before 2015	1	4,089	1.20 (0.92 to 1.47)	0.28
		2015 & after	9	17,200	1.01 (0.83 to 1.23)	
Smoking history in both parents (yes vs no)	Risk of bias	High/Probably High	3	7,960	0.78 (0.41 to 1.52)	NA
		Low/Probably Low	-	-	-	
	Definition of FA	OFC	-	-	-	NA
		No OFC	3	7,960	0.78 (0.41 to 1.52)	
	Number of FA	Single	-	-	-	NA
		Multiple	3	7,960	0.78 (0.41 to 1.52)	
	Year of Publication	Before 2015	2	4,644	0.55 (0.25 to 1.19)	0.04
		2015 & after	1	3316	1.32 (1.02 to 1.70)	
**Household income (high vs low)**	Risk of bias	High/Probably High	4	9,408	1.22 (0.96 to 1.57)	0.94

© 2026 Islam N et al. *JAMA Pediatrics*.

		Low/Probably Low	1	1,377	1.34 (0.11 to 15.34)	
	Definition of FA	OFC	2	1,535	1.31 (0.83 to 2.08)	0.78
		No OFC	3	9,250	1.21 (0.86 to 1.69)	
	Number of FA	Single	-	-	-	NA
		Multiple	4	9,422	1.13 (0.86 to 1.49)	
	Year of Publication	Before 2015	2	7,920	1.28 (0.81 to 2.01)	0.87
		2015 & after	3	2,865	1.24 (0.80 to 1.91)	
**Diphtheria-Tetanus-Pertussis (DTP) vaccination vs no DTP vaccination**	Risk of bias	High/Probably High	3	7,957	1.32 (0.69 to 2.52)	NA
		Low/Probably Low	-	-	-	
	Definition of FA	OFC	1	5276	0.77 (0.36 to 1.63)	0.09
		No OFC	2	2,681	1.76 (0.97 to 3.19)	
	Number of FA	Single	-	-	-	NA
		Multiple	3	7,957	1.32 (0.69 to 2.52)	
	Year of Publication	Before 2015	1	2,184	1.33 (0.59 to 2.99)	0.99
		2015 & after	2	5,773	1.34 (0.43 to 4.15)	
**Patterns and duration of BF**						
Exclusive BF ≥3 months	Risk of bias	High/Probably High	1	317	3.70 (1.60 to 8.40)	0.01
		Low/Probably Low	2	2,334	0.74 (0.51 to 1.07)	
	Definition of FA	OFC	-	-	-	NA
		No OFC	3	2,651	1.13 (0.57 to 2.26)	
	Number of FA	Single	-	-	-	NA
		Multiple	3	2,651	1.13 (0.57 to 2.26)	
	Year of Publication	Before 2015	2	1,198	1.46 (0.26 to 8.24)	0.61
		2015 & after	1	1,453	0.92 (0.64 to 1.32)	
Exclusive BF ≥4 months	Risk of bias	High/Probably High	2	13,083	0.85 (0.63 to 1.16)	0.10
		Low/Probably Low	5	5,901	1.37 (0.85 to 2.20)	

© 2026 Islam N et al. *JAMA Pediatrics*.

	Definition of FA	OFC	2	1,442	2.58 (1.16 to 5.73)	0.03
		No OFC	5	17,541	0.99 (0.78 to 1.27)	
	Number of FA	Single	1	11,720	0.91 (0.64 to 1.30)	0.02
		Multiple	4	2,111	1.68 (1.19 to 2.39)	
	Year of Publication	Before 2015	4	5,822	1.03 (0.71 to 1.48)	0.39
		2015 & after	3	13,162	1.57 (0.63 to 3.88)	
Exclusive BF ≥5 months	Risk of bias	High/Probably High	9	5,061	1.69 (1.14 to 2.50)	0.23
		Low/Probably Low	4	19,185	0.96 (0.41 to 2.24)	
	Definition of FA	OFC	3	9,085	1.50 (0.26 to 8.85)	0.97
		No OFC	10	15,161	1.45 (1.06 to 1.98)	
	Number of FA	Single	3	1,091	2.72 (0.62 to 11.92)	0.53
		Multiple	7	11,518	1.62 (0.80 to 3.26)	
	Year of Publication	Before 2015	3	1,953	1.98 (1.05 to 3.73)	0.35
		2015 & after	10	22,293	1.36 (0.86 to 2.17)	
Partial BF ≥6 months	Risk of bias	High/Probably High	6	9,428	1.05 (0.80 to 1.38)	0.48
		Low/Probably Low	8	42509	1.25 (0.83 to 1.88)	
	Definition of FA	OFC	1	6,209	3.90 (1.58 to 9.65)	0.01
		No OFC	13	45,728	1.05 (0.88 to 1.25)	
	Number of FA	Single	3	6,586	2.98 (1.20 to 7.39)	0.03
		Multiple	4	4,784	0.94 (0.59 to 1.51)	
	Year of Publication	Before 2015	6	14,563	1.09 (0.85 to 1.40)	0.81
		2015 & after	8	37,374	1.15 (0.78 to 1.70)	
History of BF (yes vs no)	Risk of bias	High/Probably High	4	14,655	1.63 (0.69 to 3.86)	0.02
		Low/Probably Low	1	511	0.36 (0.15 to 0.87)	
	Definition of FA	OFC	1	511	0.36 (0.15 to 0.87)	0.02

© 2026 Islam N et al. *JAMA Pediatrics*.

		No OFC	4	14,655	1.63 (0.69 to 3.86)	
	Number of FA	Single	1	511	0.36 (0.15 to 0.87)	0.01
		Multiple	3	6,370	2.23 (1.34 to 3.71)	
	Year of Publication	Before 2015	2	12,024	1.22 (0.29 to 5.17)	0.95
		2015 & after	3	3,142	1.28 (0.44 to 3.75)	
**Delayed introduction of food items in children**						
Delayed introduction of cow's milk formula (>3 months) vs early introduction (≤3 months)	Risk of bias	High/Probably High	1	789	0.80 (0.50 to 1.10)	0.47
		Low/Probably Low	2	1,184	0.61 (0.33 to 1.13)	
	Definition of FA	OFC	-	-	-	NA
		No OFC	3	1,973	0.71 (0.50 to 1.00)	
	Number of FA	Single	-	-	-	NA
		Multiple	3	1,973	0.71 (0.50 to 1.00)	
	Year of Publication	Before 2015	2	1,024	0.81 (0.57 to 1.16)	0.34
		2015 & after	1	949	0.46 (0.24 to 0.88)	
Delayed introduction of cow's milk (>1 month) vs early introduction (≤1 month)	Risk of bias	High/Probably High	1	1067	1.25 (0.77 to 2.03)	0.64
		Low/Probably Low	1	856	1.02 (0.51 to 2.04)	
	Definition of FA	OFC	-	-	-	NA
		No OFC	2	1,506	1.17 (0.79 to 1.74)	
	Number of FA	Single	-	-	-	NA
		Multiple	2	1,506	1.17 (0.79 to 1.74)	
	Year of Publication	Before 2015	2	1,506	1.17 (0.79 to 1.74)	NA
		2015 & after	-	-	-	
Delayed introduction of egg (>6 months) vs early introduction (≤6 months)	Risk of bias	High/Probably High	2	1,264	4.93 (2.08 to 11.68)	0.01
		Low/Probably Low	6	6,845	0.95 (0.61 to 1.48)	
	Definition of FA	OFC	1	105	3.14 (1.23 to 7.99)	0.12
		No OFC	7	8,004	1.29 (0.68 to 2.42)	

© 2026 Islam N et al. *JAMA Pediatrics*.

	Number of FA	Single	1	1,159	7.58 (3.08 to 18.61)	0.01
		Multiple	6	6,712	1.14 (0.67 to 1.94)	
	Year of Publication	Before 2015	2	1,720	1.27 (0.99 to 1.64)	0.73
		2015 & after	6	6,389	1.54 (0.54 to 4.45)	
Delayed introduction of peanut (>12 months) vs early introduction (≤12 months)	Risk of bias	High/Probably High	3	68,433	1.89 (0.98 to 3.65)	0.09
		Low/Probably Low	2	2,688	4.63 (2.02 to 10.30)	
	Definition of FA	OFC	3	925	2.50 (0.94 to 6.61)	0.76
		No OFC	2	70,196	3.02 (1.45 to 6.29)	
	Number of FA	Single	2	68,116	3.24 (0.82 to 12.80)	0.71
		Multiple	3	3,005	2.41 (1.11 to 5.23)	
	Year of Publication	Before 2015	-	-	-	NA
		2015 & after	5	71,121	2.55 (1.40 to 4.64)	
Delayed introduction of fish (>6 months) vs early introduction (≤6 months)	Risk of bias	High/Probably High	-	-	-	NA
		Low/Probably Low	3	3,173	1.53 (1.04 to 2.25)	
	Definition of FA	OFC	-	-	-	NA
		No OFC	3	3,173	1.53 (1.04 to 2.25)	
	Number of FA	Single	3	3,173	1.53 (1.04 to 2.25)	NA
		Multiple	-	-	-	
	Year of Publication	Before 2015	2	1,720	1.67 (1.11 to 2.51)	0.24
		2015 & after	1	1,453	0.90 (0.35 to 2.30)	
Delayed introduction of fruit (>3 months) vs early introduction (≤3 months)	Risk of bias	High/Probably High	2	1,151	2.22 (1.10 to 4.46)	0.11
		Low/Probably Low	1	1,453	1.16 (0.78 to 1.73)	
	Definition of FA	OFC	-	-	-	NA
		No OFC	3	2,604	1.68 (0.98 to 2.87)	
	Number of FA	Single	-	-	-	NA
		Multiple	3	2,604	1.68 (0.98 to 2.87)	

© 2026 Islam N et al. *JAMA Pediatrics*.

	Year of Publication	Before 2015	2	1,151	2.22 (1.10 to 4.46)	0.11
		2015 & after	1	1,453	1.16 (0.78 to 1.73)	
Delayed introduction of wheat (>6 months) vs early introduction (≤6 months)	Risk of bias	High/Probably High	2	1,488	0.94 (0.39 to 2.30)	NA
		Low/Probably Low	-	-	-	
	Definition of FA	OFC	-	-	-	
		No OFC	2	1,488	0.94 (0.39 to 2.30)	
	Number of FA	Single	2	1,488	0.94 (0.39 to 2.30)	NA
		Multiple	-	-	-	
	Year of Publication	Before 2015	2	1,488	0.94 (0.39 to 2.30)	NA
		2015 & after	-	-	-	
Delayed introduction of meat (>6 months) vs early introduction (≤6 months)	Risk of bias	High/Probably High	1	827	1.65 (1.07 to 2.55)	0.57
		Low/Probably Low	2	2,292	1.02 (0.21 to 5.02)	
	Definition of FA	OFC	-	-	-	NA
		No OFC	3	3,119	1.21 (0.49 to 2.96)	
	Number of FA	Single	-	-	-	NA
		Multiple	3	3,119	1.21 (0.49 to 2.96)	
	Year of Publication	Before 2015	2	1,666	1.79 (1.23 to 2.62)	0.01
		2015 & after	1	1,453	0.46 (0.24 to 0.90)	
**Migration history (yes vs no) in parents**						
Children born and raised in the same country vs different country	Risk of bias	High/Probably High	4	69,577	2.12 (1.25 to 3.59)	0.90
		Low/Probably Low	1	3550	2.04 (1.49 to 2.86)	
	Definition of FA	OFC	-	-	-	NA
		No OFC	5	73,127	2.10 (1.49 to 2.97)	
	Number of FA	Single	-	-	-	NA
		Multiple	5	73,127	2.10 (1.49 to 2.97)	
	Year of Publication	Before 2015	4	16,122	1.88 (1.29 to 2.75)	0.14

© 2026 Islam N et al. *JAMA Pediatrics*.

		2015 & after	1	57,005	3.03 (1.82 to 5.00)	
Migration history in parents (yes vs no)	Risk of bias	High/Probably High	2	57,110	3.28 (2.09 to 5.15)	NA
		Low/Probably Low	-	-	-	
	Definition of FA	OFC	1	5,276	4.24 (2.99 to 6.01)	0.02
		No OFC	1	57,005	2.67 (2.28 to 3.27)	
	Number of FA	Single	-	-	-	NA
		Multiple	2	57,110	3.28 (2.09 to 5.15)	
	Year of Publication	Before 2015	-	-	-	NA
		2015 & after	2	57,110	3.28 (2.09 to 5.15)	
**Pets at Home**						
Presence of dog at home (yes vs no)	Risk of bias	High/Probably High	3	7,460	0.63 (0.23 to 1.69)	0.39
		Low/Probably Low	1	516	1.00 (0.70 to 1.40)	
	Definition of FA	OFC	3	7,460	0.63 (0.23 to 1.69)	0.39
		No OFC	1	516	1.00 (0.70 to 1.40)	
	Number of FA	Single	1	1,933	1.30 (0.50 to 3.38)	0.26
		Multiple	3	6,043	0.66 (0.34 to 1.30)	
	Year of Publication	Before 2015	-	-	-	NA
		2015 & after	4	7,976	0.76 (0.44 to 1.34)	
Presence of cat at home (yes vs no)	Risk of bias	High/Probably High	2	5,637	1.51 (0.47 to 4.78)	0.52
		Low/Probably Low	1	516	1.00 (0.60 to 1.50)	
	Definition of FA	OFC	2	5,637	1.51 (0.47 to 4.78)	0.52
		No OFC	1	516	1.00 (0.60 to 1.50)	
	Number of FA	Single	-	-	-	NA
		Multiple	3	6,153	1.18 (0.69 to 2.01)	
	Year of Publication	Before 2015	-	-	-	NA
		2015 & after	3	6,153	1.18 (0.69 to 2.01)	

© 2026 Islam N et al. *JAMA Pediatrics*.

Presence of any pets at home (yes vs no)	Risk of bias	High/Probably High	2	3,475	1.13 (0.80 to 1.58)	0.05
		Low/Probably Low	1	1100	0.50 (0.24 to 1.04)	
	Definition of FA	OFC	-	-	-	NA
		No OFC	3	4,575	0.93 (0.58 to 1.47)	
	Number of FA	Single	-	-	-	NA
		Multiple	2	2,430	0.80 (0.34 to 1.89)	
	Year of Publication	Before 2015	-	-	-	NA
		2015 & after	3	4,575	0.93 (0.58 to 1.47)	

© 2026 Islam N et al. *JAMA Pediatrics*.

eTable 7. Credibility of association of significant subgroup analyses by using modified ICEMAN.

Risk Factor	1: Is the analysis of effect modification based on comparison within rather than between trials?	2: For within-trial comparisons, is the effect modification similar from trial to trial?	3: For between-trial comparisons, is the number of trials large?	4: Was the direction of effect modification correctly hypothesized a priori?	5: Does a test for interaction suggest that chance is an unlikely explanation of the apparent effect modification?	6: Did the authors test only a small number of effect modifiers or consider the number in their statistical analysis?	7: Did the authors use a random effects model?	8: If the effect modifier is a continuous variable, were arbitrary cut points avoided?	9 Optional: Are there any additional considerations that may increase or decrease credibility?	10: How would you rate the overall credibility of the proposed effect modification?
**By Risk of bias**										
Childcare/day-care attendance (yes vs no)	Completely between studies	NA	Rather small or unclear	Probably yes	Chance may not explain	Definitely yes	Definitely yes	NA	NA	Low Credibility
Family history of asthma-Mother only	Completely between studies	NA	Rather small or unclear	Probably yes	Chance a likely explanation or unclear	Definitely yes	Definitely yes	NA	NA	Low Credibility
Family history of atopic dermatitis-Mother only	Completely between studies	NA	Rather large	Probably yes	Chance a likely explanation or unclear	Definitely yes	Definitely yes	NA	NA	Low Credibility
Family history of food allergy-Father only	Completely between studies	NA	Rather small or unclear	Probably yes	Chance a likely explanation or unclear	Definitely yes	Definitely yes	NA	NA	Low Credibility
Family history of allergic rhinitis and/or conjunctivitis- Mother only	Completely between studies	NA	Rather small or unclear	Probably yes	Chance may not explain	Definitely yes	Definitely yes	NA	NA	Low Credibility
Children's age (per month increase)	Completely between studies	NA	Rather large	Definitely no	Chance may not explain	Definitely yes	Definitely yes	NA	NA	Low Credibility
History of BF (yes vs no)	Completely between studies	NA	Rather large	Probably yes	Chance a likely explanation or unclear	Definitely yes	Definitely yes	NA	NA	Low Credibility
Delayed introduction of egg (>3 months) vs early introduction (≤3 months)	Completely between studies	NA	Rather large	Probably yes	Chance may not explain	Definitely yes	Definitely yes	NA	NA	Low Credibility
**By definition of FA**										
Family history of asthma-Mother only	Completely between studies	NA	Rather small or unclear	Definitely no	Chance a likely explanation or unclear	Definitely yes	Definitely yes	NA	NA	Low Credibility
Family history of allergic	Completely between studies	NA	Rather large	Definitely no	Chance may not explain	Definitely yes	Definitely yes	NA	NA	Low Credibility

© 2026 Islam N et al. *JAMA Pediatrics*.

rhinitis and/or conjunctivitis -Father only										
Family history of allergic rhinitis and/or conjunctivitis -Siblings	Completely between studies	NA	Very small	Definitely no	Chance may not explain	Definitely yes	Definitely yes	NA	NA	Low Credibility
Birth-related factors – Pre-term birth (yes vs no)	Completely between studies	NA	Rather large	Definitely no	Chance a likely explanation or unclear	Definitely yes	Definitely yes	NA	NA	Low Credibility
Children's age (per month increase)	Completely between studies	NA	Rather large	Definitely no	Chance may not explain	Definitely yes	Definitely yes	NA	NA	Low Credibility
Maternal consumption of egg during pregnancy	Completely between studies	NA	Rather large	Probably yes	Chance a likely explanation or unclear	Definitely yes	Definitely yes	NA	NA	Low Credibility
Exclusive BF ≥4 months	Completely between studies	NA	Rather large	Definitely no	Chance a likely explanation or unclear	Definitely yes	Definitely yes	NA	NA	Low Credibility
Partial BF ≥6 months	Completely between studies	NA	Rather large	Definitely no	Chance may not explain	Definitely yes	Definitely yes	NA	NA	Low Credibility
History of BF (yes vs no)	Completely between studies	NA	Rather small or unclear	Probably yes	Chance may not explain	Definitely yes	Definitely yes	NA	NA	Low Credibility
Migration history in parents (yes vs no)	Completely between studies	NA	Very small	Definitely no	Chance a likely explanation or unclear	Definitely yes	Definitely yes	NA	NA	Low Credibility
**By year of publication**										
Family history of asthma-Mother only	Completely between studies	NA	Rather small or unclear	Probably yes	Chance a likely explanation or unclear	Definitely yes	Definitely yes	NA	NA	Low Credibility
Family history of food allergy-Mother only	Completely between studies	NA	Rather small or unclear	Probably yes	Chance may not explain	Definitely yes	Definitely yes	NA	NA	Low Credibility
Family history of allergic rhinitis and/or conjunctivitis- Mother only	Completely between studies	NA	Rather small or unclear	Probably yes	Chance may not explain	Definitely yes	Definitely yes	NA	NA	Low Credibility
Ssmoking during pregnancy both parents	Completely between studies	NA	Rather small or unclear	Definitely no	Chance a likely explanation or unclear	Definitely yes	Definitely yes	NA	NA	Low Credibility
Early life wheezing in children (yes vs no)	Completely between studies	NA	Rather large	Probably yes	Chance may not explain	Definitely yes	Definitely yes	NA	NA	Low Credibility
Presence of low Vitamin D biomarkers vs normal in children	Completely between studies	NA	Rather large	Definitely no	Chance may not explain	Definitely yes	Definitely yes	NA	NA	Low Credibility

© 2026 Islam N et al. *JAMA Pediatrics*.

Maternal consumption of peanut during pregnancy	Completely between studies	NA	Rather small or unclear	Probably yes	Chance may not explain	Definitely yes	Definitely yes	NA	NA	Low Credibility
Maternal consumption of citrus fruits during pregnancy	Completely between studies	NA	Rather small or unclear	Probably yes	Chance may not explain	Definitely yes	Definitely yes	NA	NA	Low Credibility
Delayed introduction of meat (>6 months) vs early introduction (≤6 months)	Completely between studies	NA	Rather small or unclear	Probably yes	Chance may not explain	Definitely yes	Definitely yes	NA	NA	Low Credibility
**By number of FA (single vs multiple)**										
Family history of atopic dermatitis-Mother only	Completely between studies	NA	Rather small or unclear	Probably yes	Chance a likely explanation or unclear	Definitely yes	Definitely yes	NA	NA	Low Credibility
Family history of atopic dermatitis-Father only	Completely between studies	NA	Rather small or unclear	Probably yes	Chance may not explain	Definitely yes	Definitely yes	NA	NA	Low Credibility
Family history of allergic rhinitis and/or conjunctivitis- Mother only	Completely between studies	NA	Rather small or unclear	Probably yes	Chance may not explain	Definitely yes	Definitely yes	NA	NA	Low Credibility
Children's age (per month increase)	Completely between studies	NA	Rather large	Definitely no	Chance may not explain	Definitely yes	Definitely yes	NA	NA	Low Credibility
Maternal age (per year increase)	Completely between studies	NA	Rather small or unclear	Definitely no	Chance may not explain	Definitely yes	Definitely yes	NA	NA	Low Credibility
Exclusive BF ≥4 months	Completely between studies	NA	Rather large	Probably yes	Chance a likely explanation or unclear	Definitely yes	Definitely yes	NA	NA	Low Credibility
Partial BF ≥6 months	Completely between studies	NA	Rather large	Definitely no	Chance a likely explanation or unclear	Definitely yes	Definitely yes	NA	NA	Low Credibility
History of BF (yes vs no)	Completely between studies	NA	Rather small or unclear	Probably yes	Chance may not explain	Definitely yes	Definitely yes			Low Credibility
Delayed introduction of egg (>3 months) vs early introduction (≤3 months)	Completely between studies	NA	Rather large	Probably yes	Chance may not explain	Definitely yes	Definitely yes	NA	NA	Low Credibility

*ICEMAN (Instrument for assessing the Credibility of Effect Modification Analysis)

© 2026 Islam N et al. *JAMA Pediatrics*.

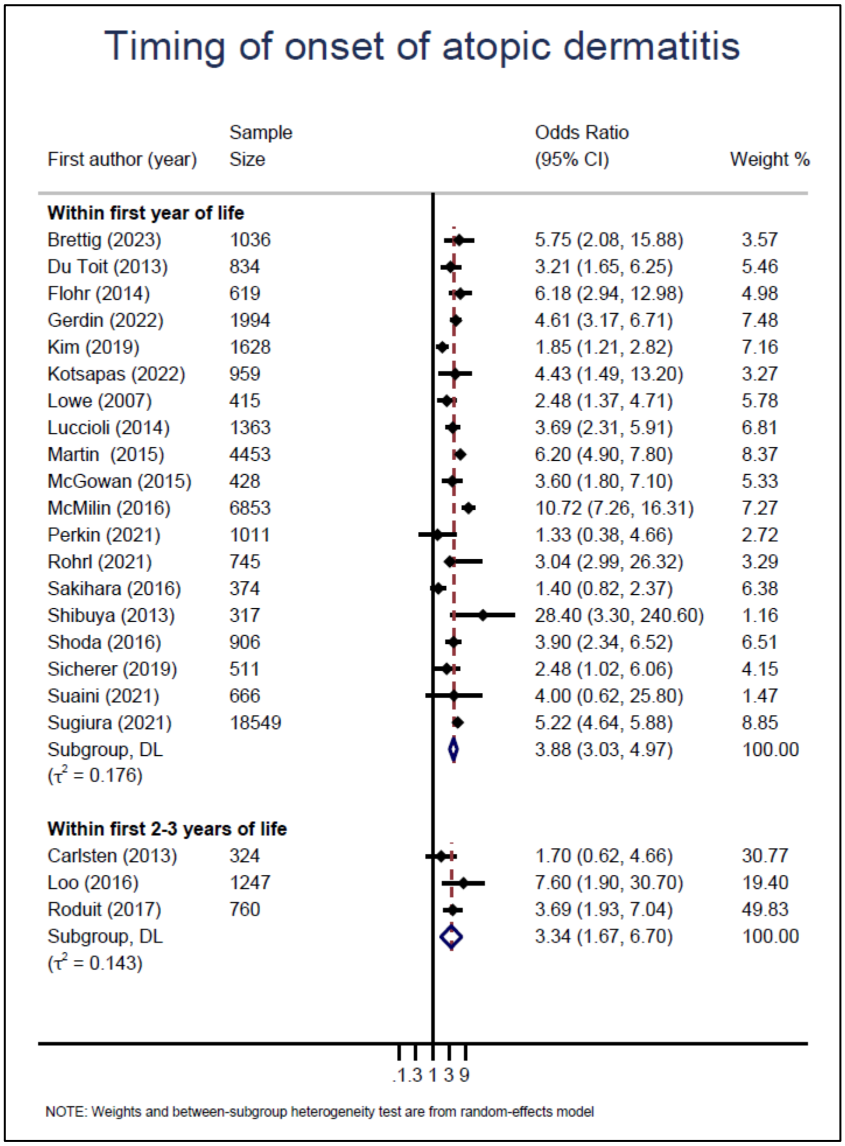

eFigure 1. Meta-Analysis of the Association Between Timing of Onset of Atopic Dermatitis in Children and Development of Food Allergy in Children

(Binary predictor: onset of AD within first year of life vs no onset, onset of AD within first 2-3 years of life vs no onset).

© 2026 Islam N et al. *JAMA Pediatrics*.

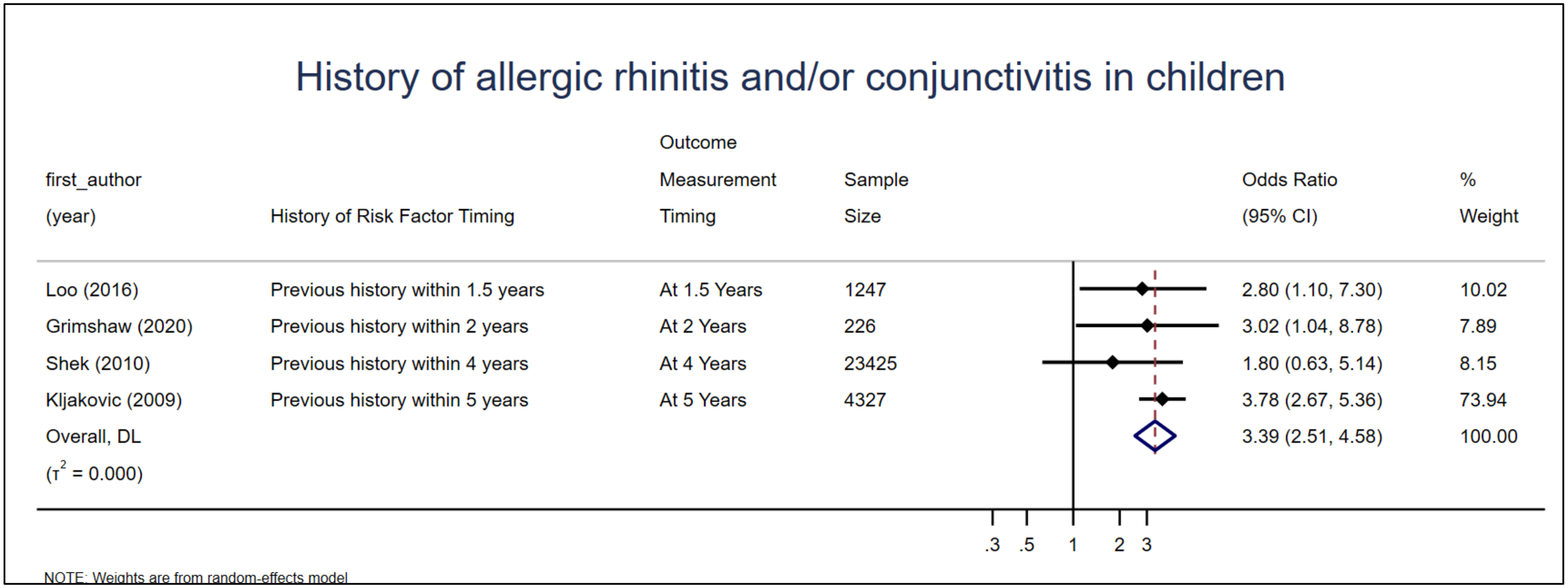

eFigure 2. Meta-Analysis of the Association Between Previous History of Allergic Rhinitis and/or Conjunctivitis in Children and Development of Food Allergy in Children

(Binary predictor: previous history of allergic rhinitis and/or conjunctivitis in children vs no previous history of allergic rhinitis and/or conjunctivitis in children).

© 2026 Islam N et al. *JAMA Pediatrics*.

History of early life wheezing in children

first_author (year)	History of Risk Factor Timing	Outcome Measurement Timing	Sample Size	Odds Ratio (95% CI)	% Weight
Shoda (2016)	Previous history within 1 year	At 3 Years	1330	1.21 (0.67, 2.19)	17.84
Loo (2016)	Previous history within 1.5 years	At 1.5 Years	1247	1.90 (0.80, 4.50)	11.66
Sakihara (2016)	Previous history within 2 years	At 2 Years	374	1.52 (0.89, 2.59)	19.56
Shek (2010)	Previous history within 4 years	At 4 Years	23425	2.93 (1.04, 8.28)	9.05
McGowan (2015)	Previous history within 5 years	At 5 Years	516	2.70 (1.40, 5.00)	16.63
Kljakovic (2009)	Previous history within 5 years	At 5 Years	4327	3.19 (2.22, 4.59)	25.25
Overall, DL (τ^2 = 0.103)				2.11 (1.46, 3.04)	100.00

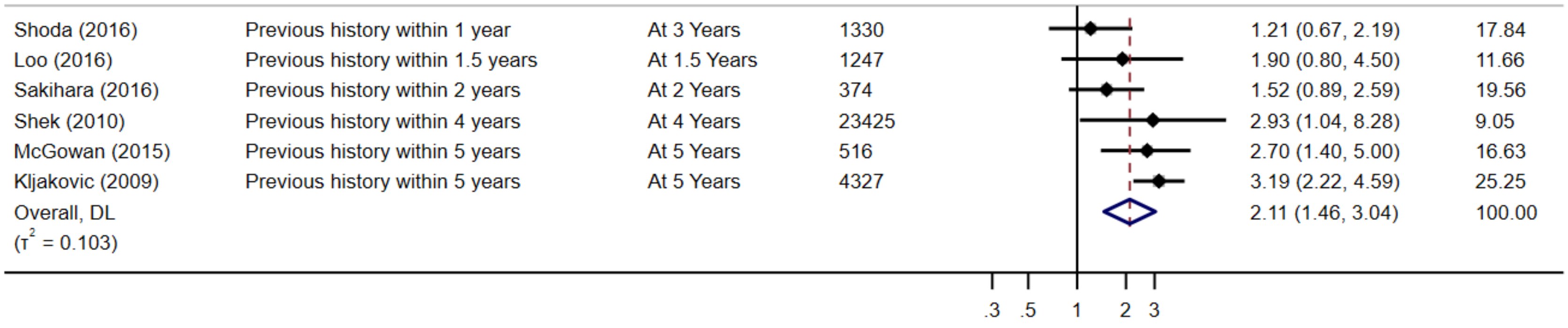

NOTE: Weights are from random-effects model

eFigure 3. Meta-Analysis of the Association Between History of Early Life Wheezing in Children and Development of Food Allergy in Children

(Binary predictor: history of early life wheezing in children vs no history of early life wheezing in children).

© 2026 Islam N et al. *JAMA Pediatrics*.

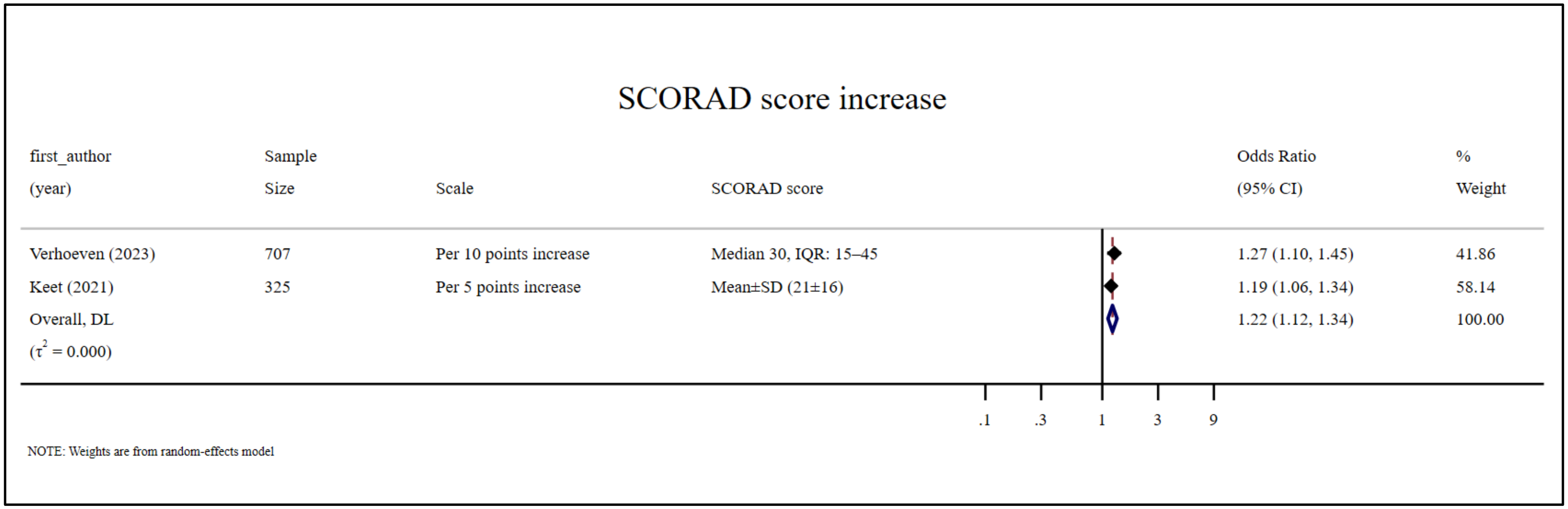

eFigure 4. Meta-Analysis of the Association Between the Severity of Atopic Dermatitis in Children and Development of Food Allergy in Children

(Continuous predictor: SCORAD score range 0–103, higher scores indicate greater severity).

© 2026 Islam N et al. *JAMA Pediatrics*.

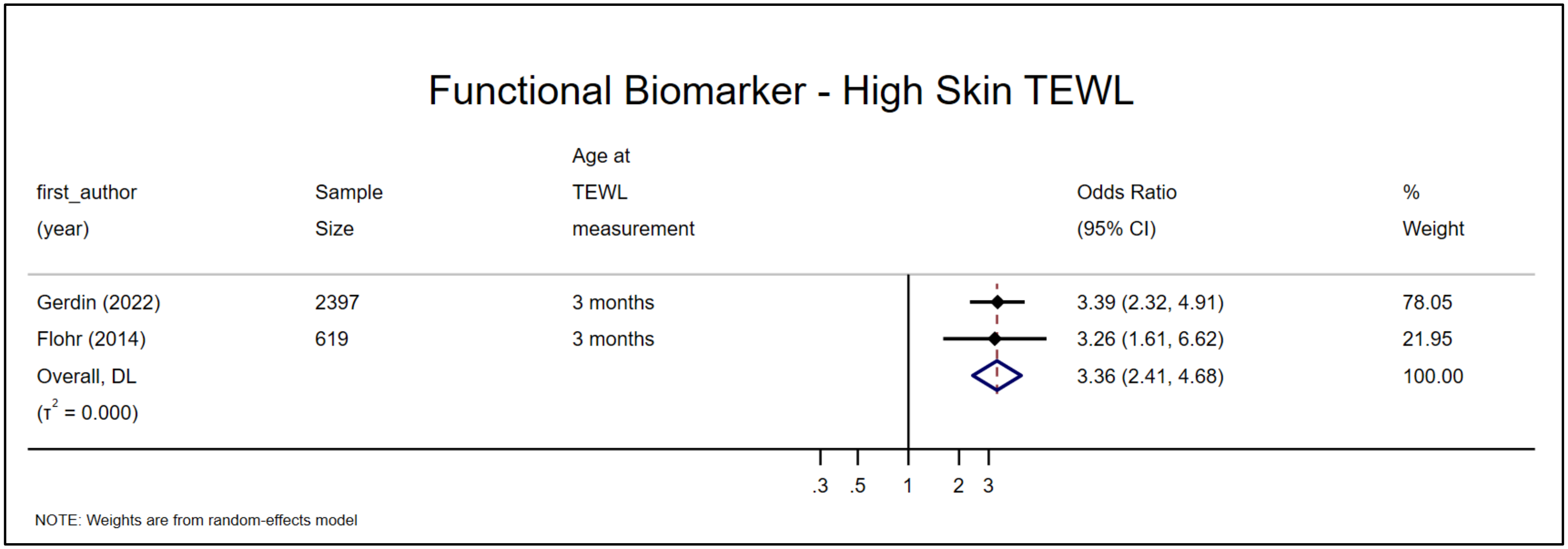

eFigure 5. Meta-Analysis of the Association Between High Skin Transepidermal Water Loss (TEWL) in Children and Development of Food Allergy in Children

(Binary predictor: high TEWL [≥9 g/m2/h] vs low TEWL [<9 g/m2/h] in children).
Additional skin-related covariates adjusted for,
Gerdin: family history of atopic diseases
Flohr: Eczema, skin TEWL

© 2026 Islam N et al. *JAMA Pediatrics*.

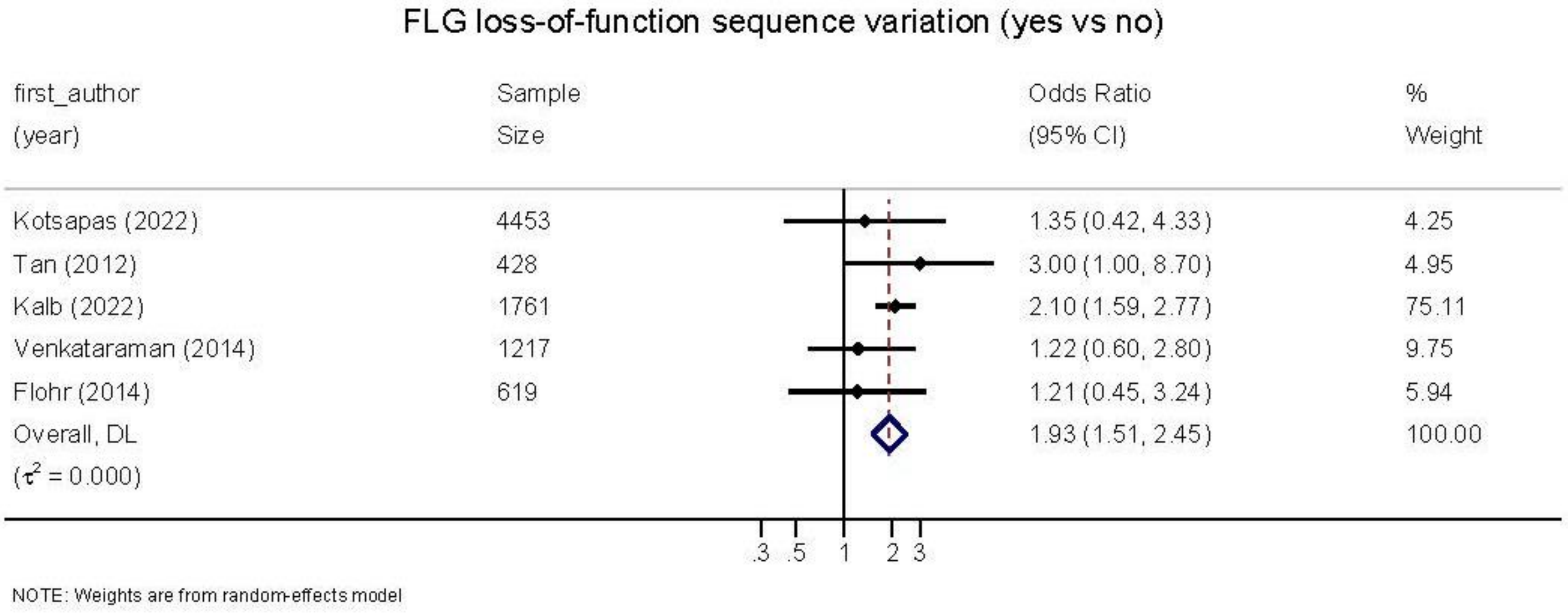

eFigure 6. Meta-Analysis of the Association Between Filaggrin Gene (FLG) Loss-of-Function Sequence Variations in Children and Development of Food Allergy in Children

(Binary predictor: FLG loss-of-function sequence variations vs no FLG loss-of-function sequence variations).
Additional skin-related covariates adjusted for,
Kalb: Eczema
Kotsapsas: Eczema, parental atopy
Tan: Eczema, family history of allergic diseases
Venkataraman: None
Flohr: Eczema, skin TEWL

© 2026 Islam N et al. *JAMA Pediatrics*.

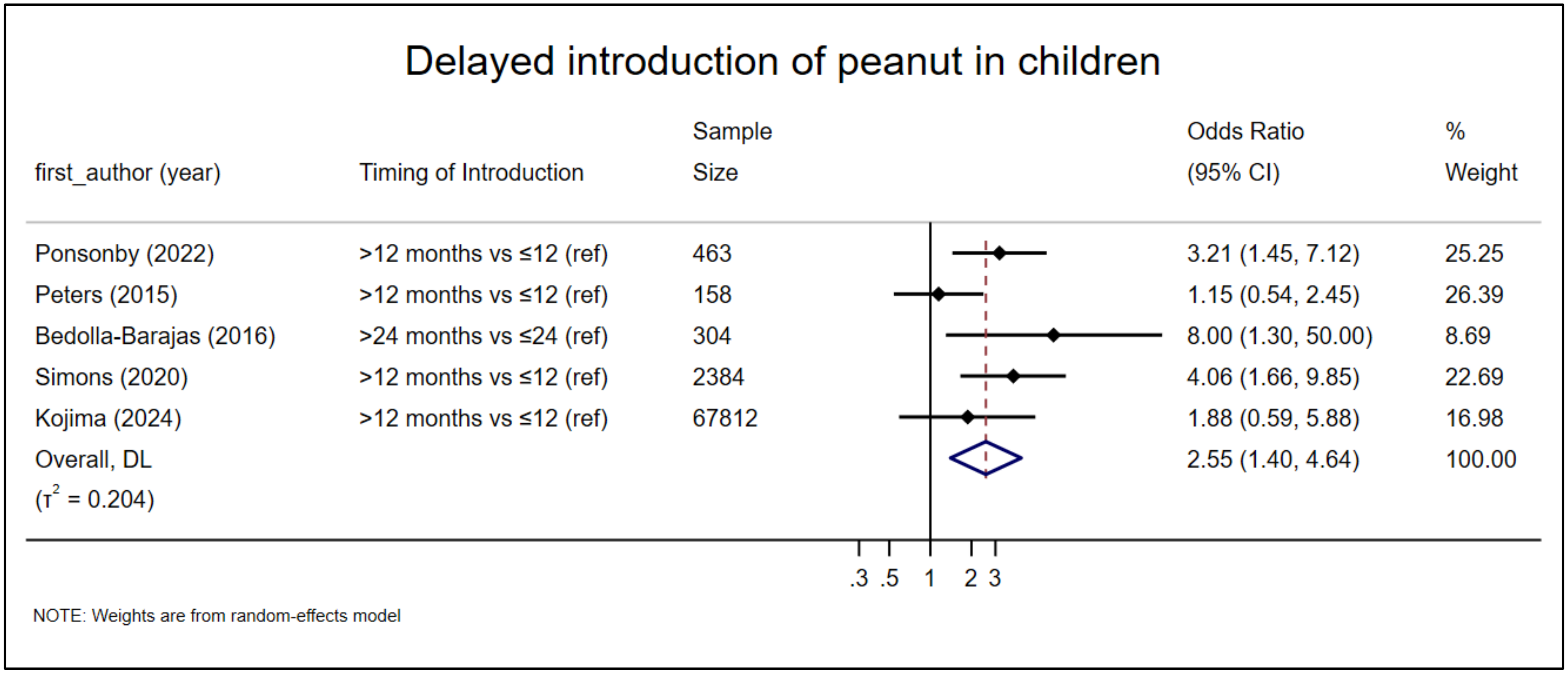

eFigure 7. Meta-Analysis of the Association Between Delayed Introduction of Peanut and Development of Food Allergy to the Displayed Food in Children

(Binary predictor: delayed introduction of peanut (>12 months) vs early introduction of peanut).

© 2026 Islam N et al. *JAMA Pediatrics*.

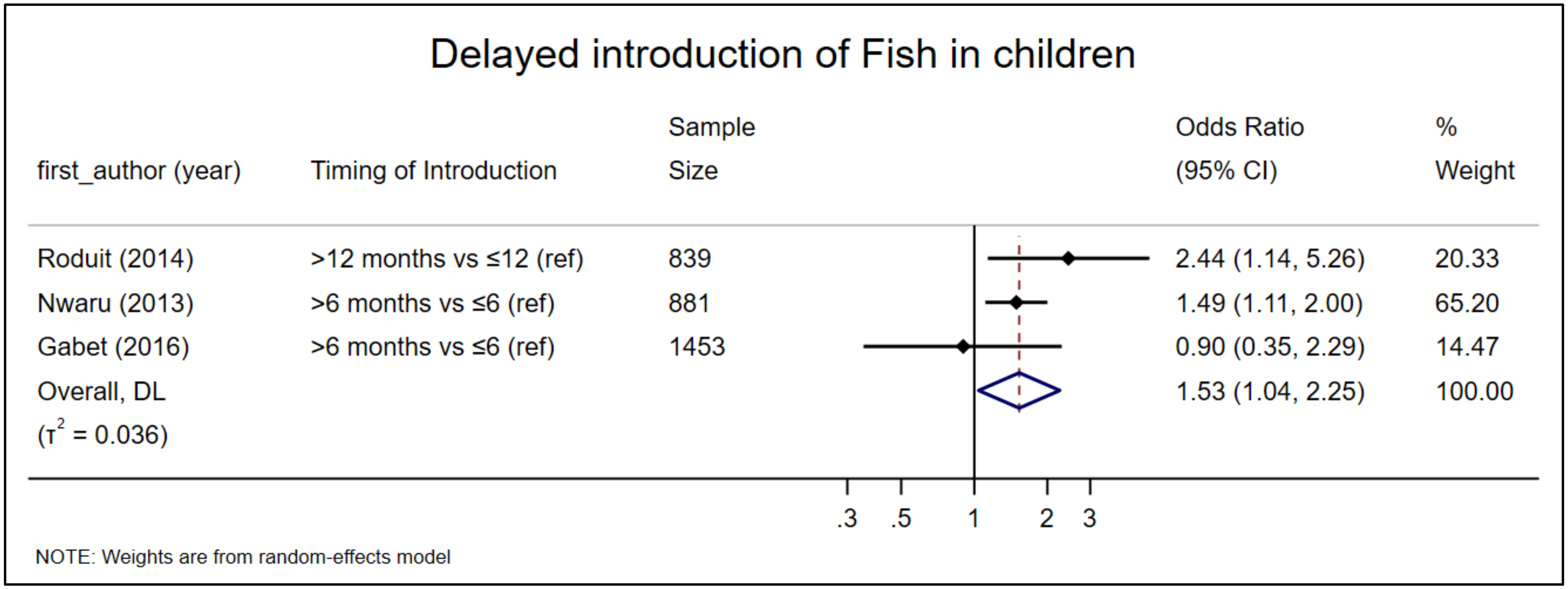

eFigure 8. Meta-Analysis of the Association Between Delayed Introduction of Fish and Development of Food Allergy to the Displayed Food in Children

(Binary predictor: delayed introduction of fish (>6 months) vs early introduction of fish).

© 2026 Islam N et al. *JAMA Pediatrics*.

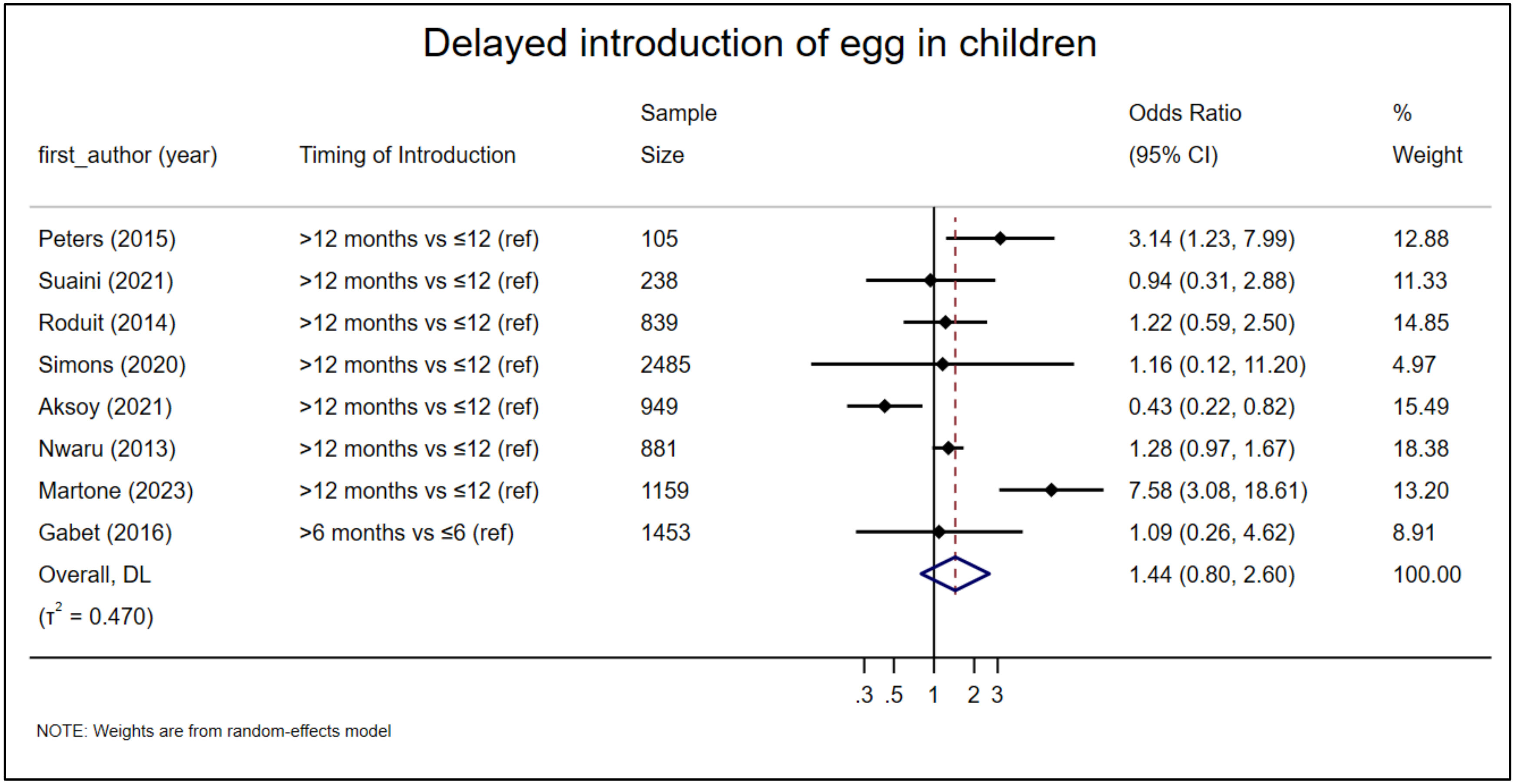

eFigure 9. Meta-Analysis of the Association Between Delayed Introduction of Egg and Development of Food Allergy to the Displayed Food in Children

© 2026 Islam N et al. *JAMA Pediatrics*.

(Binary predictor: delayed introduction of egg (>6 months) vs early introduction of egg).

© 2026 Islam N et al. *JAMA Pediatrics*.

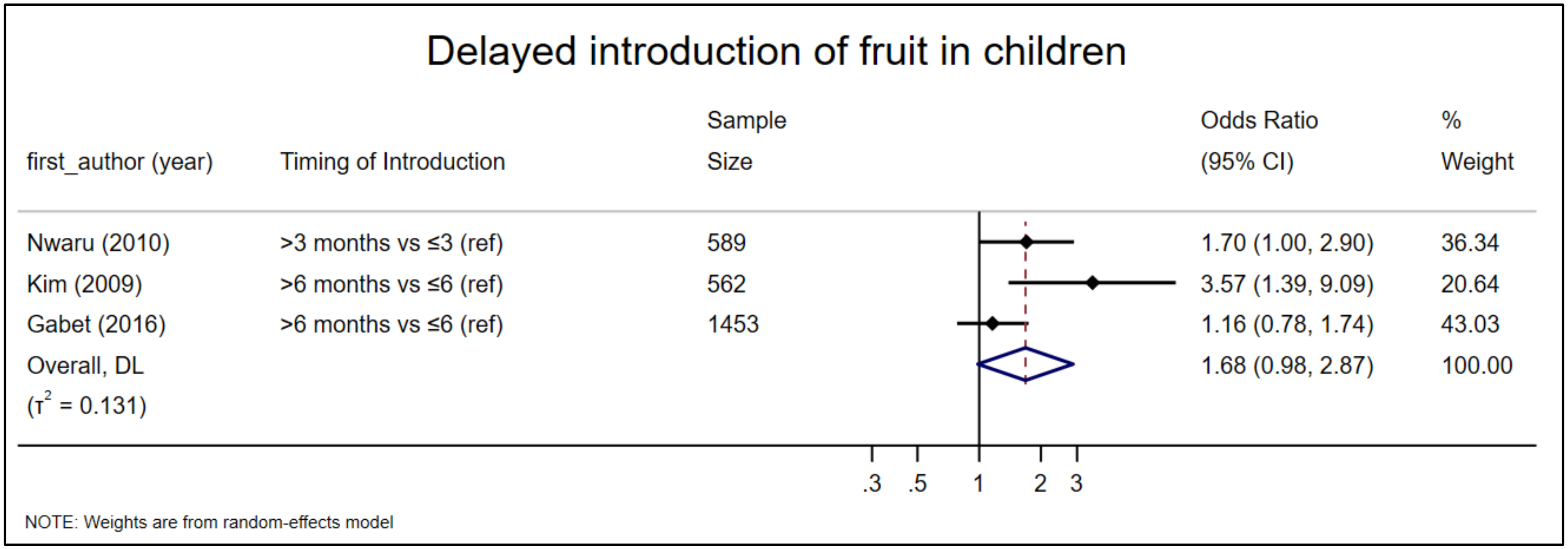

eFigure 10. Meta-Analysis of the Association Between Delayed Introduction of Fruit and Development of Food Allergy to the Displayed Food in Children

(Binary predictor: delayed introduction of fruit (>3 months) vs early introduction of fruit).

© 2026 Islam N et al. *JAMA Pediatrics*.

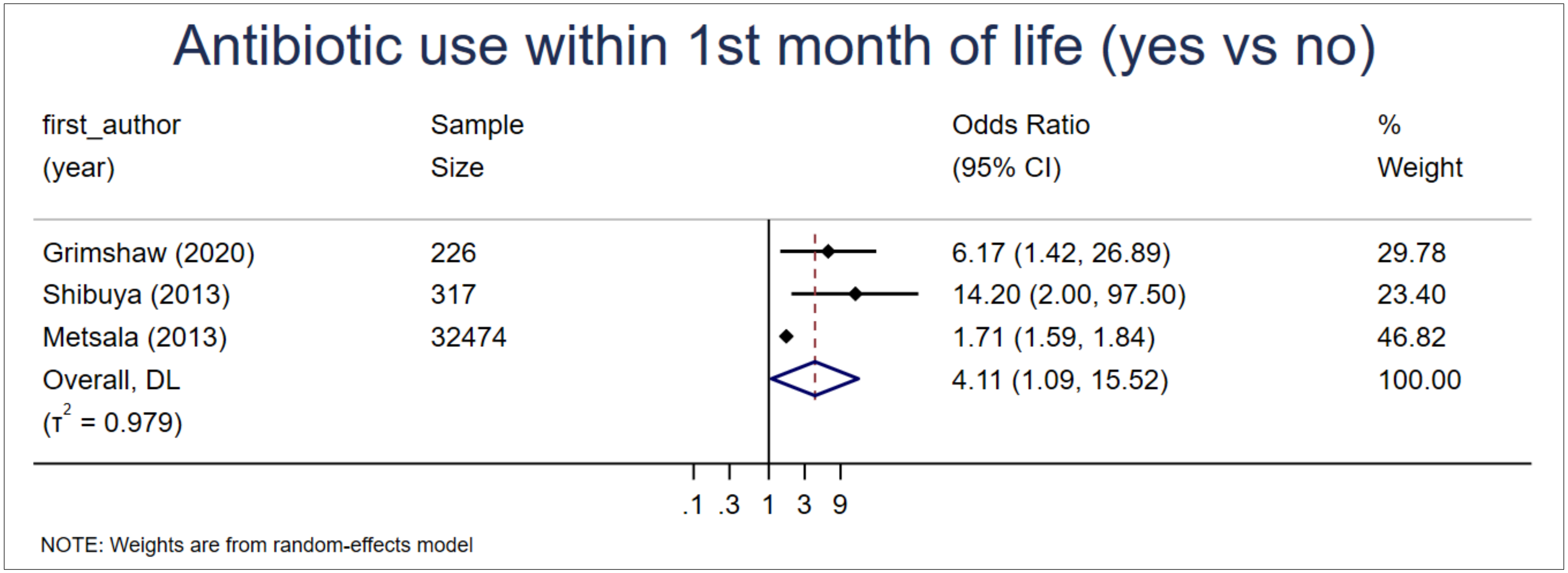

eFigure 11. Meta-Analysis of the Association Between Antibiotic Use in Children Within 1st Month of Life and Development of Food Allergy in Children

(Binary predictor: antibiotic use in children within 1st month of life vs no antibiotic use in children within 1st month of life).

© 2026 Islam N et al. *JAMA Pediatrics*.

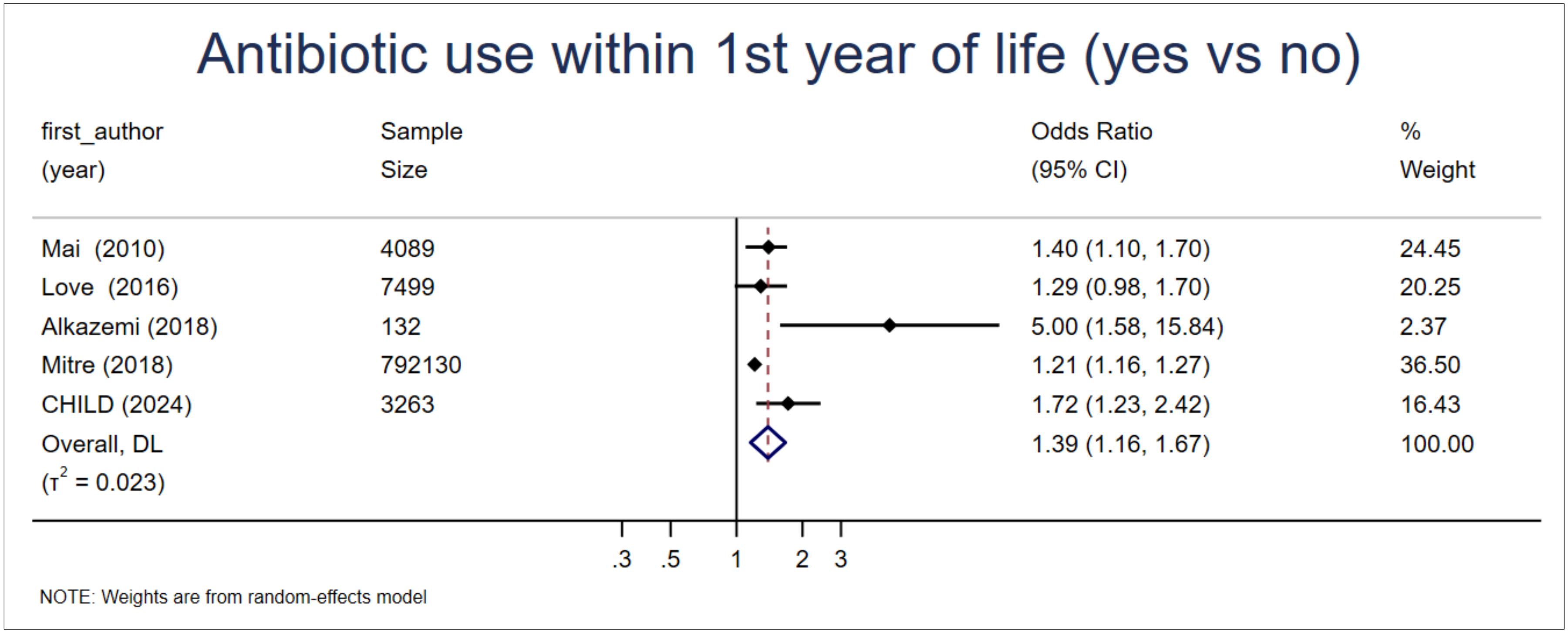

eFigure 12. Meta-Analysis of the Association Between Antibiotic Use in Children Within 1st Year of Life and Development of Food Allergy in Children

(Binary predictor: antibiotic use in children within 1st year of life vs no antibiotic use in children within 1st year of life).

© 2026 Islam N et al. *JAMA Pediatrics*.

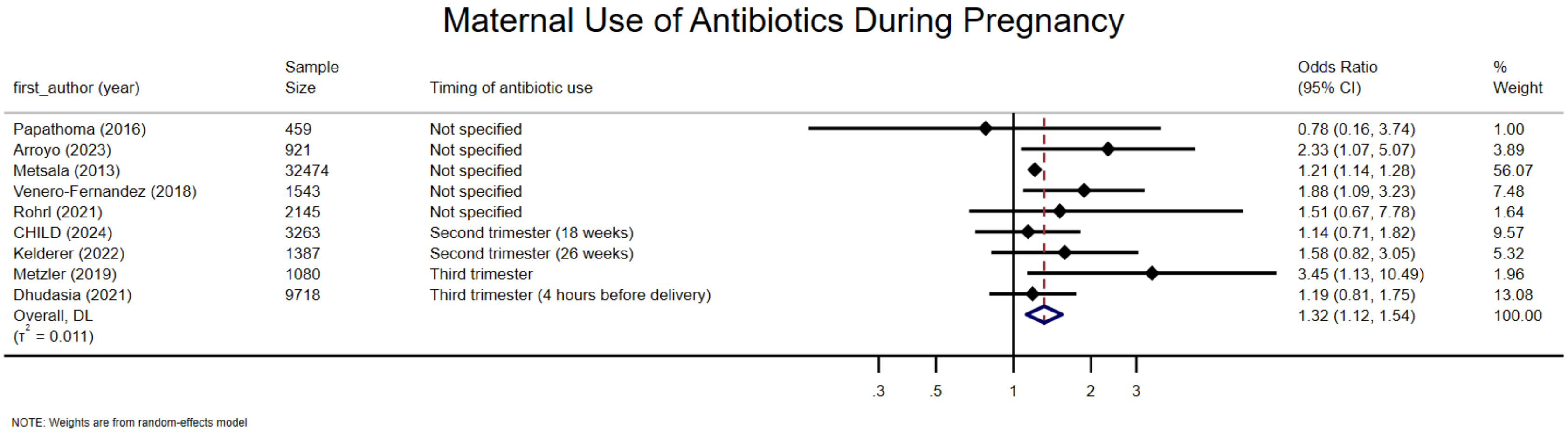

eFigure 13. Meta-Analysis of the Association Between Maternal Use of Antibiotics During Pregnancy and Development of Food Allergy in Children

(Binary predictor: maternal use of antibiotics during pregnancy vs no maternal use of antibiotics during pregnancy).

© 2026 Islam N et al. *JAMA Pediatrics*.

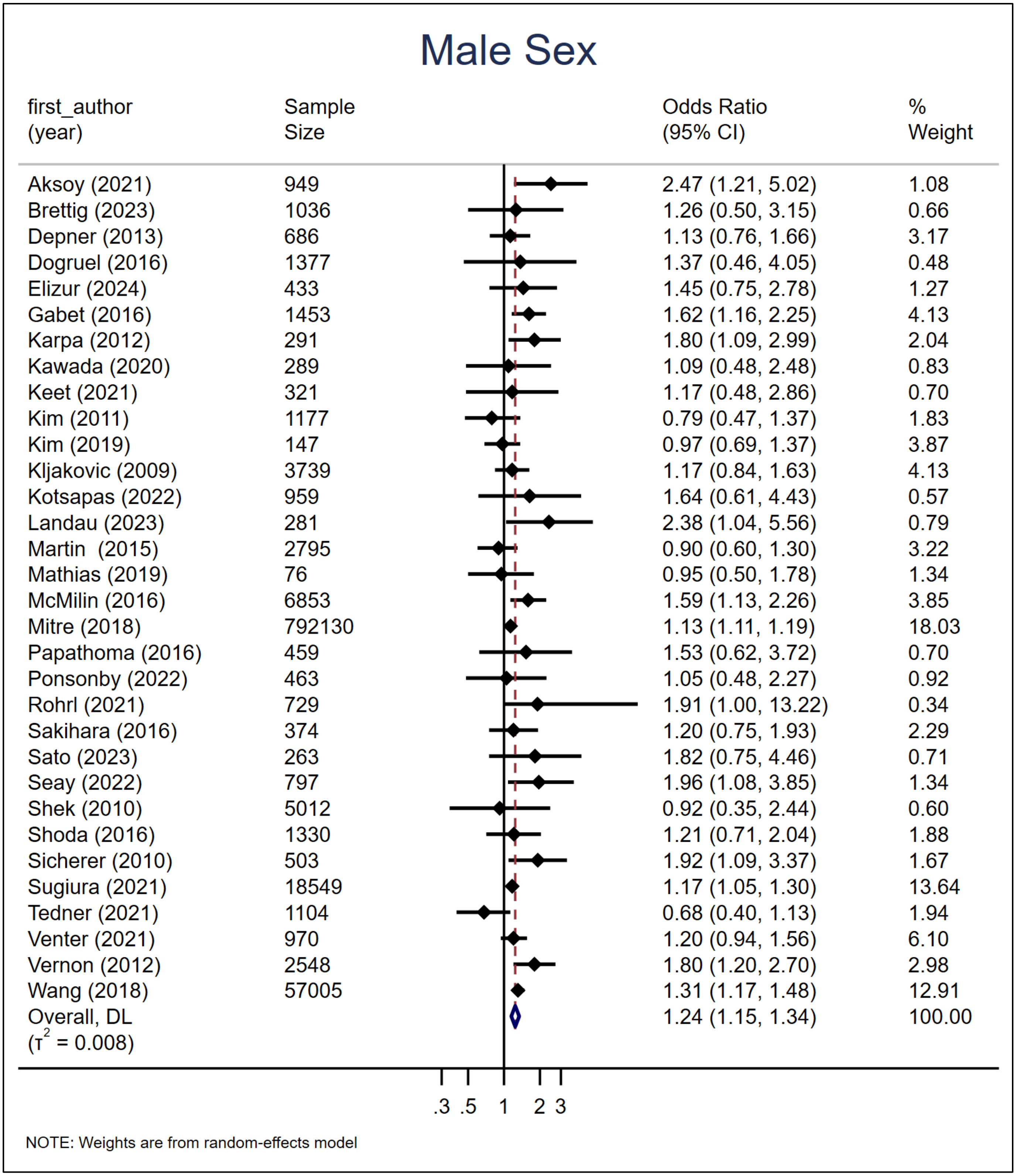

eFigure 14. Meta-Analysis of the Association Between Sex and Development of Food Allergy in Children (Binary predictor: male sex vs female sex).

© 2026 Islam N et al. *JAMA Pediatrics*.

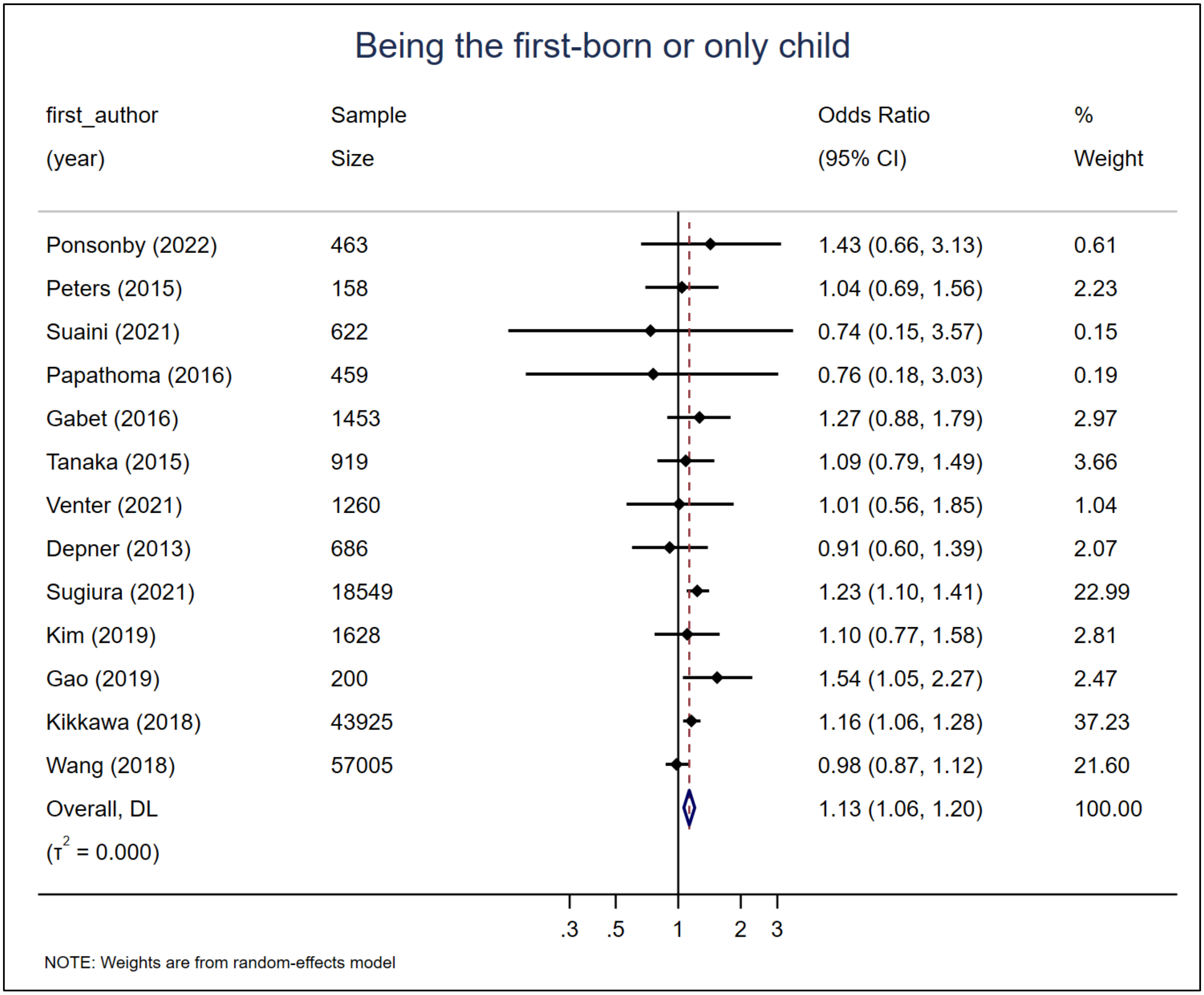

eFigure 15. Meta-Analysis of the Association Between Being the Firstborn Child and Development of Food Allergy in Children

(Binary predictor: being the first-born child vs not being the first-born child).

© 2026 Islam N et al. *JAMA Pediatrics*.

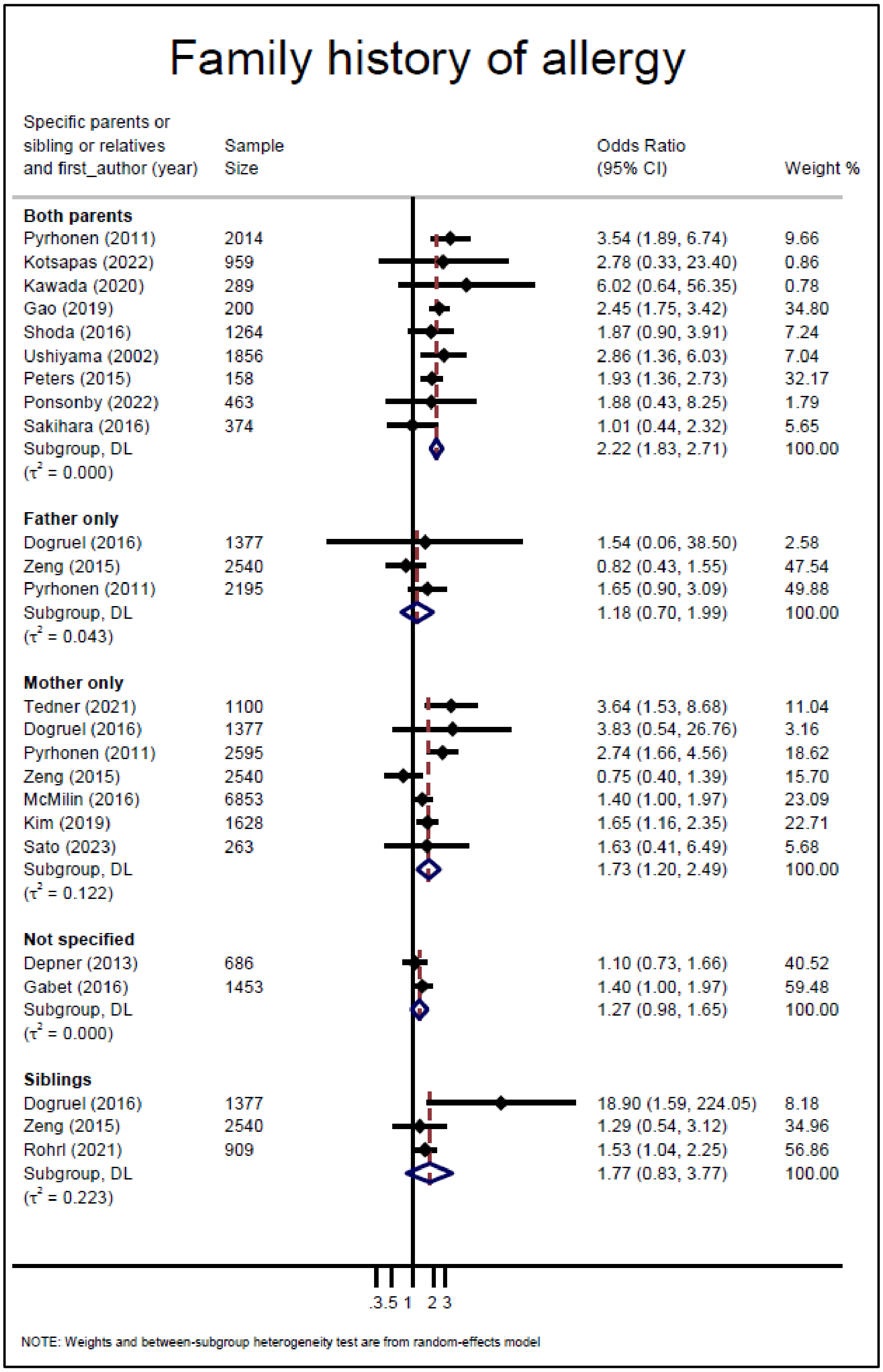

eFigure 16. Meta-Analysis of the Association Between Allergic Disorders (Asthma, Atopic Dermatitis, Food Allergy, Allergic Rhinitis, and/or Conjunctivitis) and Development of Food Allergy in Children

(Binary predictor: family history of allergy vs no family history of allergy).

© 2026 Islam N et al. *JAMA Pediatrics*.

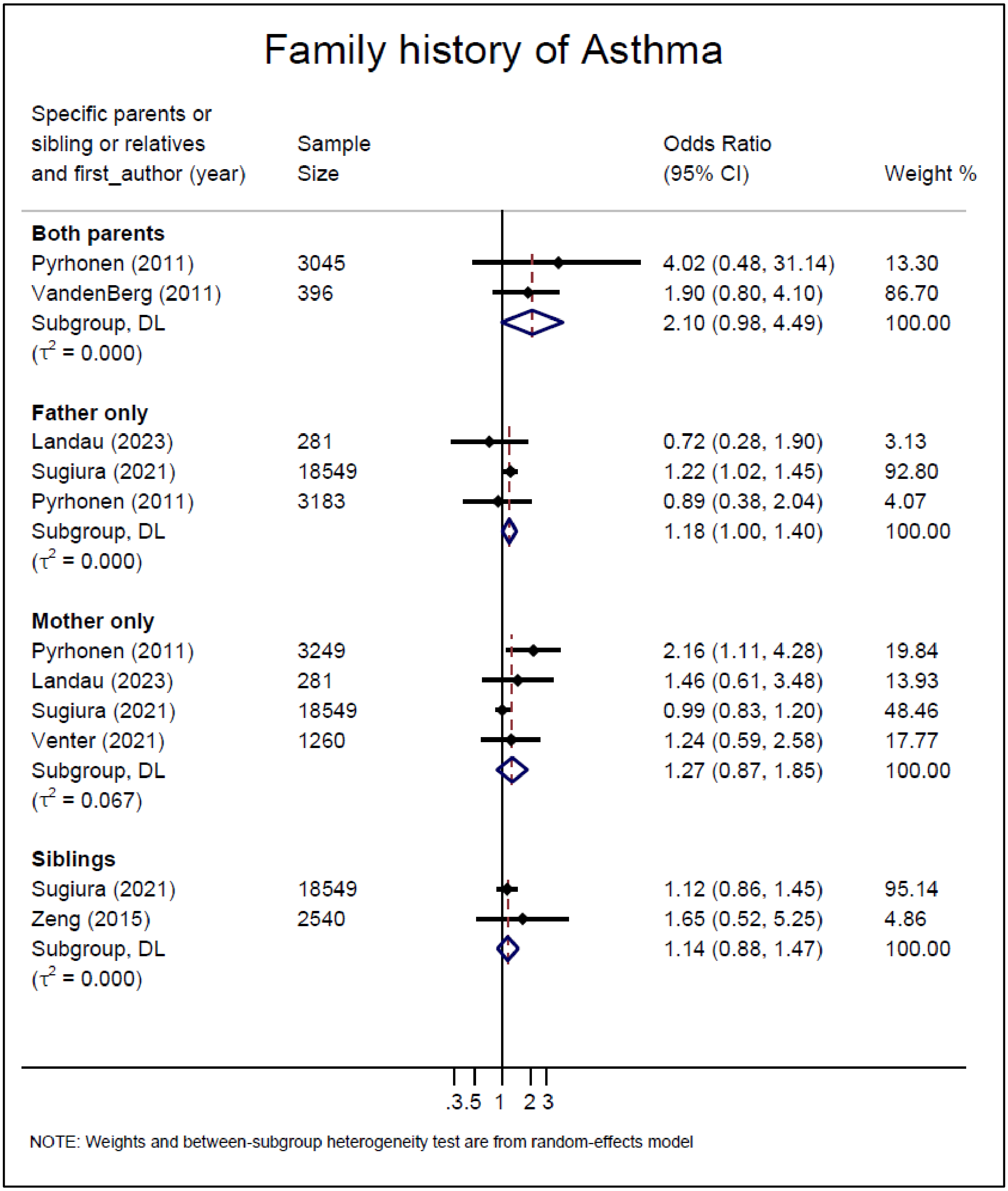

eFigure 17. Meta-Analysis of the Association Between Family History of Asthma and Development of Food Allergy in Children

(Binary predictor: family history of asthma vs no family history of asthma).

© 2026 Islam N et al. *JAMA Pediatrics*.

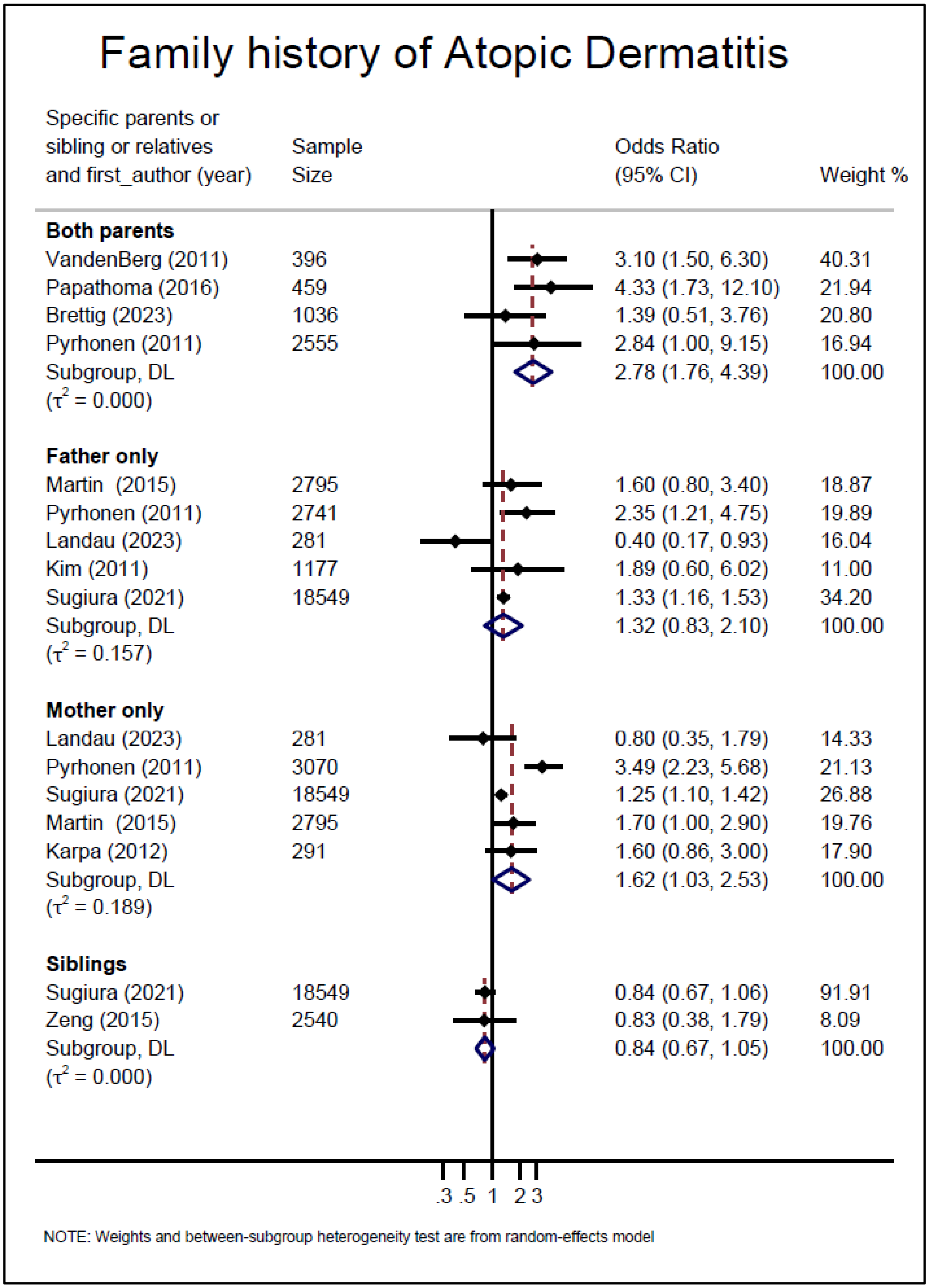

eFigure 18. Meta-Analysis of the Association Between Family History of Atopic Dermatitis (Eczema) and Development of Food Allergy in Children

(Binary predictor: family history of atopic dermatitis (eczema) vs no family history of atopic dermatitis (eczema)).

© 2026 Islam N et al. *JAMA Pediatrics*.

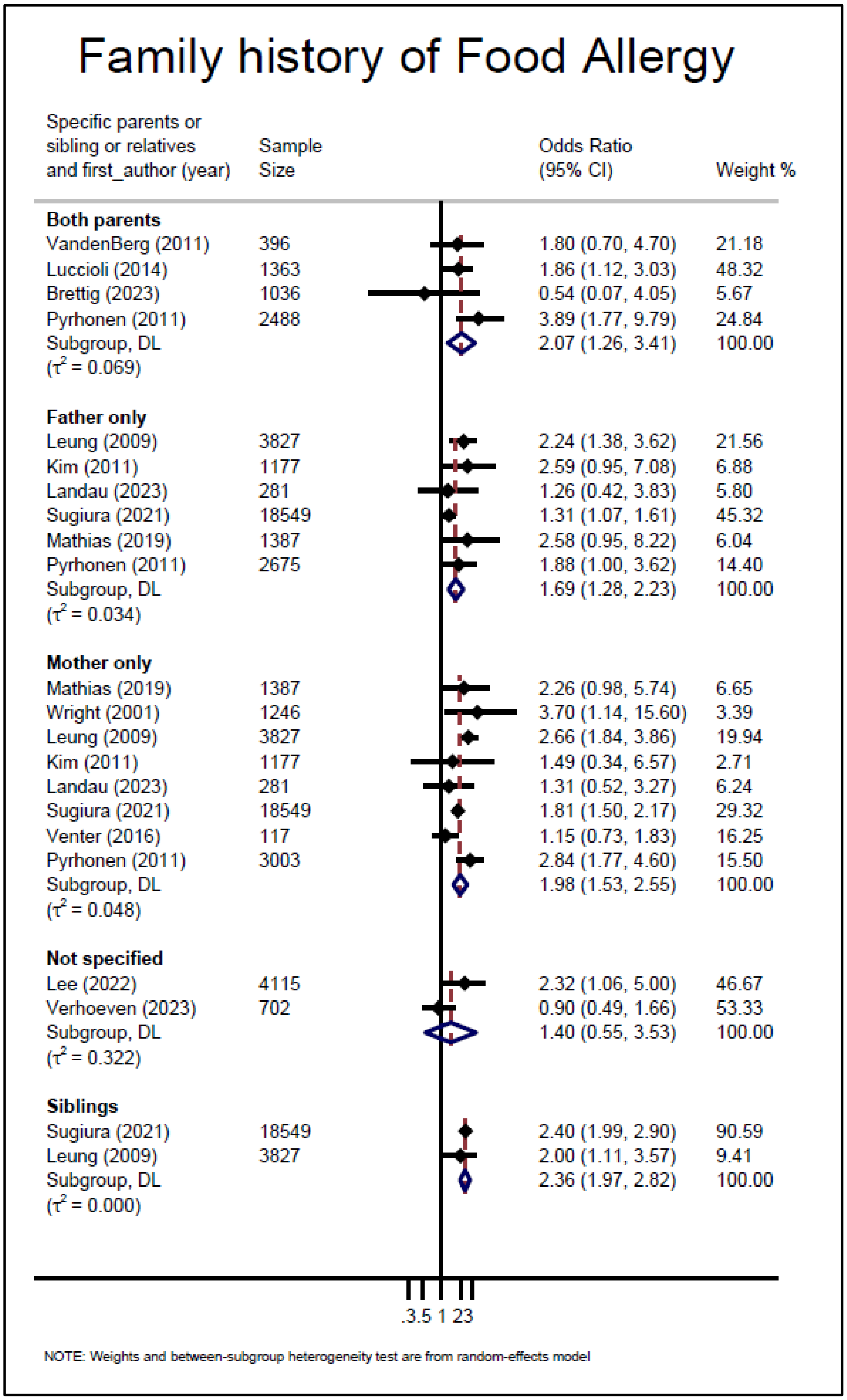

eFigure 19. Meta-Analysis of the Association Between Family History of Food Allergy and Development of Food Allergy in Children

(Binary predictor: family history of food allergy vs no family history of food allergy).

© 2026 Islam N et al. *JAMA Pediatrics*.

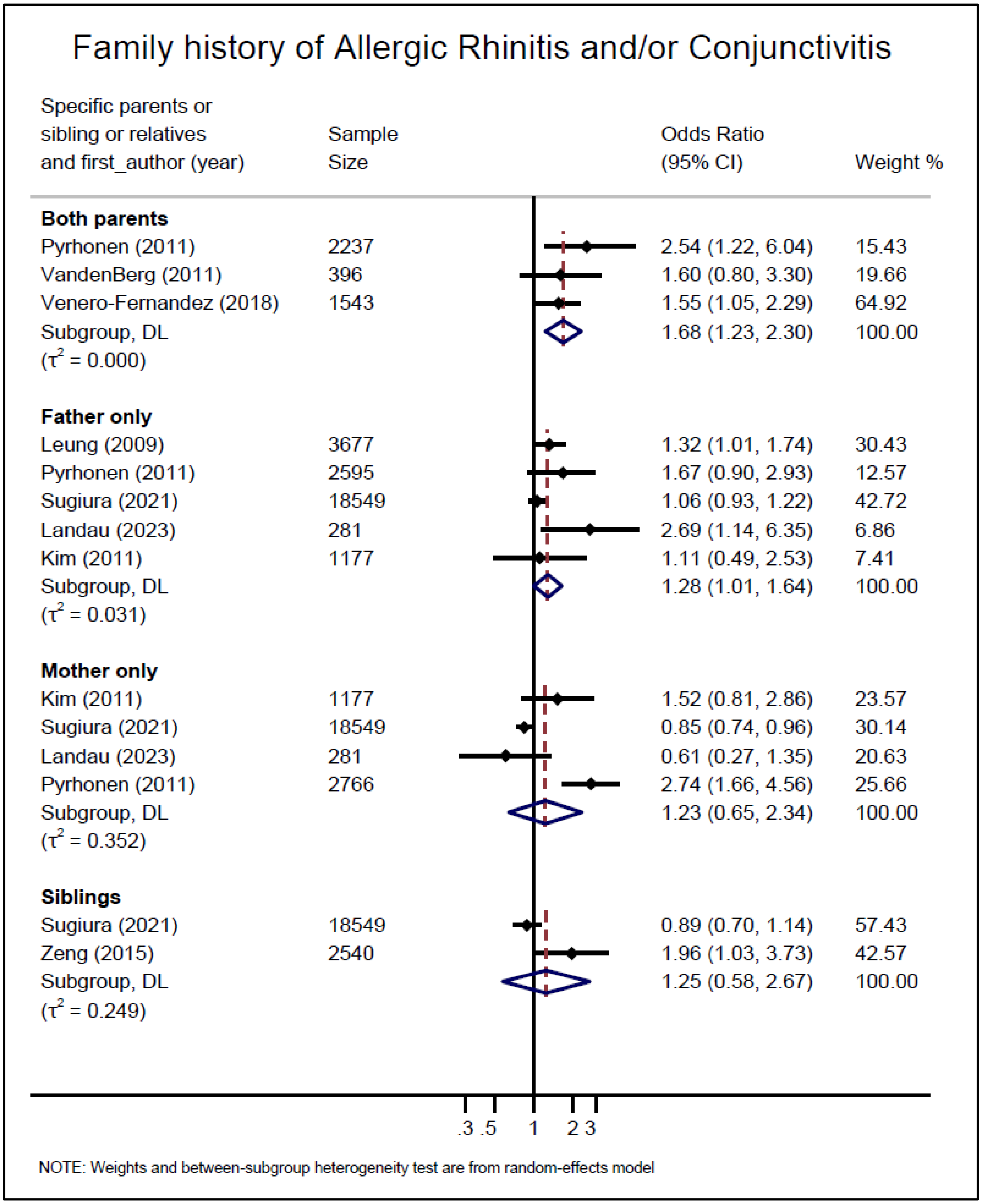

eFigure 20. Meta-Analysis of the Association Between Family History of Allergic Rhinitis and/or Conjunctivitis and Development of Food Allergy in Children

(Binary predictor: family history of allergic rhinitis and/or conjunctivitis vs no family history of allergic rhinitis and/or conjunctivitis).

© 2026 Islam N et al. *JAMA Pediatrics*.

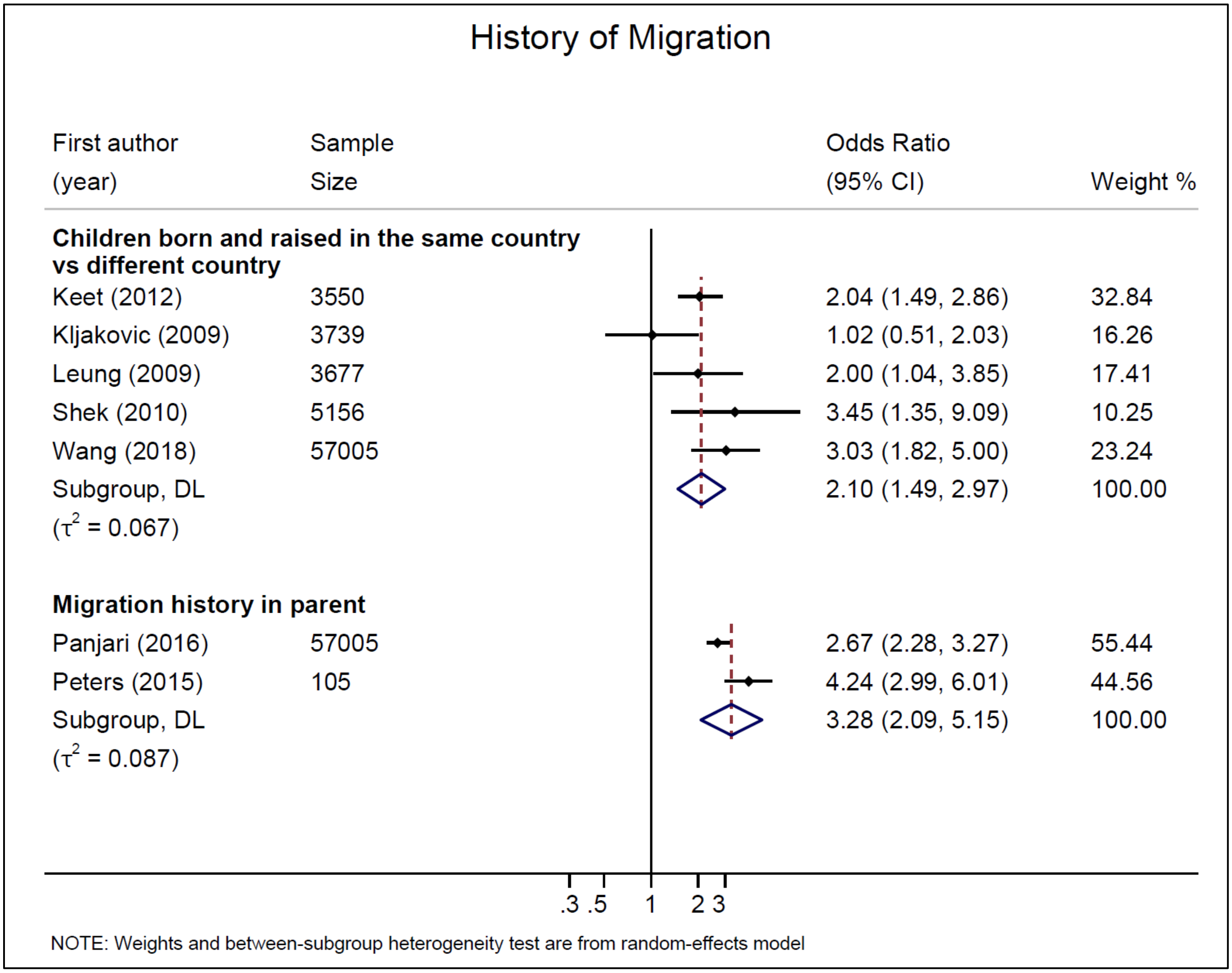

eFigure 21. Meta-Analysis of the Association Between Migration History and Development of Food Allergy in Children

(Binary predictor: Children born and raised in the same country vs different country, migration history in parents vs no migration history in parents).

© 2026 Islam N et al. *JAMA Pediatrics*.

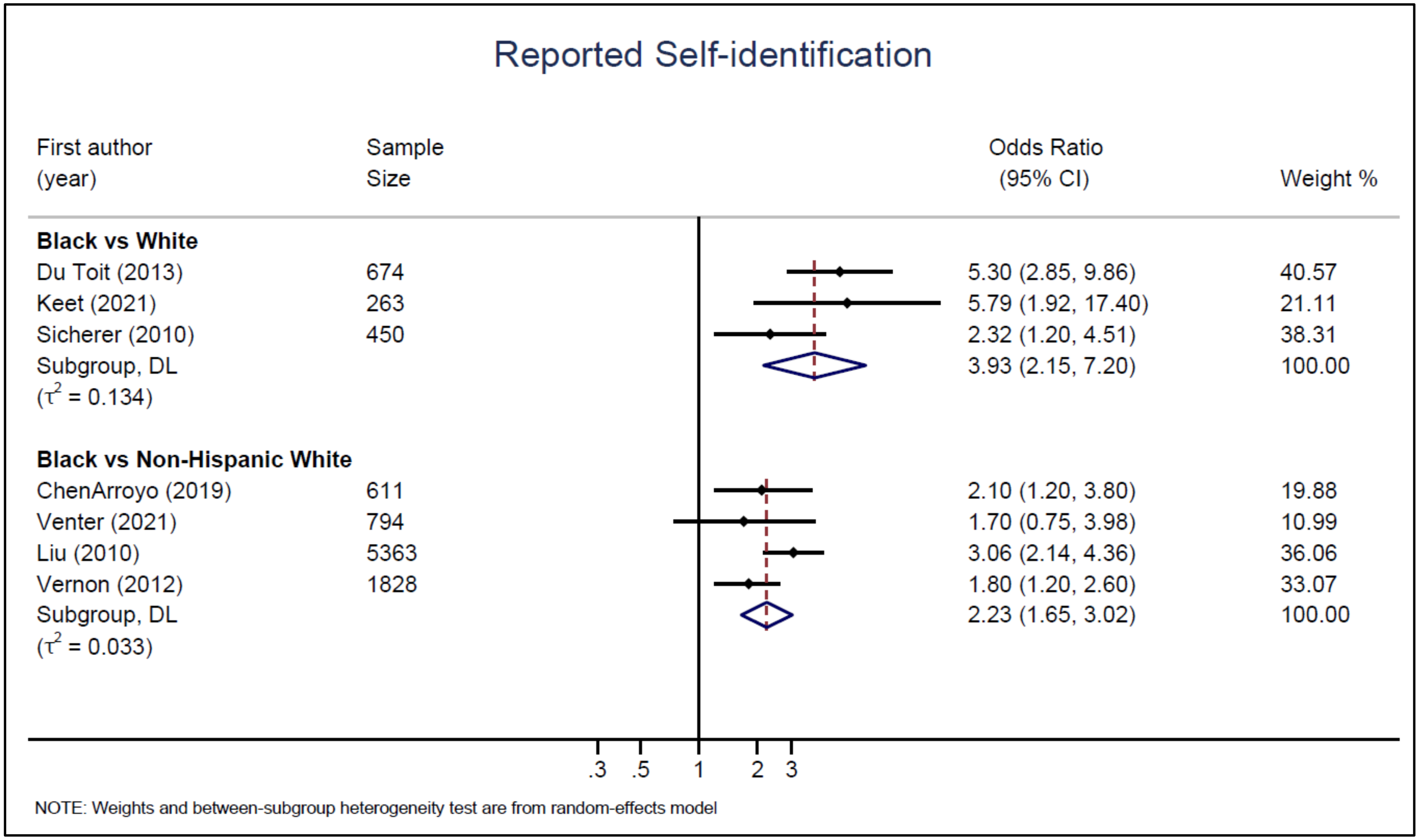

eFigure 22. Meta-Analysis of the Association Between Reported Self-Identification and Development of Food Allergy in Children

(Binary predictor: reported self-identification as Black vs White and reported self-identification as Black vs non-Hispanic White).

© 2026 Islam N et al. *JAMA Pediatrics*.

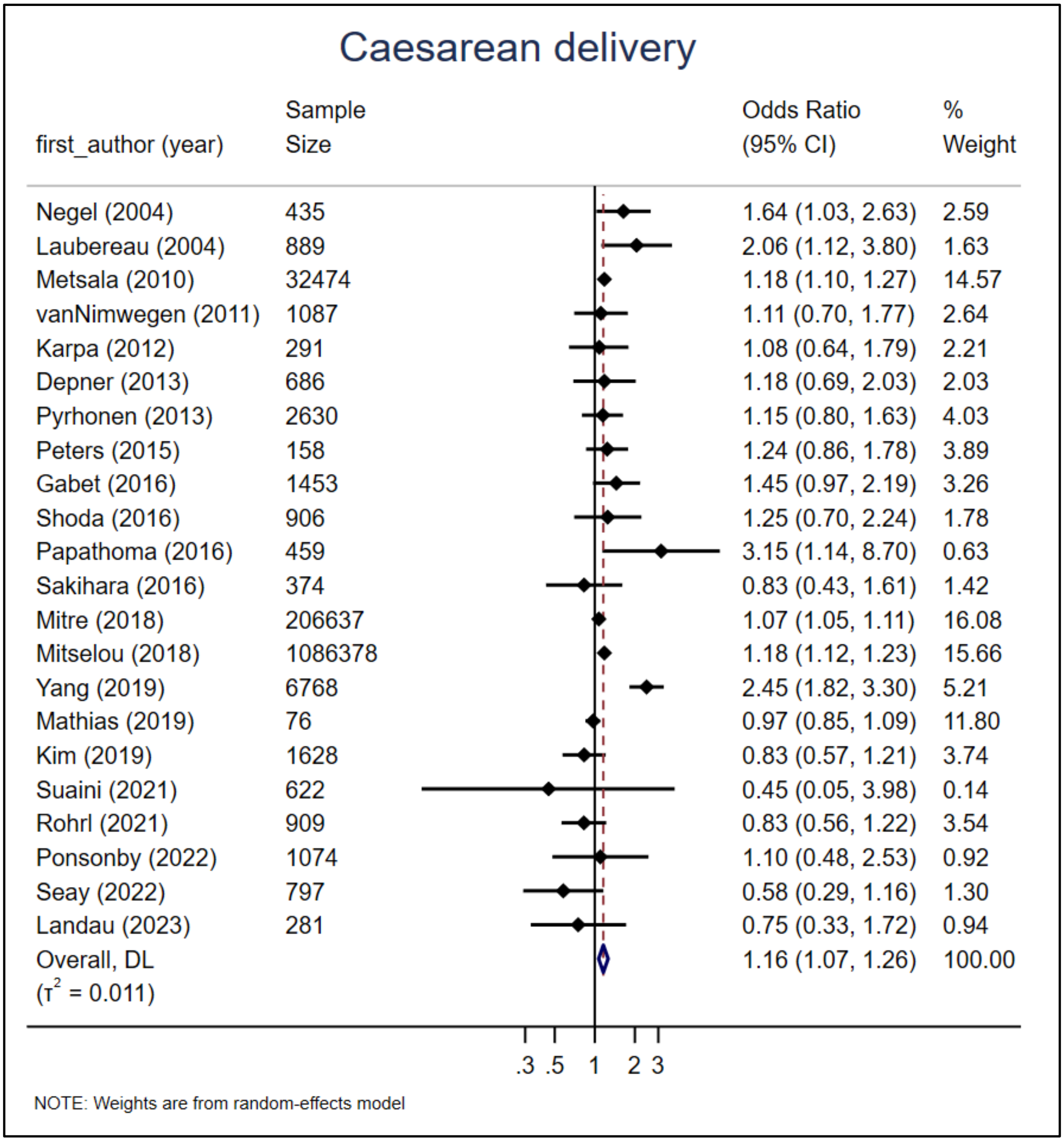

eFigure 23. Meta-Analysis of the Association Between Any Caesarean Delivery and Development of Food Allergy in Children

(Binary predictor: any caesarean delivery vs vaginal delivery).

© 2026 Islam N et al. *JAMA Pediatrics*.

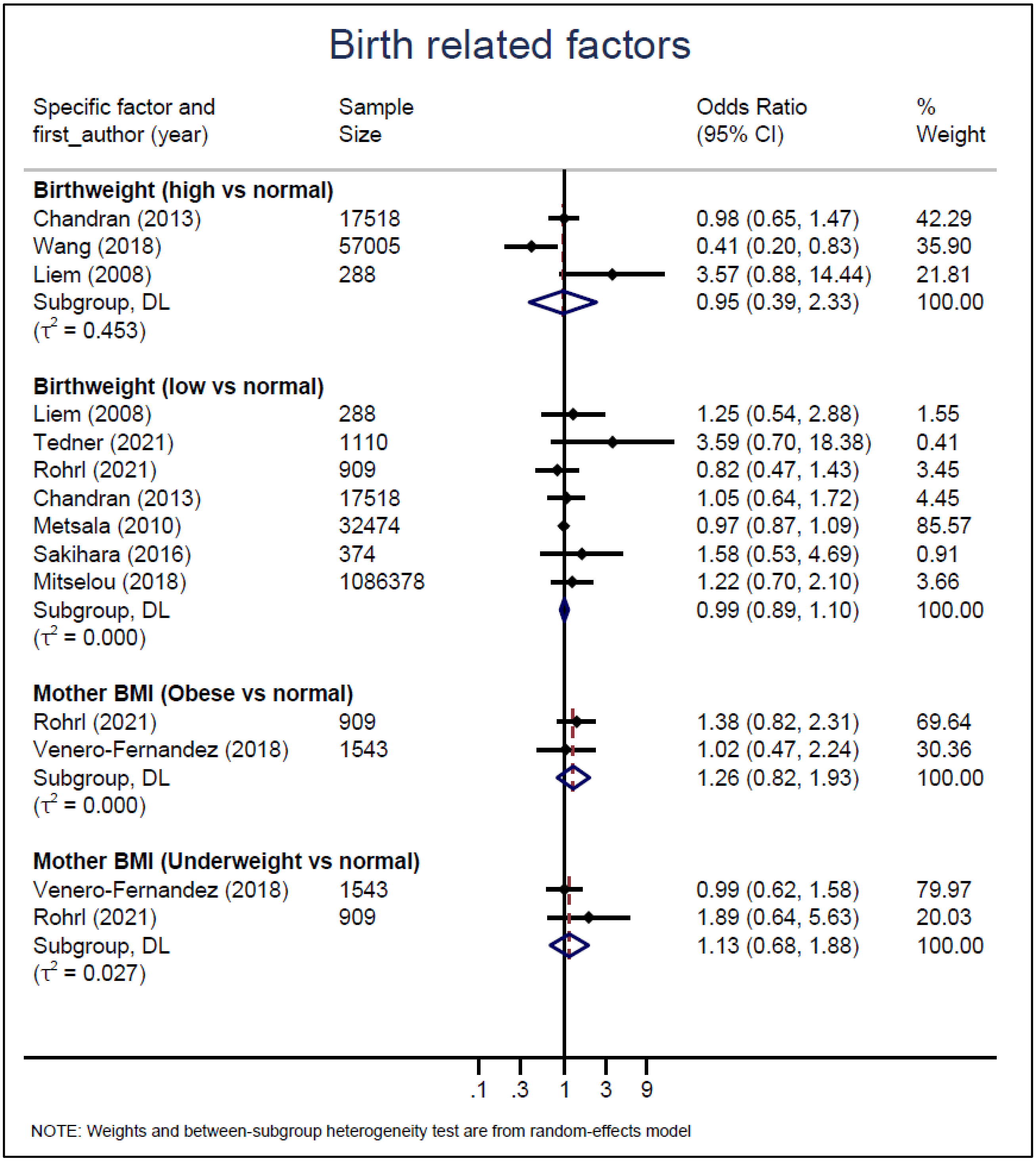

eFigure 24. Meta-Analysis of the Association Between High Birth Weight, Low Birth Weight, Mother BMI Obese, Mother BMI Underweight and Development of Food Allergy in Children

(Binary predictor: high birthweight vs normal birthweight, low birthweight vs normal birthweight, mother BMI obese vs mother BMI normal, mother BMI underweight vs mother BMI normal).

© 2026 Islam N et al. *JAMA Pediatrics*.

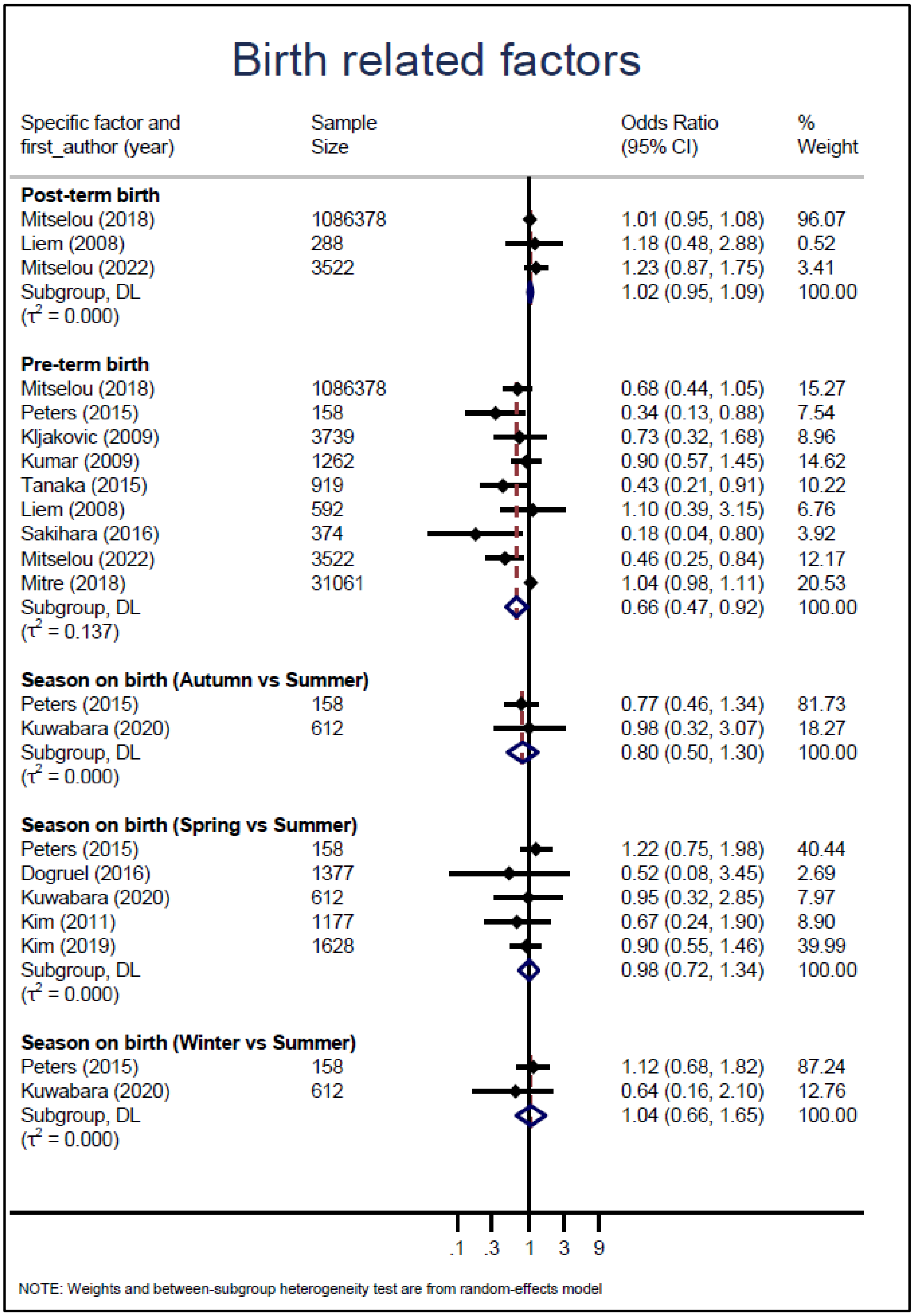

eFigure 25. Meta-Analysis of the Association Between Postterm Birth, Preterm Birth, Season of Birth (Autumn), Season of Birth (Spring), Season of Birth (Winter) and Development of Food Allergy in Children

(Binary predictor: post-term birth vs no post-term birth, pre-term birth vs no pre-term birth, season of birth (autumn) vs season of birth (summer), season of birth (spring) vs season of birth (summer) and season of birth (winter) vs season of birth (summer).

© 2026 Islam N et al. *JAMA Pediatrics*.

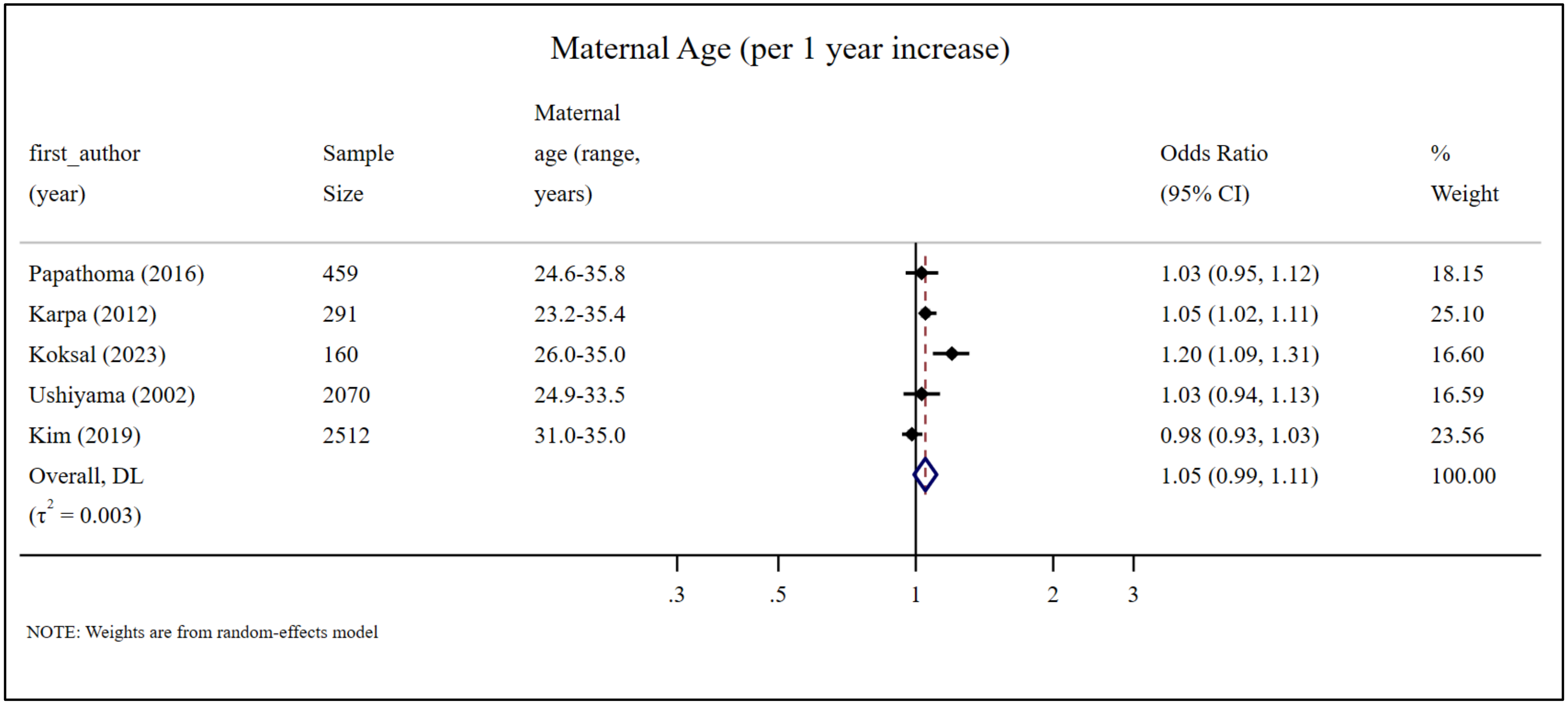

eFigure 26. Meta-Analysis of the Association Between Maternal Age and Development of Food Allergy in Children

© 2026 Islam N et al. *JAMA Pediatrics*.

(Numerical predictor: maternal age-per 1 year increase).

© 2026 Islam N et al. *JAMA Pediatrics*.

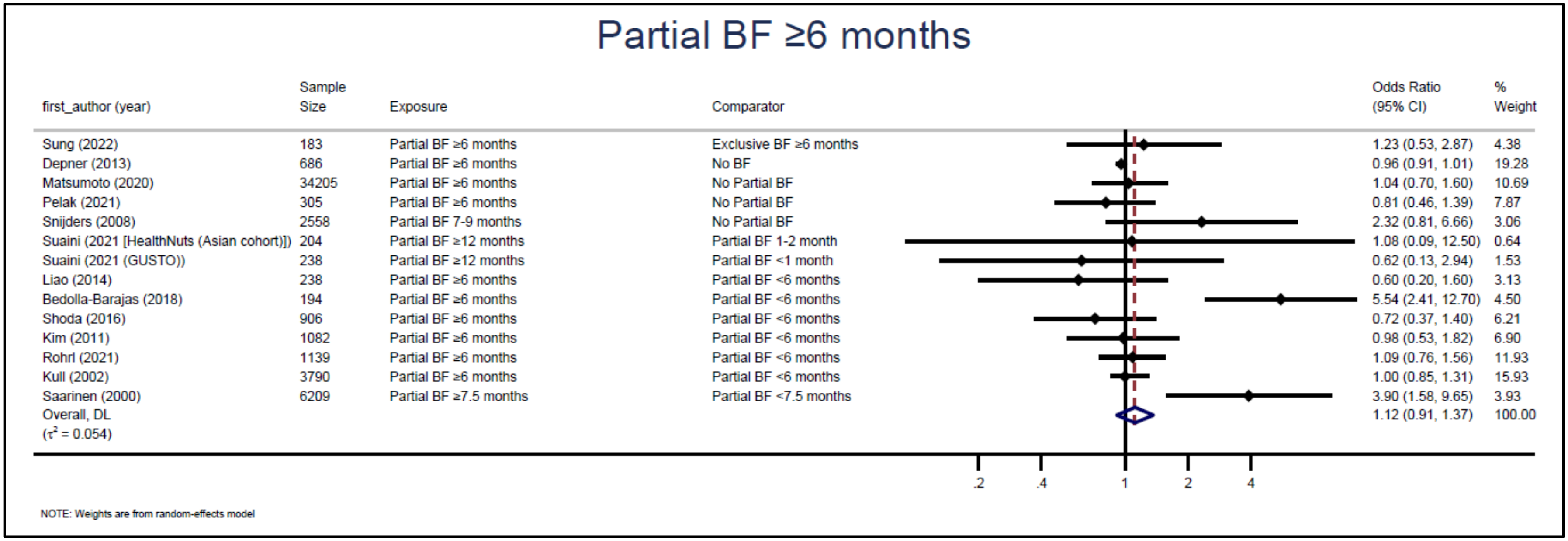

eFigure 27. Meta-Analysis of the Association Between Pattern and Duration of Breastfeeding and Development of Food Allergy in Children

(Binary predictor: partial breastfeeding (≥6 months) vs comparators).

© 2026 Islam N et al. *JAMA Pediatrics*.

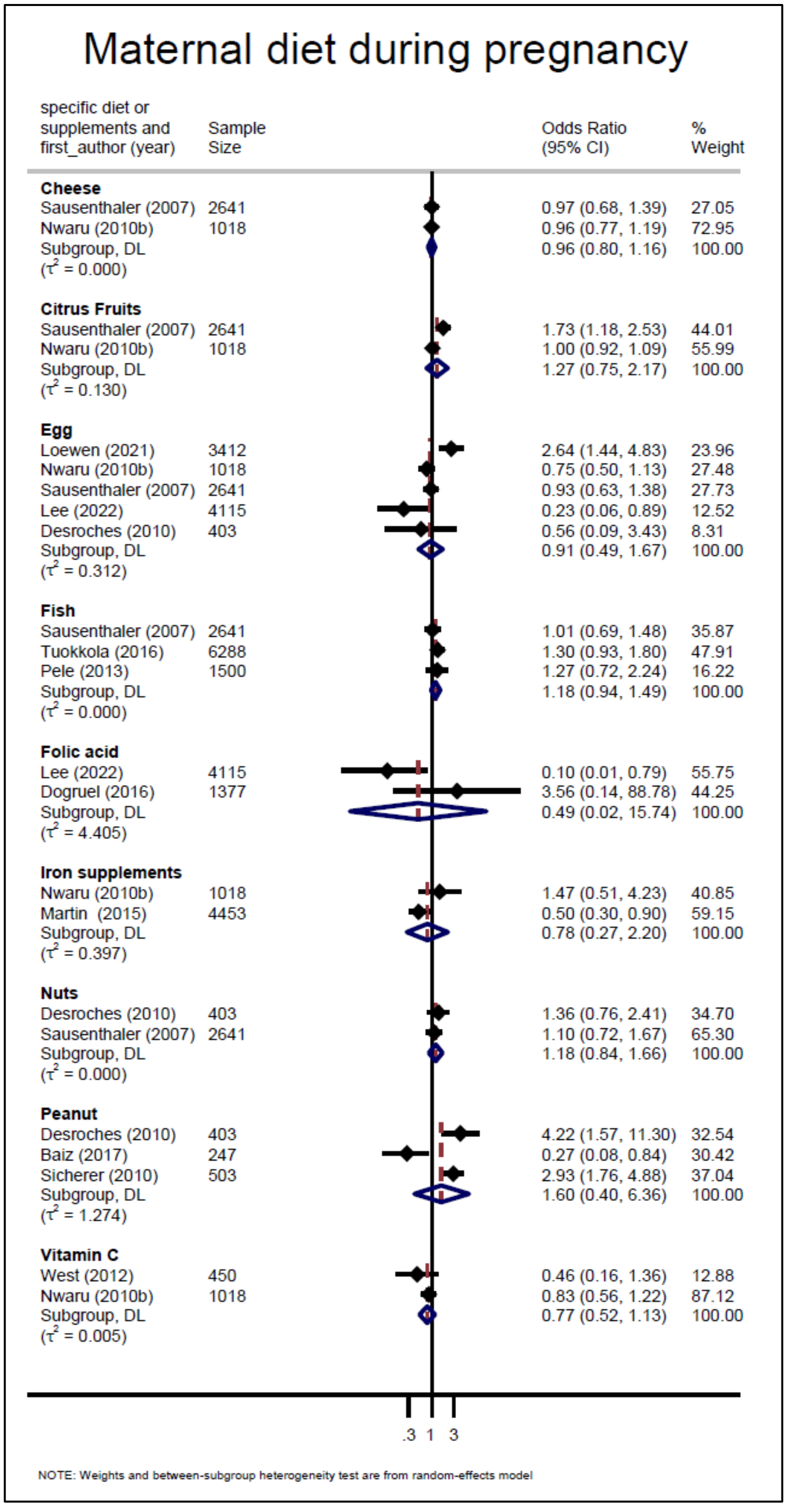

eFigure 28. Meta-Analysis of the Association Between Maternal Diet During Pregnancy and Development of Food Allergy in Children

© 2026 Islam N et al. *JAMA Pediatrics*.

(Binary predictor: cheese vs no cheese, citrus fruits vs no citrus fruits, egg vs no egg, fish vs no fish, folic acid vs no folic acid, iron supplements vs no iron supplements, nuts vs no nuts, peanut vs no peanut, vitamin c vs no vitamin c).

© 2026 Islam N et al. *JAMA Pediatrics*.

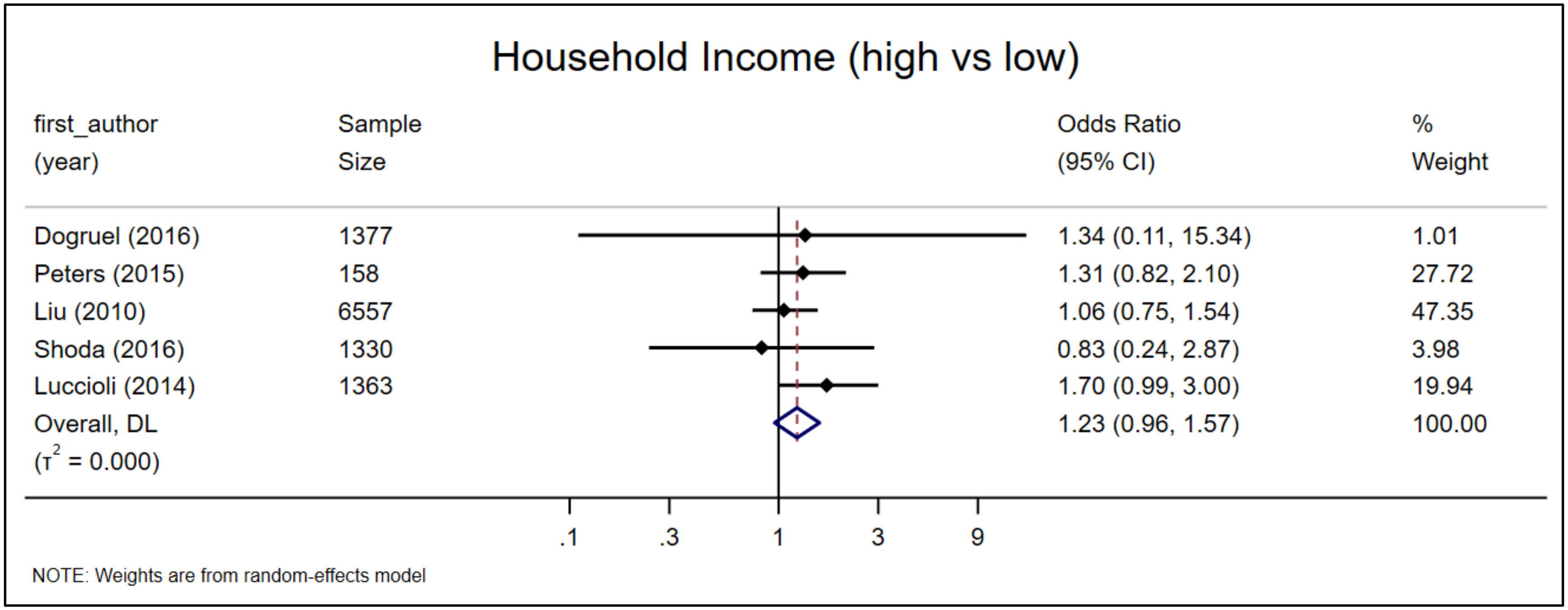

eFigure 29. Meta-Analysis of the Association Between Household Income and Development of Food Allergy in Children

(Binary predictor: high household income vs low household income).

© 2026 Islam N et al. *JAMA Pediatrics*.

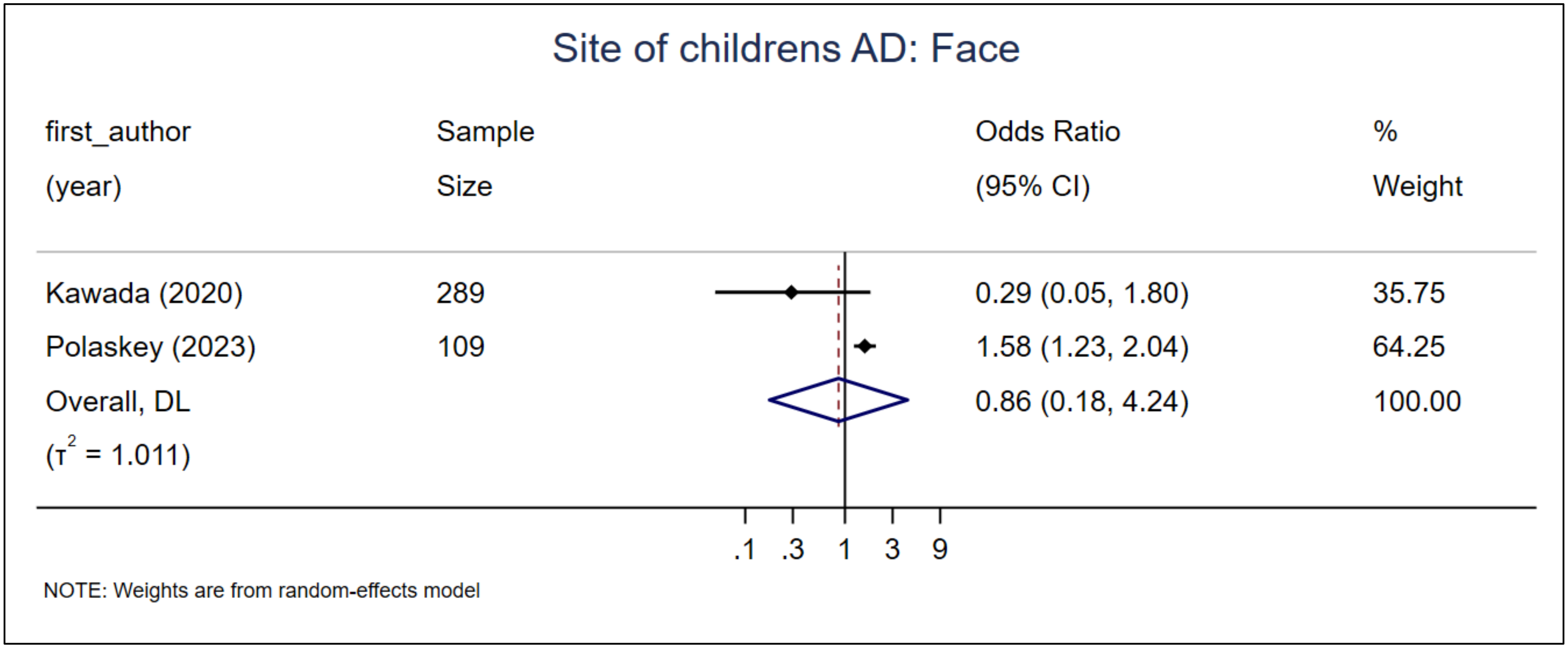

eFigure 30. Meta-Analysis of the Association Between the Site of Atopic Dermatitis in Children and Development of Food Allergy in Children

(Binary predictor: atopic dermatitis in the face vs no atopic dermatitis).

© 2026 Islam N et al. *JAMA Pediatrics*.

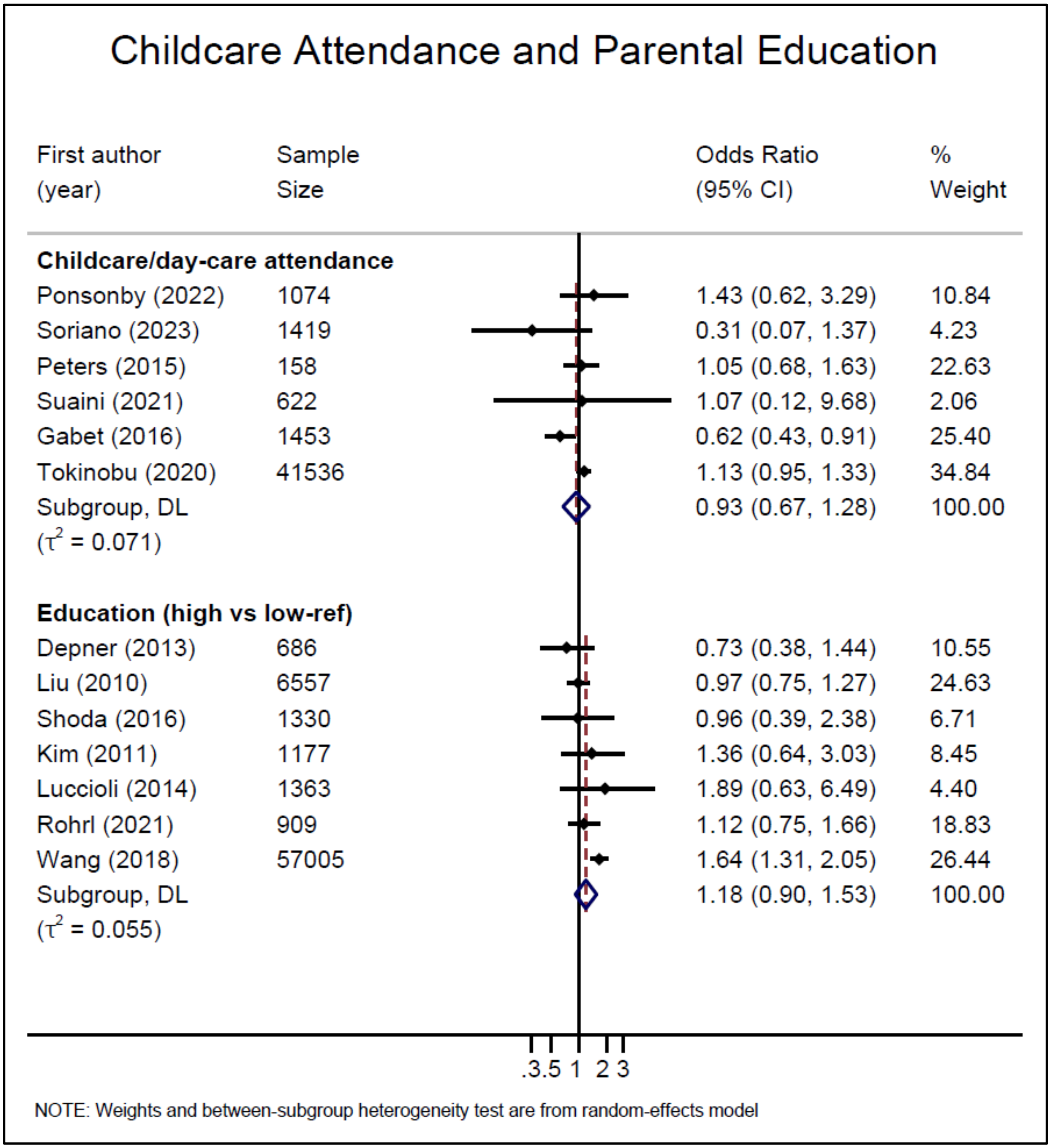

eFigure 31. Meta-Analysis of the Association Between Childcare/Day-Care Attendance, Parental Education and Development of Food Allergy in Children

(Binary predictor: childcare/day-care attendance vs no childcare/day-care attendance, high parental education vs low parental education).

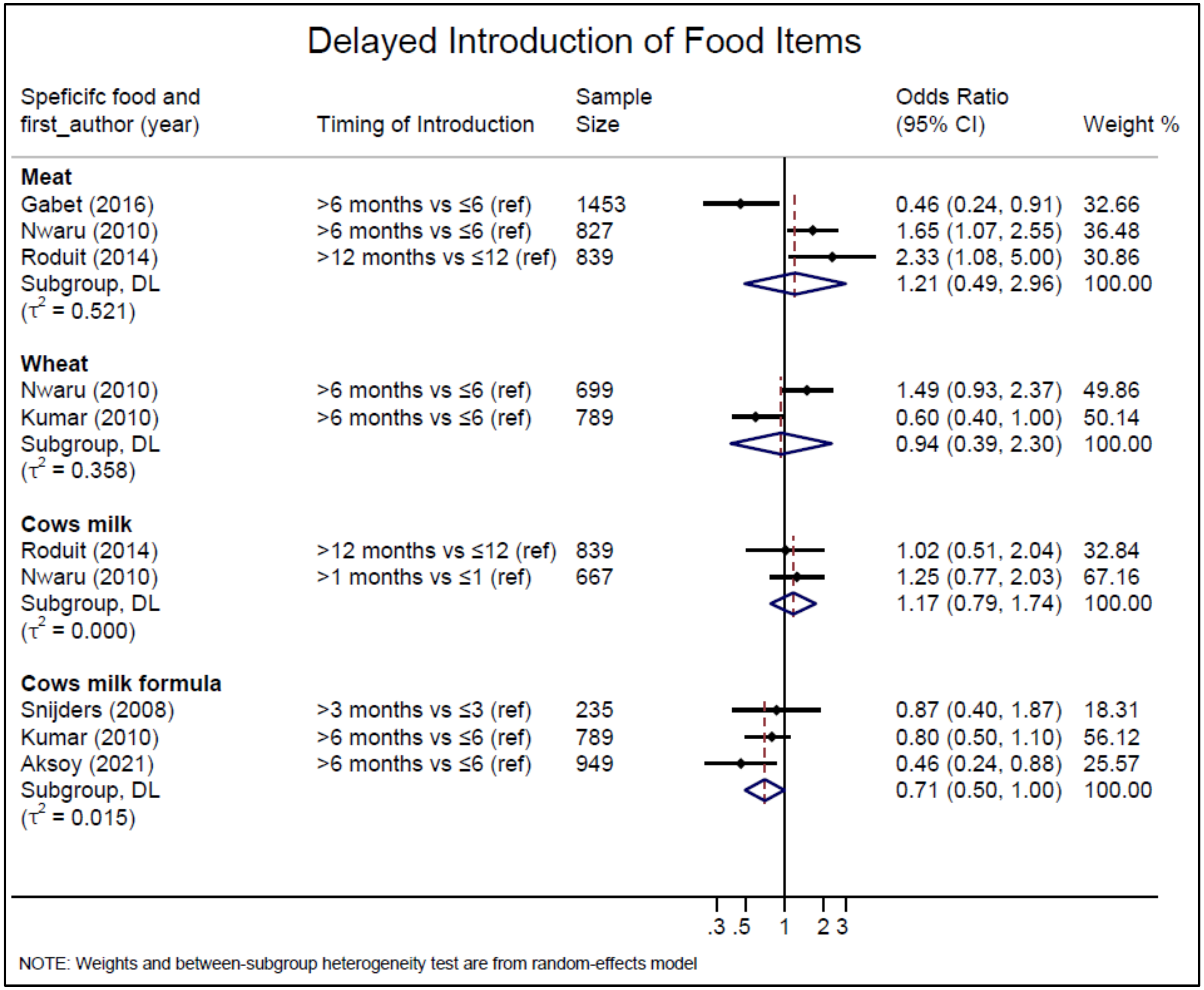

eFigure 32. Meta-Analysis of the Association Between Delayed Introduction of Food Items and Development of Food Allergy in Children

© 2026 Islam N et al. *JAMA Pediatrics*.

(Binary predictor: delayed introduction of cow's milk (>1 month) vs early introduction of cow's milk formula, delayed introduction of cow's milk formula (>3 months) vs early introduction of cow's milk formula, delayed introduction of wheat (>6 months) vs early introduction of wheat, delayed introduction of meat (>6 months) vs early introduction of meat).

© 2026 Islam N et al. *JAMA Pediatrics*.

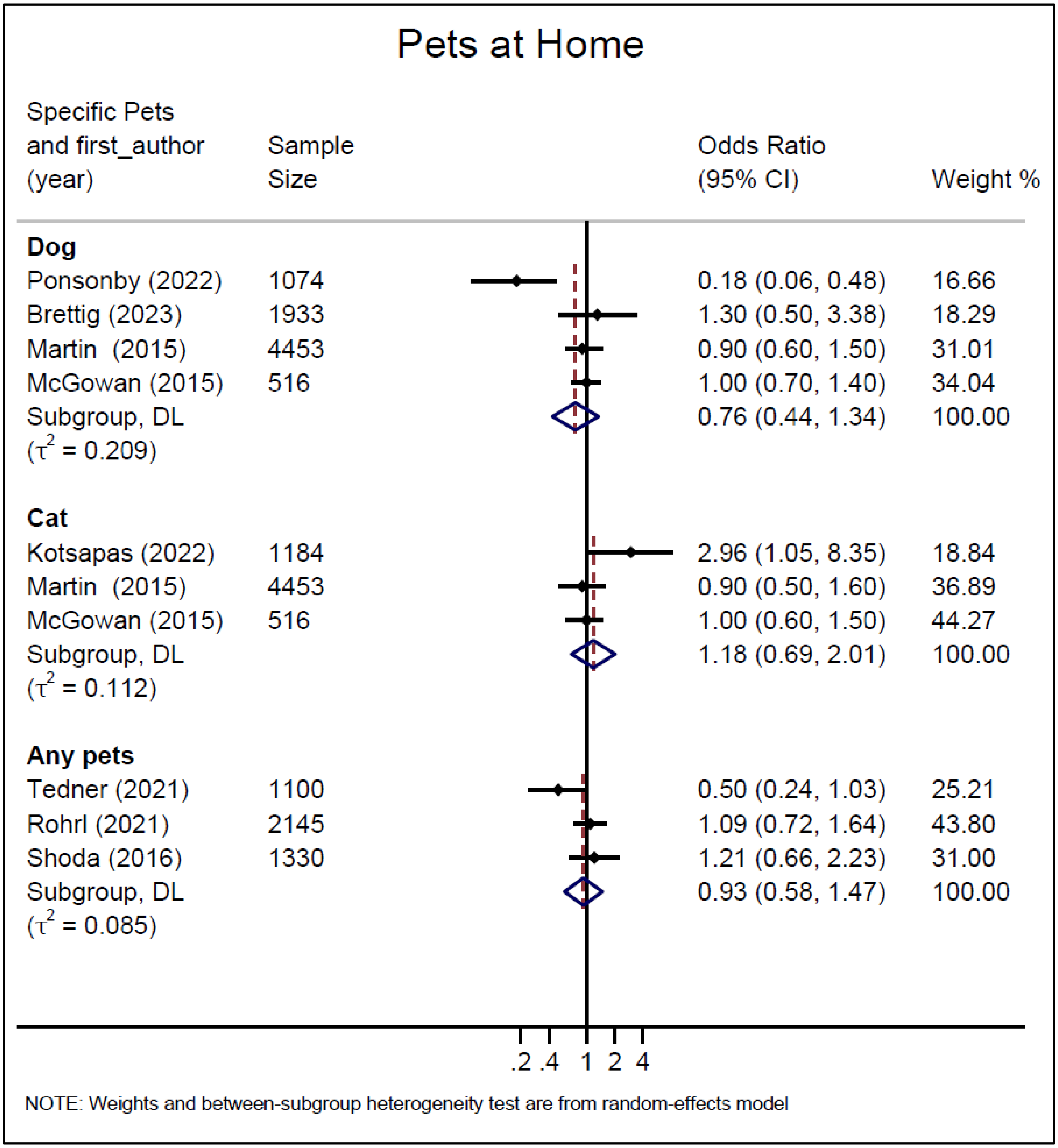

eFigure 33. Meta-Analysis of the Association Between Presence of Pets and Development of Food Allergy in Children

(Binary predictor: presence of cat vs no presence of cat at home, presence of dog at home vs no presence of dog at home, presence of any pets at home vs no presence of any pets at home).

© 2026 Islam N et al. *JAMA Pediatrics*.

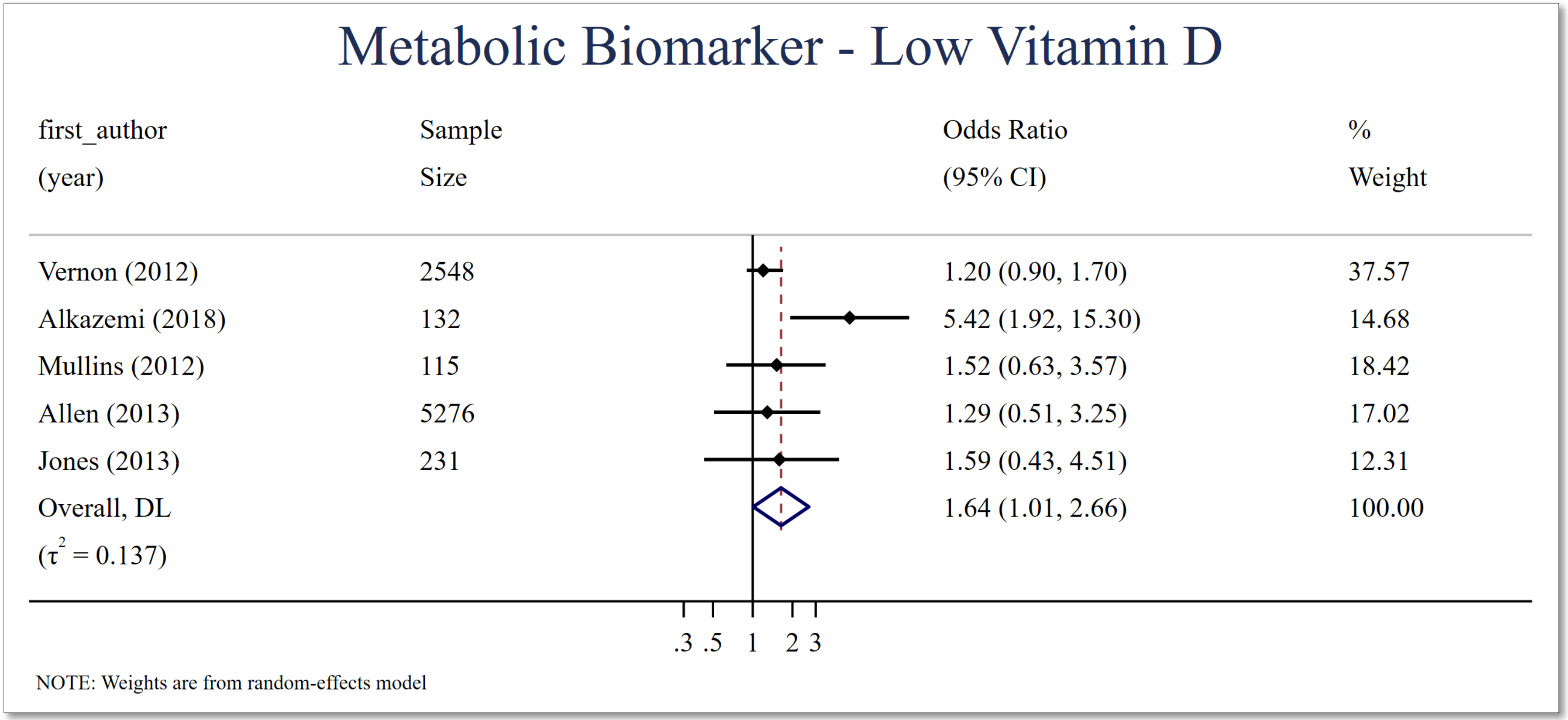

eFigure 34. Meta-Analysis of the Association Between Low Vitamin D in Children and Development of Food Allergy in Children

(Binary predictor: low Vitamin D [<50 nmol/L (<20 ng/mL)] vs high Vitamin D [≥75 nmol/L (≥30 ng/mL)] in children).

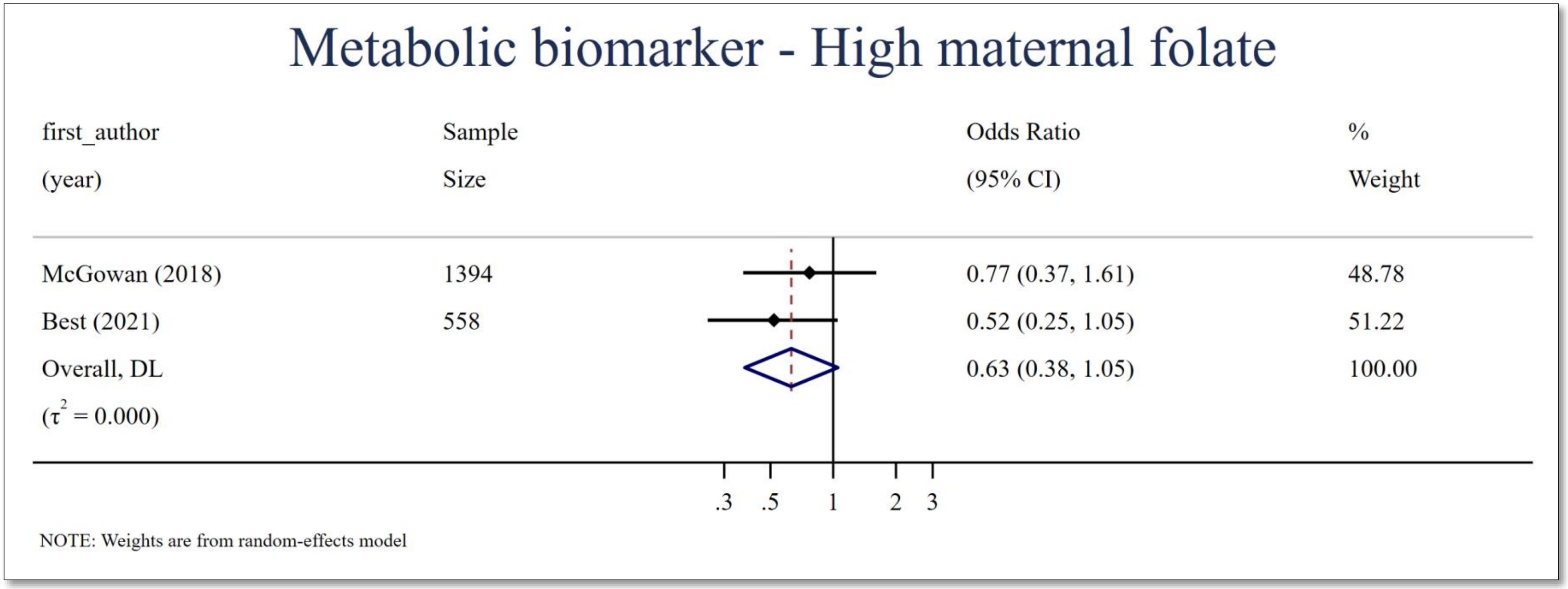

eFigure 35. Meta-Analysis of the Association Between High Maternal Folate and Development of Food Allergy in Children

(Binary predictor: high maternal folate [≥74.5 nmol/L] vs low maternal folate [<32.6 nmol/L]).

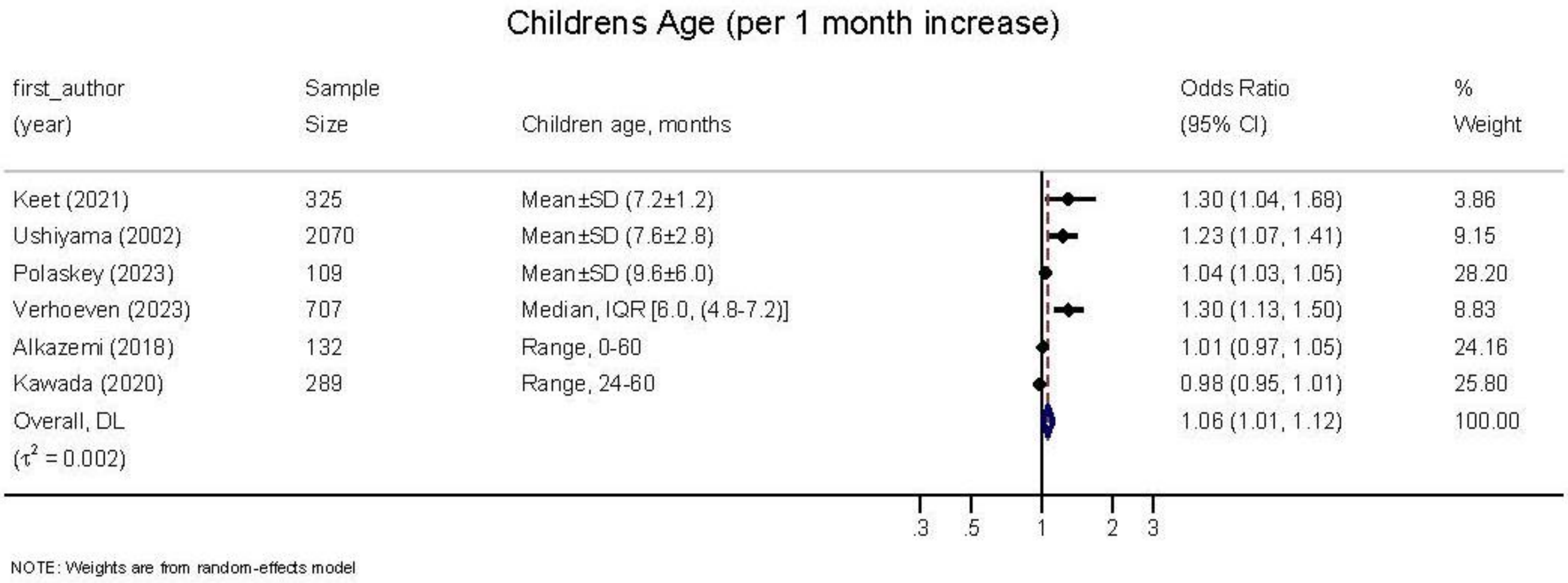

eFigure 36. Meta-Analysis of the Association Between Children's Age and Development of Food Allergy in Children

(Numerical predictor: children's age-per 1 month increase)

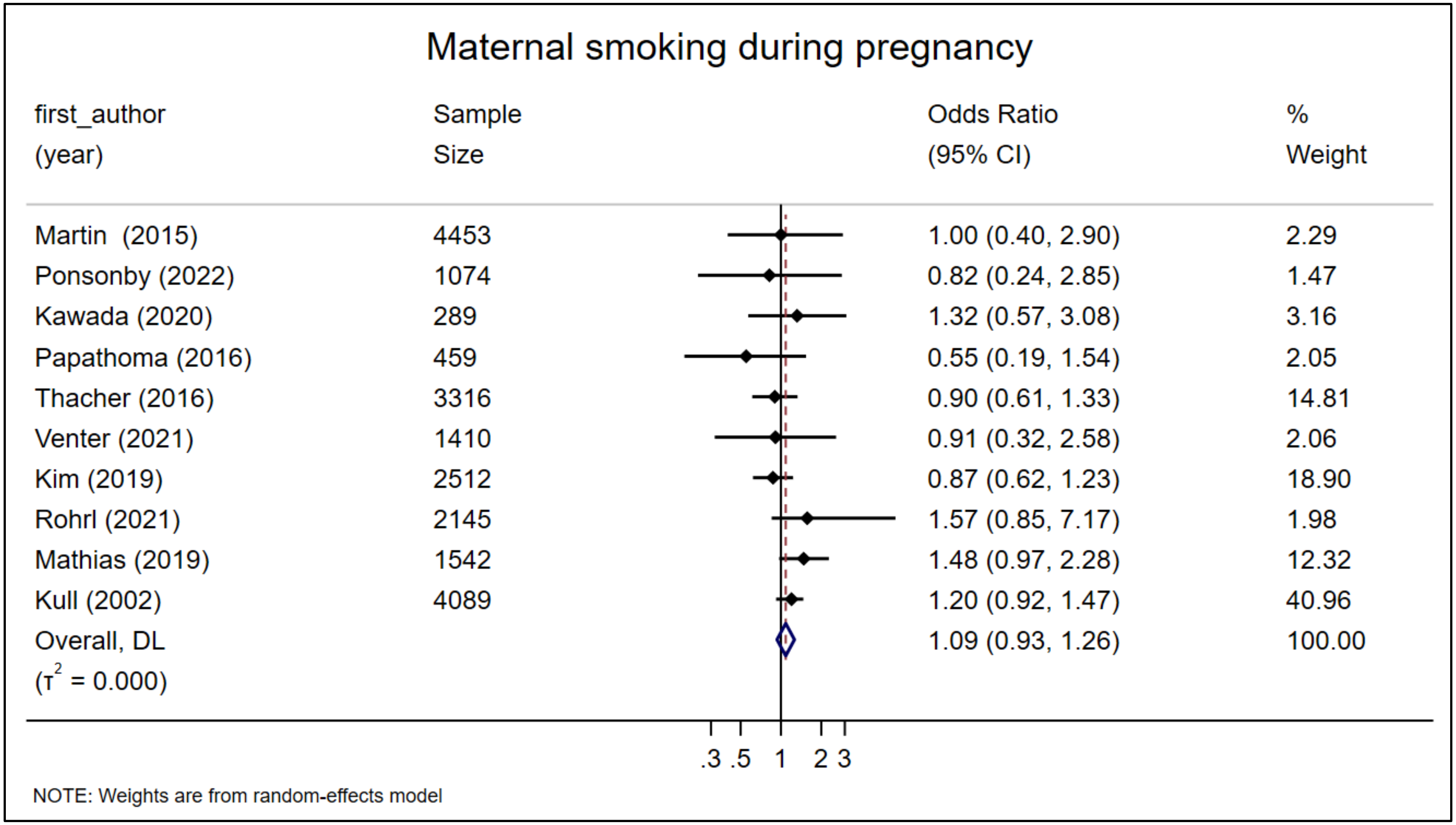

eFigure 37. Meta-Analysis of the Association Between Maternal Smoking During Pregnancy and Development of Food Allergy in Children

(Binary predictor: maternal smoking during pregnancy vs no maternal smoking during pregnancy).

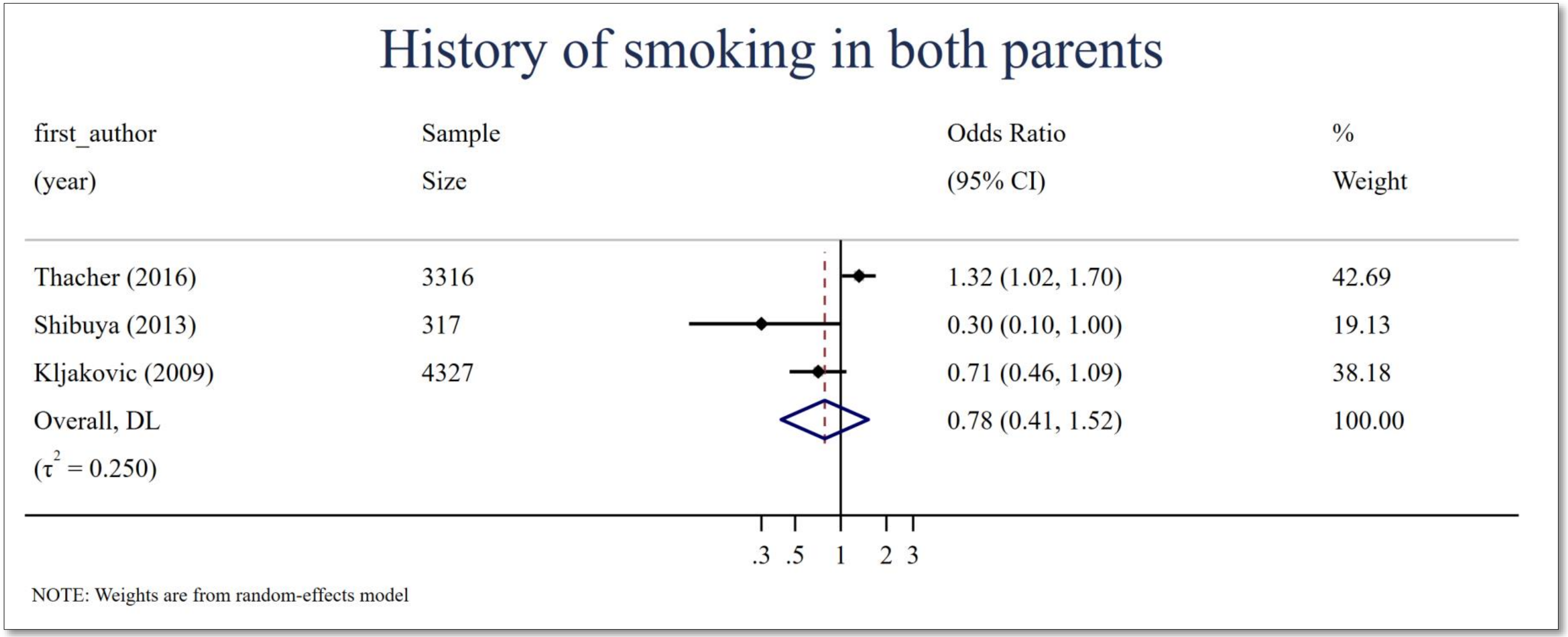

eFigure 38. Meta-Analysis of the Association Between History of Smoking in Both Parents and Development of Food Allergy in Children

(Binary predictor: History of smoking in both parents vs no history of smoking in both parents).

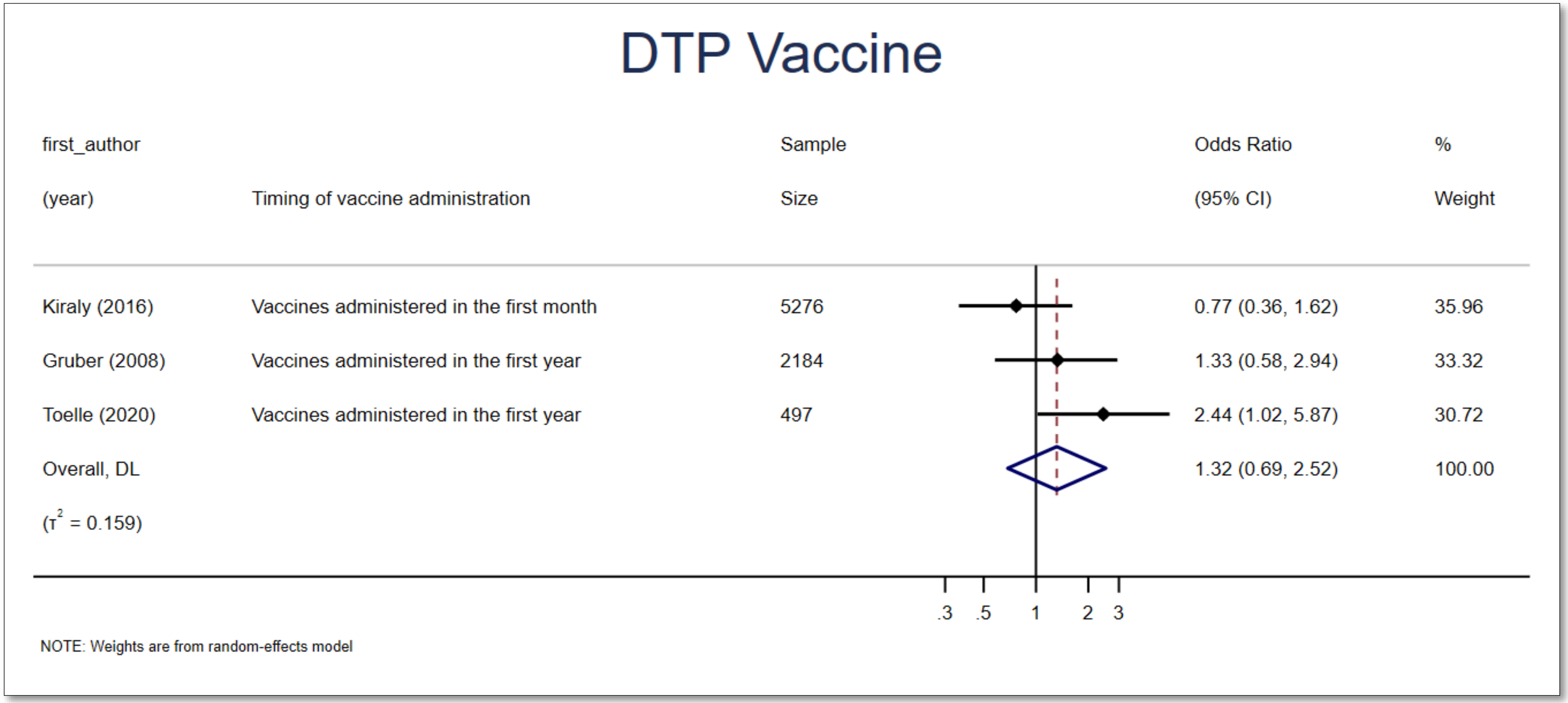

eFigure 39. Meta-Analysis of the Association Between Diphtheria-Tetanus-Pertussis (DTP) Vaccination and Development of Food Allergy in Children

(Binary predictor: diphtheria-tetanus-pertussis (DTP) vaccination vs no DTP administration).

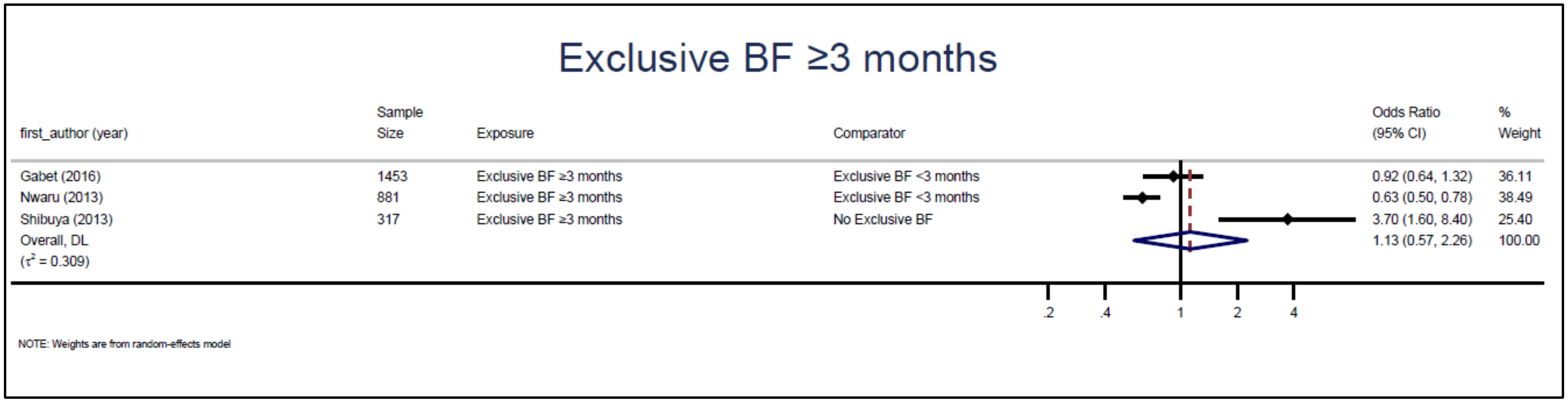

eFigure 40. Meta-Analysis of the Association Between Pattern and Duration of Breastfeeding and Development of Food Allergy in Children

(Binary predictor: exclusive breastfeeding (≥3 months) vs comparators).

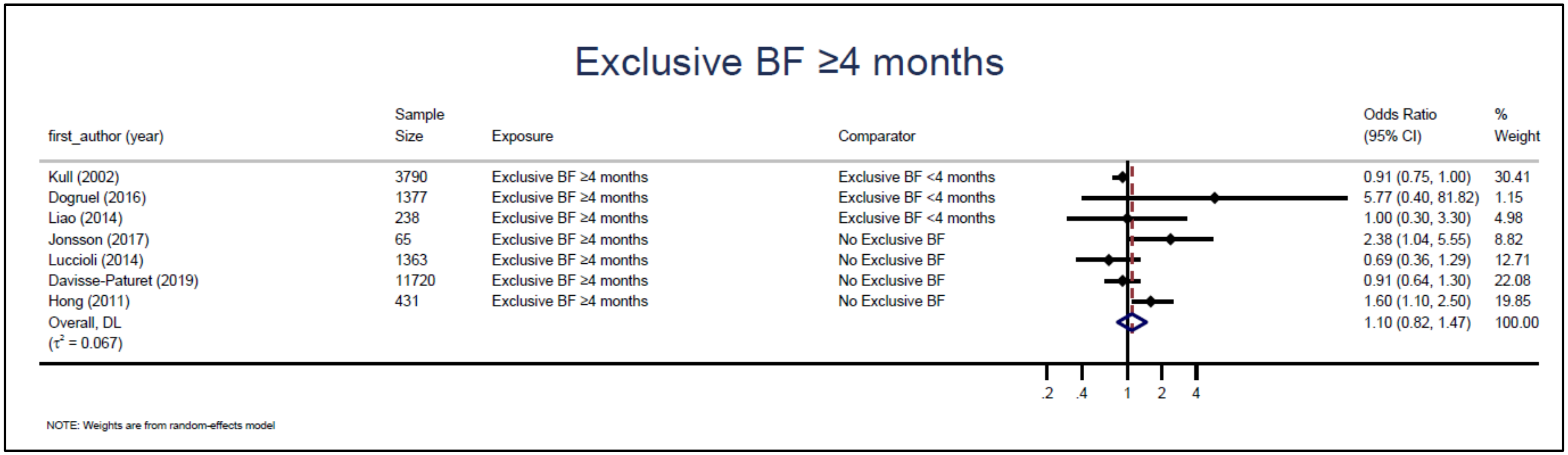

eFigure 41. Meta-Analysis of the Association Between Pattern and Duration of Breastfeeding and Development of Food Allergy in Children

(Binary predictor: exclusive breastfeeding (≥4 months) vs comparators).

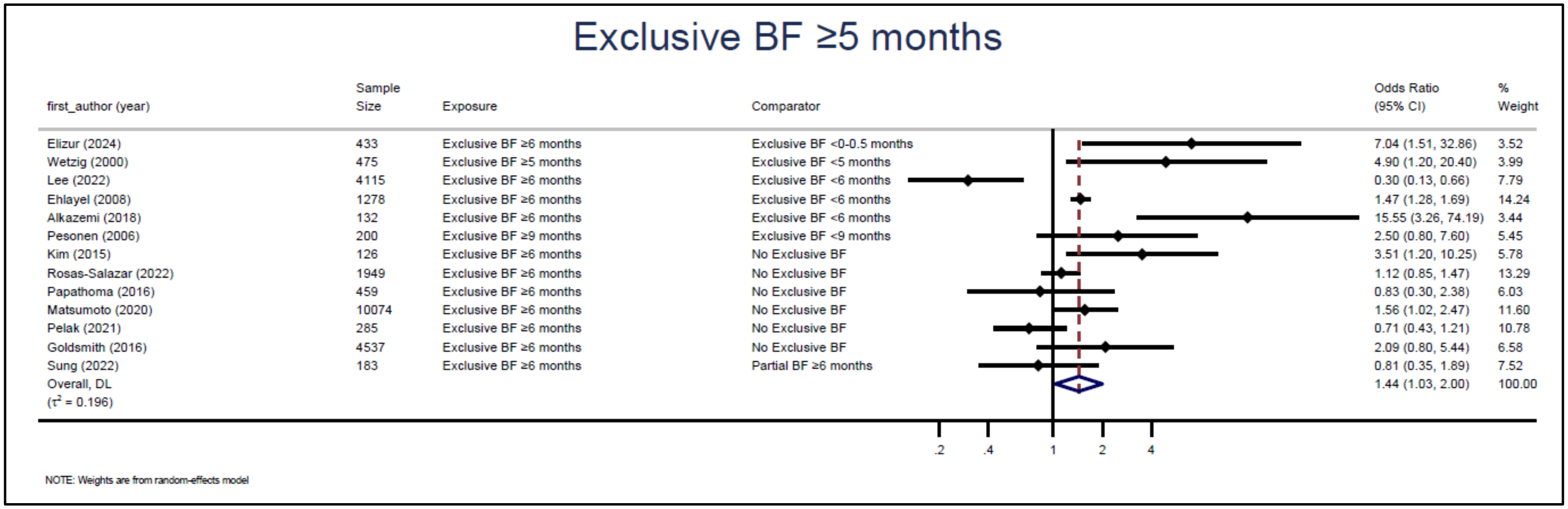

eFigure 42. Meta-Analysis of the Association Between Pattern and Duration of Breastfeeding and Development of Food Allergy in Children

(Binary predictor: exclusive breastfeeding (≥5 months) vs comparators).

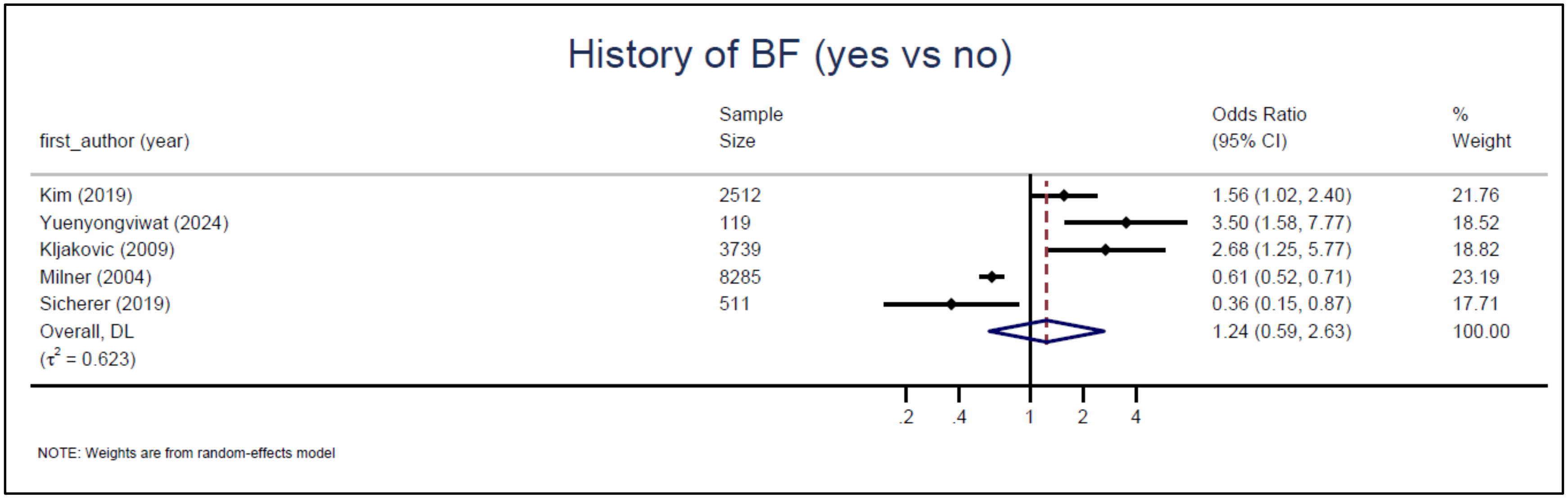

eFigure 43. Meta-Analysis of the Association Between History of Breastfeeding and Development of Food Allergy in Children

(Binary predictor: history of breastfeeding vs no history of breastfeeding).

eReferences.

1. Subbarao P, Anand SS, Becker AB, et al. The Canadian Healthy Infant Longitudinal Development (CHILD) Study: examining developmental origins of allergy and asthma. *Thorax* 2015; **70**(10): 998-1000.
2. Sears MR. The Canadian Healthy Infant Longitudinal Development (CHILD) study. *Can Respir J* 2010; **17**(Suppl A): 36A-7A.
3. Takaro TK, Scott JA, Allen RW, et al. The Canadian Healthy Infant Longitudinal Development (CHILD) birth cohort study: assessment of environmental exposures. *Journal of exposure science & environmental epidemiology* 2015; **25**(6): 580-92.
4. Tibshirani R. Regression shrinkage and selection via the lasso. *Journal of the Royal Statistical Society Series B: Statistical Methodology* 1996; **58**(1): 267-88.
5. Rubin DB. Multiple imputation. Flexible Imputation of Missing Data, Second Edition: Chapman and Hall/CRC; 2018: 29-62.
6. Azur MJ, Stuart EA, Frangakis C, Leaf PJ. Multiple imputation by chained equations: what is it and how does it work? *International journal of methods in psychiatric research* 2011; **20**(1): 40-9.
7. Iorio A, Spencer FA, Falavigna M, et al. Use of GRADE for assessment of evidence about prognosis: rating confidence in estimates of event rates in broad categories of patients. *bmj* 2015; **350**.
8. Chu DK, Golden DBK, Guyatt GH. Translating evidence to optimize patient care using GRADE. *The Journal of Allergy and Clinical Immunology: In Practice* 2021; **9**(12): 4221-30.
9. Schünemann HJ, Oxman AD, Higgins JPT, Vist GE, Glasziou P, Guyatt GH. Presenting results and 'Summary of findings' tables. *Cochrane handbook for systematic reviews of interventions: Cochrane book series* 2008: 335-57.
10. Guyatt GH, Oxman AD, Kunz R, et al. GRADE guidelines: 7. Rating the quality of evidence—inconsistency. *Journal of clinical epidemiology* 2011; **64**(12): 1294-302.
11. Guyatt G, Zhao Y, Mayer M, et al. GRADE guidance 36: updates to GRADE's approach to addressing inconsistency. *Journal of Clinical Epidemiology* 2023; **158**: 70-83.
12. Aksoy AG, Boran P, Karakoc-Aydiner E, et al. Prevalence of allergic disorders and risk factors associated with food allergy in Turkish preschoolers. *Allergologia Et Immunopathologia* 2021; **49**(1): 11-6.
13. Alkazemi D, Albeajan M, Kubow S. Early Infant Feeding Practices as Possible Risk Factors for Immunoglobulin E-Mediated Food Allergies in Kuwait. *International Journal of Pediatrics* 2018; **2018**: 1701903.
14. Allen K, Koplin J, Ponsonby AL, et al. Food allergy and anaphylaxis-2052. Vitamin D insufficiency is associated with challenge-proven food allergy in infants. *World Allergy Organization Journal* 2013; **6**(SUPPL. 1).
15. Arroyo A, Mehta G, Qi Y, et al. Maternal exposures to acid suppressant medications or antibiotics and infant food allergy in a bronchiolitis cohort. *Journal of Allergy and Clinical Immunology* 2023; **151**(2): AB173.
16. Ashley SE, Tan HT, Vuillermin P, et al. The skin barrier function gene SPINK 5 is associated with challenge-proven IgE-mediated food allergy in infants. *Allergy* 2017; **72**(9): 1356-64.
17. Azad MB, Konya T, Guttman DS, et al. Infant gut microbiota and food sensitization: associations in the first year of life. *Clinical & Experimental Allergy* 2015; **45**(3): 632-43.
18. Baiz N, Just J, Chastang J, Annesi-Maesano I. Earlier than early exposure to allergenic food and prevention of allergic sensitization in young children. *Revue Francaise d'Allergologie* 2017; **57**(5): 400-2.
19. Bedolla Barajas M, Alcala-Padilla G, Morales Romero J, Camacho Fregoso J, Rivera Mejia V. Peanut allergy in Mexican children: what is the effect of age at first consumption? *Iranian journal of allergy, asthma, and immunology* 2016; **15**(1): 53-61.
20. Bedolla-Barajas M, Morales-Romero J, Gaxiola-Arredondo BY, Alcalá-Padilla G, Romero-Velarde E. Prolonged breastfeeding and delayed introduction of whole cow's milk into the diet are factors associated with egg sensitization: A matched case–control study. *Allergologia et Immunopathologia* 2018; **46**(6): 539-45.
21. Best KP, Green TJ, Sulistyoningrum DC, et al. Maternal Late-Pregnancy Serum Unmetabolized Folic Acid Concentrations Are Not Associated with Infant Allergic Disease: A Prospective Cohort Study. *Journal of Nutrition* 2021; **151**(6): 1553-60.
22. Bock SA. Prospective appraisal of complaints of adverse reactions to foods in children during the first 3 years of life. *Pediatrics* 1987; **79**(5): 683-8.
23. Bouma F, Hoek G, Koppelman GH, et al. Exposure to ambient ultrafine particles and allergic sensitization in children up to 16 years. *Environ Res* 2023; **219**: 115102.

24. Brettig T, Soriano VX, Dharmage SC, et al. Cashew Allergy Prevalence and Sensitization in 1-Year-Old Infants. *J Allergy Clin Immunol Pract* 2023; **11**(11): 3478-84.e5.
25. Carlsten C, Dimich-Ward H, Ferguson A, et al. Atopic dermatitis in a high-risk cohort: natural history, associated allergic outcomes, and risk factors. *Annals of Allergy Asthma & Immunology* 2013; **110**(1): 24-8.
26. Chandran U, Demissie K, Echeverria SE, Long JB, Mizan S, Mino J. Food Allergy Among Low Birthweight Children in a National Survey. *Maternal and Child Health Journal* 2013; **17**(1): 165-71.
27. Chen Arroyo AJ, Robinson LB, Espinola JA, et al. Racial/ethnic differences in food sensitization and food allergy in a diverse multi-center cohort of U.S. infants. *Journal of Allergy and Clinical Immunology* 2019; **143**(2 Supplement): AB55.
28. Cho H, Kim J, Kim S, et al. Postpartum maternal anxiety affects the development of food allergy through dietary and gut microbial diversity during early infancy. *Allergy, Asthma & Immunology Research* 2024; **16**(2): 154.
29. Clausen M, Jonasson K, Keil T, Beyer K, Sigurdardottir ST. Fish oil in infancy protects against food allergy in Iceland-Results from a birth cohort study. *Allergy: European Journal of Allergy and Clinical Immunology* 2018; **73**(6): 1305-12.
30. Collier F, Ponsonby A-L, O'Hely M, et al. Naive regulatory T cells in infancy: Associations with perinatal factors and development of food allergy. *Allergy* 2019; **74**(9): 1760-8.
31. Davisse-Paturet C, Raherison C, Adel-Patient K, et al. Use of partially hydrolysed formula in infancy and incidence of eczema, respiratory symptoms or food allergies in toddlers from the ELFE cohort. *Pediatric Allergy and Immunology* 2019; **30**(6): 614-23.
32. Depner M, Ege MJ, Genuneit J, et al. Atopic sensitization in the first year of life. *Journal of Allergy and Clinical Immunology* 2013; **131**(3): 781-+.
33. Roduit C, Frei R, Depner M, et al. Increased food diversity in the first year of life is inversely associated with allergic diseases. *The Journal of allergy and clinical immunology* 2014; **133**(4): 1056-64.
34. von Mutius E, Schmid S. The PASTURE project: EU support for the improvement of knowledge about risk factors and preventive factors for atopy in Europe. *Allergy* 2006; **61**(4): 407-13.
35. Vuitton DA, Dalphin JC. Hygiène et allergie: les micro-organismes des fermes sont-ils protecteurs? *Journal de Mycologie Médicale* 2006; **16**(4): 220-38.
36. Frei R, Ferstl R, Roduit C, et al. Exposure to nonmicrobial N-glycolylneuraminic acid protects farmers' children against airway inflammation and colitis. *Journal of Allergy and Clinical Immunology* 2018; **141**(1): 382-90.e7.
37. Loss G, Bitter S, Wohlgensinger J, et al. Prenatal and early-life exposures alter expression of innate immunity genes: The PASTURE cohort study. *Journal of Allergy and Clinical Immunology* 2012; **130**(2): 523-30.e9.
38. Chauveau A, Dalphin ML, Mauny F, et al. Skin prick tests and specific IgE in 10-year-old children: Agreement and association with allergic diseases. *Allergy* 2017; **72**(9): 1365-73.
39. Roduit C, Frei R, Depner M, et al. An increased diversity of food introduced in the first year of life as a protective factor for allergic diseases. *European Respiratory Journal* 2013; **42**(SUPPL. 57).
40. Depner M, Taft DH, Kirjavainen PV, et al. Maturation of the gut microbiome during the first year of life contributes to the protective farm effect on childhood asthma. *Nature Medicine* 2020; **26**(11): 1766-75.
41. Pfefferle PI, Sel S, Ege MJ, et al. Cord blood allergen-specific IgE is associated with reduced IFN-γ production by cord blood cells: The Protection against Allergy—Study in Rural Environments (PASTURE) study. *Journal of Allergy and Clinical Immunology* 2008; **122**(4): 711-6.
42. Hose AJ, Pagani G, Karvonen AM, et al. Excessive Unbalanced Meat Consumption in the First Year of Life Increases Asthma Risk in the PASTURE and LUKAS2 Birth Cohorts. *Frontiers in Immunology* 2021; **12**.
43. Roduit C, Frei R, Depner M, et al. Phenotypes of Atopic Dermatitis Depending on the Timing of Onset and Progression in Childhood. *Jama Pediatrics* 2017; **171**(7): 655-62.
44. Nicklaus S, Divaret-Chauveau A, Chardon M-L, et al. The protective effect of cheese consumption at 18 months on allergic diseases in the first 6 years. *ALLERGY* 2019; **74**(4): 788-98.
45. Vuitton DA, Divaret-Chauveau A, Dalphin ML, Laplante JJ, von Mutius E, Dalphin JC. Protection contre l'allergie par l'environnement de la ferme : en 15 ans, qu'avons-nous appris de la cohorte européenne « PASTURE » ? *Bulletin de l'Académie Nationale de Médecine* 2019; **203**(7): 618-30.
46. Chauveau A, Dalphin M-L, Kaulek V, et al. Disagreement between Skin Prick Tests and Specific IgE in Early Childhood. *International Archives of Allergy and Immunology* 2016; **170**(2): 69-74.

47. Orivuori L, Mustonen K, Roduit C, et al. Immunoglobulin A and immunoglobulin G antibodies against β-lactoglobulin and gliadin at age 1 associate with immunoglobulin E sensitization at age 6. *Pediatric Allergy and Immunology* 2014; **25**(4): 329-37.
48. Desroches A, Infante-Rivard C, Paradis L, Paradis J, Haddad E. Peanut allergy: Is maternal transmission of antigens during pregnancy and breastfeeding a risk factor. *Journal of Investigational Allergology and Clinical Immunology* 2010; **20**(4): 289-94.
49. Dhudasia MB, Spergel JM, Puopolo KM, et al. Intrapartum Group B Streptococcal Prophylaxis and Childhood Allergic Disorders. *Pediatrics* 2021; **147**(5).
50. Doğruel D, Bingöl G, Altıntaş DU, Yılmaz M, Güneşer Kendirli S. Clinical features of food allergy during the 1st year of life: the ADAPAR birth cohort study. *International archives of allergy and immunology* 2016; **169**(3): 171-80.
51. Du Toit G, Roberts G, Sayre PH, et al. Identifying infants at high risk of peanut allergy: the Learning Early About Peanut Allergy (LEAP) screening study. *The Journal of allergy and clinical immunology* 2013; **131**(1): 135-12.
52. Ehlayel MS, Bener A. Duration of breast-feeding and the risk of childhood allergic diseases in a developing country. 2008.
53. Elizur A, Rachel-Jossefi S, Rachmiel M, Eisenberg E, Katz Y. Consumption of cow's milk formula in the nursery and the development of milk allergy. *Clinical and Translational Allergy* 2024; **14**(4): e12352.
54. Flohr C, Perkin M, Logan K, et al. Atopic dermatitis and disease severity are the main risk factors for food sensitization in exclusively breastfed infants. *The Journal of investigative dermatology* 2014; **134**(2): 345-50.
55. Fox AT, Sasieni P, du Toit G, Syed H, Lack G. Household peanut consumption as a risk factor for the development of peanut allergy. *Journal of Allergy and Clinical Immunology* 2009; **123**(2): 417-23.
56. Gabet S, Just J, Couderc R, Seta N, Momas I. Allergic sensitisation in early childhood: Patterns and related factors in PARIS birth cohort. *International Journal of Hygiene and Environmental Health* 2016; **219**(8): 792-800.
57. Gao X, Yan Y, Zeng G, et al. Influence of prenatal and early-life exposures on food allergy and eczema in infancy: a birth cohort study. *BMC Pediatr* 2019; **19**(1): 239.
58. Gao Y, Stokholm J, O'Hely M, et al. Gut microbiota maturity mediates the protective effect of siblings on food allergy. *Journal of Allergy and Clinical Immunology* 2023; **152**(3): 667-75.
59. Goldsmith AJ, Koplin JJ, Lowe AJ, et al. Formula and breast feeding in infant food allergy: A population-based study. *Journal of paediatrics and child health* 2016; **52**(4): 377-84.
60. Grimshaw KEC, Bryant T, Oliver EM, et al. Incidence and risk factors for food hypersensitivity in UK infants: results from a birth cohort study. *Clinical and translational allergy* 2015; **6**(101576043): 1.
61. Grimshaw KEC, Bryant T, Oliver EM, et al. Incidence and risk factors for food hypersensitivity in UK infants: Results from a birth cohort study. *Clinical and Translational Allergy* 2016; **6**(1): 1.
62. Grimshaw KEC, Roberts G, Selby A, et al. Risk Factors for Hen's Egg Allergy in Europe: EuroPrevall Birth Cohort. *The journal of allergy and clinical immunology In practice* 2020; **8**(4): 1341-8.e5.
63. Gruber C, Warner J, Hill D, Bauchau V, Group ES. Early atopic disease and early childhood immunization--is there a link? *Allergy* 2008; **63**(11): 1464-72.
64. Halpern SR, Sellars WA, Johnson RB, Anderson DW, Saperstein S, Reisch JS. DEVELOPMENT OF CHILDHOOD ALLERGY IN INFANTS FED BREAST, SOY, OR COW MILK. *Journal of Allergy and Clinical Immunology* 1973; **51**(3): 139-51.
65. Hesselmar B, Saalman R, Rudin A, Adlerberth I, Wold AE. Early fish introduction is associated with less eczema, but not sensitization, in infants. *Acta Paediatrica* 2010; **99**(12): 1861-7.
66. Hong X, Wang G, Liu X, et al. Gene polymorphisms, breast-feeding, and development of food sensitization in early childhood. *The Journal of allergy and clinical immunology* 2011; **128**(2): 374-81.e2.
67. Banks JR, Andrews T. Gene polymorphisms, breastfeeding, and development of food sensitization in early childhood. *Pediatrics* 2012; **130**(SUPPL.1): S8.
68. Hong X, Nadeau K, Wang G, et al. Metabolomic profiles during early childhood and risk of food allergies and asthma in multiethnic children from a prospective birth cohort. *Journal of Allergy and Clinical Immunology* 2024.
69. Hurley S, Franklin R, McCallion N, et al. Allergy related outcomes at 12 months in the CORAL birth cohort of Irish children born during the first COVID 19 lockdown. *Authorea Preprints* 2022.
70. Jones AP, Palmer D, Zhang G, Prescott S. Allergic diseases of the skin and drug allergies-2006. Cord blood 25-hydroxyvitamin D3 and allergic disease during infancy. *World Allergy Organization Journal* 2013; **6**(SUPPL. 1).

71. Jonsson K, Barman M, Brekke HK, et al. Late introduction of fish and eggs is associated with increased risk of allergy development - results from the FARMFLORA birth cohort. *Food & Nutrition Research* 2017; **61**(1): 1393306.
72. Joseph CLM, Ownby DR, Havstad SL, et al. Early complementary feeding and risk of food sensitization in a birth cohort. *The Journal of allergy and clinical immunology* 2011; **127**(5): 1203-10.e5.
73. Joseph CLM, Zoratti EM, Ownby DR, et al. Exploring racial differences in IgE-mediated food allergy in the WHEALS birth cohort. *Annals of allergy, asthma & immunology : official publication of the American College of Allergy, Asthma, & Immunology* 2016; **116**(3): 219-24.e1.
74. Dowhower Karpa K, Paul IM, Leckie JA, et al. A retrospective chart review to identify perinatal factors associated with food allergies. *Nutrition journal* 2012; **11**(101152213): 87.
75. Kalb B, Marenholz I, Jeanrenaud ACSN, et al. Filaggrin loss-of-function mutations are associated with persistence of egg and milk allergy. *Journal of Allergy and Clinical Immunology* 2022; **150**(5): 1125-34.
76. Kawada S, Futamura M, Hashimoto H, et al. Association between sites and severity of eczema and the onset of cow's milk and egg allergy in children. *PLoS ONE* 2020; **15**(10 October): e0240980.
77. Keet CA, Wood RA, Matsui EC. Personal and parental nativity as risk factors for food sensitization. *The Journal of allergy and clinical immunology* 2012; **129**(1): 169-5.
78. Keet C, Pistiner M, Plesa M, et al. Age and eczema severity, but not family history, are major risk factors for peanut allergy in infancy. *Journal of Allergy and Clinical Immunology* 2021; **147**(3): 984-+.
79. Kelderer F, Mogren I, Eriksson C, Silfverdal SA, Domellöf M, West CE. Associations between pre-and postnatal antibiotic exposures and early allergic outcomes: A population-based birth cohort study. *Pediatric Allergy and Immunology* 2022; **33**(9): e13848.
80. Keski-Nisula L, Karvonen A, Pfefferle PI, Renz H, Büchele G, Pekkanen J. Birth-related factors and doctor-diagnosed wheezing and allergic sensitization in early childhood. *Allergy* 2010; **65**(9): 1116-25.
81. Kikkawa T, Yorifuji T, Fujii Y, et al. Birth order and paediatric allergic disease: A nationwide longitudinal survey. *Clinical and Experimental Allergy* 2018; **48**(5): 577-85.
82. Kim JS, Arguelles LM, Kumar R, Pongracic JA, Wang X. Protective effect of early fresh fruit ingestion on the development of food allergy. *Journal of Allergy and Clinical Immunology* 2009; **123**(2 SUPPL. 1): S268.
83. Kim J, Chang E, Han Y, Ahn K, Lee S-I. The incidence and risk factors of immediate type food allergy during the first year of life in Korean infants: a birth cohort study. *Pediatric Allergy and Immunology* 2011; **22**(7): 715-9.
84. Kim NY, Kim GR, Kim JH, et al. Food allergen sensitization in young children with typical signs and symptoms of immediate-type food allergies: a comparison between monosensitized and polysensitized children. *Korean journal of pediatrics* 2015; **58**(9): 330.
85. Kim YH, Kim KW, Lee S-Y, et al. Maternal Perinatal Dietary Patterns Affect Food Allergy Development in Susceptible Infants. *Journal of Allergy and Clinical Immunology-in Practice* 2019; **7**(7): 2337-+.
86. Kiraly N, Koplin JJ, Crawford NW, et al. Timing of routine infant vaccinations and risk of food allergy and eczema at one year of age. *Allergy* 2016; **71**(4): 541-9.
87. Kjaer HF, Eller E, Høst A, Andersen KE, Bindslev-Jensen C. The prevalence of allergic diseases in an unselected group of 6-year-old children. The DARC birth cohort study. *Pediatric allergy and immunology* 2008; **19**(8): 737-45.
88. Kljakovic M, Gatenby P, Hawkins C, et al. The parent-reported prevalence and management of peanut and nut allergy in school children in the Australian Capital Territory. *Journal of Paediatrics and Child Health* 2009; **45**(3): 98-103.
89. Kojima R, Shinohara R, Kushima M, et al. Infantile peanut introduction and peanut allergy in regions with a low prevalence of peanut allergy: the Japan Environment and Children's Study (JECS). *Journal of Epidemiology* 2024; **34**(7): 324-30.
90. Koksal ZG, Uysal P, Mercan A, Bese SA, Erge D. Does maternal fermented dairy products consumption protect against cow's milk protein allergy in toddlers? *Annals of allergy, asthma & immunology : official publication of the American College of Allergy, Asthma, & Immunology* 2023; **130**(3): 333-9.
91. Koplin JJ, Dharmage SC, Ponsonby AL, et al. Environmental and demographic risk factors for egg allergy in a population-based study of infants. *Allergy* 2012; **67**(11): 1415-22.
92. Koplin JJ, Peters RL, Ponsonby AL, et al. Increased risk of peanut allergy in infants of Asian-born parents compared to those of Australian-born parents. *Allergy* 2014; **69**(12): 1639-47.
93. Kotsapas C, Nicolaou N, Haider S, et al. Early-life predictors and risk factors of peanut allergy, and its association with asthma in later-life: Population-based birth cohort study. *Clinical & Experimental Allergy* 2022.

94. Kull I, Wickman M, Lilja G, Nordvall SL, Pershagen G. Breast feeding and allergic diseases in infants - a prospective birth cohort study. *Archives of Disease in Childhood* 2002; **87**(6): 478-81.
95. Kumar R, Ouyang F, Story RE, et al. Gestational diabetes, atopic dermatitis, and allergen sensitization in early childhood. *Journal of Allergy and Clinical Immunology* 2009; **124**(5): 1031-8.
96. Kumar R, Caruso DM, Arguelles L, et al. Early life eczema, food introduction, and risk of food allergy in children. *Pediatric, Allergy, Immunology, and Pulmonology* 2010; **23**(3): 175-82.
97. Kuwabara Y, Nii R, Tanaka K, Ishii E, Nagao M, Fujisawa T. Season of birth is associated with increased risk of atopic dermatitis in Japanese infants: A retrospective cohort study. *Allergy, Asthma and Clinical Immunology* 2020; **16**(1): 44.
98. Lamminsalo A, Metsälä J, Takkinen H-M, et al. Maternal energy-adjusted fatty acid intake during pregnancy and the development of cows' milk allergy in the offspring. *British Journal of Nutrition* 2022; **128**(8): 1607-14.
99. Landau T, Vakulenko-Lagun B, Brandwein M. The protective effect of moderate maternal peanut consumption on peanut sensitization and allergy. *Annals of Allergy, Asthma & Immunology* 2023; **131**(2): 231-8.
100. Laubereau B, Filipiak-Pittroff B, von Berg A, et al. Caesarean section and gastrointestinal symptoms, atopic dermatitis, and sensitisation during the first year of life. *Archives of disease in childhood* 2004; **89**(11): 993-7.
101. Lee AJ, Tham EH, Goh AE-N, et al. Prevalence of IgE-mediated cow milk, egg, and peanut allergy in young Singapore children. *Asia Pacific Allergy* 2022; **12**(3): e31.
102. Lee-Sarwar KA, Chen YC, Lasky-Su J, et al. Early-life fecal metabolomics of food allergy. *Allergy* 2023; **78**(2): 512-21.
103. Leung TF, Yung E, Wong YS, Lam CWK, Wong GWK. Parent-reported adverse food reactions in Hong Kong Chinese pre-schoolers: epidemiology, clinical spectrum and risk factors. *Pediatric Allergy and Immunology* 2009; **20**(4): 339-46.
104. Levin ME, Botha M, Basera W, et al. Environmental factors associated with allergy in urban and rural children from the South African Food Allergy (SAFFA) cohort. *Journal of Allergy and Clinical Immunology* 2020; **145**(1): 415-26.
105. Liao S-L, Lai S-H, Yeh K-W, et al. Exclusive breastfeeding is associated with reduced cow's milk sensitization in early childhood. *Pediatric allergy and immunology : official publication of the European Society of Pediatric Allergy and Immunology* 2014; **25**(5): 456-61.
106. Huang JL, Chiu CY, Yeh KW. Early-onset eczema is associated with increased milk sensitization and risk of rhinitis and asthma in early childhood. *Journal of Allergy and Clinical Immunology* 2018; **141**(2 Supplement 1): AB5.
107. Liem JJ, Kozyrskyj AL, Huq SI, Becker AB. The risk of developing food allergy in premature or low-birth-weight children. *Journal of allergy and clinical immunology* 2007; **119**(5): 1203-9.
108. Liu AH, Jaramillo R, Sicherer SH, et al. National prevalence and risk factors for food allergy and relationship to asthma: results from the National Health and Nutrition Examination Survey 2005-2006. *The Journal of allergy and clinical immunology* 2010; **126**(4): 798-806.e13.
109. Liu X, Wang G, Hong X, et al. Gene-vitamin D interactions on food sensitization: a prospective birth cohort study. *Allergy* 2011; **66**(11): 1442-8.
110. Liu X, Arguelles L, Zhou Y, et al. Longitudinal trajectory of vitamin D status from birth to early childhood in the development of food sensitization. *Pediatric research* 2013; **74**(3): 321-6.
111. Loo EXL, Sim JZT, Goh A, et al. Predictors of allergen sensitization in Singapore children from birth to 3 years. *Allergy Asthma and Clinical Immunology* 2016; **12**: 56.
112. Lopez DJ, Lodge CJ, Bui DS, et al. Air pollution is associated with persistent peanut allergy in the first 10 years. *Journal of Allergy and Clinical Immunology* 2024; **154**(6): 1489-99.
113. Love BL, Mann JR, Hardin JW, Lu ZK, Cox C, Amrol DJ. Antibiotic prescription and food allergy in young children. *Allergy Asthma and Clinical Immunology* 2016; **12**: 41.
114. Lowe AJ, Abramson MJ, Hosking CS, et al. The temporal sequence of allergic sensitization and onset of infantile eczema. *Clinical and experimental allergy : journal of the British Society for Allergy and Clinical Immunology* 2007; **37**(4): 536-42.
115. Loewen K, Moraes TJ, Turvey SE, et al. Prenatal egg consumption and infant sensitization and allergy to egg, peanut, and cow's milk in the CHILD Cohort. *The Journal of Allergy and Clinical Immunology: In Practice* 2021; **9**(5): 2109-12.
116. Luccioli S, Zhang Y, Verrill L, Ramos-Valle M, Kwegyir-Afful E. Infant Feeding Practices and Reported Food Allergies at 6 Years of Age. *Pediatrics* 2014; **134**: S21-S8.

117. Ma Z, Chen L, Xian R, Fang H, Wang J, Hu Y. Time trends of childhood food allergy in China: Three cross-sectional surveys in 1999, 2009, and 2019. *Pediatric Allergy and Immunology* 2021; **32**(5): 1073-9.
118. Mai XM, Kull I, Wickman M, Bergstrom A. Antibiotic use in early life and development of allergic diseases: respiratory infection as the explanation. *Clinical and Experimental Allergy* 2010; **40**(8): 1230-7.
119. Martin PE, Eckert JK, Koplin JJ, et al. Which infants with eczema are at risk of food allergy? Results from a population-based cohort. *Clinical and experimental allergy : journal of the British Society for Allergy and Clinical Immunology* 2015; **45**(1): 255-64.
120. Martone GM, Lehman HK, Rideout TC, et al. Delayed egg introduction beyond infancy and increased egg allergy risk in childhood. *Journal of paediatrics and child health* 2023; **59**(1): 53-7.
121. Mathias JG, Zhang H, Soto-Ramirez N, Karmaus W. The association of infant feeding patterns with food allergy symptoms and food allergy in early childhood. *International Breastfeeding Journal* 2019; **14**(1): 43.
122. Matsumoto N, Yorifuji T, Nakamura K, Ikeda M, Tsukahara H, Doi H. Breastfeeding and risk of food allergy: A nationwide birth cohort in Japan. *Allergology International* 2020; **69**(1): 91-7.
123. Marenholz I, Grosche S, Rüschendorf F, et al. Evaluation of food allergy candidate loci in the Genetics of Food Allergy study. *Journal of Allergy and Clinical Immunology* 2018; **142**(4): 1368-70.
124. McGowan EC, Bloomberg GR, Gergen PJ, et al. Influence of early-life exposures on food sensitization and food allergy in an inner-city birth cohort. *The Journal of allergy and clinical immunology* 2015; **135**(1): 171-8.
125. McGowan EC, Hong X, Selhub J, et al. The association between folate/folic acid metabolites and the development of food allergy (FA) in Children. *Journal of Allergy and Clinical Immunology* 2018; **141**(2 Supplement 1): AB86.
126. McGowan EC, Hong X, Selhub J, et al. Association Between Folate Metabolites and the Development of Food Allergy in Children. *The journal of allergy and clinical immunology In practice* 2020; **8**(1): 132-40.e5.
127. McMilin CR, Grant C, Morton SMB, Camargo Jr C. Risk factors for childhood peanut allergy in a large birth cohort study: Growing up in New Zealand. *Journal of Allergy and Clinical Immunology* 2016; **137**(2 SUPPL. 1): AB395.
128. Metsala J, Lundqvist A, Kaila M, Gissler M, Klaukka T, Virtanen SM. Maternal and Perinatal Characteristics and the Risk of Cow's Milk Allergy in Infants up to 2 Years of Age: A Case-Control Study Nested in the Finnish Population. *American Journal of Epidemiology* 2010; **171**(12): 1310-6.
129. Metsala J, Lundqvist A, Virta LJ, Kaila M, Gissler M, Virtanen SM. Mother's and Offspring's Use of Antibiotics and Infant Allergy to Cow's Milk. *Epidemiology* 2013; **24**(2): 303-9.
130. Metzler S, Frei R, Schmaußer-Hechfellner E, et al. Association between antibiotic treatment during pregnancy and infancy and the development of allergic diseases. *Pediatric Allergy and Immunology* 2019; **30**(4): 423-33.
131. Miliku K, Robertson B, Sharma AK, et al. Human milk oligosaccharide profiles and food sensitization among infants in the CHILD Study. *ALLERGY* 2018; **73**(10): 2070-3.
132. Miliku K, Richelle J, Becker AB, et al. Sex-specific associations of human milk long-chain polyunsaturated fatty acids and infant allergic conditions. *Pediatric Allergy and Immunology* 2021; **32**(6): 1173-82.
133. Milner JD, Stein DM, McCarter R, Moon RY. Early infant multivitamin supplementation is associated with increased risk for food allergy and asthma. *Pediatrics* 2004; **114**(1): 27-32.
134. Mitre E, Susi A, Kropp LE, Schwartz DJ, Gorman GH, Nylund CM. Association Between Use of Acid-Suppressive Medications and Antibiotics During Infancy and Allergic Diseases in Early Childhood. *JAMA PEDIATRICS* 2018; **172**(6).
135. Mitselou N, Hallberg J, Stephansson O, Almqvist C, Melen E, Ludvigsson JF. Cesarean delivery, preterm birth, and risk of food allergy: Nationwide Swedish cohort study of more than 1 million children. *Journal of Allergy and Clinical Immunology* 2018; **142**(5): 1510-+.
136. Mitselou N, Andersson N, Bergström A, et al. Preterm birth reduces the risk of IgE sensitization up to early adulthood: A population-based birth cohort study. *Allergy* 2022; **77**(5): 1570-82.
137. Molloy J, Collier F, Saffery R, et al. Folate levels in pregnancy and offspring food allergy and eczema. *Pediatric allergy and immunology : official publication of the European Society of Pediatric Allergy and Immunology* 2020; **31**(1): 38-46.
138. Mullins RJ, Clark S, Wiley V, Eyles D, Camargo CA, Jr. Neonatal vitamin D status and childhood peanut allergy: a pilot study. *Annals of allergy, asthma & immunology : official publication of the American College of Allergy, Asthma, & Immunology* 2012; **109**(5): 324-8.

139. Negele K, Heinrich J, Borte M, et al. Mode of delivery and development of atopic disease during the first 2 years of life. *Pediatric allergy and immunology : official publication of the European Society of Pediatric Allergy and Immunology* 2004; **15**(1): 48-54.
140. Nwaru BI, Erkkola M, Ahonen S, et al. Age at the introduction of solid foods during the first year and allergic sensitization at age 5 years. *Pediatrics* 2010; **125**(1): 50-9.
141. Nwaru BI, Ahonen S, Kaila M, et al. Maternal diet during pregnancy and allergic sensitization in the offspring by 5 yrs of age: a prospective cohort study. *Pediatric allergy and immunology : official publication of the European Society of Pediatric Allergy and Immunology* 2010; **21**(1 Pt 1): 29-37.
142. Nwaru BI, Erkkola M, Ahonen S, et al. Maternal diet during lactation and allergic sensitization in the offspring at age of 5. *Pediatric Allergy and Immunology* 2011; **22**(3): 334-41.
143. Nwaru BI, Takkinen HM, Niemela O, et al. Introduction of complementary foods in infancy and atopic sensitization at the age of 5 years: timing and food diversity in a Finnish birth cohort. *Allergy* 2013; **68**(4): 507-16.
144. Panjari M, Koplin JJ, Dharmage SC, et al. Nut allergy prevalence and differences between Asian-born children and Australian-born children of Asian descent: a state-wide survey of children at primary school entry in Victoria, Australia. *Clinical and Experimental Allergy* 2016; **46**(4): 602-9.
145. Papathoma E, Triga M, Fouzas S, Dimitriou G. Cesarean section delivery and development of food allergy and atopic dermatitis in early childhood. *Pediatric Allergy and Immunology* 2016; **27**(4): 419-24.
146. Pelak G, Wiese AM, Maskarinec JM, Phillips WL, Keim SA. Infant Feeding Practices During the First Postnatal Year and Risk of Asthma and Allergic Disease During the First 6 Years of Life. *Breastfeeding Medicine* 2021; **16**(7): 539-46.
147. Pele F, Bajeux E, Gendron H, et al. Maternal fish and shellfish consumption and wheeze, eczema and food allergy at age two: a prospective cohort study in Brittany, France. *Environmental Health* 2013; **12**: 102.
148. Chacón GP, Fathima P, Jones M, et al. Infant whole-cell versus acellular pertussis vaccination in 1997 to 1999 and risk of childhood hospitalization for food-induced anaphylaxis: linked administrative databases cohort study. *The Journal of Allergy and Clinical Immunology: In Practice* 2024; **12**(3): 670-80.
149. Perkin MR, Logan K, Marrs T, et al. Association of frequent moisturizer use in early infancy with the development of food allergy. *Journal of Allergy and Clinical Immunology* 2021; **147**(3): 967-76.
150. Pesonen M, Kallio MJT, Ranki A, Siimes MA. Prolonged exclusive breastfeeding is associated with increased atopic dermatitis: a prospective follow-up study of unselected healthy newborns from birth to age 20 years. *Clinical & Experimental Allergy* 2006; **36**(8): 1011-8.
151. Peters RL, Allen KJ, Dharmage SC, et al. Differential factors associated with challenge-proven food allergy phenotypes in a population cohort of infants: a latent class analysis. *Clinical and experimental allergy : journal of the British Society for Allergy and Clinical Immunology* 2015; **45**(5): 953-63.
152. Polaskey MT, Bendelow A, Mukherji J, Buranosky B, Silverberg JI, Fishbein A. Facial atopic dermatitis is associated with sensitization to cow's milk, egg whites, and peanuts in children under 36 months. *Pediatric dermatology* 2023; **40**(3): 497-9.
153. Ponsonby AL, Collier F, O'Hely M, et al. Household size, T regulatory cell development, and early allergic disease: a birth cohort study. *Pediatric Allergy and Immunology* 2022; **33**(6): e13810.
154. Pretorius RA, McKinnon E, Palmer DJ. Higher maternal bread and thiamine intakes are associated with increased infant allergic disease. *Pediatric Allergy and Immunology* 2024; **35**(9): e14237.
155. Pyrhonen K, Hiltunen L, Kaila M, Nayha S, Laara E. Heredity of food allergies in an unselected child population: an epidemiological survey from Finland. *Pediatric allergy and immunology : official publication of the European Society of Pediatric Allergy and Immunology* 2011; **22**(1 Pt 2): e124-32.
156. Pyrhonen K, Nayha S, Hiltunen L, Laara E. Caesarean section and allergic manifestations: insufficient evidence of association found in population-based study of children aged 1 to 4 years. *Acta paediatrica (Oslo, Norway : 1992)* 2013; **102**(10): 982-9.
157. Pyrhonen K, Kulmala P. Occurrence of pollen season at the end of the first trimester predicts clinical atopic diseases in the offspring: A Finnish population-based study. *International Journal of Hygiene and Environmental Health* 2020; **225**: 113452.
158. Röhrl DV, Brandstetter S, Siziba LP, et al. Food allergy in infants assessed in two German birth cohorts 10 years after the EuroPrevall Study. *Pediatric Allergy and Immunology* 2021; **n/a**(n/a).
159. Rosas-Salazar C, Shilts MH, Tang Z-Z, et al. Exclusive breast-feeding, the early-life microbiome and immune response, and common childhood respiratory illnesses. *Journal of Allergy and Clinical Immunology* 2022; **150**(3): 612-21.

160. Ruohomäki A, Toffol E, Airaksinen V, et al. The impact of postpartum depressive symptoms on self-reported infant health and analgesic consumption at the age of 12 months: A prospective cohort study. *Journal of Psychiatric Research* 2021; **136**: 388-97.
161. Saarinen, Savilahti. Infant feeding patterns affect the subsequent immunological features in cow's milk allergy. *Clinical & Experimental Allergy* 2000; **30**(3): 400-6.
162. Sakihara T, Sugiura S, Ito K. The ingestion of cow's milk formula in the first 3 months of life prevents the development of cow's milk allergy. *Asia Pacific Allergy* 2016; **6**(4): 207-12.
163. Sánchez-Valverde F, Gil F, Martinez D, et al. The impact of caesarean delivery and type of feeding on cow's milk allergy in infants and subsequent development of allergic march in childhood. *Allergy* 2009; **64**(6): 884-9.
164. Sato N, Yamaide F, Nakano T, Yonekura S, Okamoto Y, Shimojo N. Association of umbilical cord serum TARC/CCL17 with childhood allergies: A birth cohort study. *Allergology International* 2023; **72**(4): 551-6.
165. Sausenthaler S, Koletzko S, Schaaf B, et al. Maternal diet during pregnancy in relation to eczema and allergic sensitization in the offspring at 2 y of age. *American Journal of Clinical Nutrition* 2007; **85**(2): 530-7.
166. Savage JH, Matsui EC, Wood RA, Keet CA. Urinary levels of triclosan and parabens are associated with aeroallergen and food sensitization. *The Journal of allergy and clinical immunology* 2012; **130**(2): 453-60.e7.
167. Sbihi H, Allen RW, Becker A, et al. Perinatal Exposure to Traffic-Related Air Pollution and Atopy at 1 Year of Age in a Multi-Center Canadian Birth Cohort Study. *Environmental Health Perspectives* 2015; **123**(9): 902-8.
168. Seay HL, Martin VM, Virkud YV, Marget M, Shreffler WG, Yuan Q. Prospective associations between acid suppressive therapy and food allergy in early childhood. *Clinical and experimental allergy: journal of the British Society for Allergy and Clinical Immunology* 2022.
169. Shek LP-C, Cabrera-Morales EA, Soh SE, et al. A population-based questionnaire survey on the prevalence of peanut, tree nut, and shellfish allergy in 2 Asian populations. *Journal of Allergy and Clinical Immunology* 2010; **126**(2): 324-U50.
170. Shibuya N, Saito E, Karasawa C. [Dermatitis in early infancy as a risk factor for sensitization and allergic diseases during the first year of life]. *Arerugi = [Allergy]* 2013; **62**(12): 1598-610.
171. Shoda T, Futamura M, Yang L, et al. Timing of eczema onset and risk of food allergy at 3 years of age: A hospital-based prospective birth cohort study. *Journal of Dermatological Science* 2016; **84**(2): 144-8.
172. Sicherer SH, Wood RA, Stablein D, et al. Maternal consumption of peanut during pregnancy is associated with peanut sensitization in atopic infants. *The Journal of allergy and clinical immunology* 2010; **126**(6): 1191-7.
173. Sicherer SH, Wood RA, Perry TT, et al. Clinical factors associated with peanut allergy in a high-risk infant cohort. *Allergy* 2019; **74**(11): 2199-211.
174. Sidorchuk A, Wickman M, Pershagen G, Lagarde F, Linde A. Cytomegalovirus infection and development of allergic diseases in early childhood: Interaction with EBV infection? *Journal of Allergy and Clinical Immunology* 2004; **114**(6): 1434-40.
175. Simons E, Balshaw R, Lefebvre DL, et al. Timing of Introduction, Sensitization, and Allergy to Highly Allergenic Foods at Age 3 Years in a General-Population Canadian Cohort. *Journal of Allergy and Clinical Immunology: In Practice* 2020; **8**(1): 166.
176. Smejda K, Polanska K, Merecz-Kot D, et al. Maternal stress during pregnancy and allergic diseases in children during the first year of life. *Respiratory care* 2018; **63**(1): 70-6.
177. Snijders BEP, Thijs C, van Ree R, van den Brandt PA. Age at first introduction of cow milk products and other food products in relation to infant atopic manifestations in the first 2 years of life: The KOALA birth cohort study. *Pediatrics* 2008; **122**(1): E115-E22.
178. Soriano VX, Koplin JJ, Forrester M, et al. Infant pacifier sanitization and risk of challenge- proven food allergy: A cohort study. *Journal of Allergy and Clinical Immunology* 2021; **147**(5): 1823-+.
179. Soriano VX, Lee HY, Dharmage SC, Perrett KP, Peters RL, Koplin JJ. Prevalence and risk factors of cow's milk sensitization and allergy in southeast Australia. *The Journal of Allergy and Clinical Immunology: In Practice* 2023; **11**(11): 3541-3.
180. Stokholm J, Sevelsted A, Anderson UD, Bisgaard H. Preeclampsia Associates with Asthma, Allergy, and Eczema in Childhood. *American journal of respiratory and critical care medicine* 2017; **195**(5): 614-21.
181. Suaini NHA, Loo EX-L, Peters RL, et al. Children of Asian ethnicity in Australia have higher risk of food allergy and early-onset eczema than those in Singapore. *ALLERGY* 2021.
182. Suaini NHA, Koh QY, Toh JY, et al. Maternal and Infant Dietary Patterns Are Not Related to Food Allergy Risk in Singapore Children: GUSTO Cohort Study. *The Journal of nutrition* 2024.

183. Sugiura S, Hiramitsu Y, Futamura M, et al. Development of a prediction model for infants at high risk of food allergy. *Asia Pacific Allergy* 2021; **11**(1): e5.
184. Sung W-H, Yeh K-W, Huang J-L, et al. Longitudinal changes in body mass index Z-scores during infancy and risk of childhood allergies. *Journal of Microbiology, Immunology and Infection* 2022; **55**(5): 956-64.
185. Tan H-TT, Ellis JA, Koplin JJ, et al. Filaggrin loss-of-function mutations do not predict food allergy over and above the risk of food sensitization among infants. *Journal of Allergy and Clinical Immunology* 2012; **130**(5): 1211-3.
186. Tanaka K, Matsui T, Sato A, et al. The relationship between the season of birth and early-onset food allergies in children. *Pediatric Allergy and Immunology* 2015; **26**(7): 607-13.
187. Tanaka K, Okubo H, Sasaki S, Arakawa M, Miyake Y. Maternal caffeine intake during pregnancy and risk of food allergy in young Japanese children. *Journal of Paediatrics and Child Health* 2021; **57**(6): 903-7.
188. Tedner SG, Soderhall C, Konradsen JR, et al. Extract and molecular-based early infant sensitization and associated factors-A PreventADALL study. *ALLERGY* 2021; **76**(9): 2730-9.
189. Thacher JD, Gruzieva O, Pershagen G, et al. Parental smoking and development of allergic sensitization from birth to adolescence. *ALLERGY* 2016; **71**(2): 239-48.
190. Thijs C, Muller A, Rist L, et al. Fatty acids in breast milk and development of atopic eczema and allergic sensitisation in infancy. *Allergy: European Journal of Allergy and Clinical Immunology* 2011; **66**(1): 58-67.
191. Toelle BG, Garden FL, McIntyre PB, Wood N, Marks GB. Pertussis vaccination and allergic illness in Australian children. *Pediatric Allergy and Immunology* 2020; **31**(7): 857-61.
192. Tokinobu A, Yorifuji T, Yamakawa M, Tsuda T, Doi H. Association of early daycare attendance with allergic disorders in children: a longitudinal national survey in Japan. *Archives of Environmental & Occupational Health* 2020; **75**(1): 18-26.
193. Tuokkola J, Luukkainen P, Tapanainen H, et al. Maternal diet during pregnancy and lactation and cow's milk allergy in offspring. *European Journal of Clinical Nutrition* 2016; **70**(5): 554-9.
194. Tuokkola J, Lamminsalo A, Metsala J, et al. Maternal antioxidant intake during pregnancy and the development of cows' milk allergy in the offspring. *British Journal of Nutrition* 2021; **125**(12): 1386-93.
195. Ushiyama Y, Matsumoto K, Shinohara M, et al. Nutrition during pregnancy may be associated with allergic diseases in infants. *Journal of Nutritional Science and Vitaminology* 2002; **48**(5): 345-51.
196. van den Berg ME, Flokstra-de Blok BMJ, Vlieg-Boerstra BJ, et al. Parental Eczema Increases the Risk of Double-Blind, Placebo-Controlled Reactions to Milk but Not to Egg, Peanut or Hazelnut. *International Archives of Allergy and Immunology* 2011; **158**(1): 77-83.
197. van Nimwegen FA, Penders J, Stobberingh EE, et al. Mode and place of delivery, gastrointestinal microbiota, and their influence on asthma and atopy. *Journal of Allergy and Clinical Immunology* 2011; **128**(5): 948-U371.
198. Venero-Fernández SJ, Bringues-Menzie V, Méndez-Rotger MT, et al. Prevalencia, incidencia y factores asociados con reacción adversa a alimentos en infantes cubanos. Estudio de cohorte de base poblacional. *Revista alergia México* 2018; **65**(2): 117-27.
199. Venter C, Pereira B, Voigt K, et al. Factors associated with maternal dietary intake, feeding and weaning practices, and the development of food hypersensitivity in the infant. *Pediatric allergy and immunology : official publication of the European Society of Pediatric Allergy and Immunology* 2009; **20**(4): 320-7.
200. Venter C, Maslin K, Dean T, Arshad SH. Does concurrent breastfeeding alongside the introduction of solid food prevent the development of food allergy? *Journal of Nutritional Science* 2016; **5**((Venter, Maslin, Dean) School of Health Sciences and Social Work, University of Portsmouth, Portsmouth, United Kingdom(Venter, Maslin, Dean, Arshad) David Hide Asthma and Allergy Research Centre, Newport-Isle-of-Wight, United Kingdom(Arshad) Clinical and): e40.
201. Venter C, Palumbo MP, Sauder KA, et al. Incidence and timing of offspring asthma, wheeze, allergic rhinitis, atopic dermatitis, and food allergy and association with maternal history of asthma and allergic rhinitis. *World Allergy Organization Journal* 2021; **14**(3): 100526.
202. Venter C, Pickett-Nairne K, Leung D, et al. Maternal allergy-preventive diet index, offspring infant diet diversity, and childhood allergic diseases. *Allergy* 2024; **79**(12): 3475-88.
203. Verhoeven DHJ, Herpertz ICEM, Hol J, et al. Reactions to peanut at first introduction in infancy are associated with age≥ 8 months and severity of eczema. *Pediatric Allergy and Immunology* 2023; **34**(6): ei13983.
204. Vernon N, Jerschow E, Jariwala S, De Vos G, Rosenstreich D. Relationship between environmental phenols and aeroallergen and food allergies in the US: Results from the national health and nutrition examination survey 2005-2006. *Journal of Allergy and Clinical Immunology* 2012; **129**(2 SUPPL. 1): AB204.

205. Venkataraman D, Soto-Ramírez N, Kurukulaaratchy RJ, et al. Filaggrin loss-of-function mutations are associated with food allergy in childhood and adolescence. *Journal of Allergy and Clinical Immunology* 2014; **134**(4): 876-82.
206. Wang Y, Allen KJ, Suaini NHA, Peters RL, Ponsonby A-L, Koplin JJ. Asian children living in Australia have a different profile of allergy and anaphylaxis than Australian-born children: A State-wide survey. *Clinical and Experimental Allergy* 2018; **48**(10): 1317-24.
207. Wärnberg Gerdin S, Lie A, Asarnoj A, et al. Impaired skin barrier and allergic sensitization in early infancy. *Allergy* 2022; **77**(5): 1464-76.
208. Wen X, Martone GM, Lehman HK, et al. Frequency of infant egg consumption and risk of maternal-reported egg allergy at 6 years. *The Journal of Nutrition* 2023; **153**(1): 364-72.
209. West CE, Dunstan J, McCarthy S, et al. Associations between maternal antioxidant intakes in pregnancy and infant allergic outcomes. *Nutrients* 2012; **4**(11): 1747-58.
210. Wetzig H, Schulz R, Diez U, Herbarth O, Viehweg B, Borte M. Associations between duration of breast-feeding, sensitization to hens' eggs and eczema infantum in one and two year old children at high risk of atopy. *International journal of hygiene and environmental health* 2000; **203**(1): 17-21.
211. Winters A, Bahnson HT, Ruczinski I, et al. The MALT1 locus and peanut avoidance in the risk for peanut allergy. *Journal of Allergy and Clinical Immunology* 2019; **143**(6): 2326-9.
212. Wright AL, Stern DA, Halonen M. The association of allergic sensitization in mother and child in breast-fed and formula-fed infants. *Advances in experimental medicine and biology* 2001; **501**(0121103, 2lu): 249-55.
213. Yang M, Tan M, Wu J, et al. Prevalence, characteristics, and outcome of cow's milk protein allergy in Chinese infants: a population-based survey. *Journal of Parenteral and Enteral Nutrition* 2019; **43**(6): 803-8.
214. Yuenyongviwat A, Koosakulchai V, Treepaiboon Y, Jessadapakorn W, Sangsupawanich P. Risk factors of food sensitization in young children with atopic dermatitis. *Asian Pacific Journal of Allergy and Immunology* 2024; **42**(1): 30-5.
215. Zeng G-Q, Luo J-Y, Huang H-M, et al. Food allergy and related risk factors in 2540 preschool children: an epidemiological survey in Guangdong Province, southern China. *World Journal of Pediatrics* 2015; **11**(3): 219-25.
216. Zhou C, Ibanez G, Miramont V, et al. Prenatal maternal depression related to allergic rhinoconjunctivitis in the first 5 years of life in children of the EDEN mother-child cohort study. *Allergy & Rhinology* 2017; **8**(3): ar-2017.
