# Supplementary material for: Risk Factors for the Development of Food Allergy in Infants and Children: A Systematic Review and Meta-Analysis
Source: JAMA Pediatr. 2026 Feb 9;180(5):486–99. doi: 10.1001/jamapediatrics.2025.6105 (PMC12887841; doi:10.1001/jamapediatrics.2025.6105)
Supplement: Supplement 2. — Data Sharing Statement [file jamapediatr-e256105-s002.pdf]

Data Sharing Statement

Islam. Risk Factors for the Development of Food Allergy in Infants and Children. *JAMA Pediatr*. Published February 09, 2026. doi:10.1001/jamapediatrics.2025.6105

Data
Data available: No

Additional Information
Explanation for why data not available: The available data are shared within the presented figures, tables, and supplement, and source data can be derived from the included publications.
